# Understanding the role of ring strain in β-alkyl migration at Mg and Zn centres†

**DOI:** 10.1039/d2sc06288g

**Published:** 2023-01-10

**Authors:** Joseph M. Parr, Andreas Phanopoulos, Aaranjah Vickneswaran, Mark R. Crimmin

**Affiliations:** a Molecular Sciences Research Hub, Department of Chemistry, Imperial College London 82 Wood Lane, White City, Shepherds Bush London W12 0BZ UK m.crimmin@imperial.ac.uk

## Abstract

The activation of C–C σ-bonds within strained three- and four-membered hydrocarbons at electrophilic Mg and Zn centres is reported. This was achieved in a two-step process involving (i) hydrometallation of a methylidene cycloalkane followed by (ii) intramolecular C–C bond activation. While hydrometallation of methylidene cyclopropane, cyclobutane, cyclopentane and cyclohexane occurs for both Mg and Zn reagents, the C–C bond activation step is sensitive to ring size. For Mg, both cyclopropane and cyclobutane rings participate in C–C bond activation. For Zn, only the smallest cyclopropane ring reacts. These findings were used to expand the scope of catalytic hydrosilylation of C–C σ-bonds to include cyclobutane rings. The mechanism of C–C σ-bond activation was investigated through kinetic analysis (Eyring), spectroscopic observation of intermediates, and a comprehensive series of DFT calculations, including activation strain analysis. Based on our current understanding, C–C bond activation is proposed to occur by a β-alkyl migration step. β-Alkyl migration is more facile for more strained rings and occurs with lower barriers for Mg compared to Zn. Relief of ring strain is a key factor in determining the thermodynamics of C–C bond activation, but not in stabilising the transition state for β-alkyl migration. Rather, we ascribe the differences in reactivity to the stabilising interaction between the metal centre and the hydrocarbon ring-system, with the smaller rings and more electropositive metal (Mg) leading to a smaller destabilisation interaction energy as the transition state is approached. Our findings represent the first example of C–C bond activation at Zn and provide detailed new insight into the factors at play in β-alkyl migration at main group centres.

## Abstract 2

The activation of C–C σ-bonds within strained three- and four-membered hydrocarbons at electrophilic Mg and Zn centres is reported.

## Graphical abstract

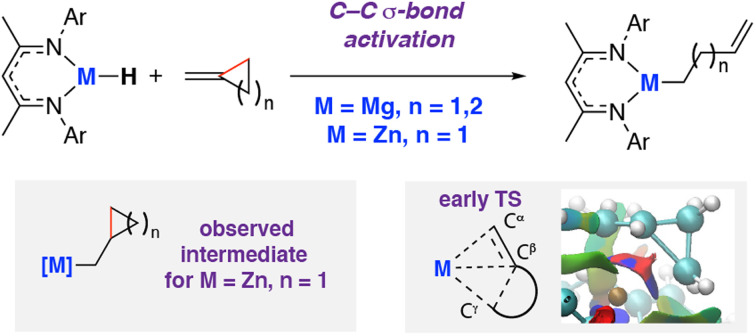

## Introduction

Reactions that break strong carbon–carbon bonds are of broad importance. For example, C–C bond activation is emerging as a fundamental transformation that can be used to achieve skeletal modification of organic molecules, offering a novel approach for synthetic disconnections.^1,2^ Reactions that break C–C bonds also underpin our global energy sector. Catalytic cracking of C–C bonds in hydrocarbon feedstocks, such as crude oil, converts high molecular weight alkanes to more valuable alkenes and medium-length hydrocarbons (*e.g.* C_7_ to C_9_ alkanes). Breaking C–C bonds is, unsurprisingly, very challenging. C–C bonds are ubiquitous but strong and non-polar. The bonding orbitals associated with C–C bonds are often sterically congested, making them kinetically inaccessible. Take for instance, the bonds found in alkanes: the C–C bond of CH_3_–CH_3_ has a homolytic bond dissociation energy of 90.1 ± 0.1 kcal mol^−1^.^3^ Chemoselectivity further complicates C–C bond activation, with surrounding C–H bonds often the first sites to react.

**Fig. 1:**
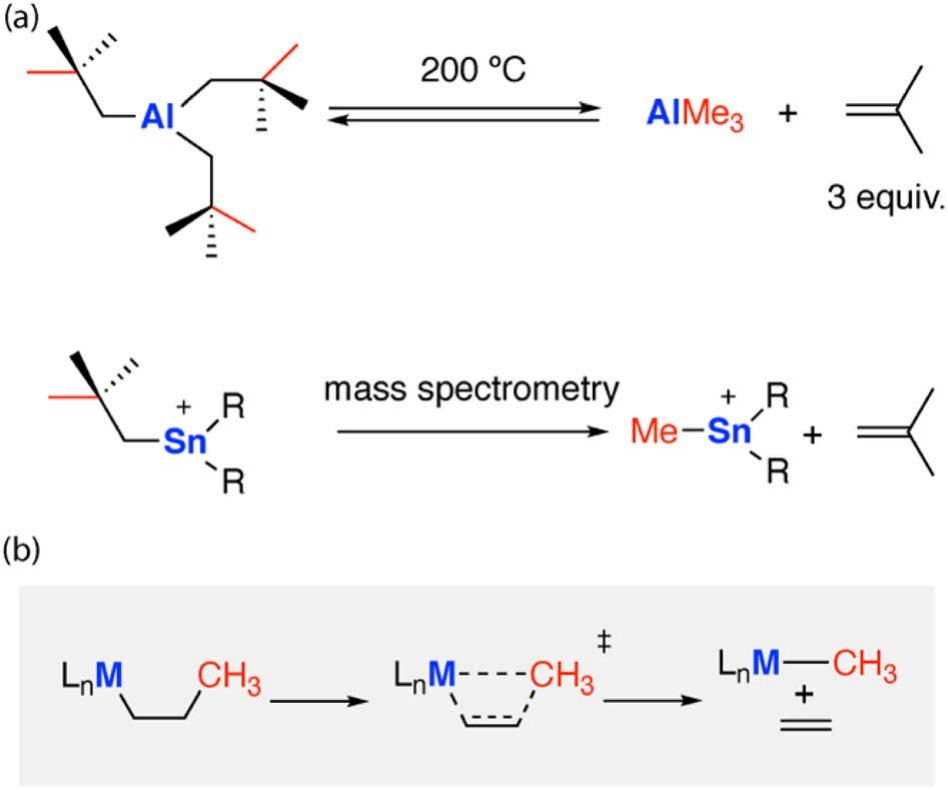
(a) Carbon–carbon bond activation *via* β-alkyl elimination at main-group centres. (b) β-Alkyl elimination mechanism.

C–C bond activation has been achieved on the surface of heterogenous catalysts,^4^ within the active sites of enzymes,^5^ and under homogenous conditions using metal complexes.^6,7^ Reported reactivity typically employs a late transition metal (Rh, Ir, Pd, Pt) as part of the active site for C–C activation.^8^ Reactivity often follows classical mechanisms, divided into two broad classes: (i) the direct oxidative addition of the transition metal to the C–C bond;^9^ (ii) β-alkyl elimination at a metal centre (Fig. 1);^10^ β-alkyl migration can be defined as a sub-class of β-alkyl elimination, wherein the substrate remains intact on the transition metal throughout the pathway.

There is an urgent requirement for more sustainable approaches to this fundamental transformation through replacement of the rare and expensive transition metals often used for bond breaking. To that end, main-group metals have been investigated toward C–C activation. Main-group metals are light, non-toxic alternatives that are abundant and readily accessible; common examples include Al, Mg, and Zn. Though limited, some notable contributions toward main-group C–C bond activation have been reported.

One of the first examples of main group C–C activation was proposed to occur through a β-alkyl migration mechanism. The thermolysis of tris(neo-pentyl)aluminium [Al{CH_2_C(Me)_3_}_3_] at high temperatures (200 °C) led to the reversible formation of iso-butene and trimethylaluminium, *via* sp^3^ C–C bond activation.^11^ The release of iso-butene gas provides an entropic driving force for this reaction. A related reaction of the tris(neopentyl)stannyl cation [Sn{CH_2_C(Me)_3_}_3_]^+^ has been proposed to occur during fragmentation in mass spectrometry measurements (Fig. 1).^12^ Related migration processes likely underpin rearrangements of strained polycyclic systems which incorporate main group elements. For example, the addition of aluminium nucleophiles to the π-system of biphenylene^13–15^ and naphthalene^14^ creates strained bicyclic motifs that undergo skeletal rearrangements to break strong C–C bonds. Low-valent main group complexes have also been proposed to mediate C–C bond activation of arenes through a Büchner ring-expansion mechanism, this involves a stepwise [2 + 2] cycloaddition to form a nocaradiene-type intermediate followed by an electrocyclic ring-opening.^16^ Ring-opening of cyclopropanes by C–C bond activation has been reported using frustrated Lewis-pairs.^17^

**Scheme 1:**
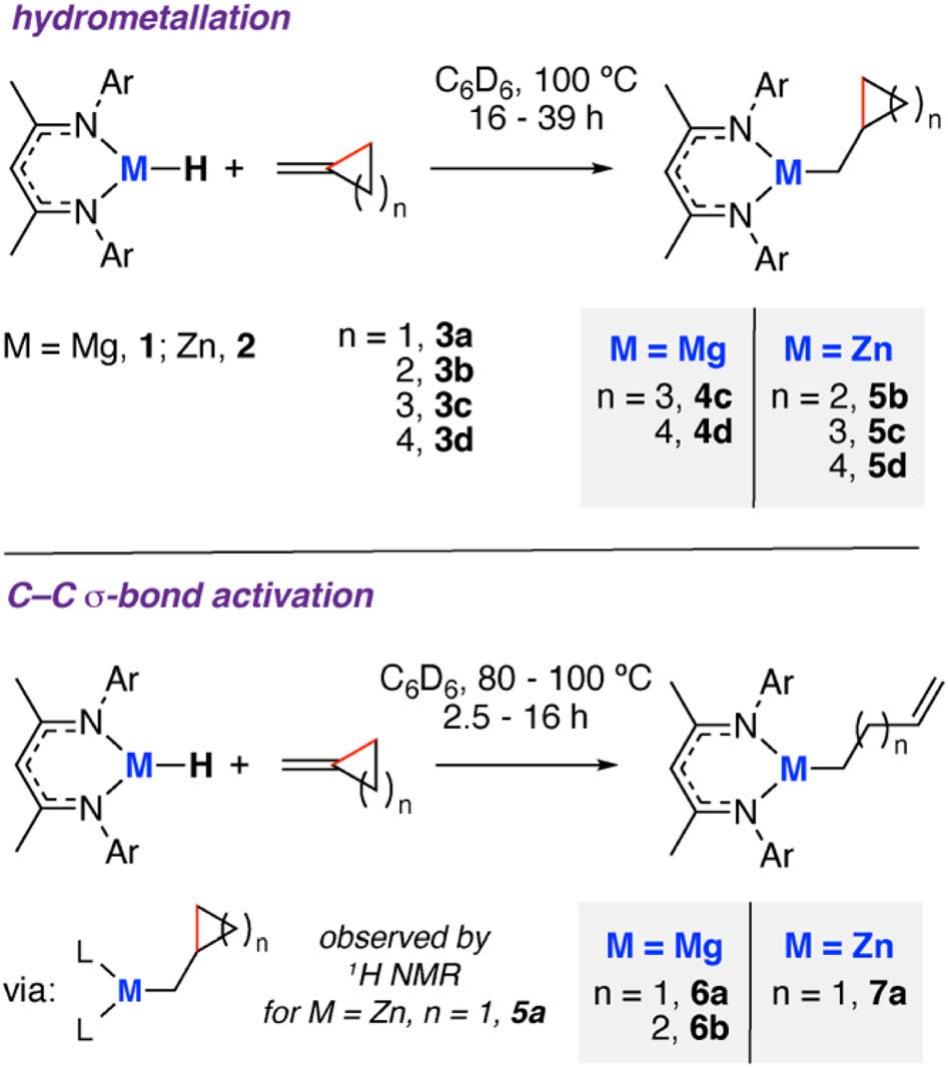
Reaction of methylidene cycloalkanes with 1 and 2. Ar = 2,6-di-iso-propylphenyl.

As part of an investigation of C–C bond activation at main group centres, we recently communicated an example of β-alkyl migration at a Mg centre.^18^ The reaction of a β-diketiminate stabilised magnesium hydride (1)^19^ with methylidene cyclopropane (3a) resulted in the formation of the ring-opened but-4-en-1-ylmagnesium complex (Scheme 1). Here, we report a comprehensive investigation of this system. We expand the scope to a series of four- to six-membered methylidene cycloalkanes (3b–d) and show, for the first time, that analogous Zn complexes are capable of C–C bond activation. Kinetic analysis and DFT studies support a stepwise mechanism involving first hydrometallation of the C=C bond of the substrate, following by ring-opening by a β-alkyl migration mechanism. We show that both the nature of the metal and ring size impacts the relative rate of key C–C bond activation step.

## Results and discussion

### Hydrometallation of methylidene cycloalkanes

The reaction of 1 with compounds containing 4–6 membered methylidene cycloalkane rings (3b–d) was investigated (Scheme 1). For the substrates containing 5 and 6-membered rings after heating at 100 °C overnight, hydromagnesiated products 4c and 4d and were isolated in high yields (>80%). The reaction occurs exclusively with anti-Markovnikov addition of the Mg–H bond of 1 to the alkene. The products were characterised by a diagnostic Mg–CH_2_ unit in the ^1^H NMR spectrum in d_6_-benzene solution, *δ*_H_ = −0.07 and −0.18 ppm for 4c and 4d respectively. Corresponding methylene resonances were observed in the ^13^C NMR spectrum at *δ*_C_ = 13.7 and 17.7 ppm and confirmed by 2D-experiments. 4c has been prepared previously, and the spectroscopic data matches that reported by Hill and co-workers.^20^

The study was expanded to include reaction of corresponding zinc hydride complex 2 with methylidene cycloalkanes (Scheme 1).^21^ In all cases, addition of 2 to 3b–d resulted in analogous reactivity and insertion of the alkene into the Zn–H bond. Reaction of 2 with 3b, 3c or 3d at 100 °C for 39 h yielded single alkene insertion products in high selectivity and good isolated yields (>70%). Again the products were characterised by a diagnostic Zn–CH_2_ unit in the ^1^H NMR spectrum in d_6_-benzene solution at *δ*_H_ = 0.48, 0.40 and 0.27 ppm or 5b, 5c, and 5d respectively. Related hydrozincation reactions of acyclic alkenes with 2 have recently been reported by our group.^22^

**Fig. 2:**
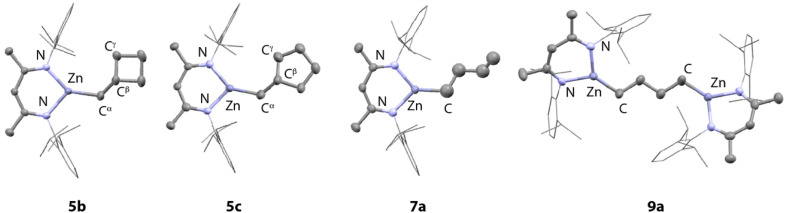
Structures of 5b, 5c, 7a and 9a from single crystal X-ray diffraction experiments. Selected bond lengths (Å) and angles (°): 5b: Zn1–N1 1.9797(13), Zn1–N2 1.9554(12), Zn1–C30 1.9697(18), C30–C31 1.504(3), N1–Zn1–N2 95.86(5), N1–Zn1–C30 124.04(8), C30–Zn1–N2 140.06(8); 5c: Zn1–N1 1.9745(3), Zn1–N2 1.975(3), Zn1–C30 1.965(4), C30–C31 1.475(7), N1–Zn1–N2 95.52(11), N1–Zn1–C30 141.81(15), C30–Zn1–N2 122.56(15); 7a: Zn1–N1 1.956(2), Zn1–N2 1.964(2), Zn1–C30 1.960(3), C32A–C33A 1.157(9), N1–Zn1–N2 95.83(9), N1–Zn1–C30 131.84(10), C30–Zn1–N2 132.26(10); 9a: Zn1–N1 1.9651(16), Zn1–N2 1.9595(17), Zn1–C59 1.945(2), Zn2–N3 1.9589(17), Zn2–N4 1.9709(19), Zn2–C62 1.943(3), N1–Zn1–N2 95.47(8), N1–Zn1–C59 130.33(9), C59–Zn1–N2 134.21(8), N3–Zn2–N4 95.37(17), N3–Zn2–C62 134.54(9), C62–Zn2–N4 130.07(9).

Crystals of 5b and 5c suitable for single crystal X-ray diffraction were grown from *n*-pentane solution (Fig. 2). Both structures show the expected three-coordinate geometry at zinc supported by the β-diketiminate ligand and a hydrocarbon fragment that contains the intact carbocycle. The bond lengths to Zn are consistent with related structures and largely unremarkable.^23–27^ The sum of the N–Zn–N, N–Zn–C, and C–Zn–N bond angles for both is close to 360° for both 5b (359.96(12) °) and 5c (359.99(24) °) suggestive of sp^2^ hybridisation and coordinative unsaturation at the metal. The nearest approach of the centroid of the C^β^–C^γ^ bond to the metal is beyond 3 Å and does not occur in a way this moiety approaches a proposed vacant site at Zn perpendicular to the plane of the β-diketiminate ligand.

Kinetic measurements were undertaken to better understand the hydrometallation step. Eyring analysis was conducted over a temperature range 70–100 °C. Kinetic data for the addition of 1 to 3c was modelled as first order in 1. The activation parameters for hydromagnesiation were found to be: Δ*H*^‡^ = 15.0 ± 2.0 kcal mol^−1^, Δ*S*^‡^ = −34.4 ± 14.7 cal K^−1^ mol^−1^ and an associated Δ*G*_(298 K)_^‡^ = 25.2 ± 2.0 kcal mol^−1^. Activation parameters for the closely related step of alkene insertion at transition metal centres have been derived from NMR studies. For comparison, intramolecular alkene insertion of [(Cp*)_2_Nb(H)(η^2^-CH_2_=CH_2_)] has been determined to occur with Δ*H*^‡^ = 14.7 ± 1.0 kcal mol^−1^ and Δ*S*^‡^ = −11.2 ± 2.9 cal K^−1^ mol^−1^.^28^ A related reaction of *cis*-[Rh(P^i^Pr_3_)_2_(H)(η^2^-CH_2_=CH_2_)] proceeds with Δ*H*^‡^ = 13.0 kcal mol^−1^ and Δ*S*^‡^ = −2.0 cal K^−1^ mol^−1^.^29^ Although both processes occur with similar activation enthalpies, the entropy of activation of hydromagnesiation is large and negative, suggestive of a substantial increase in order at the transition state required for intermolecular hydromagnesiation compared to intramolecular alkene insertion at transition metals.

### C–C bond activation

Addition of 1 to methylidene cyclobutene (3b) and heating (60–100 °C) gave a mixture of the ring-opened pent-5-en-1-ylmagnesium compound 6b and double metalation product 8b (*vide infra*). The reaction is proposed to proceed *via* an initial hydrometallation, forming the intermediate 4b. 4b was not observed spectroscopically during the reaction, though DFT calculations support its formation and facile ring-opening through β-alkyl elimination (*vide infra*). Reaction of 1 and 3b at 40–60 °C similarly did not show clear spectroscopic evidence of intermediate 4b. The product of ring-opening 6b was characterised by a diagnostic Mg–CH_2_ unit in the ^1^H NMR spectrum in d_6_-benzene solution, *δ*_H_ = −0.25 ppm (t, ^3^*J*_H–H_ = 8.1 Hz). Heteronuclear Single Quantum Coherence (HSQC) experiments were used to assign the alkene resonances at *δ*_H_ = 4.74–4.87 and 5.76 ppm, and *δ*_C_ = 113.3 and 141.8 ppm. The infrared spectra for 6b shows a characteristic *ν*(C=C) stretching frequency at 1648 cm^−1^. A labelling study using a Mg–D isotopomer of 1 confirmed that the deuteride is exclusively observed at the internal alkene CH position in 6b (see ESI†). We have previously reported ring-opening of methylidene cyclopropane (3a) with 1 to form 6a.^18^ Hence, with these Mg-reagents C–C bond activation of 3- and 4-membered hydrocarbon rings is possible, but not larger 5 or 6-membered cycloalkanes.

Addition of 2 to 3a also leads to C–C bond activation and formation of the ring-opened product 7a. In this case, there is spectroscopic support for the formation of the hydrometallated intermediate 5a by a series of diagnostic resonances in the high-field region of the ^1^H NMR spectrum. Multiplets at −0.39 to −0.36 ppm (2H) and 0.15–0.20 ppm (2H) can be assigned as the diastereotopic ring CH_2_ groups (both resonances are bound to the same carbon environment by HSQC), while a multiplet at 0.35–0.43 ppm (1H) is assigned as the bridgehead CH group. The CH_2_ group adjacent to the Zn centre is observed as a doublet centred at 0.24 ppm (*J* = 7.2 Hz). Selective excitation TOCSY experiments confirmed all the resonances were part of the same coupled spin system. The final reaction product 7a was unambiguously characterised by multinuclear NMR and single crystal X-ray diffraction (Fig. 2), along with infrared spectroscopy which shows a characteristic *ν*(C=C) stretching frequency at 1633 cm^−1^. Even at elevated temperature C–C bond activation of 4-, 5- and 6-membered hydrocarbons was not observed with Zn. From these experiments, we can suggest that the scope of reactivity is broader for Mg compared to Zn and, given that the monomeric units of 1 and 2 are isostructural, that C–C bond activation is more facile with the more electropositive metal magnesium.

### Double metallation

In the presence of a second equivalent of the main group hydride, the products of β-alkyl migration are not stable. Addition of 1 to the ring-opened pent-5-en-1-ylmagnesium complex 6b resulted in a second anti-Markovnikov hydromagnesiation reaction, yielding the 1,5-dimagnesio pentane 8b.^18,30^ Similarly a second hydrozincation of 7a yields the 1,4-dizincio butane complex 9a (Fig. 2).

### Catalytic hydrosilylation

**Fig. 3:**
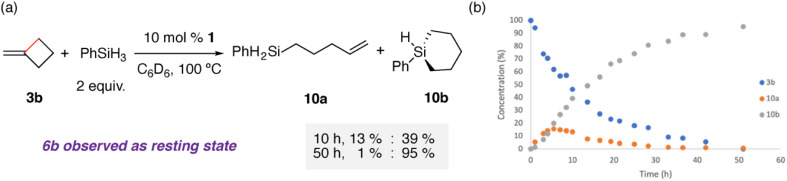
(a) Catalytic hydrosilylation of 3b with 1, NMR yields for key time points noted; (b) reaction progress plot for catalytic hydrosilylation of 3b with 1 at 100 °C.

A catalytic reaction of 3b, two equiv. of phenylsilane, and 10 mol% 1, led to the formation of a mixture of the hydrosilylated products 10a and 10b in near quantitative yield (Fig. 3). The reaction did not proceed in the absence of 1. Following the reaction as a function of time strongly suggests that 10a is a kinetic product that undergoes a second intramolecular hydrosilylation to give the 1-phenyl-1-silacyclohexane 10b. 10b could be isolated following column chromatography in 83% yield. 6b was observed spectroscopically during the reaction procedure, supporting a mechanism involving the hydromagnesiation and β-alkyl migration steps identified for addition of 1 to 3b in the absence of silane. Catalytic hydrosilyation of 3a with 2 yielded only stoichiometric formation of the acyclic hydrosilylation product Ph_2_Si(H)(CH_2_CH_2_CH=CH_2_), suggesting that catalytic turnover is more challenging for Zn compared with Mg.

### DFT calculations

DFT calculations using the ωB97X-D^31^ functional were used to gain further insight into the hydrometallation and β-alkyl migration step. A model was developed to explain the effect of both the cycloalkane ring-size and nature of the metal on reactivity. Methods were benchmarked against the solid state structures and the activation parameters from kinetics data (*vide supra*).^32,33^ Metal atoms (Mg, Zn) were described with Stuttgart SDDAll RECPs and associated basis sets, while a hybrid basis set was used for the other atoms: 6-31g**(C, H)/6-311+g*(N).^34–36^ Complete potential energy surfaces for reactions of 1 and 2 with 3a–d, along with functional testing and computational details, can be found in the ESI.†

**Fig. 4:**
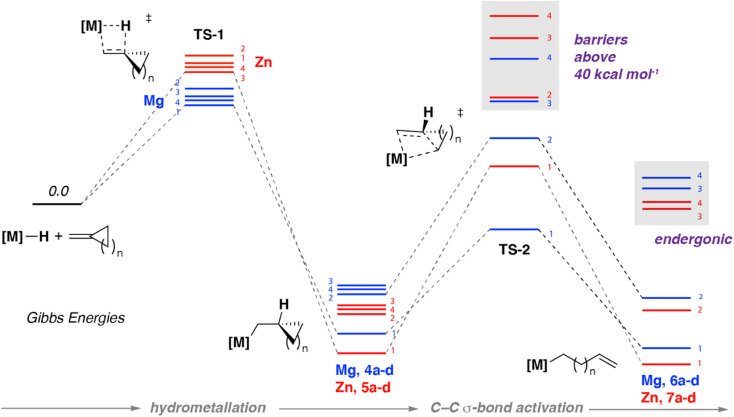
Calculated reaction pathway for hydrometallation and β-alkyl migration (ωB97XD). Each stationary point is marked with the value of *n* (=1, 2, 3, 4) for hydrocarbon ring size and coded for Mg (blue) and Zn (red).

Hydrometallation of methylidene cycloalkanes with either 1 or 2 both proceed *via* a four membered transition state TS-1 (Fig. 4, Table 1). No clear trend between ring size and activation barrier was noted. The nature of the main-group metal was determined to be the most important factor in determining the activation barrier for hydrometallation. Gibbs activation energies for the hydrometallation transition state TS-1 ranged from 21.7–24.0 kcal mol^−1^ for Mg, and 30.7–32.5 kcal mol^−1^ for Zn (Table 1). These calculations are consistent with higher temperatures and longer reaction times observed experimentally upon reaction of 2 with methylidene cycloalkanes compared to 1. In all cases, the hydrometallation step is exergonic with the most energetically stable products being those in which there is relief of strain due to sp^2^ to sp^3^ rehybridisation on hydrometallation. For example, the difference in strain energy between methylidene cyclopropene and cyclopropane can be estimated as 13.4 kcal mol^−1^.^37^

Calculated local barriers for hydrometallation of methylidene cycloalkanes and β-alkyl migration at Mg and Zn. Gibbs energies values in kcal mol^−1^

**Table:** 

M = Mg	Hydrometallation		β-Alkyl migration	
*N*	TS-1	4a–d	TS-2	6a–d
1	21.7	−24.7	19.0	−3.1
2	24.0	−15.6	28.0	−0.5
3	22.4	−14.8	**40.7**	**19.0**
4	22.2	−15.5	**53.6**	**23.4**

**Table:** 

M = Zn	Hydrometallation		β-Alkyl migration	
*N*	TS-1	5a–d	TS-2	7a–d
1	32.2	−27.6	35.2	−2.8
2	32.5	−18.2	**44.4**	0.8
3	30.7	−16.8	**56.7**	**19.4**
4	30.9	−17.5	**70.1**	**23.0**

Natural Bond Orbital (NBO) analysis of the calculated stationary points for the hydrometallation step were undertaken (Tables S6 and S7†). Analysis of the Wiberg Bond Index (WBI) on stationary points across the pathway shows the expected decrease in C=C bond strength that occurs with loss of the unsaturated bond in 3a–b upon addition of 1 or 2. Take addition of 1 to 3b as a representative example; the WBI of the breaking C=C bond decreases from 3b → TS-1 → 5b (1.94 → 1.50 → 1.03). A concomitant increase in calculated bond length is observed as expected (1.32 → 1.39 → 1.52 Å).

In all cases, C–C σ-bond activation was calculated to proceed *via* a concerted intramolecular transition state TS-2 best described as a β-alkyl migration step (Fig. 4, Table 1). For example, C–C activation of the cyclopropane ring in 3a with 1 is calculated to occur from intermediate 4a with a modest energy barrier (Δ*G*^‡^ = 19.0 kcal mol^−1^) *via*TS-2, forming the isolable complex 6a. Comparison of this energy barrier with that calculated for hydromagnesiation *via*TS-1 (Δ*G*^‡^ = 21.7 kcal mol^−1^) is consistent with the lack of spectroscopic observation of the intermediate 4a during the reaction course.

The computational model can be used to explain the effects of both ring-size and influence of metal on the β-alkyl migration step. The activation barriers for β-alkyl migration increase with increasing ring size of cycloalkane ring *i.e.* 3 < 4 < 5 < 6. In all cases these barriers are higher for Zn then they are for Mg. Hence, while β-alkyl migration for 3-membered ring systems is just about accessible for both metals (Mg, Δ*G*^‡^ = 19 kcal mol^−1^; Zn, Δ*G*^‡^ = 35.2 kcal mol^−1^), for 4-membered rings only the Mg analogue is predicted to facilitate β-alkyl migration under the experimental conditions (Mg, Δ*G*^‡^ = 28.0 kcal mol^−1^; Zn, Δ*G*^‡^ = 44.4 kcal mol^−1^). For both metals, β-alkyl migration of substrates containing five- and six-membered systems by TS-2 have energy barriers that are inaccessible under the reaction conditions (Δ*G*^‡^ > 40 kcal mol^−1^). Furthermore, the overall ring opening process for larger ring sizes are calculated to be endergonic, so would be expected to be reversible even if the activation barriers were traversed. The ring strain energies of methylidene cyclopropane, cyclobutene, cyclopentane and cyclohexane have been determined as 40.9, 26.9, 6.1, and −1.1 kcal mol^−1^ respectively.^38^ Our calculations are consistent with relief of ring-strain as a definitive factor in driving the thermodynamics of C–C bond activation.

**Fig. 5:**
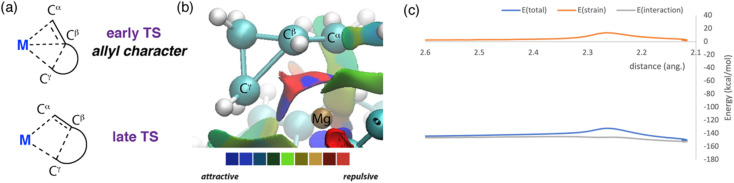
(a) Depiction of allyl like ligand binding to the metal centre. (b) NCI plot for TS-2 (M = Mg, *n* = 1). (c) activation strain analysis (ASA): TS-2 (M = Mg, *n* = 1) along the Mg–C_γ_ bond. ASA plots for complete TS-2 series can be found in the ESI (Table S8, Fig. S19–S36†).

The C–C σ-bond activation step can be understood in more detail through further consideration and analysis of transition state. In all cases, breaking of the M–C_α_, and making of the M–C_γ_ bond accompanies C_β_–C_γ_ bond activation. As a result, the C_α_=C_β_⋯C_γ_ motif adopts an electronic structure reminiscent of an allyl ligand in the TS (Fig. 5a). For example, TS-2 when M = Mg and *n* = 1 has a short C_α_=C_β_ bond length of 1.42 Å, stretched C_β_–C_γ_ bond of 1.94 Å, and even, near symmetric, Mg–C_α_ and Mg–C_γ_ distances of 2.22 and 2.26 Å respectively. Charge localisation occurs across the allyl-like moiety, but primarily at the terminal carbons, C_α_ (−1.01) and C_γ_ (−0.94), rather than C_β_ (−0.18). AIM calculations capture bond paths between both C_α_ and C_γ_ with Mg, but not between C_β_ and C_γ_ in the breaking bond. Wiberg bond indices from NBO provide a similar picture of electron distribution in this TS, with partial double bond character in the C_α_=C_β_ unit (1.42) and a weakened C_β_⋯C_γ_ bond (0.57). Second order perturbation from the NBO calculations show donation of electron density from the C_α_ atom (35.2 kcal mol^−1^), C_β_–C_γ_ bond (17.5 kcal mol^−1^), C_α_–C_β_ bond (2.6 kcal mol^−1^) and C_γ_ atom (1.4 kcal mol^−1^) to Mg. An NCI plot further supports the proposed allyl character of the hydrocarbon ligand, as it shows attractive interactions between each carbon atom and the magnesium site (Fig. 5b).

### Activation strain analysis

While relief of ring strain is clearly an important thermodynamic driving force for these reactions, it is not immediately clear if this is also a factor in determining the kinetics of C–C bond activation. Activation Strain Analysis (ASA) was performed to address this question.^39–41^ This approach interrogates energy changes on the potential energy surface around the transition state, at the simplest level deconvoluting the total energy of the system into the interaction energy Δ*E*_int_(*ζ*) between two fragments and the strain energy Δ*E*_strain_(*ζ*) required to distort said fragments along the potential energy surface. Typically, ASA is applied to bimolecular reactions, although there are examples of unimolecular systems where this approach has provided useful insight.^42,43^ In intramolecular cases, the choice of fragments has been proposed to be crucial in obtaining meaningful information on the factors that impact reactivity. Here the interaction of the cycloalkane fragment (*e.g.* C_4_H_7_ or C_5_H_9_) with the metal fragment [M{(NArCMe)_2_CH}] was considered (M = Mg, Zn; Ar = 2,6-di-iso-propylphenyl). Hence, our ASA calculations should provide insight into the strain required to distort each of these fragments to the TS geometry, and the difference in interaction energies (*i.e.* the binding energy) between the metal and the hydrocarbon ligand as the TS is approached.

Activation strain analysis on the potential energy surface associated with β-alkyl migration transition state TS-2 suggests that there is only a slight difference in the contribution from strain energy in these two reactions (Fig. 5c). For both systems, the reaction strain energy Δ*E*_strain_(*ζ*) reaches a maximum at the transition state. For Mg, there is little difference in the strain energy, ΔΔ*E*_strain_‡ from reactant to transition state, TS-2 (*n* = 1, 12.0 kcal mol^−1^; *n* = 2, 11.9 kcal mol^−1^). Nearly all the contribution to the strain energy is from distortion of the hydrocarbon fragment. On further inspection of the transition state geometries this conclusion becomes more intuitive, there are only small distortions in bond angles in the ring systems of these structures when compared to the parent cycloalkanes. Hence, relief of ring strain does not occur at the transition state but rather later along the reaction coordinate as the system progresses toward the products. The interaction energy Δ*E*_int_(*ζ*) is negative and relatively flat across the reaction coordinate. This is perhaps expected, as the calculated data points describe a concerted bond breaking and bond making event in which stabilising interactions from forming bonds are counterbalanced by destabilising interactions from breaking bonds. From reactant to transition state, the change in interaction energy is positive, suggestive of a less stabilising interaction between the metal site and hydrocarbon fragment as the TS is approached. For the Mg system, comparing ΔΔ*E*^‡^_int_ for TS-2 (*n* = 1, 1.1 kcal mol^−1^; *n* = 2, 7.7 kcal mol^−1^) suggests that the origin of the higher activation energy for 4-membered *vs.* 3-membered rings can be traced to a weaker binding of the hydrocarbon to the metal. Based on these calculations, it can be speculated that more facile activation of the smaller ring sizes is not a consequence of relief of ring strain in the transition state, but rather a more stabilising interaction of the allyl type ligand with the metal at the TS.

Polarisation and charge delocalisation across the C_α_=C_β_⋯C_γ_ moiety may also play a role in the differences in reactivity for Mg and Zn. Transition states for C–C bond activation are calculated to be higher in energy for Zn than for Mg across the whole series. For Zn, activation strain analysis again suggests that there is little difference in the relief of ring strain on going from reactant to the transition state ΔΔ*E*^‡^_strain_ for TS-2 (*n* = 1, 11.1 kcal mol^−1^; *n* = 2, 12.9 kcal mol^−1^). Rather the interaction energy is again the key factor at play. ΔΔ*E*^‡^_int_ for TS-2 (*n* = 1, 8.1 kcal mol^−1^; *n* = 2, 19.7 kcal mol^−1^) is less destabilising for the smaller ring size. In addition, ΔΔ*E*^‡^_int_ values for TS-2 for both ring sizes are less destabilising for Mg compared to Zn. The Mg site is both larger and more electropositive than Zn (*r*_cov_ Mg = 1.39, Zn = 1.18 Å; *χ*_p_ Mg = 1.31, Zn = 1.65). As such it is likely better placed to polarise the C_α_=C_β_⋯C_γ_ moiety and stabilise the resultant fragment through electrostatic interactions with all three carbon centres. The Zn atom is less well suited to accommodate coordination of all three carbon atoms involved in C–C bond activation. This supposition is supported by the calculated TS geometries for Zn which show a greater asymmetry in the Zn–C_α_ and Zn–C_γ_ distances compared to Mg analogues. For example, in TS-2 (*n* = 1) there is a *Δ* = 0.22 Å difference in these values for Zn, whereas it is only *Δ* = 0.05 Å for Mg. We speculate these differences make an important contribution to raising the energy of the C–C bond activation step for Zn relative to Mg.

## Conclusions

In summary, C–C σ-bond activation of strained cyclic hydrocarbons has been observed to occur at both Mg and Zn metal centres. While ring-opening of both 3- and 4-membered rings was observed with Mg reagents, isostructural Zn species were only capable of reacting with smaller 3-membered rings. DFT calculations, benchmarked against the experiment data and activation parameters from kinetics, support a mechanism for C–C σ-bond activation involving β-alkyl migration. While relief of ring strain provides a thermodynamic driving force for β-alkyl migration, activation strain analysis suggests that it does not lower the barrier for breaking the C–C σ-bond by an appreciable amount. Rather, the interaction energy between the metal and the hydrocarbon fragment at the transition state is likely the key contributor to determining the kinetic barrier to β-alkyl migration. This is more favourable for smaller ring sizes (3 *vs.* 4) and for the more electropositive metal (Mg *vs.* Zn). In combination, the analysis suggests the β-alkyl migration at main group centres should become more favourable for systems in which the metal can polarise the hydrocarbon fragment, and the hydrocarbon fragment can best accommodate and delocalise the associated charge.

## Data availability

Experimental procedures, details of the calculations, and additional data can be found in the ESI (.pdf).† X-ray data is available in .cif format.

## Author contributions

JP, AP, and AV conducted experimental work. JP conducted all computational work. AP solved and refined all single crystal XRD data. JP, AP, and MRC wrote the manuscript. All authors agreed on the final version of the manuscript.

## Conflicts of interest

There are no conflicts to declare.

## Supplementary Material

SC-014-D2SC06288G-s001

SC-014-D2SC06288G-s002

SC-014-D2SC06288G-s003

## References

[cit1] Wang B., Perea M. A., Sarpong R. (2020). Angew. Chem., Int. Ed..

[cit2] Murakami M., Ishida N. (2016). J. Am. Chem. Soc..

[cit3] Blanksby S. J., Ellison G. B. (2003). Acc. Chem. Res..

[cit4] Benitez V. M., Grau J. M., Yori J. C., Pieck C. L., Vera C. R. (2006). Energy Fuels.

[cit5] Guengerich F. P., Yoshimoto F. K. (2018). Chem. Rev..

[cit6] Gozin M., Welsman A., Ben-David Y., Milstein D. (1993). Nature.

[cit7] Jun C. (2004). Chem. Soc. Rev..

[cit8] Xia Y., Lu G., Liu P., Dong G. (2016). Nature.

[cit9] Souillart L., Cramer N. (2015). Chem. Rev..

[cit10] O'Reilly M. E., Dutta S., Veige A. S. (2016). Chem. Rev..

[cit11] Ziegler K. (1956). Angew. Chem..

[cit12] Dakternieks D., Lim A. E. K., Lim K. F. (1999). Chem. Commun..

[cit13] Kong R. Y., Crimmin M. R. (2021). Angew. Chem., Int. Ed..

[cit14] Zhang X., Liu L. L. (2022). Angew. Chem., Int. Ed..

[cit15] Koshino K., Kinjo R. (2020). J. Am. Chem. Soc..

[cit16] Hicks J., Vasko P., Goicoechea J. M., Aldridge S. (2019). J. Am. Chem. Soc..

[cit17] Morton J. G. M., Dureen M. A., Stephan D. W. (2010). Chem. Commun..

[cit18] Kong R. Y., Crimmin M. R. (2020). J. Am. Chem. Soc..

[cit19] Green S. P., Jones C., Stasch A. (2008). Angew. Chem., Int. Ed..

[cit20] Garcia L., Dinoi C., Mahon M. F., Maron L., Hill M. S. (2019). Chem. Sci..

[cit21] Spielmann J., Piesik D., Wittkamp B., Jansen G., Harder S. (2009). Chem. Commun..

[cit22] Baker G. J. , White A. J. P., Casely I. J., Grainger D. and Crimmin M. R., *ChemRxiv*, 2022, preprint, 10.26434/chemrxiv-2022-3tcn2-v2

[cit23] Cheng M., Moore D. R., Reczek J. J., Chamberlain B. M., Lobkovsky E. B., Coates G. W. (2001). J. Am. Chem. Soc..

[cit24] Sarish S. P., Schaffner D., Sun Y., Thiel W. R. (2013). Chem. Commun..

[cit25] Prust J., Stasch A., Zheng W., Roesky H. W., Alexopoulos E., Usón I., Böhler D., Schuchardt T. (2001). Organometallics.

[cit26] Pietrzak T., Justyniak I., Kubisiak M., Bojarski E., Lewiński J. (2019). Angew. Chem., Int. Ed..

[cit27] Cole S. C., Coles M. P., Hitchcock P. B. (2004). Organometallics.

[cit28] Doherty N. M., Bercaw J. E. (1985). J. Am. Chem. Soc..

[cit29] Foley H. C., Decanio S. J., Tau K. D., Chao K. J., Onuferko J. H., Dybowski C., Gates B. C. (1983). J. Am. Chem. Soc..

[cit30] Yuvaraj K., Douair I., Maron L., Jones C. (2020). Chem.–Eur. J..

[cit31] Da Chai J., Head-Gordon M. (2008). J. Chem. Phys..

[cit32] Garçon M., Mun N. W., White A. J. P., Crimmin M. R. (2021). Angew. Chem., Int. Ed..

[cit33] Butler M. J., White A. J. P., Crimmin M. R. (2016). Angew. Chem., Int. Ed..

[cit34] Hehre W. J., Ditchfield R., Pople J. A. (1972). J. Chem. Phys..

[cit35] Hariharan P. C., Pople J. A. (1973). Theor. Chim. Acta.

[cit36] Clark T., Chandrasekhar J., Spitznagel G. W., Schleyer P. v R. (1983). J. Comput. Chem..

[cit37] Wiberg K. B. (1986). Angew. Chem., Int. Ed..

[cit38] Dudev T., Lim C. (1998). J. Am. Chem. Soc..

[cit39] Svatunek D., Houk K. N. (2019). J. Comput. Chem..

[cit40] Vermeeren P., van der Lubbe S. C. C., Fonseca Guerra C., Bickelhaupt F. M., Hamlin T. A. (2020). Nat. Protoc..

[cit41] Bickelhaupt F. M., Houk K. N. (2017). Angew. Chem., Int. Ed..

[cit42] Fernández I., Bickelhaupt F. M., Cossío F. P. (2014). Chem.–Eur. J..

[cit43] Fernández I., Bickelhaupt F. M., Cossío F. P. (2012). Chem.–Eur. J..

