# Supplementary material for: Understanding the role of ring strain in β-alkyl migration at Mg and Zn centres
Source: Chem Sci. 2023 Jan 10;14(6):1590–7. doi: 10.1039/d2sc06288g (PMC9906642; doi:10.1039/d2sc06288g)
Supplement: SC-014-D2SC06288G-s002 [file SC-014-D2SC06288G-s002.pdf]

Electronic Supplementary Material (ESI) for Chemical Science.
This journal is © The Royal Society of Chemistry 2023

Understanding the Role of Ring Strain in b-Alkyl Migration at Mg and Zn Centres

Joseph M. Parr,[a] *Andreas Phanopoulos,*[a] *Aaranjah Vickneswaran,*[a] *Mark R Crimmin**[a]

Corresponding author email: m.crimmin@imperial.ac.uk

[a] Molecular Sciences Research Hub, Department of Chemistry, Imperial College London, 82 Wood Lane, White City, Shepherds Bush, London, W12 0BZ, UK.

Contents

1. General Experimental ***1***

2. Synthetic Procedures ***2***

Synthesis of 4c 2

Synthesis of 4d 3

Synthesis of 5b 7

Synthesis of 5c 11

Synthesis of 5d 15

Synthesis of 6b 19

Synthesis of 7a 25

Synthesis of 8b 30

Synthesis of 9a 34

Labelling reaction 38

Catalytic hydrosilylation with magnesium 39

Hydrosilylation with zinc 41

Kinetics 43

3. X-ray Crystallography ***45***

Crystal structure of 5b 46

Crystal structure of 5c 46

Crystal structure of 7a 48

Crystal structure of 9a 49

4. Computational Calculations ***50***

Computed structures, energies and analysis 51

NBO analysis of select TS-2 61

Activation Strain Analysis 62

Non-covalent interactions (NCI) plots 81

Computational coordinates 83

5. References ***110***

1. General Experimental

All manipulations were carried out under standard Schlenk-line or glovebox techniques under an inert atmosphere of nitrogen. A MBraun Labmaster glovebox was employed operating at concentrations of H_2O and O_2 below 0.1 ppm. Glassware was dried for 12 hours at 120 °C prior to use. C_6D_6 was freeze-pump thaw degassed thrice and stored over molecular sieves for twelve hours before use. All other solvents were dried using a Grubbs type solvent purification system. ^{1}H, COSY, TOCSY, ^{13}C{^{1}H}, DEPT-135, HSQC and HMBC NMR experiments were run within J-Young Tap NMR tubes on Bruker Avance 400 or 500 MHz spectrometers. Spectra were referenced to known residual protio solvent peaks. NMR analysis was conducted in MestReNova and TopSpin with baseline and phase corrections applied to spectra. Chemical shift values are reported in ppm and coupling constants *J* in Hz. Microanalysis (CHN) were performed under inert atmosphere by Elemental Microanalysis Ltd. Infrared spectra were obtained on a Cary630 spectrometer (placed with in an MBraun glovebox) from crystalline solids or toluene thin-films on an ATR cell. **1,**[1] **2,**[2] and **3a**[3] were prepared as described previously. All other reagents were purchased from commercial vendors and used without further purification.

2. Synthetic Procedures

Synthesis of 4c

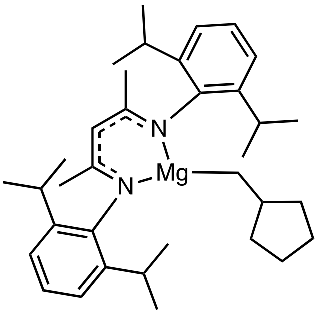

In a N_2 filled glovebox, a suspension of **1** (20 mg, 0.045 mmol) in C_6D_6 (0.8 mL) was added to a J-Young NMR tube. Methylidene cyclopentane (**3c**) (4.8 µL, 3.8 mg, 0.045 mmol) was added via micropipette and the NMR tube sealed. The resulting colourless solution was heated to 100 °C for 16 hours, or until the complete consumption of **1** was observed *via* ^1H NMR spectroscopy. The J-Young NMR tube was returned to the glovebox, the solution diluted with toluene (0.5 mL) and decanted into a scintillation vial. The volatiles were removed in *vacuo*, leaving an off-white crystalline solid. The solid was dissolved in n-pentane (2 x 0.5 mL) and filtered. The volatiles were removed in *vacuo*, leaving **4c** as an analytically pure white solid. Yield: 21.4 mg, 0.041 mmol, 90 %. NMR spectra match those reported in the literature.[4]

^1H NMR (400 MHz, C_6D_6) δ: -0.07 (d, $^3J_{H-H}$ = 6.8 Hz, 2H, Mg–C**H**$_2$), 0.66 (m, 2H, ((C**H**$_2$)$_2$(CH$_2$)$_2$CH), 1.15 (d, $^3J_{H-H}$ = 6.9 Hz, 12H, CH(C**H**$_3$)$_2$), 1.29 (d, $^3J_{H-H}$ = 6.9 Hz, 12H, CH(C**H**$_3$)$_2$), 1.44 (m, 4H, ((CH$_2$)$_2$(C**H**$_2$)$_2$CH), 1.68 (s, 6H, NC(C**H**$_3$)), 1.92 (m, 1H, MgCH$_2$C**H**), 3.16 (sept, $^3J_{H-H}$ = 6.8 Hz, 4H, C**H**(CH$_3$)$_2$), 4.94 (s, 1H, (CH$_3$)C(C**H**)C(CH$_3$)), 7.08-7.13 (m, 6H, Ar-**H**).

^{13}C NMR (101 MHz, C_6D_6) δ: 13.7 (Mg–**C**H$_2$), 23.6 (NC**C**H$_3$), 23.7 (NC**C**H$_3$), 24.6 (2x **C**(CH$_3$)$_2$), 25.8 (MgCH$_2$CH(CH$_2$)$_2$(**C**H$_2$)$_2$), 28.7 (2x **C**(CH$_3$)$_2$), 40.4 (MgCH$_2$CH(**C**H$_2$)$_2$), 41.3 (MgCH$_2$**C**H), 95.4 ((CH$_3$)C(**C**H)C(CH$_3$)), 124.1 (*meta*-Ar-**C**H), 126.0 (*para*-Ar-**C**H), 141.9 (*ortho*-Ar-**C**), 144.1 (*ipso*-Ar-**C**), 169.3 (N**C**(CH$_3$)).

Synthesis of 4d

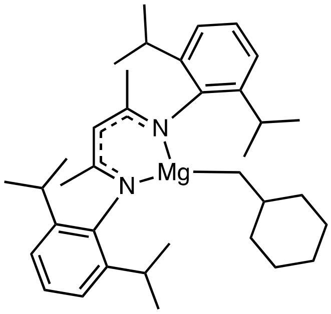

In a N_2 filled glovebox, a suspension of **1** (25 mg, 0.0565 mmol) in C_6D_6 (0.8 mL) was added to a J-Young NMR tube. Methylidene cyclohexane (**3d**) (6.5 μL, 5.4 mg, 0.0565 mmol) was added via micropipette and the NMR tube sealed. The resulting colourless solution was heated to 100 °C for 24 hours, or until the complete consumption of **1** was observed *via* ^{1}H NMR spectroscopy. The J-Young NMR tube was returned to the glovebox, the solution diluted with toluene (0.5 mL) and decanted into a scintillation vial. The volatiles were removed in *vacuo*, leaving an off-white crystalline solid. The solid was dissolved in n-pentane (2 x 0.5 mL) and filtered. The volatiles were removed in *vacuo*, leaving **4d** as an analytically pure white solid. Yield: 30 mg, 0.046 mmol, 83 %.

^{1}H NMR (400 MHz, C_6D_6) δ: -0.18 (d, $^3J_{H\text{-}H}$ = 6.3 Hz, 2H, Mg–C**H**$_2$), 0.54 (m, 2H, $CH(CH_2)_2(CH_2)_2C\mathbf{H}_2$), 0.93-1.13 (m, 4H, mixture of cyclohexyl CH_2 groups), 1.16 (d, $^3J_{H\text{-}H}$ = 6.8 Hz, 12H, $CH(C\mathbf{H}_3)_2$), 1.24-1.28 (m, 2H, mixture of cyclohexyl CH_2 groups and Mg–$CH_2C\mathbf{H}$), 1.29 (d, $^3J_{H\text{-}H}$ = 6.8 Hz, 12H, $CH(C\mathbf{H}_3)_2$), 1.53-1.61 (m, 3H, mixture of cyclohexyl CH_2 groups), 1.68 (s, 6H, $NC(C\mathbf{H}_3)$), 3.16 (sept, $^3J_{H\text{-}H}$ = 6.8 Hz, 4H, $C\mathbf{H}(CH_3)_2$), 4.94 (s, 1H, $(CH_3)C(C\mathbf{H})C(CH_3)$), 7.08-7.14 (m, 6H, Ar-**H**).

^{13}C NMR (101 MHz, C_6D_6) δ: 17.7 (Mg–$\mathbf{C}H_2$), 23.7 ($NC\mathbf{C}H_3$), 24.6 ($CH(\mathbf{C}H_3)_2$), 26.8 ($CH(\mathbf{C}H_2)_2$), 27.8 ($CH(CH_2)_2(\mathbf{C}H_2)_2$), 28.7 ($\mathbf{C}H(CH_3)_2$), 38.1 (Mg–$CH_2\mathbf{C}H$), 41.2 ($CH(CH_2)_2(CH_2)_2(\mathbf{C}H_2)$), 95.3 ($(\mathbf{C}H)C(CH_3)_2$), 124.1 (*para*-Ar-**C**H), 126.0 (*meta*-Ar-**C**H), 141.9 (*ortho*-Ar-**C**), 144.0 (*ipso*-Ar-**C**), 169.3 ($N\mathbf{C}(CH_3)$).

FT-IR (cm^{-1}): 3053, 3021, 2955, 2908, 2864, 2841, 1520, 1457, 1431, 1389, 1357, 1310, 1257, 1251, 1230, 1174, 1100, 1082, 1052, 1025, 962, 933, 900, 880, 866, 850, 840, 795, 772, 759, 749, 727, 702, 693, 642, 629, 595, 552, 542, 524, 508.

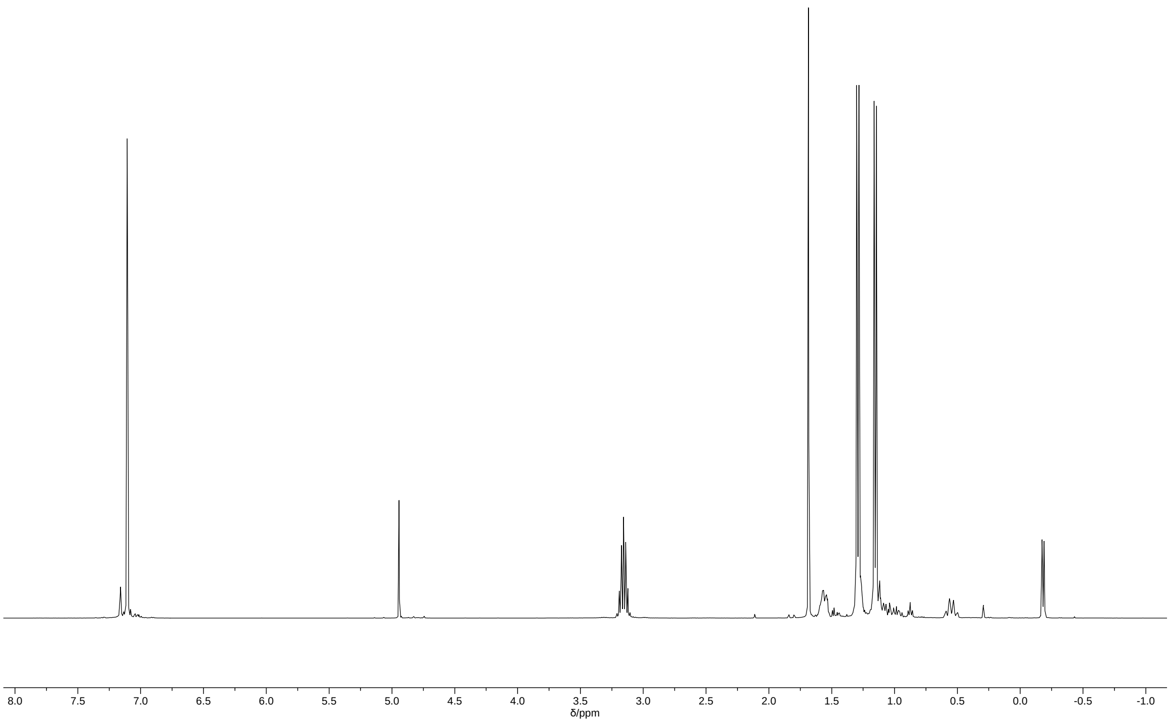

Figure S1. ^{1}H NMR spectrum of **4d**

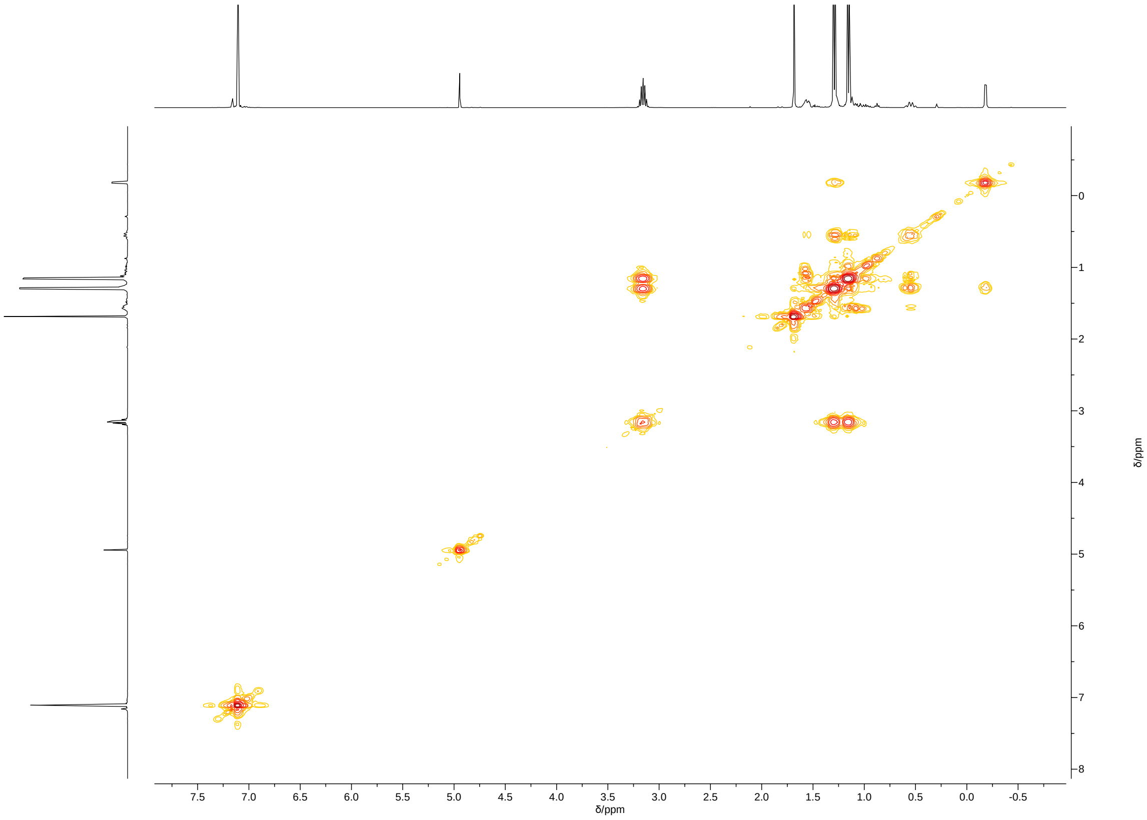

Figure S2. ^{1}H COSY NMR spectrum of **4d**

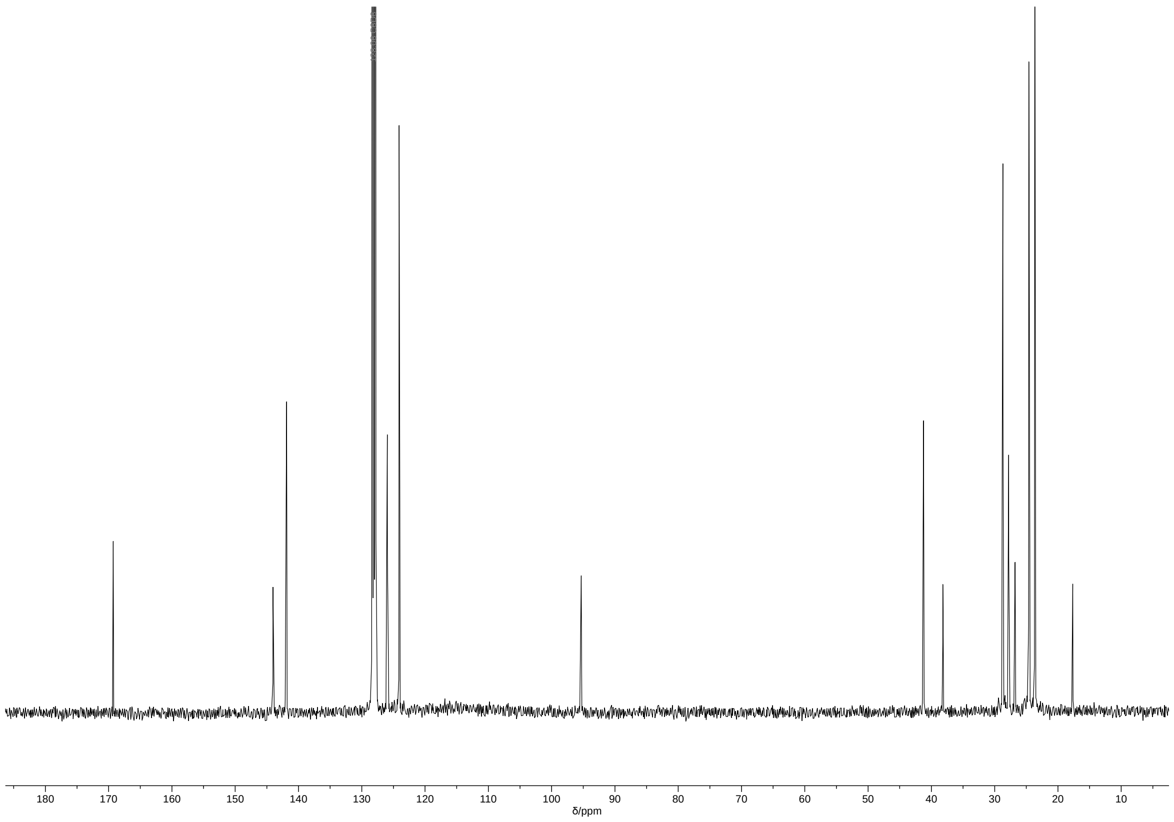

Figure S3. $^{13}C\{^{1}H\}$ NMR spectrum of **4d**

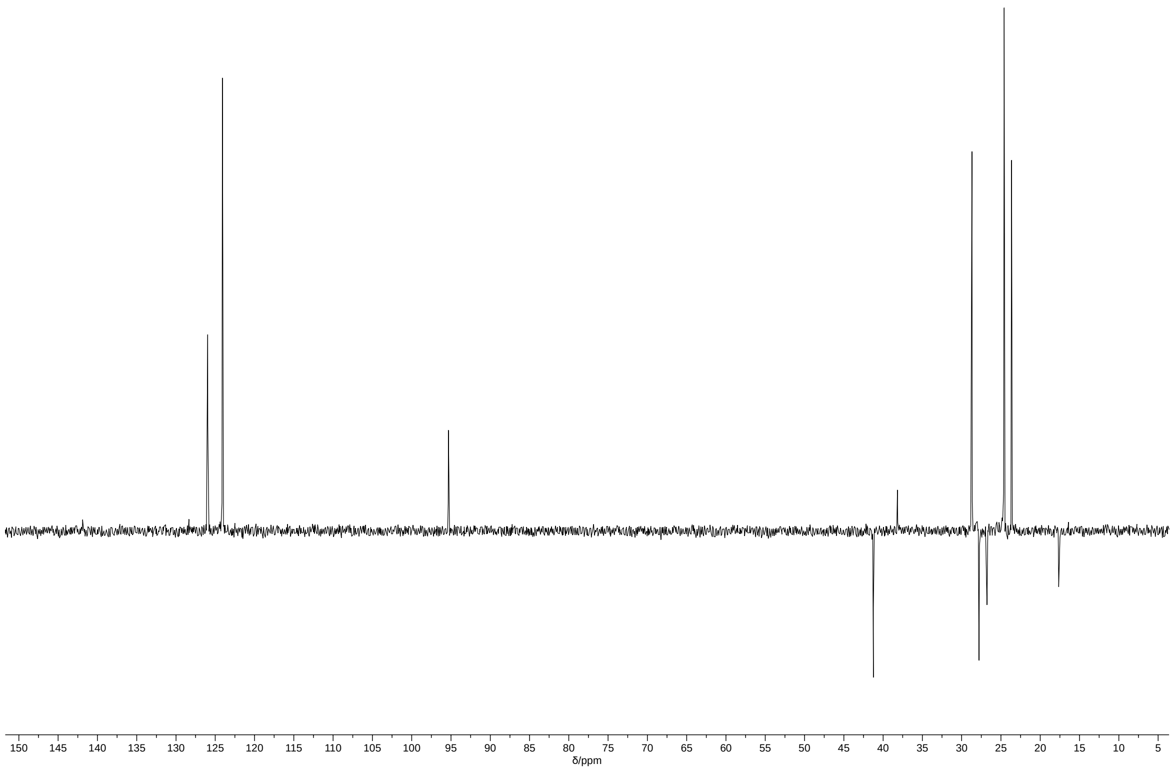

Figure S4. $^{13}C\{^{1}H\}$ DEPT-135 NMR spectrum of **4d**

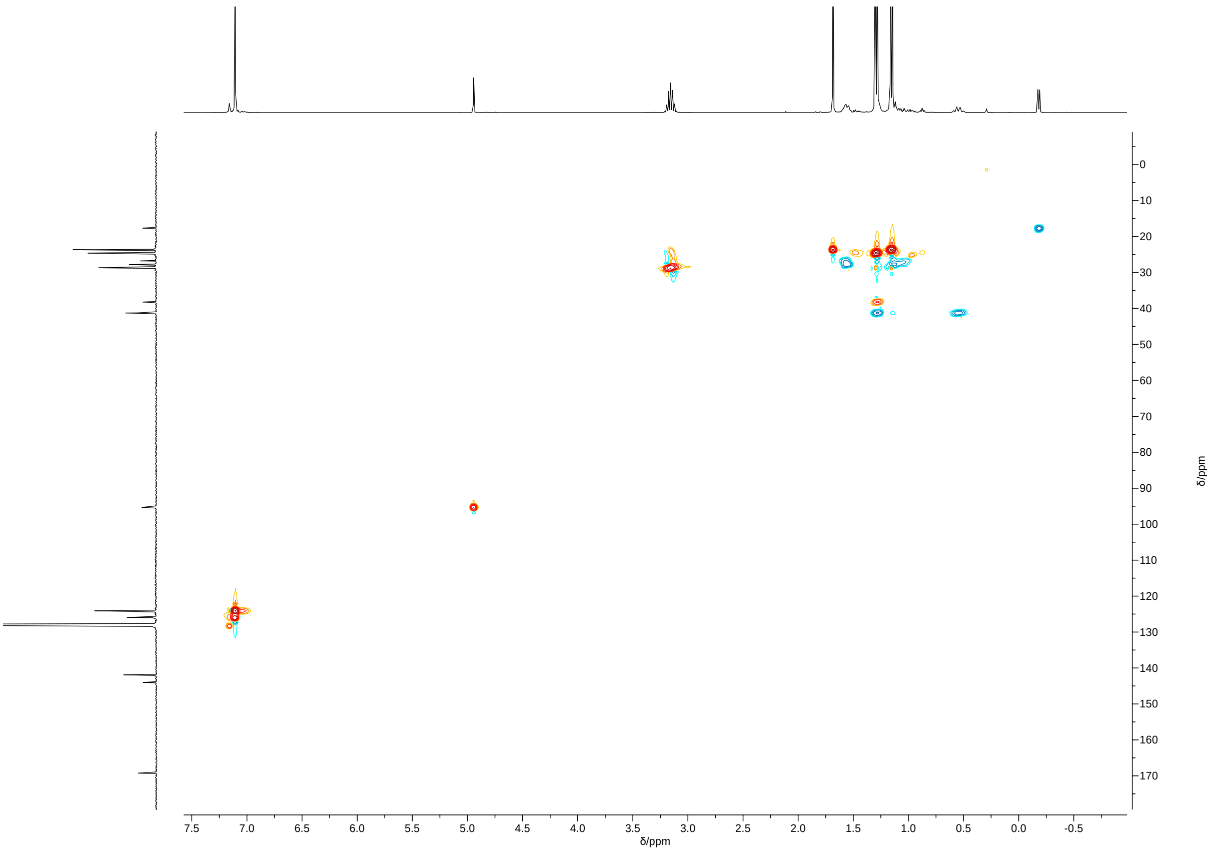

Figure S5. ^{1}H–^{13}C HSQC NMR spectrum of **4d**

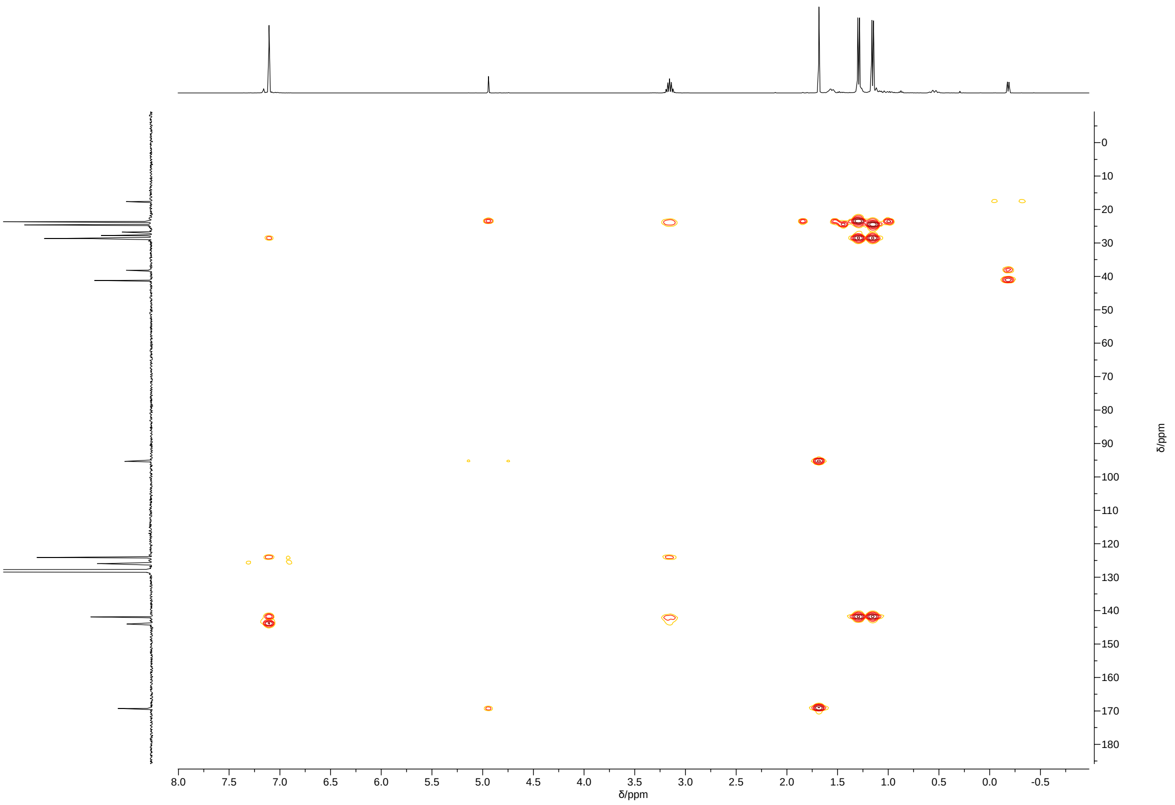

Figure S6. ^{1}H–^{13}C HMBC NMR spectrum of **4d**

Synthesis of 5b

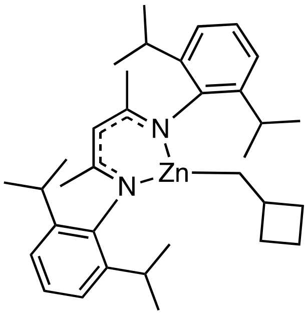

In a N_2 filled glovebox, a suspension of **2** (20 mg, 0.041 mmol) in C_6D_6 (0.6 mL) was added to a J-Young NMR tube. Methylidene cyclobutane (**3b**) (4.6 μL, 3.4 mg, 0.050 mmol) was added via micropipette and the NMR tube sealed. The resulting colourless solution was heated to 100 °C for 39 hours, or until the complete consumption of **2** was observed *via* ^{1}H NMR spectroscopy. The J-Young NMR tube was returned to the glovebox, the solution diluted with toluene (0.5 mL) and decanted into a scintillation vial. The volatiles were removed in *vacuo*, leaving an off-white solid. The solid was dissolved in n-pentane (1 mL) and filtered. The volatiles were removed in *vacuo*, leaving **5b** as an analytically pure white solid. Yield: 17.9 mg, 0.032 mmol, 79 %.

^{1}H NMR (400 MHz, C_6D_6) δ: 0.48 (d, $^3J_{H-H}$ = 7.9 Hz, 2H, Zn–C**H**$_2$), 1.16 (d, $^3J_{H-H}$ = 7.1 Hz, 12H, CH(C**H**$_3$)$_2$), 1.17-1.24 (m, 2H, CHC**H**$_2$CH$_2$), 1.29 (d, $^3J_{H-H}$ = 7.1 Hz, 12H, CH(C**H**$_3$)$_2$), 1.47-1.60 (m, 2H, CH(CH$_2$)C**H**$_2$), 1.61-1.68 (m, 2H, CHC**H**$_2$CH$_2$), 1.70 (s, 6H, NC(C**H**$_3$)), 2.26 (pseudo-sept, 1H, $^3J_{H-H}$ = 8.0 Hz, ZnCH$_2$C**H**), 3.17 (sept, 4H, $^3J_{H-H}$ = 7.1 Hz, C**H**(CH$_3$)$_2$), 4.99 (s, 1H, (CH$_3$)C(C**H**)C(CH$_3$)), 7.12 (m, 6H, Ar-**H**).

^{13}C NMR (101 MHz, C_6D_6) δ: 17.1 (CHCH$_2$**C**H$_2$), 18.3 (Zn-**C**H$_2$), 23.5 (NC(**C**H$_3$)), 23.6 (CH(**C**H$_3$)$_2$), 24.37 (CH(**C**H$_3$)$_2$), 28.6 (**C**H(CH$_3$)$_2$), 34.1 (CH**C**H$_2$CH$_2$), 37.4 (ZnCH$_2$**C**H), 95.5 ((CH$_3$)C(**C**H)C(CH$_3$)), 123.9 (*meta*-Ar-**C**H), 126.0 (*para*-Ar-**C**H), 141.6 (*ortho*-Ar-**C**), 145.1 (*ipso*-Ar-**C**), 167.5 (N**C**(CH$_3$)).

FT-IR (cm^{-1}): 3057, 3020, 2956, 2921, 2863, 1550, 1524, 1436, 1380, 1360, 1317, 1264, 1251, 1232, 1175, 1100, 1056, 1018, 936, 856, 793, 755, 718, 703, 657, 625, 569, 528, 431.

Anal. Calcd. ($C_{34}H_{50}N_2Zn$): C, 73.96; H, 9.13; N, 5.07. Found: C, 73.60; H, 8.90; N, 4.95.

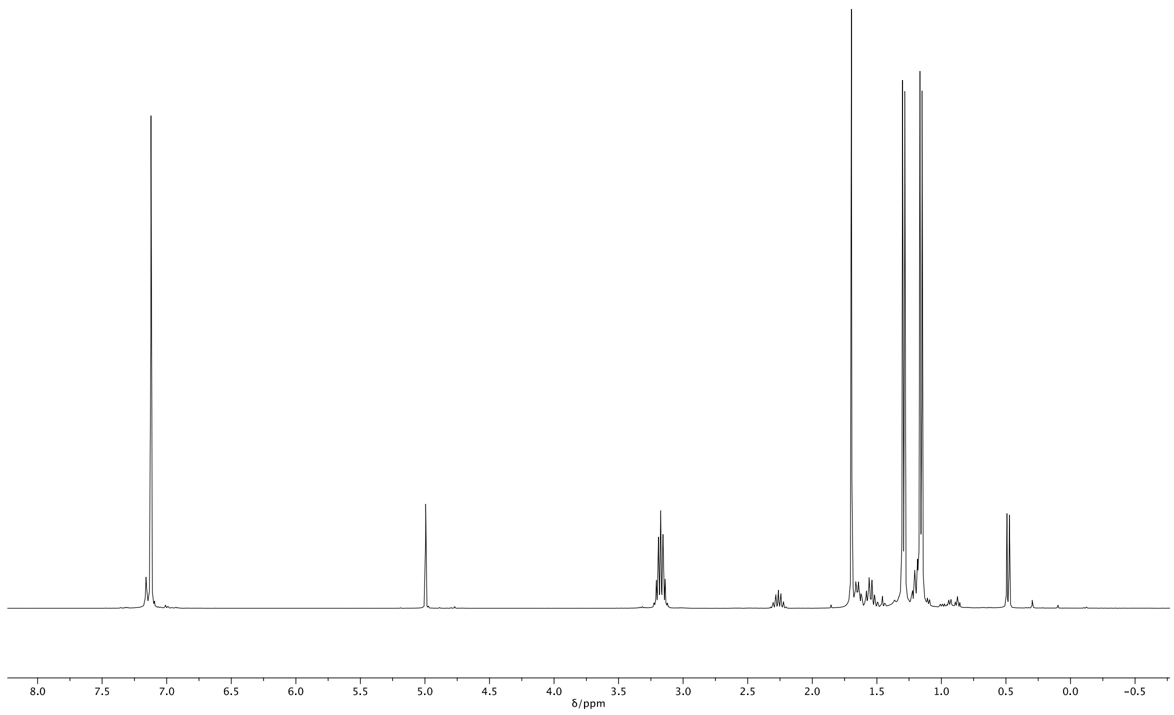

Figure S7. ^{1}H NMR spectrum of **5b**

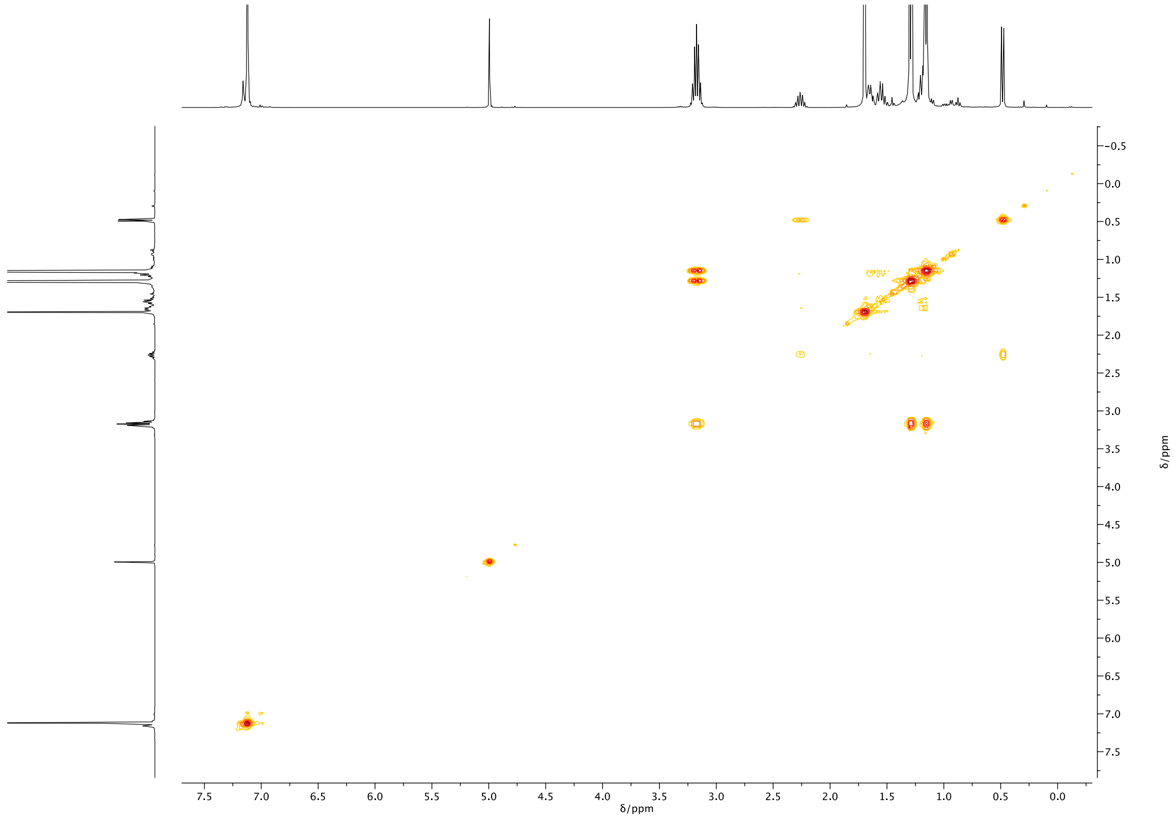

Figure S8. ^{1}H COSY NMR spectrum of **5b**

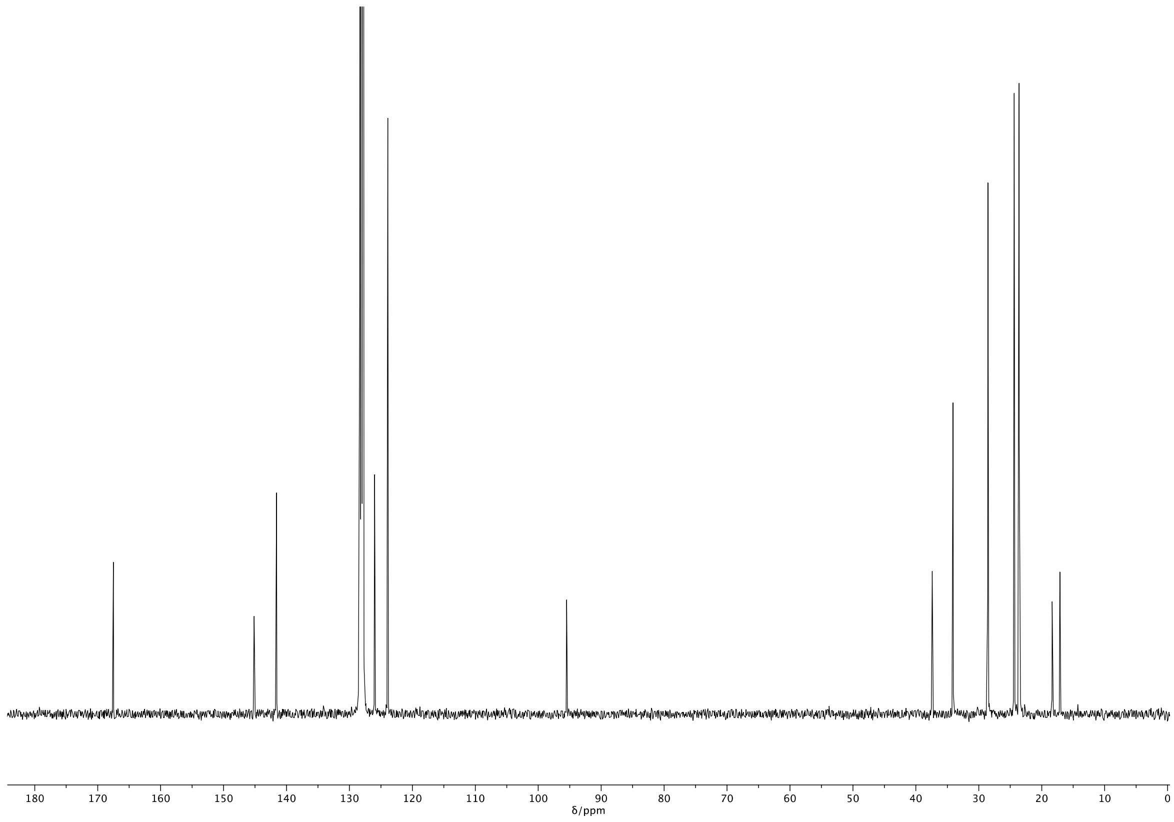

Figure S9. $^{13}C\{^1H\}$ NMR spectrum of **5b**

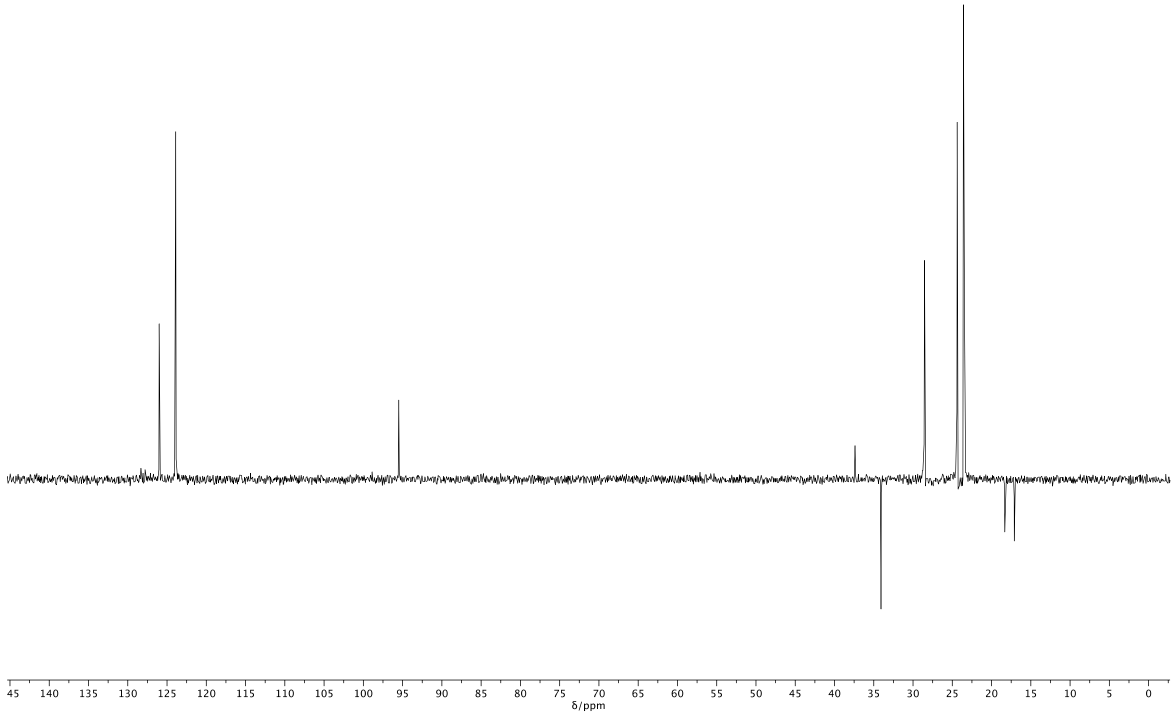

Figure S10. $^{13}C\{^1H\}$ DEPT-135 NMR spectrum of **5b**

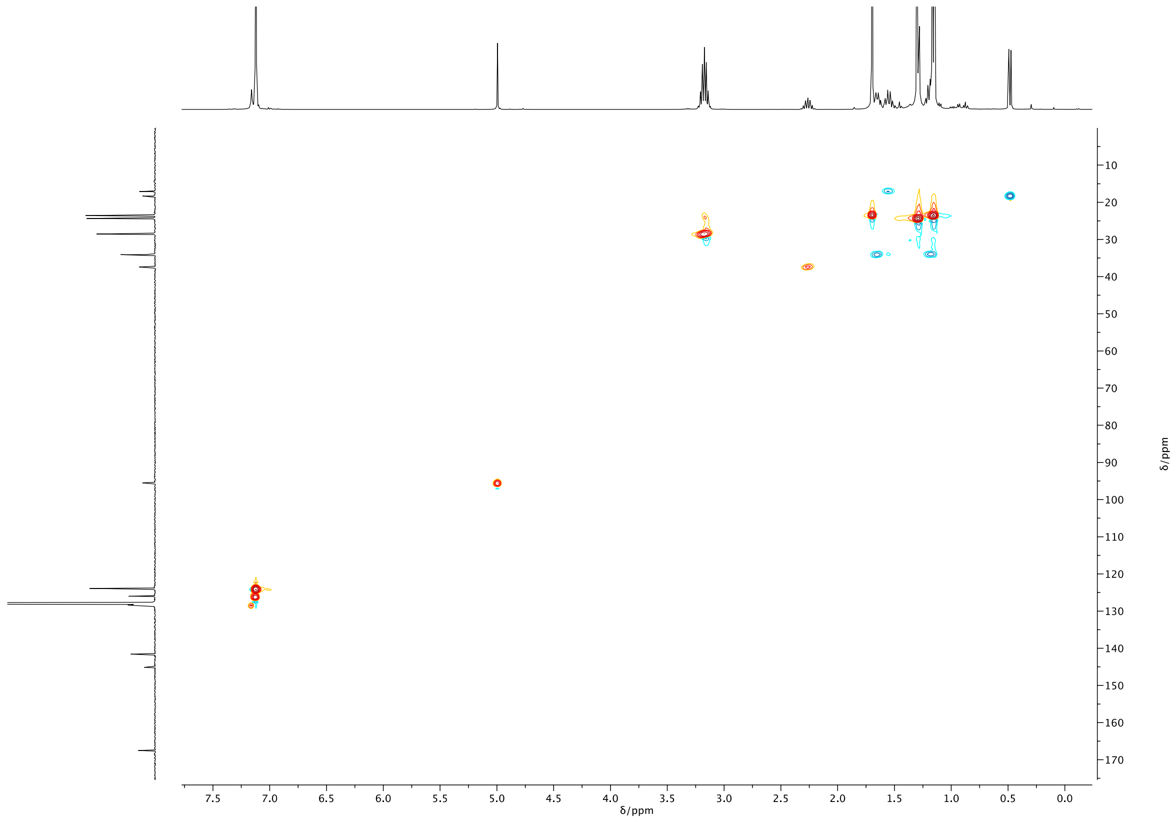

Figure S11. ^{1}H–^{13}C HSQC NMR spectrum of **5b**

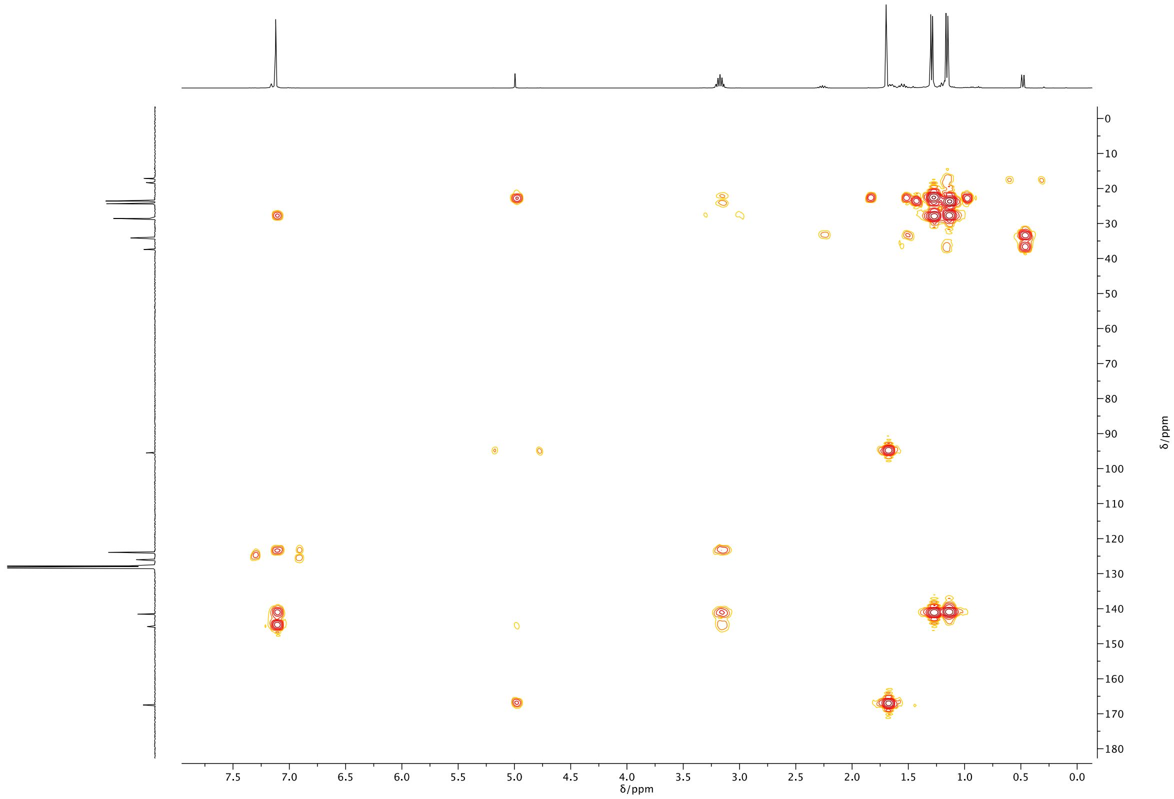

Figure S12. ^{1}H–^{13}C HMBC NMR spectrum of **5b**

Synthesis of 5c

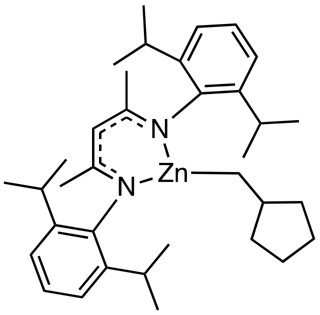

In a N_2 filled glovebox, a suspension of **2** (20 mg, 0.041 mmol) in C_6D_6 (0.6 mL) was added to a J-Young NMR tube. Methylidene cyclopentane (**3c**) (5.6 μL, 4.4 mg, 0.053 mmol) was added via micropipette and the NMR tube sealed. The resulting colourless solution was heated to 100 °C for 39 hours, or until the complete consumption of **2** was observed *via* ^{1}H NMR spectroscopy. The J-Young NMR tube was returned to the glovebox, the solution diluted with toluene (0.5 mL) and decanted into a scintillation vial. The volatiles were removed in *vacuo*, leaving an off-white solid. The solid was dissolved in n-pentane (1 mL) and filtered. The volatiles were removed in *vacuo*, leaving **5c** as an analytically pure white solid. Yield: 17.7 mg, 0.031 mmol, 76%.

^{1}H NMR (400 MHz, C_6D_6) δ: 0.40 (d, $^3J_{H\text{-}H}$ = 7.1 Hz, 2H, Zn–C**H**$_2$), 0.64-0.73 (m, 2H, $(CH_2)_2$(C**H**$_2$)$_2$CH), 1.16 (d, $^3J_{H\text{-}H}$ = 7.3 Hz, 12H, CH(C**H**$_3$)$_2$), 1.20-1.27 (m, 2H, $(CH_2)_2$(C**H**$_2$)$_2$CH), 1.30 (d, $^3J_{H\text{-}H}$ = 7.3 Hz, 12H, CH(C**H**$_3$)$_2$), 1.35-1.43 (m, 2H, (C**H**$_2$)$_2$$(CH_2)_2$CH), 1.46-1.57 (m, 2H, (C**H**$_2$)$_2$$(CH_2)_2$CH), 1.71 (s, 6H, NC(C**H**$_3$)), 1.75-1.85 (m, 1H, $(CH_2)_2(CH_2)_2$C**H**), 3.20 (sept, 4H, $^3J_{H\text{-}H}$ = 7.3 Hz, C**H**$(CH_3)_2$), 5.01 (s, 1H, (CH_3)C(C**H**)C(CH_3)), 7.12 (m, 6H, Ar-**H**).

^{13}C NMR (101 MHz, C_6D_6) δ: 15.6 (Zn-**C**H$_2$), 23.5 (NC(**C**H$_3$)), 23.7 (CH(**C**H$_3$)$_2$), 24.3 (CH(**C**H$_3$)$_2$), 25.7 ((**C**H$_2$)$_2$$(CH_2)_2$CH), 28.6 (**C**H$(CH_3)_2$), 38.7 ($(CH_2)_2$(**C**H$_2$)$_2$CH), 40.5 ($(CH_2)_2(CH_2)_2$**C**H), 95.5 ($(CH_3)$C(**C**H)C$(CH_3)$), 123.9 (*meta*-Ar-**C**H), 126.0 (*para*-Ar-**C**H), 141.7 (*ortho*-Ar-**C**), 145.2 (*ipso*-Ar-**C**), 167.5 (N**C**(CH$_3$)).

FT-IR (cm^{-1}): 3057, 3020, 2956, 2921, 2863, 1550, 1524, 1436, 1380, 1360, 1317, 1264, 1251, 1232, 1175, 1100, 1056, 1018, 936, 856, 793, 755, 718, 703, 657, 625, 569, 528, 431.

Anal. Calcd. ($C_{35}H_{52}N_2Zn$): C, 74.25; H, 9.26; N, 4.95. Found: C, 73.38; H, 9.18; N, 4.94.

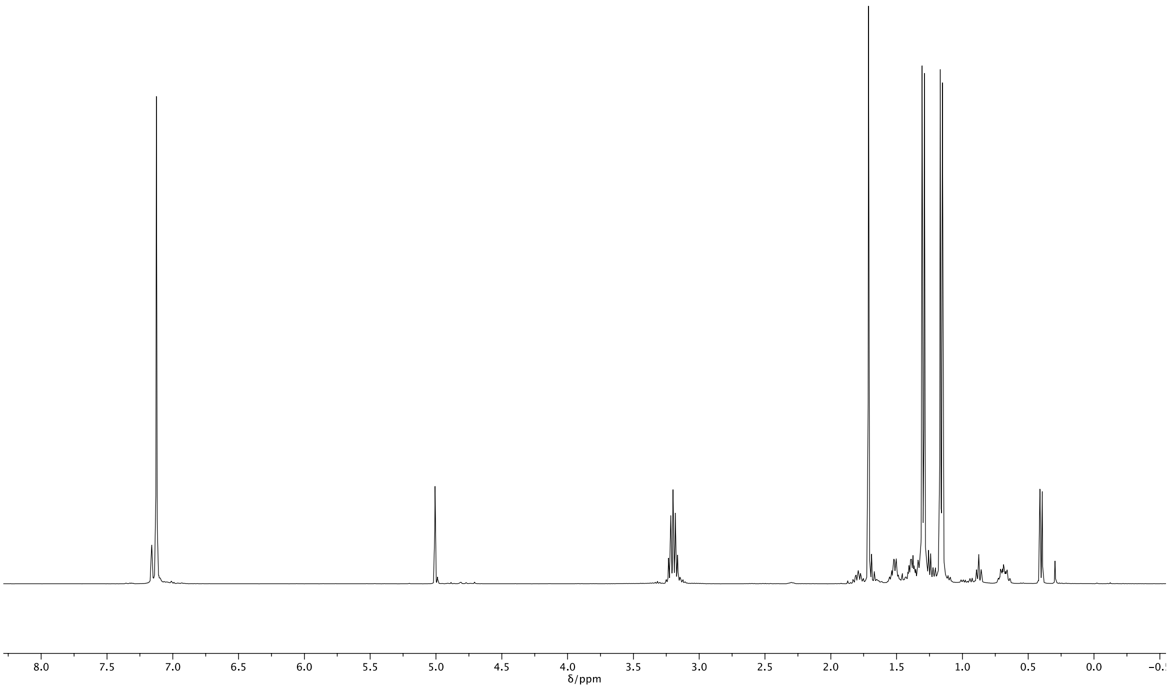

Figure S13. ^{1}H NMR spectrum of **5c**

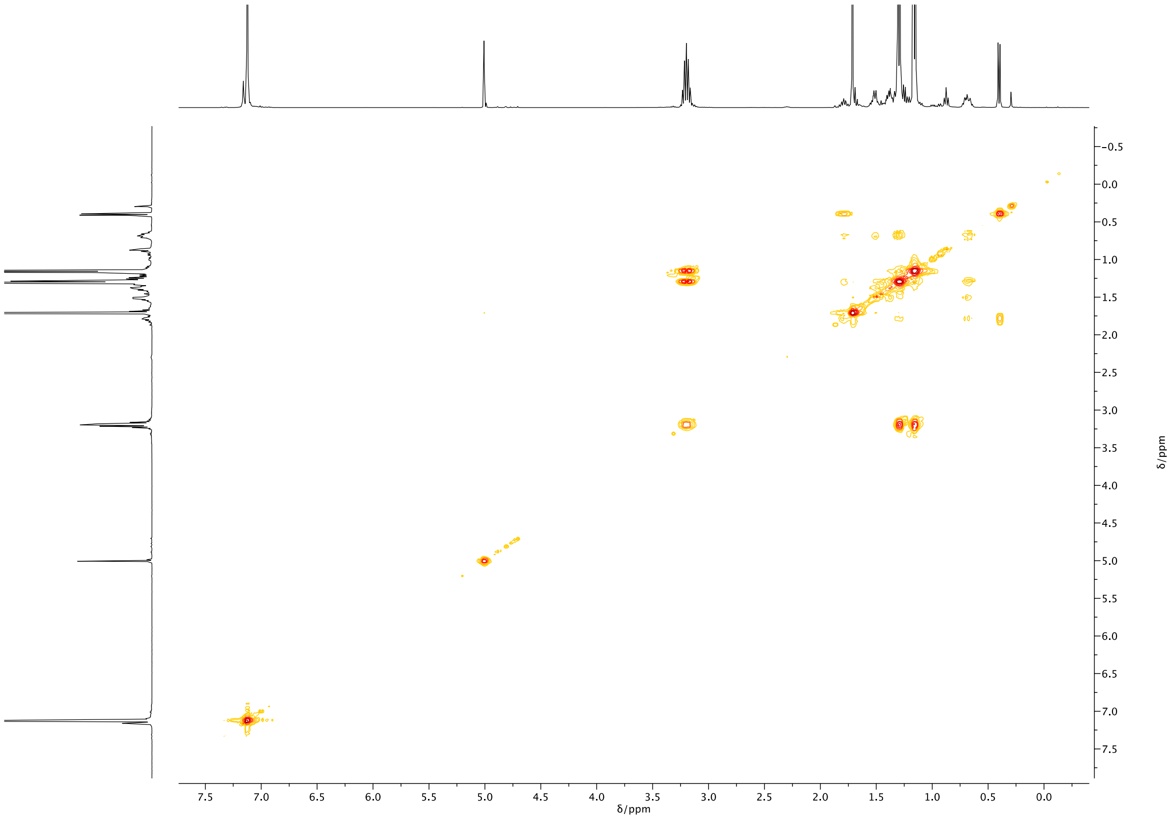

Figure S14. ^{1}H COSY NMR spectrum of **5c**

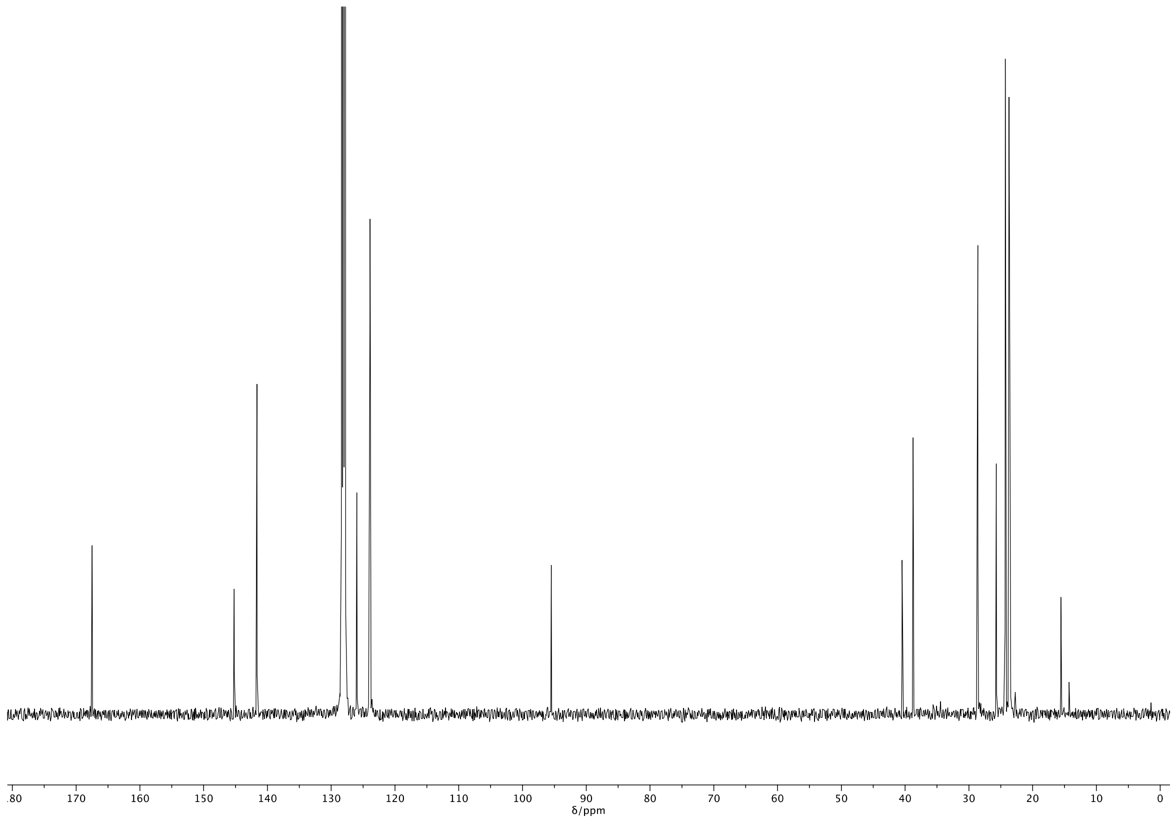

Figure S15. $^{13}C\{^{1}H\}$ NMR spectrum of **5c**

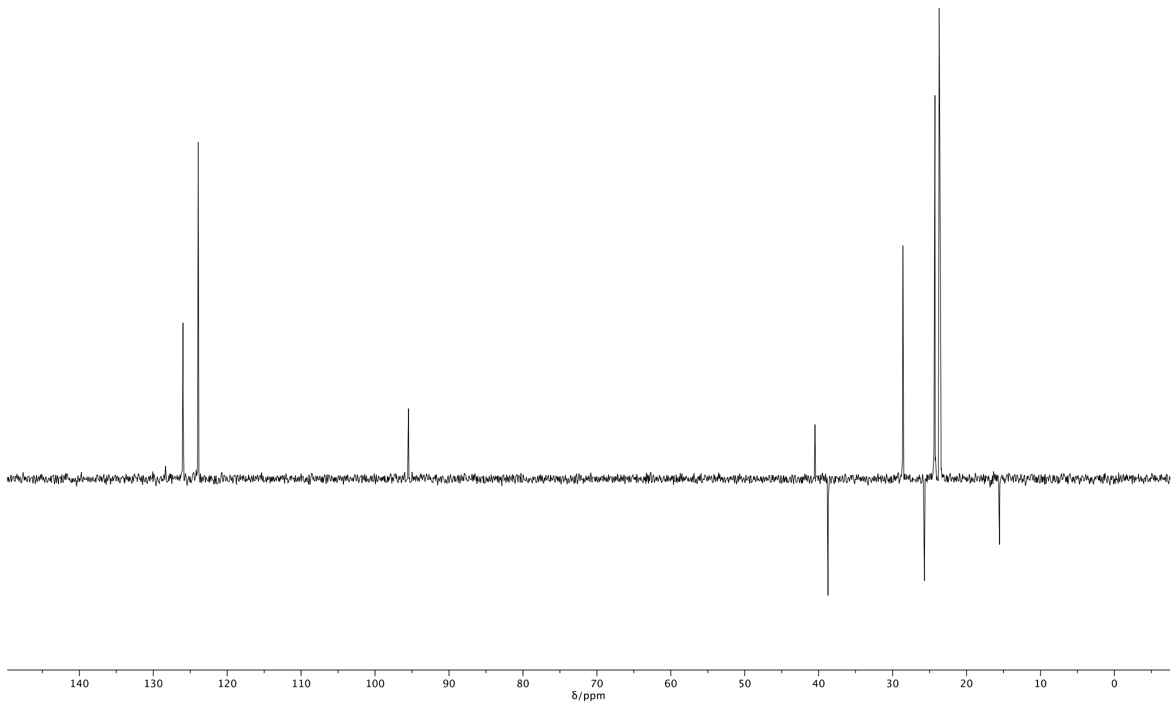

Figure S16. $^{13}C\{^{1}H\}$ DEPT-135 NMR spectrum of **5c**

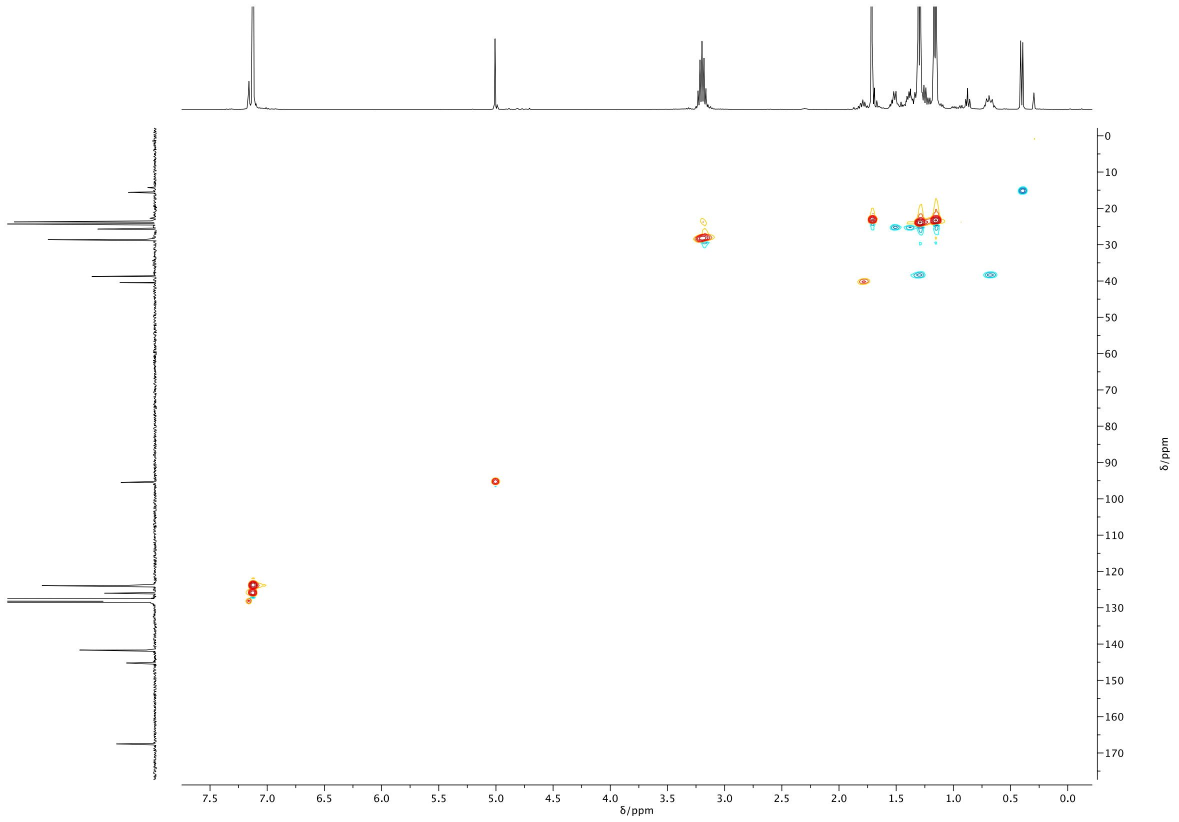

Figure S17. ^{1}H–^{13}C HSQC NMR spectrum of **5c**

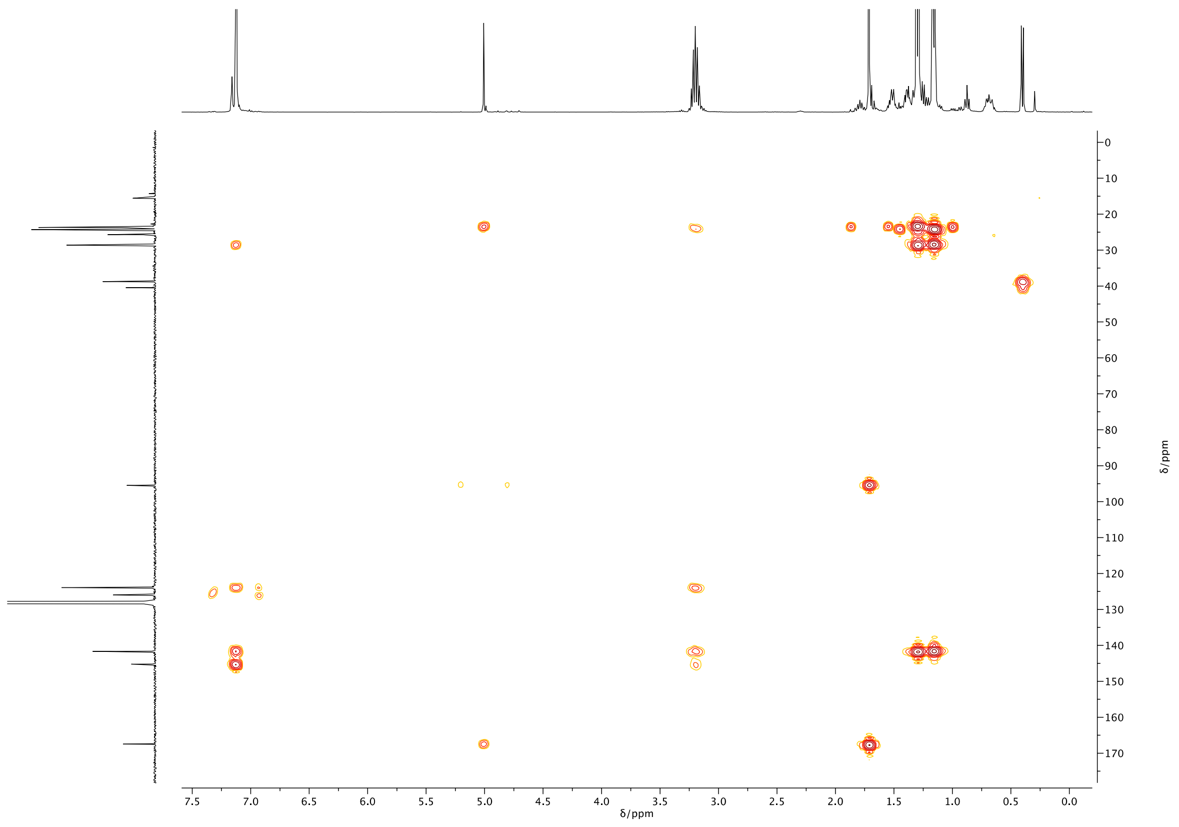

Figure S18. ^{1}H–^{13}C HMBC NMR spectrum of **5c**

Synthesis of 5d

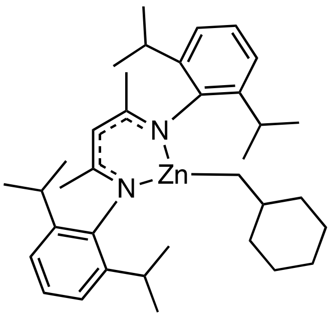

In a N_2 filled glovebox, a suspension of **2** (20 mg, 0.041 mmol) in C_6D_6 (0.6 mL) was added to a J-Young NMR tube. Methylidene cyclohexane (**3d**) (6.0 µL, 4.8 mg, 0.050 mmol) was added via micropipette and the NMR tube sealed. The resulting colourless solution was heated to 100 °C for 39 hours, or until the complete consumption of **2** was observed *via* ^{1}H NMR spectroscopy. The J-Young NMR tube was returned to the glovebox, the solution diluted with toluene (0.5 mL) and decanted into a scintillation vial. The volatiles were removed in *vacuo*, leaving an off-white solid. The solid was dissolved in n-pentane (1 mL) and filtered. The volatiles were removed in *vacuo*, leaving **5d** as an analytically pure white solid. Yield: 17.6 mg, 0.030 mmol, 73%. $CH(CH_2)_2(CH_2)_2CH_2$

^{1}H NMR (400 MHz, C_6D_6) δ: 0.27 (d, $^3J_{H-H}$ = 6.7 Hz, 2H, Zn–C**H**$_2$), 0.52-0.62 (m, 2H, CH(C**H**$_2$)$_2$(CH$_2$)$_2$CH$_2$), 0.98-1.08 (m, 3H, CH(CH$_2$)$_2$(C**H**$_2$)$_2$C**H**$_2$), 1.08-1.14 (m, 1H, C**H**(CH$_2$)$_2$(CH$_2$)$_2$CH$_2$), 1.16 (d, $^3J_{H-H}$ = 7.1 Hz, 12H, CH(C**H**$_3$)$_2$), 1.18-1.22 (m, 2H, CH(C**H**$_2$)$_2$(CH$_2$)$_2$CH$_2$), 1.29 (d, $^3J_{H-H}$ = 7.1 Hz, 12H, CH(C**H**$_3$)$_2$), 1.50-1.58 (m, 3H, CH(CH$_2$)$_2$(C**H**$_2$)$_2$C**H**$_2$), 1.72 (s, 6H, NC(C**H**$_3$)), 3.19 (sept, 4H, $^3J_{H-H}$ = 7.1 Hz, C**H**(CH$_3$)$_2$), 5.01 (s, 1H, (CH$_3$)C(C**H**)C(CH$_3$)), 7.12 (m, 6H, Ar-**H**).

^{13}C NMR (101 MHz, C_6D_6) δ: 19.2 (Zn-**C**H$_2$), 23.6 (NC(**C**H$_3$)), 23.8 (CH(**C**H$_3$)$_2$), 24.3 (CH(**C**H$_3$)$_2$), 26.7 (CH(CH$_2$)$_2$(**C**H$_2$)$_2$**C**H$_2$), 27.6 (CH(CH$_2$)$_2$(**C**H$_2$)$_2$**C**H$_2$), 28.6 (**C**H(CH$_3$)$_2$), 37.5 (**C**H(CH$_2$)$_2$(CH$_2$)$_2$CH$_2$), 39.5 (CH(**C**H$_2$)$_2$(CH$_2$)$_2$CH$_2$), 95.4 ((CH$_3$)C(**C**H)C(CH$_3$)), 123.9 (*meta*-Ar-**C**H), 125.9 (*para*-Ar-**C**H), 141.7 (*ortho*-Ar-**C**), 145.2 (*ipso*-Ar-**C**), 167.4 (**C**N(CH$_3$)).

FT-IR (cm^{-1}): 3055, 3020, 2955, 2909, 2865, 2845, 1550, 1525, 1437, 1396, 1381, 1361, 1316, 1263, 1251, 1231, 1178, 1100, 1054, 1026, 1016, 934, 885, 858, 796, 779, 759, 753, 728, 719, 702, 642, 608, 528, 439, 434.

Anal. Calcd. ($C_{36}H_{54}N_2Zn$): C, 74.52; H, 9.38; N, 4.83. Found: C, 74.36; H, 9.32; N, 4.76.

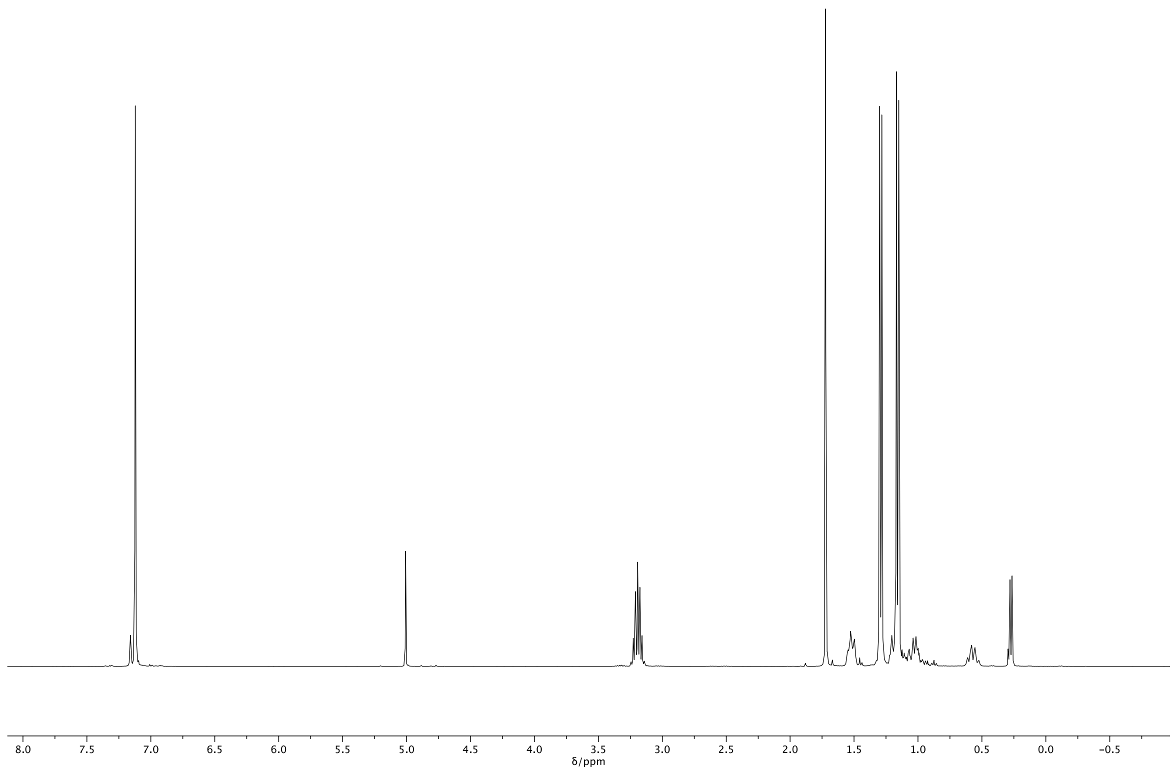

Figure S19. ^{1}H NMR spectrum of **5d**

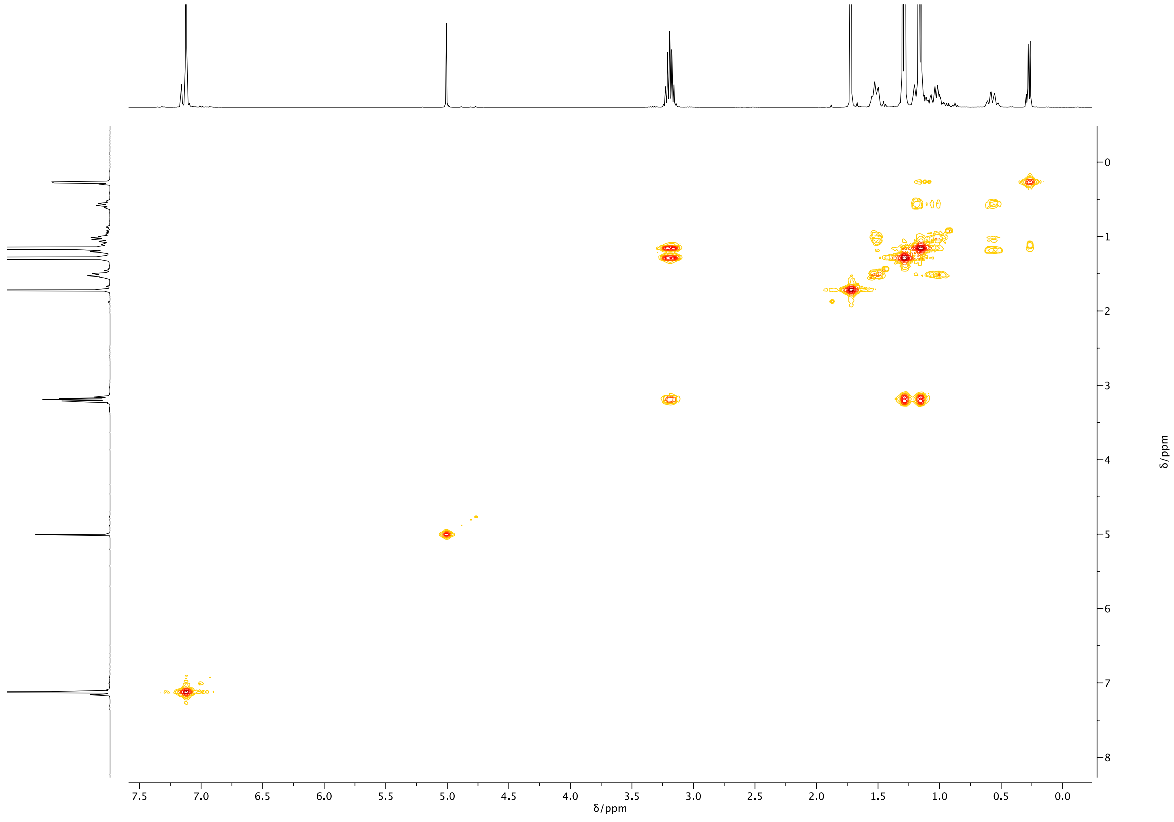

Figure S20. ^{1}H COSY NMR spectrum of **5d**

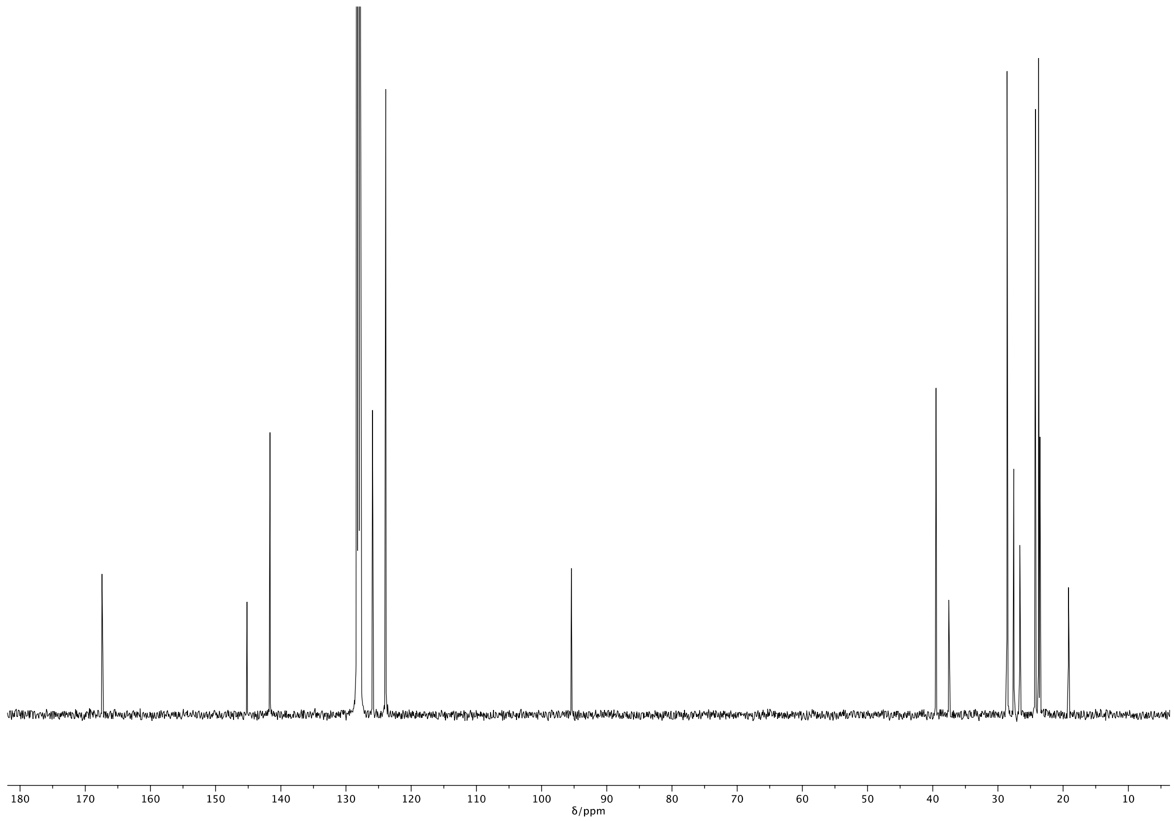

Figure S21. $^{13}C\{^{1}H\}$ NMR spectrum of **5d**

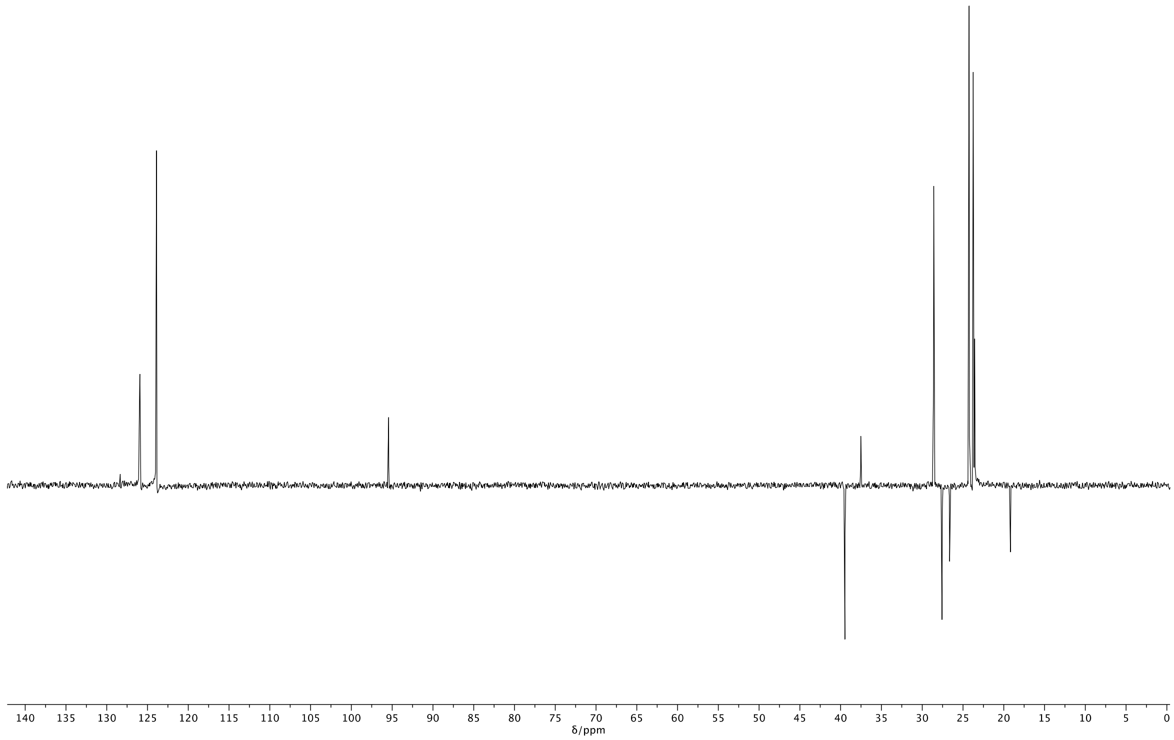

Figure S22. $^{13}C\{^{1}H\}$ DEPT-135 NMR spectrum of **5d**

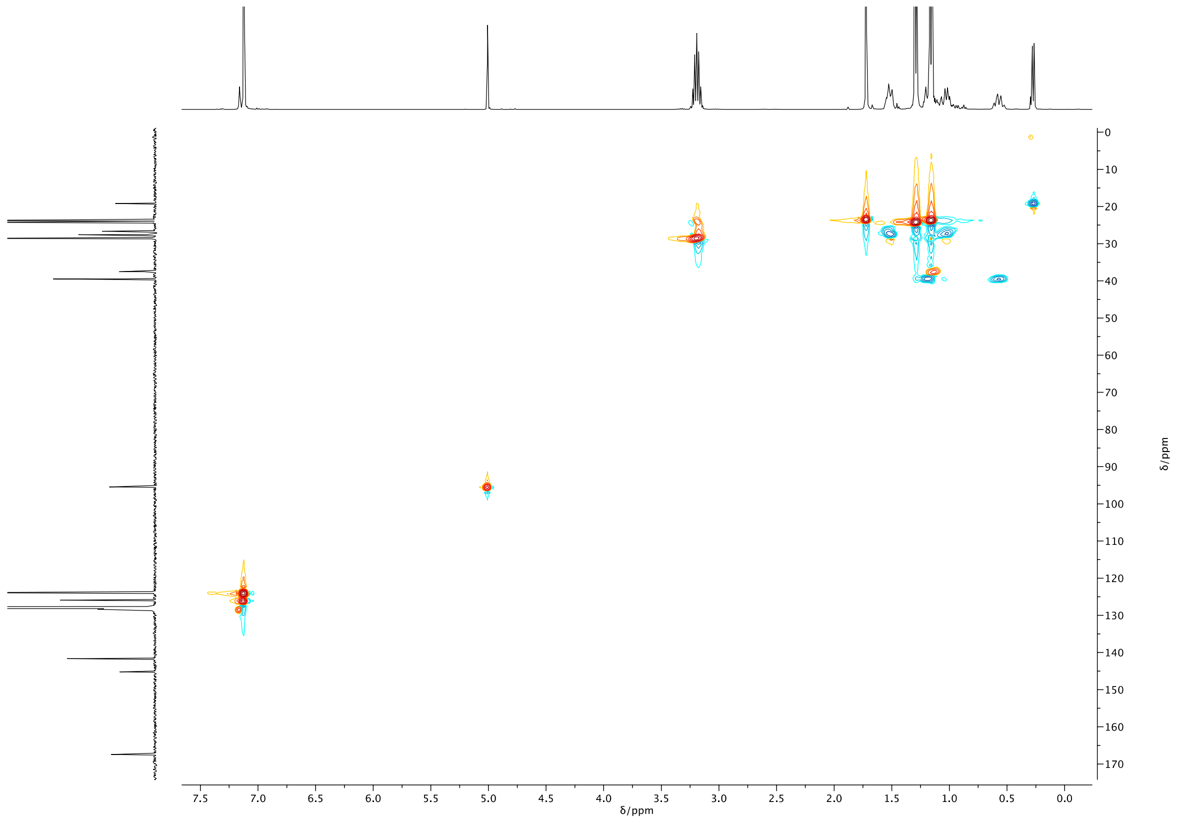

Figure S23. ^{1}H–^{13}C HSQC NMR spectrum of **5d**

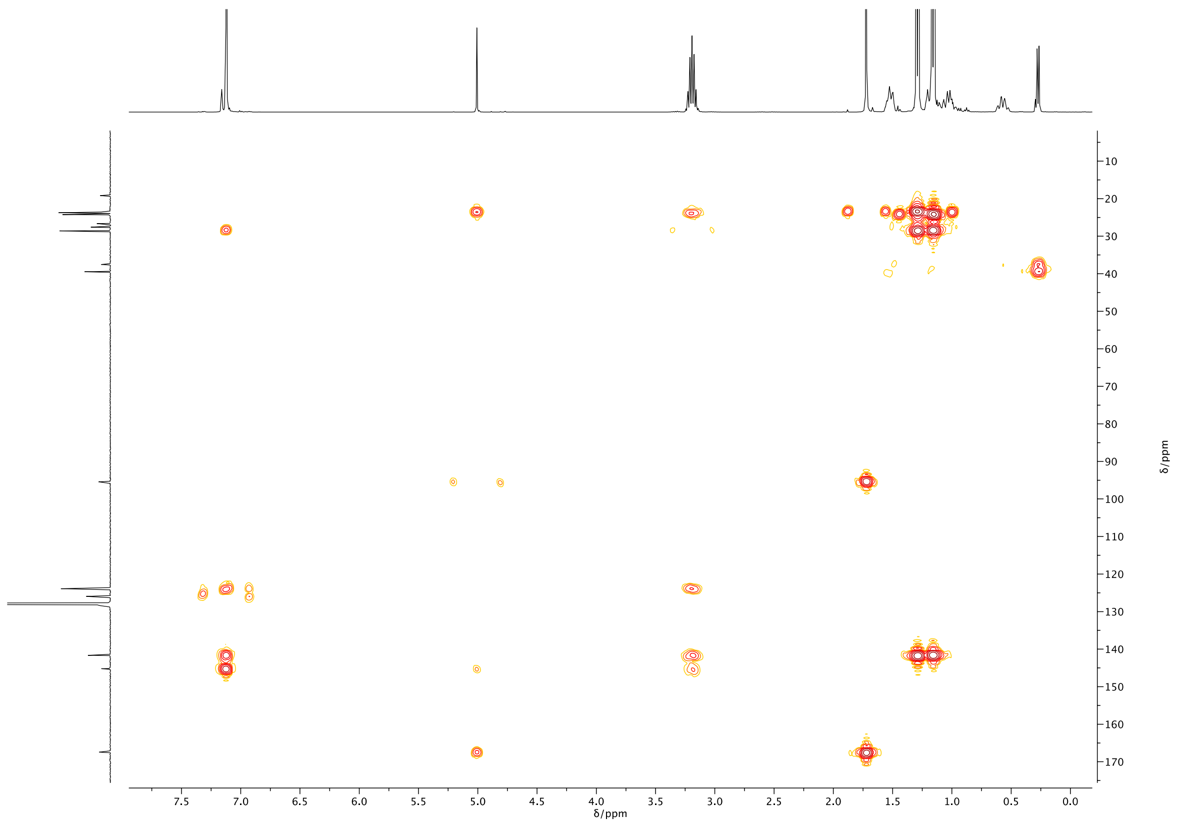

Figure S24. ^{1}H–^{13}C HMBC NMR spectrum of **5d**

Synthesis of 6b

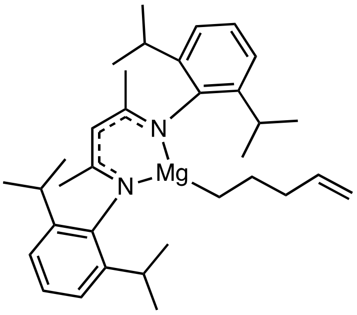

In a N_2 filled glovebox, a suspension of **1** (20 mg, 0.045 mmol) in C_6D_6 (0.6 mL) was added to a J-Young NMR tube. Methylidene cyclobutane (**3b**) (20 μL, 14.7 mg, 0.22 mmol) was added via micropipette and the NMR tube sealed. The resulting pale-yellow solution was monitored over 24 hours at 60, 80 and 100 °C. At each temperature the concomitant formation of both **6b** and **8b** was observed, in a **6b**:**8b** ratio of 6.0:1, 4.9:1 and 3.2:1, respectively, after heating for 5 hours. The highest ratio of **6b** in the reaction mixture is observed at 60 °C, but longer reaction times (72 h) are required for the reaction to reach completion. The J-Young NMR tube was returned to the glovebox, the solution diluted with toluene (0.5 mL) and decanted into a scintillation vial. The volatiles were removed in *vacuo*, leaving an off-white crystalline solid. The solid was dissolved in n-pentane (2 x 0.5 mL) and filtered. The volatiles were removed in *vacuo*, leaving a mixture of **6b** and **8b** as a white powder (22.1 mg). A mixture of **6b** and **8b** is always observed after work-up (typically in a **6b**:**8b** ratio of 5:1), and the similar solubilities of the two species made separation impossible in our hands. Yield: 18.4 mg, 0.036 mmol, 80 % (as component of mixture). A combination of selective excitation TOCSY, DEPT-edited HSQC and HMBC NMR experiments were used to determine alkene ^{1}H and ^{13}C resonances of **6b**, alongside pure ^{1}H and ^{13}C{^{1}H} spectra of **8b**.

^{1}H NMR (400 MHz, C_6D_6) δ: -0.25 (t, $^3J_{H-H}$ = 8.1 Hz, 2H, Mg–C**H**$_2$), 1.15 (d, $^3J_{H-H}$ = 6.8 Hz, 12H, CH(C**H**$_3$)$_2$), 1.27 (d, $^3J_{H-H}$ = 6.8 Hz, 12H, CH(C**H**$_3$)$_2$), 1.44 (m, 2H, MgCH$_2$C**H**$_2$), 1.65 (s, 12H, NC(C**H**$_3$)), 1.77 (m, 2H, MgCH$_2$CH$_2$C**H**$_2$), 3.16 (sept, $^3J_{H-H}$ = 7.1 Hz, 4H, C**H**(CH$_3$)$_2$), 4.81-4.91 (overlapping ddt, 2H, CHC**H**$_2$), 4.93 (s, 2H, (CH$_3$)C(C**H**)C(CH$_3$)), 5.77 (ddt, 1H, CH$_2$C**H**CH$_2$), 7.08-7.13 (multi, 6H, Ar-**H**).

^{13}C NMR (101 MHz, C_6D_6) δ: 5.5 (Mg-**C**H$_2$), 23.5 (CH(**C**H$_3$)$_2$), 23.6 (NC**C**H$_3$), 24.7 (CH(**C**H$_3$)$_2$), 28.3 (MgCH$_2$**C**H$_2$), 28.6 (**C**H(CH$_3$)$_2$), 42.0 (MgCH$_2$CH$_2$**C**H$_2$), 95.3 ((**C**H)C(CH$_3$)$_2$), 113.4 (CH=**C**H$_2$), 124.1 (*para*-Ar-**C**H), 126.0 (*meta*-Ar-**C**H), 141.3 (*ortho*-Ar-**C**), 142.0 (*ipso*-Ar-**C**), 141.9 (**C**H=CH$_2$), 169.3 (4x N**C**(CH$_3$)).

FT-IR (cm^{-1}): 3071, 2975, 2846, **1648 (C=C stretch)**, 1605, 1565, 1486, 1398, 1329, 1299, 1241, 1206, 1133, 1076, 1027, 986, 954, 922, 845, 797.

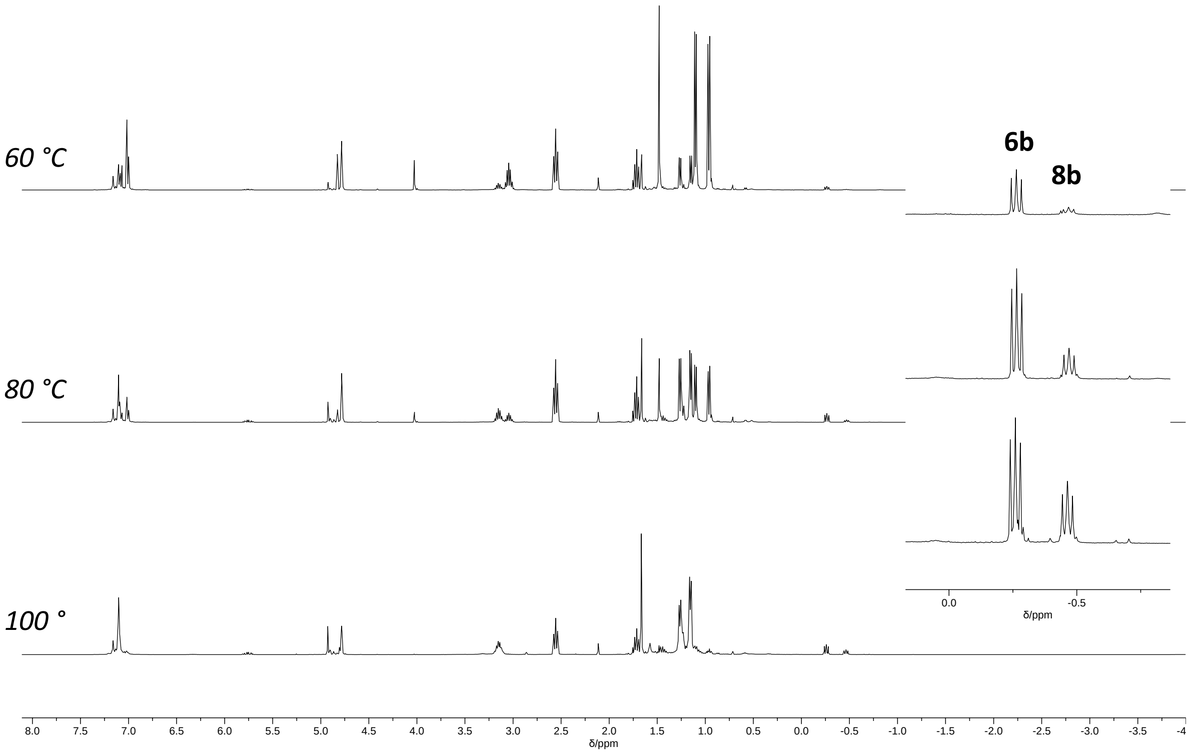

Figure S25. Stacked ^{1}H NMR spectra of reaction between **1** and **3b** at 60–100 °C after 5 h: formation of both **6b** and **8b** are observed

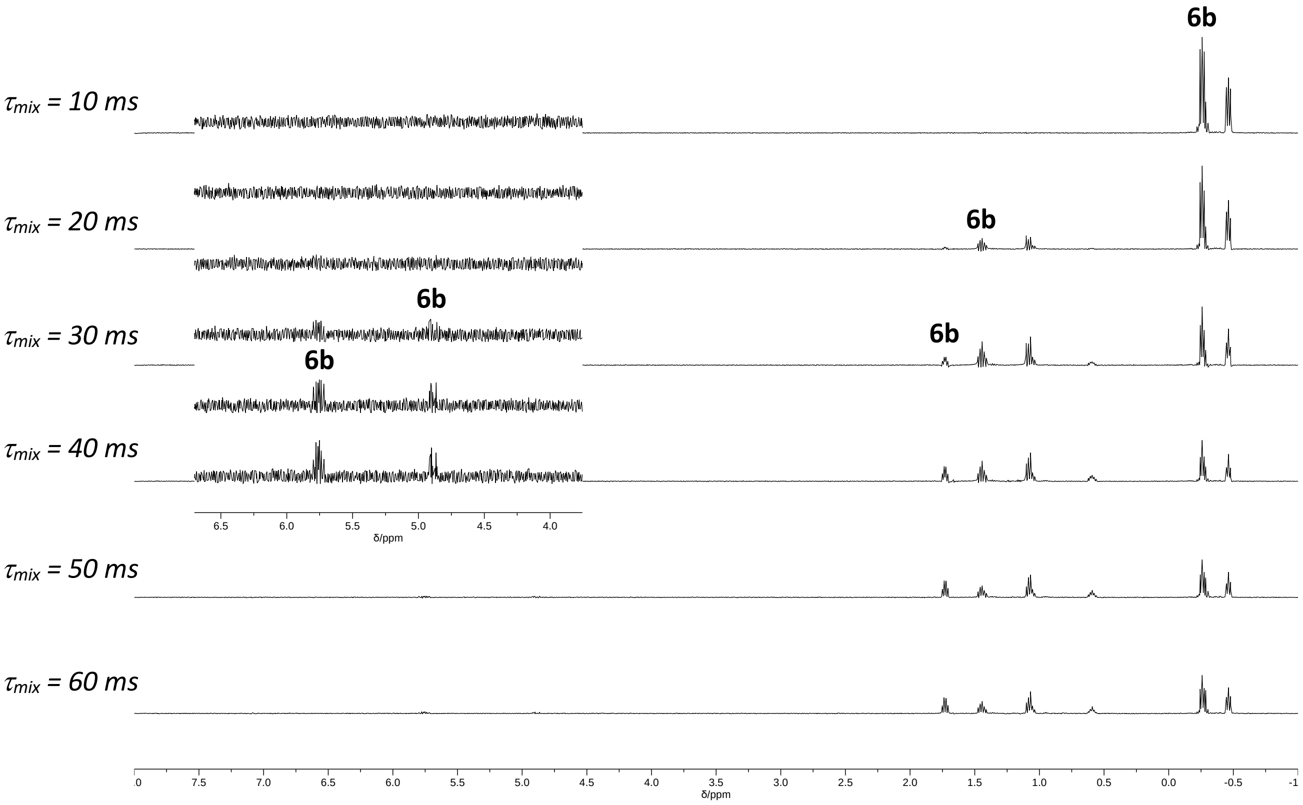

Figure S26. Labelled stacked selective excitation (-0.25 ppm) ^{1}H TOCSY NMR spectra showing CH_x groups along hydrocarbon chain of **6b** (note a small amount of **8b** is also observed)

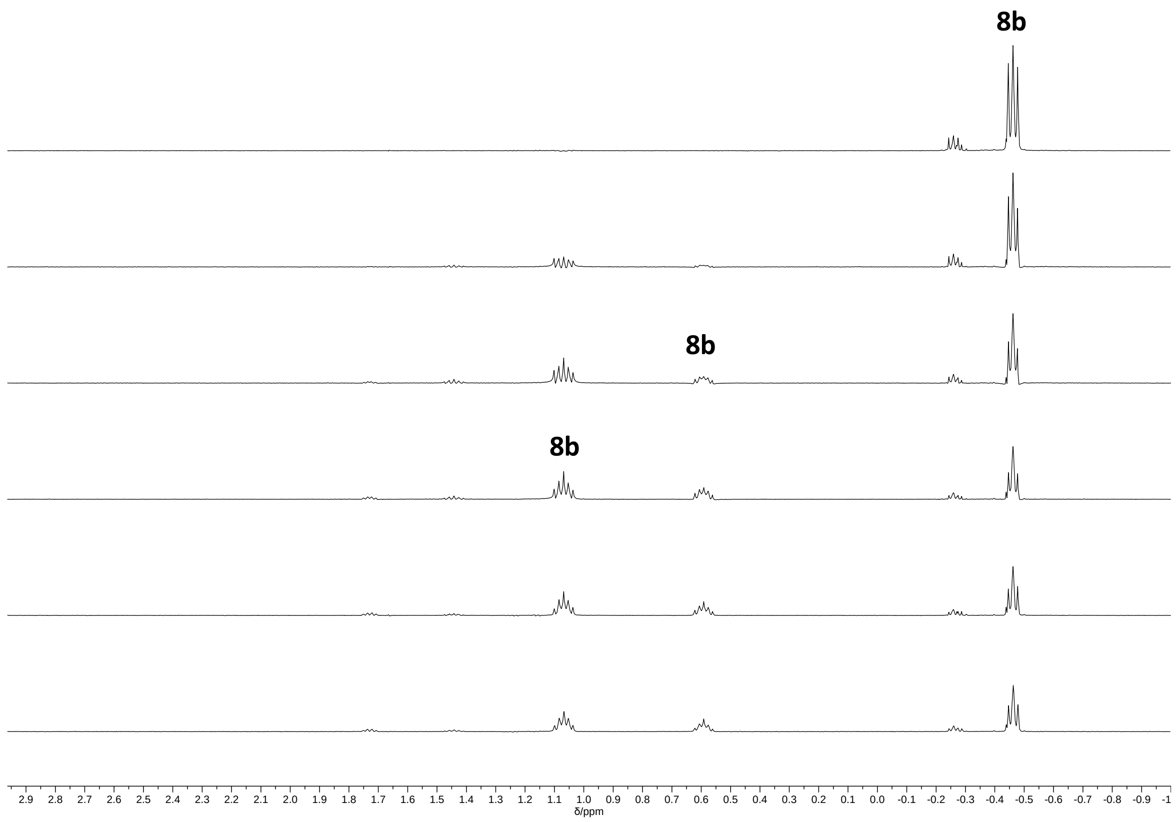

Figure S27. Labelled stacked selective excitation (-0.45 ppm) ^{1}H TOCSY NMR spectra showing CH_2 groups along hydrocarbon chain of **8b** (note a small amount of **6b** is also observed)

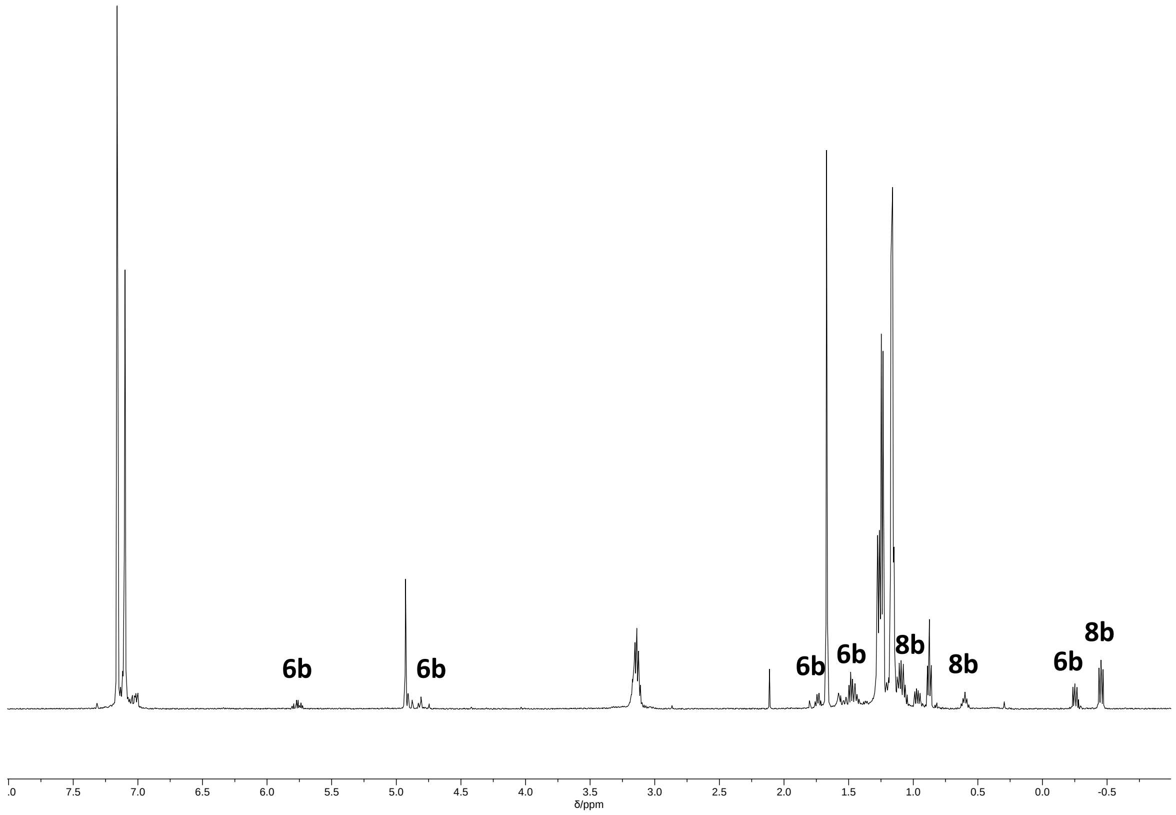

Figure S28. Labelled ^{1}H NMR spectrum of mixture of **6b** and **8b** (obtained from reaction of **1** and **3b** after 5 h at 100 °C)

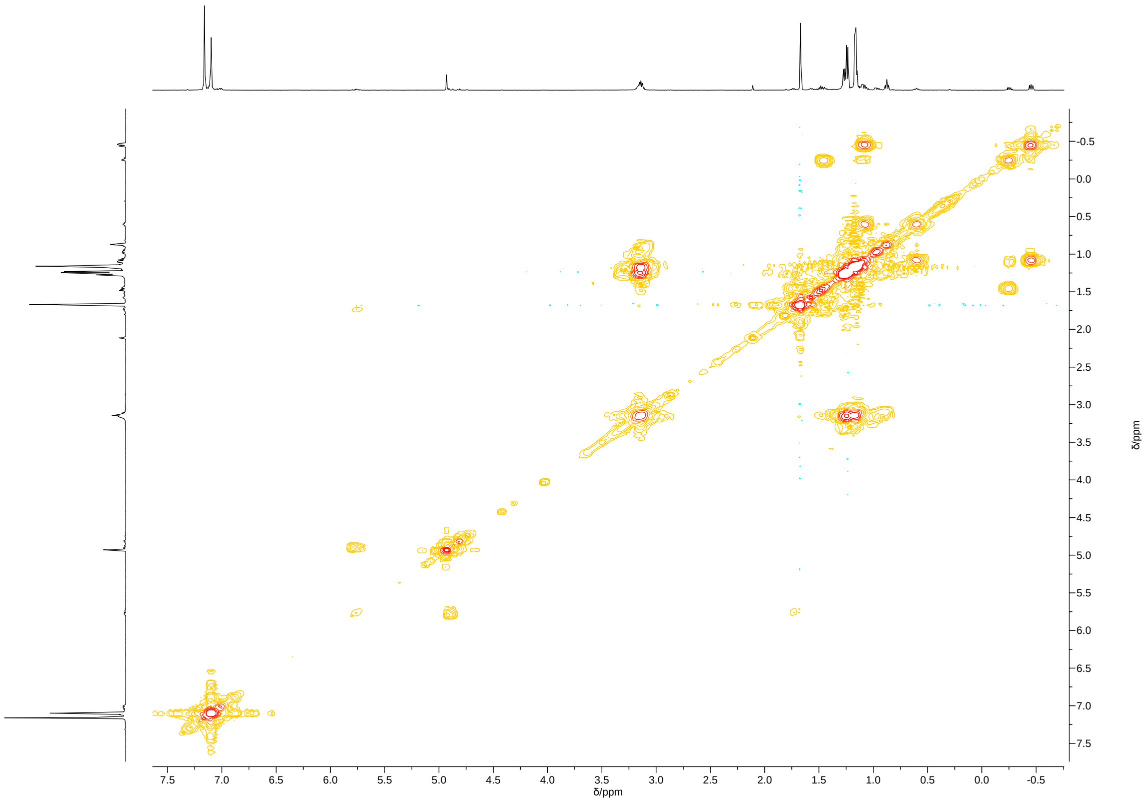

Figure S29. ^{1}H COSY NMR spectrum of mixture of **6b** and **8b** (obtained from reaction of **1** and **3b** after 5 h at 100 °C)

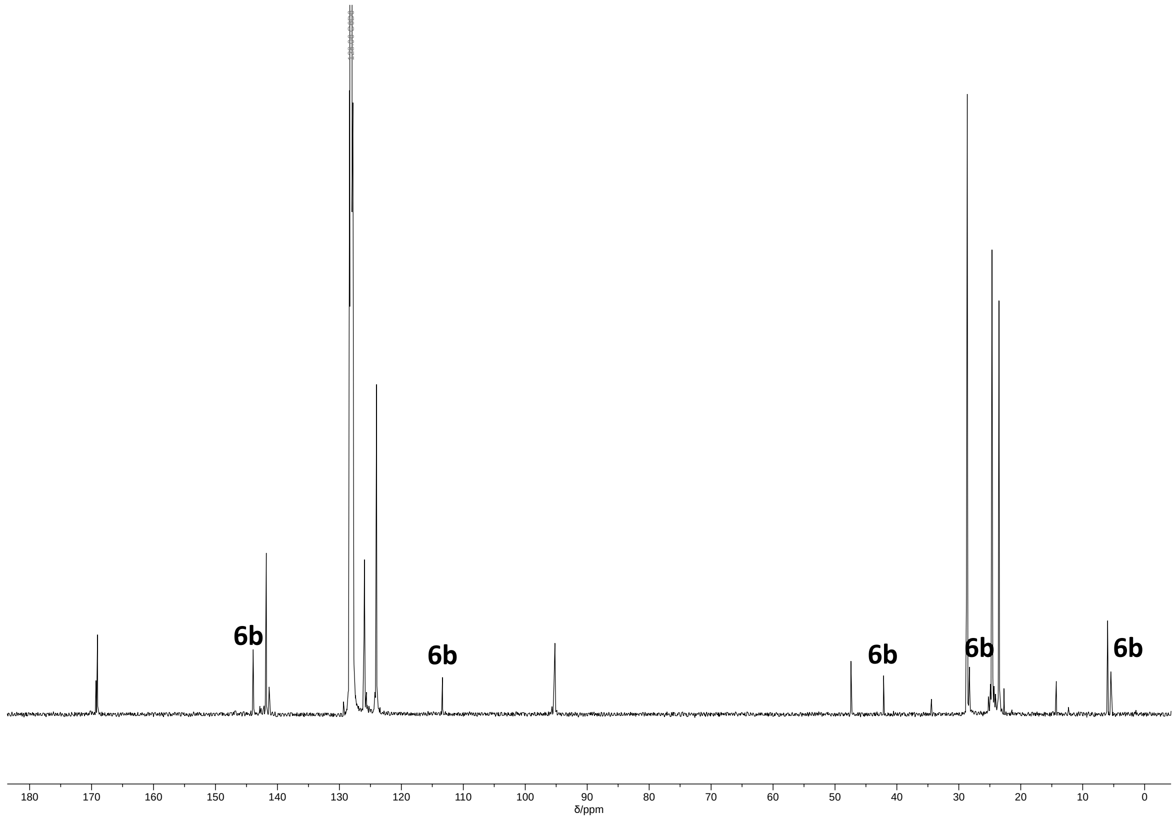

Figure S30. Labelled $^{13}C\{^{1}H\}$ NMR spectrum of mixture of **6b** and **8b** (obtained from reaction of **1** and **3b** after 5 h at 100 °C)

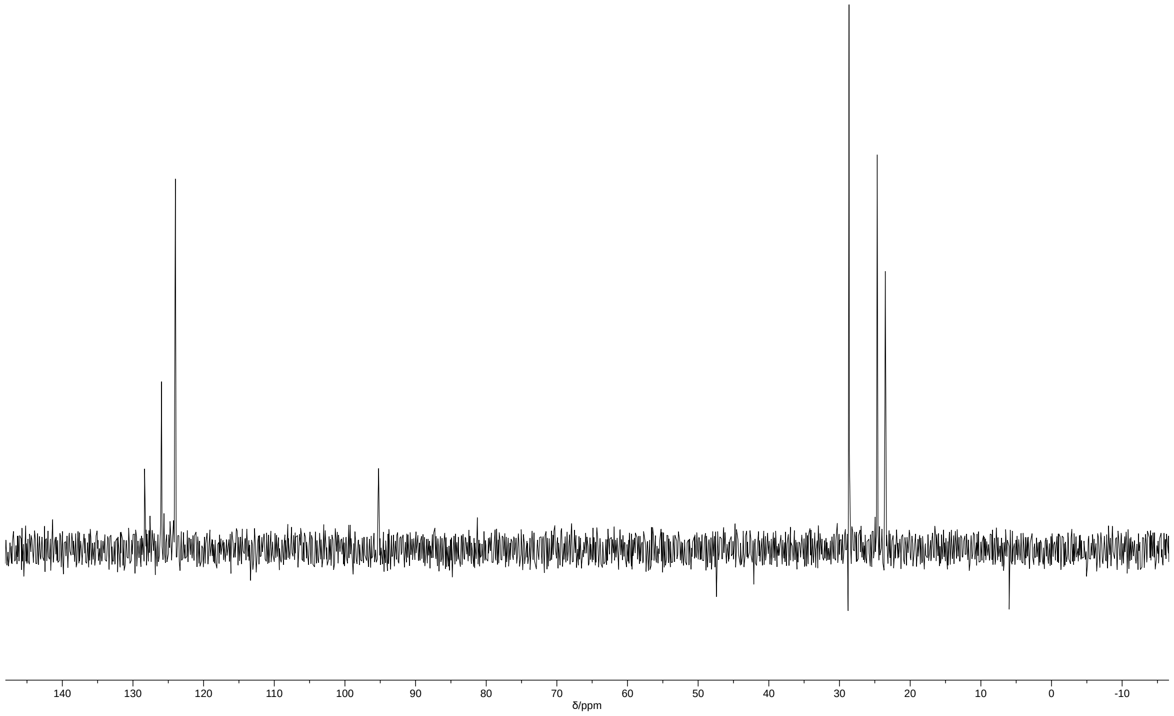

Figure S31. $^{13}C\{^{1}H\}$ DEPT-135 NMR spectrum of mixture of **6b** and **8b** (obtained from reaction of **1** and **3b** after 5 h at 100 °C)

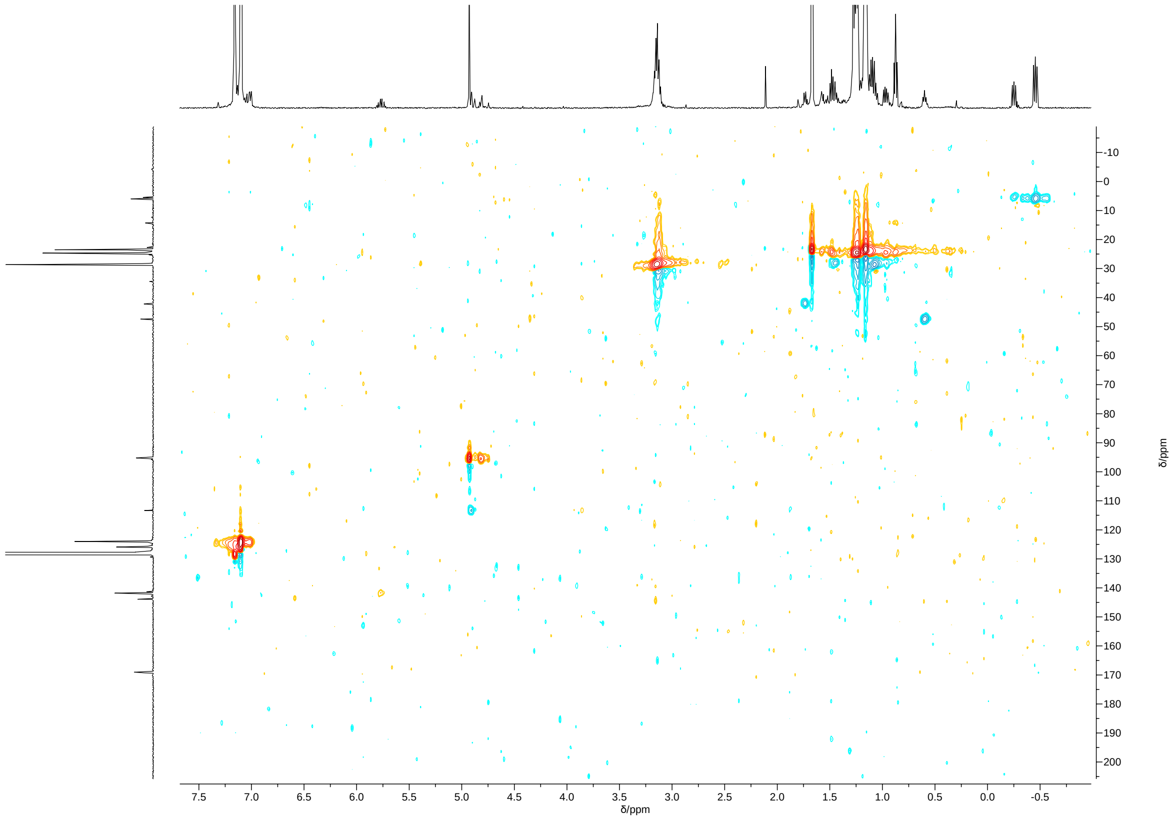

Figure S32. ^{1}H–^{13}C HSQC NMR spectrum of mixture of **6b** and **8b** (obtained from reaction of **1** and **3b** after 5 h at 100 °C)

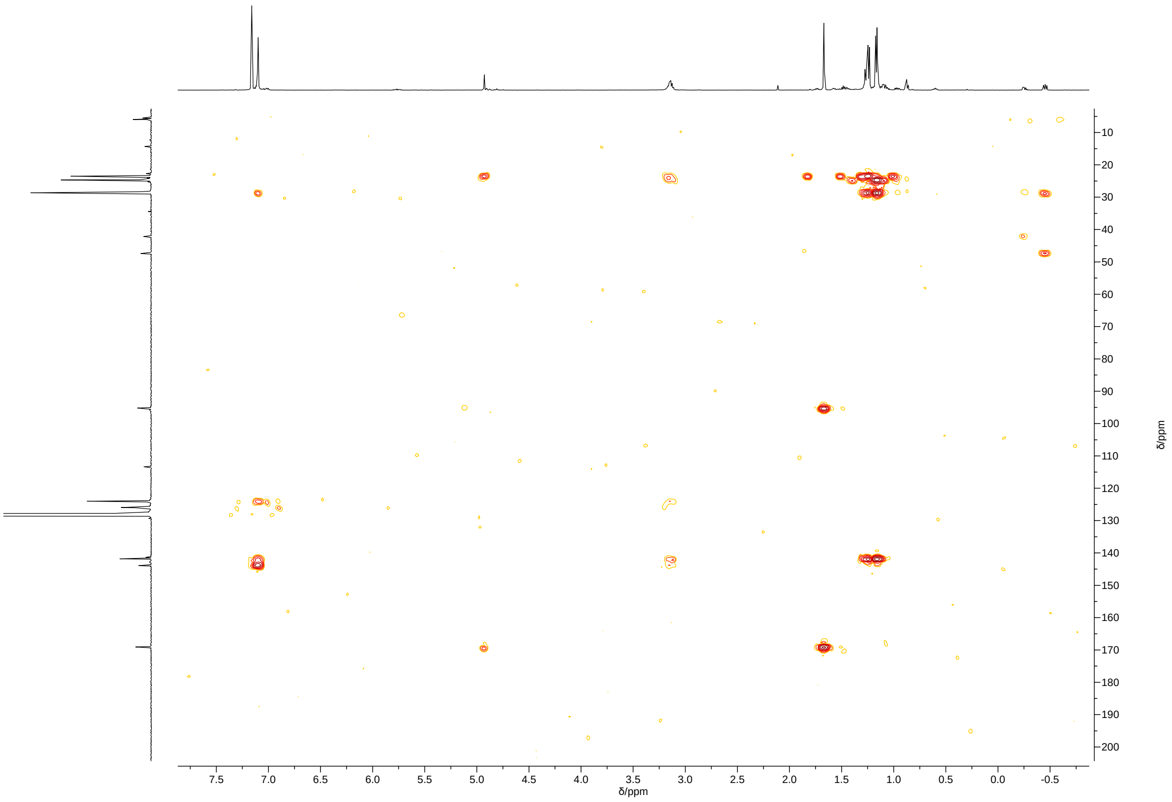

Figure S33. ^{1}H–^{13}C HMBC NMR spectrum of mixture of **6b** and **8b** (obtained from reaction of **1** and **3b** after 5 h at 100 °C)

Synthesis of 7a

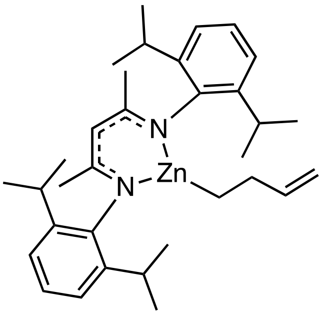

In a N_2 filled glovebox, a suspension of **2** (20 mg, 0.041 mmol) in C_6D_6 (0.6 mL) was added to a J-Young NMR tube. Methylidene cyclopropane (**3a**) was added cold via micropipette and the NMR tube sealed. Note, the high volatility of **3a** (boiling point = 11 ^{o}C) makes accurate addition of this reagent challenging and in practice an excess is added. The resulting colourless solution was heated to 80 °C for 16 hours. After this time, analysis by ^{1}H NMR spectroscopy reveals a large excess of **3a** remained unreacted with **2** had been completely consumed. Additional portions of **2** were added and subsequently heated to 80 °C overnight (16-23 hours) until complete consumption of **3a** was observed *via* ^{1}H NMR spectroscopy. Note, a secondary species was also observed to form in tandem with **7a**, proposed to be the non-ring opened product (**5a**), identified through diagnostic resonances at -0.39 – -0.36 ppm (m, 2H, $CH_2CH(C\mathbf{H}_2)_2$), 0.15 – 0.20 ppm (m, 2H, $CH_2CH(C\mathbf{H}_2)_2$), 0.24 ppm (d, 2H, $^{3}J_{H\text{-}H}$ = 7.20 Hz, Zn–C**H**$_2$) and 0.35 – 0.43 ppm (m, 1H $CH_2C\mathbf{H}(CH_2)_2$). The high field region of the ^{1}H NMR spectrum of an approximate 1:3 mixture of **5a**:**7a** is presented below, alongside a selective excitation TOCSY spectrum of the same region (excited at -0.37 ppm) which shows these resonances are all part of the same spin system (Figure S34). **5a** can be converted to **7a** with extended heating (~2 weeks) at 100 °C. The J-Young NMR tube was returned to the glovebox, the solution diluted with toluene (0.5 mL) and decanted into a scintillation vial. The volatiles were removed in *vacuo*, leaving an off-white residue. The residue was dissolved in n-pentane (2 x 0.5 mL) and filtered. The volatiles were removed in *vacuo*, leaving **7a** as an analytically pure white solid. Yield: 65 mg, 0.12 mmol, 92 %. HSQC NMR experiment was used to determine alkene ^{1}H and ^{13}C resonances observed.

^{1}H NMR (400 MHz, C_6D_6) δ: 0.31 (m, 2H, Zn–C**H**$_2$), 1.15 (d, $^{3}J_{H\text{-}H}$ = 7.1 Hz, 12H, $CH(C\mathbf{H}_3)_2$), 1.26 (d, $^{3}J_{H\text{-}H}$ = 7.1 Hz, 12H, $CH(C\mathbf{H}_3)_2$), 1.70 (s, 6H, $NC(C\mathbf{H}_3)$), 1.85-1.92 (m, 2H, $ZnCH_2C\mathbf{H}_2$), 3.18 (sept., 4H, $^{3}J_{H\text{-}H}$ = 7.1 Hz, $C\mathbf{H}(CH_3)_2$), 4.68-4.83 (m, 2H, $ZnCH_2CH_2CHC\mathbf{H}_2$), 5.00 (s, 1H, $(CH_3)C(C\mathbf{H})C(CH_3)$), 5.52-5.62 (m, 1H, $ZnCH_2CH_2C\mathbf{H}$), 7.11 (m, 6H, Ar-**H**).

^{13}C NMR (101 MHz, C_6D_6) δ: 6.9 (Zn-**C**H$_2$), 23.4 ($NC(\mathbf{C}H_3)$), 23.6 ($CH(\mathbf{C}H_3)_2$), 24.3 ($CH(\mathbf{C}H_3)_2$), 28.5 ($\mathbf{C}H(CH_3)_2$), 32.2 ($ZnCH_2\mathbf{C}H_2$), 95.5 ($(CH_3)C(\mathbf{C}H)C(CH_3)$), 110.9 ($ZnCH_2CH_2CH\mathbf{C}H_2$), 123.9 (*meta*-Ar-**C**H), 126.1 (*para*-Ar-**C**H), 141.6 (*ortho*-Ar-**C**), 145.0 ($ZnCH_2CH_2\mathbf{C}H$), 145.2 (*ipso*-Ar-**C**), 167.6 ($N\mathbf{C}(CH_3)$).

FT-IR (cm^{-1}): 3059, 3024, 2956, 2921, 2865, **1633 (C=C stretch)**, 1550, 1523, 1436, 1381, 1360, 1317, 1263, 1251, 1230, 1176, 1100, 1054, 1022, **990 (out-of-plane C-H bend)**, 934, **902 (out-of-plane C-H bend)**, 858, 794, 756, 719, 704, 671, 636, 605, 555, 529, 434.

Anal. Calcd. ($C_{33}H_{48}N_2Zn$): C, 73.65; H, 8.99; N, 5.21. Found: C, 71.93; H, 8.37; N, 5.18. Note, the highly air-sensitive nature of this compound prevented collection of accurate elemental analysis.

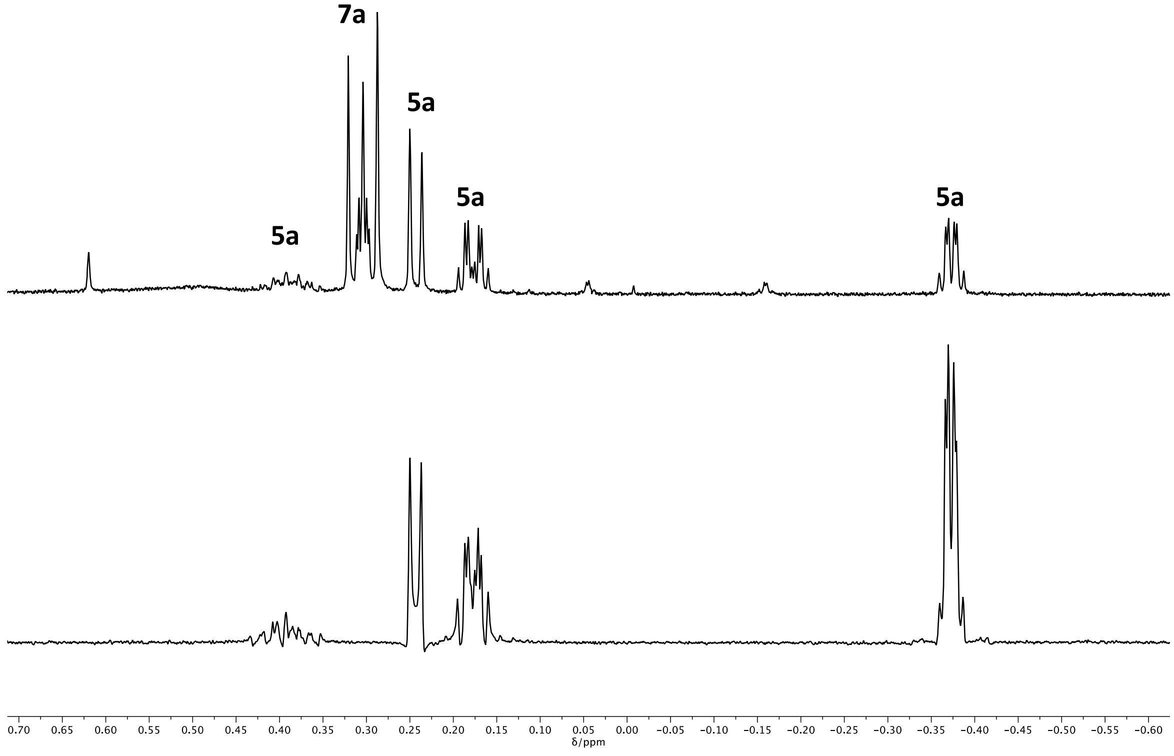

Figure S34. Stacked high field region of the ^{1}H NMR spectra (-0.60 – 0.7 ppm) of a 1:3 mixture of **5a**:**7a** (top) and a selective excitation TOCSY with excitation at -0.37 ppm (bottom)

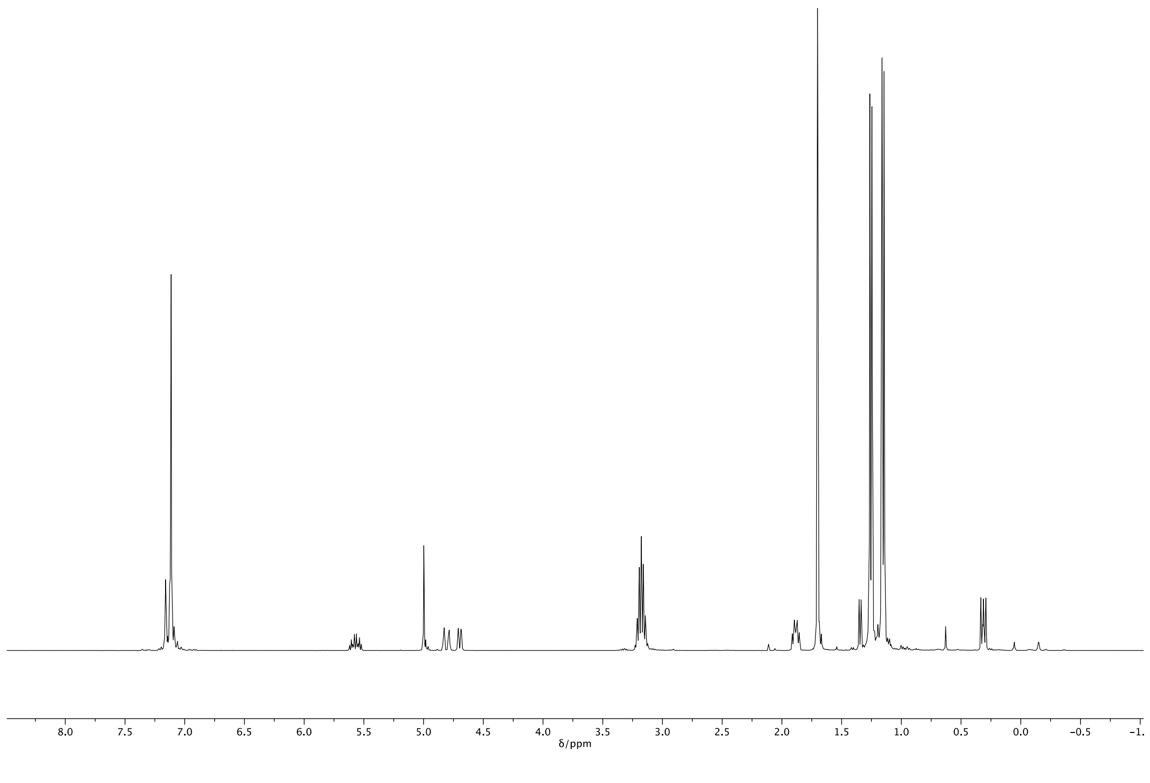

Figure S35. ^{1}H NMR spectrum of **7a**

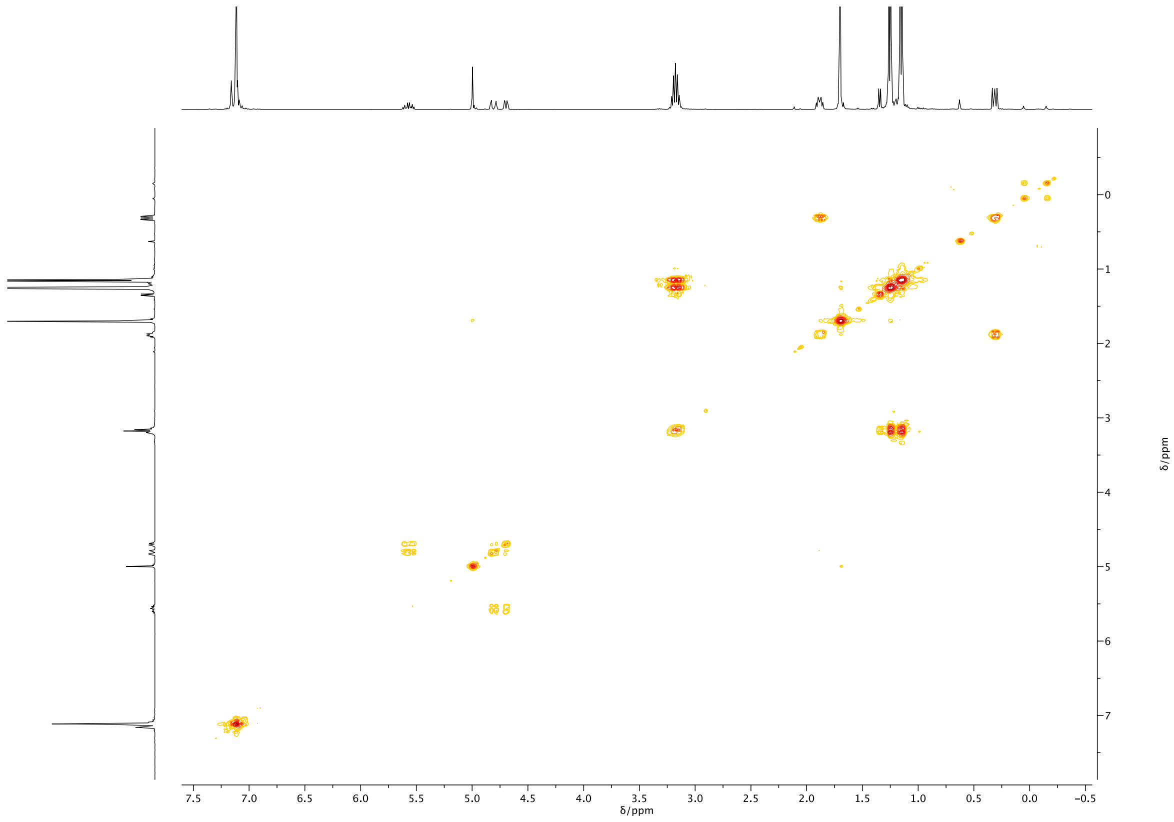

Figure S36. ^{1}H COSY NMR spectrum of **7a**

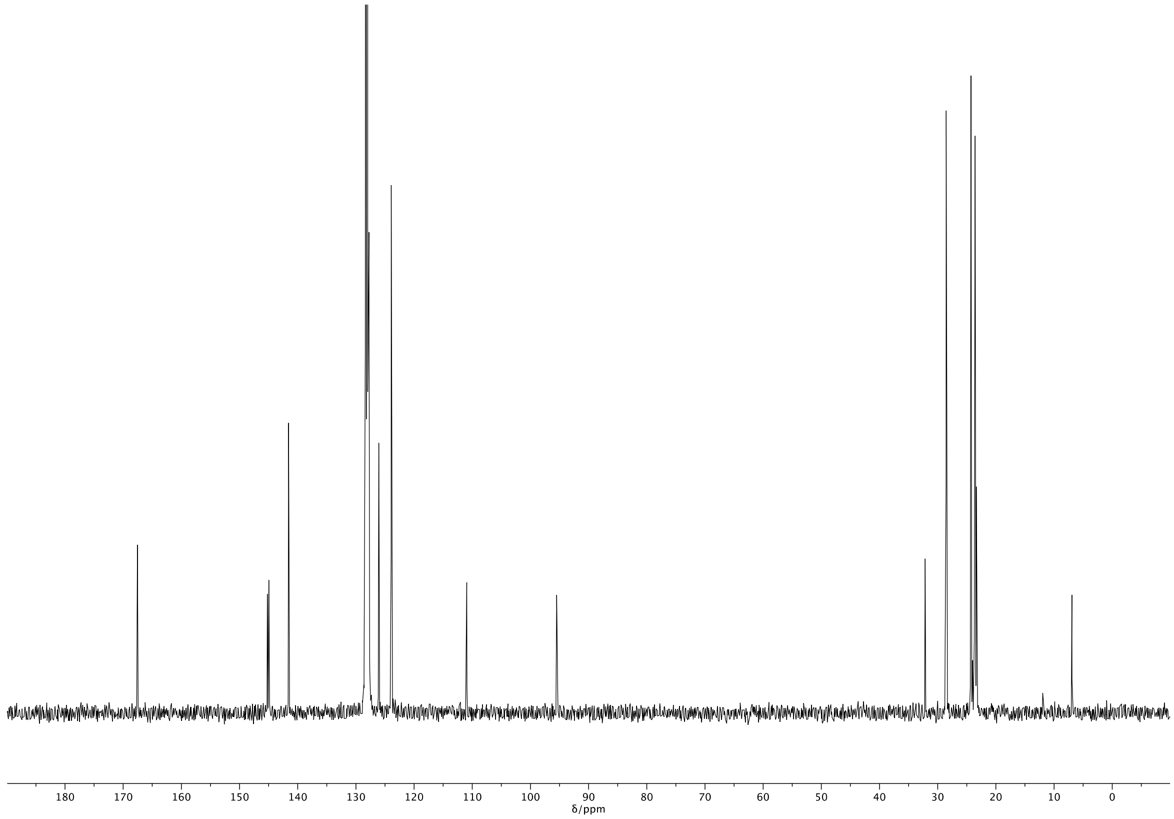

Figure S37. $^{13}C\{^{1}H\}$ NMR spectrum of **7a**

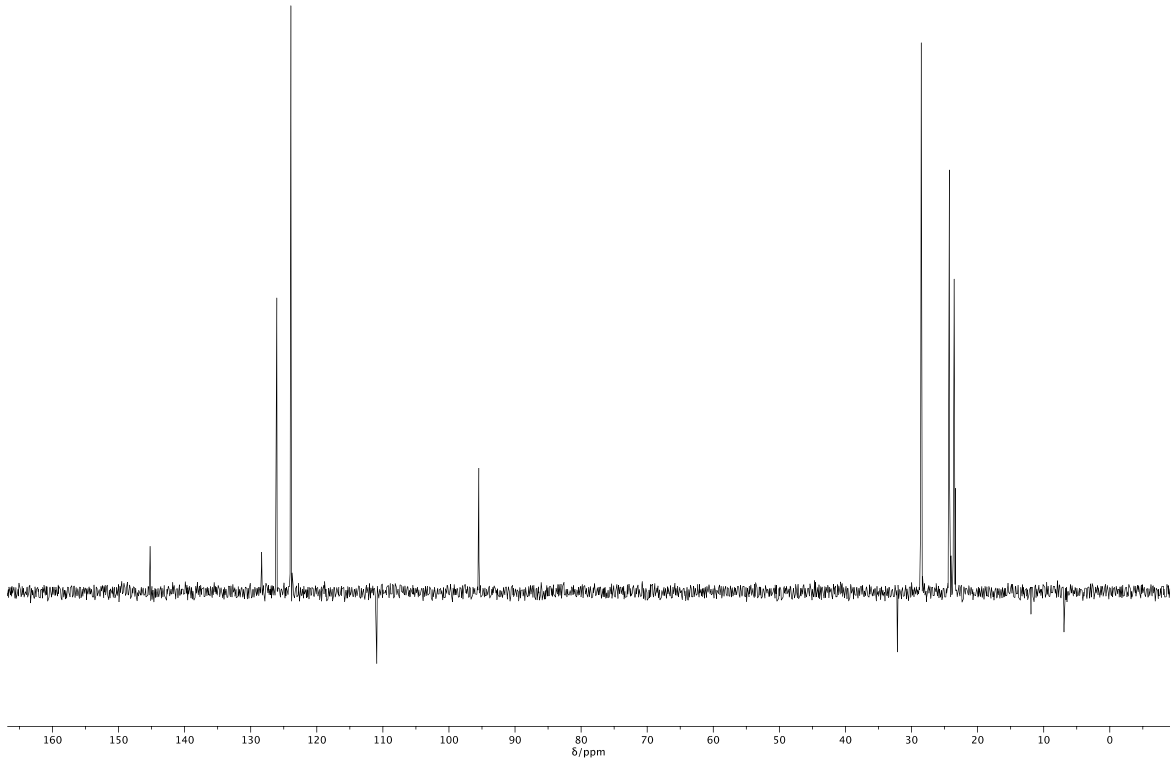

Figure S38. $^{13}C\{^{1}H\}$ DEPT-135 NMR spectrum of **7a**

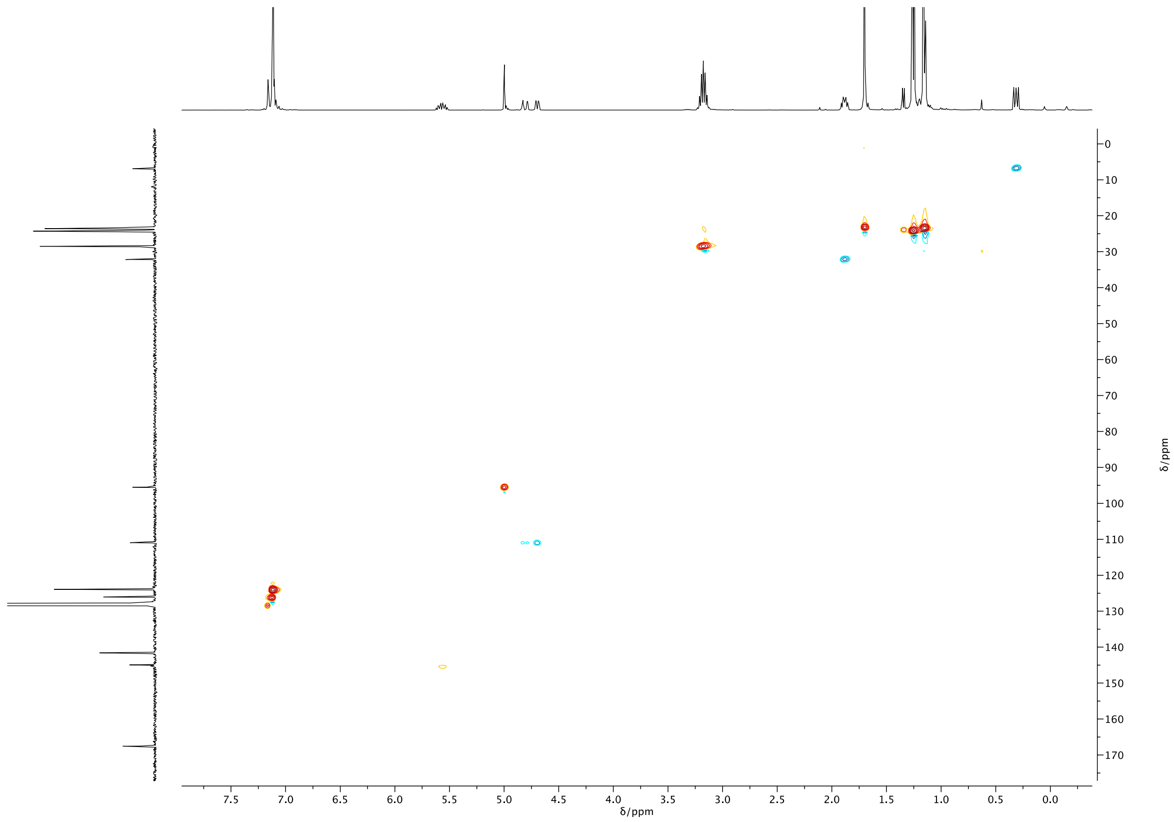

Figure S39. $^{1}H–^{13}C$ HSQC NMR spectrum of **7a**

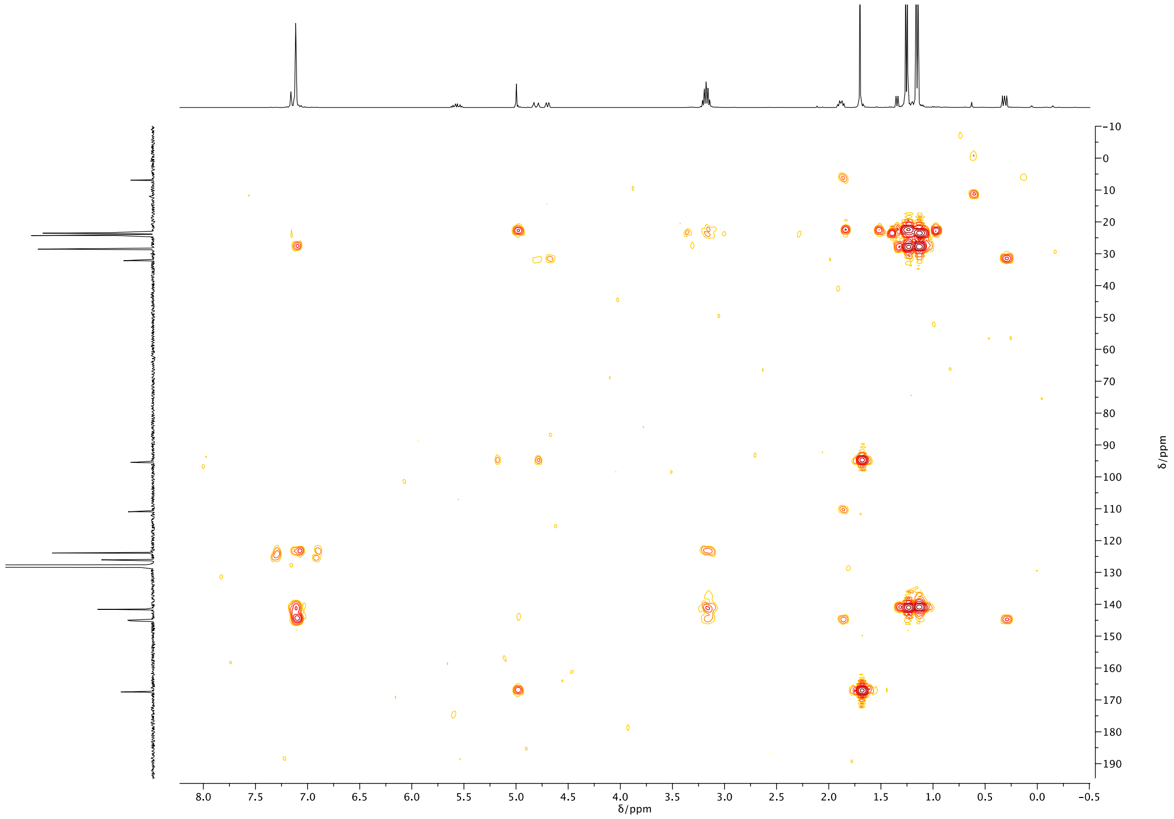

Figure S40. ^{1}H–^{13}C HMBC NMR spectrum of **7a**

Synthesis of 8b

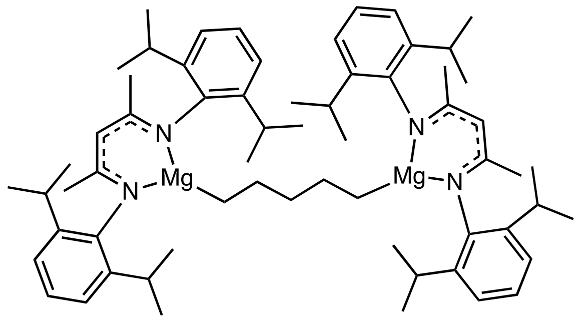

In a N_2 filled glovebox, a suspension of **1** (25 mg, 0.057 mmol) in C_6D_6 (0.8 mL) was added to a J-Young NMR tube. Methylidene cyclobutene (**3b**) (2.6 μL, 2.0 mg, 0.057 mmol) was added via micropipette and the NMR tube sealed. The resulting pale-yellow solution was heated to 120 °C for 16 hours, or until the complete consumption of **1** was observed *via* ^{1}H NMR spectroscopy. The J-Young NMR tube was returned to the glovebox, the solution diluted with toluene (0.5 mL) and decanted into a scintillation vial. The volatiles were removed in *vacuo*, leaving an off-white crystalline solid. The solid was dissolved in n-pentane (2 x 0.5 mL) and filtered. The volatiles were removed in *vacuo*, leaving **8b** as a colourless solid contaminated with **1**. All attempts to separate this mixture by crysallisation were unsuccessful. Yield: 54.0 mg.

^{1}H NMR (400 MHz, C_6D_6) δ: -0.45 (t, $^{3}J_{H-H}$ = 8.0 Hz, 4H, Mg–C**H**$_2$), 0.60 (m, 4H, MgCH$_2$CH$_2$C**H$_2$**), 1.07 (m, 2H, MgCH$_2$C**H$_2$**), 1.17 (d, $^{3}J_{H-H}$ = 6.9 Hz, 24H, CH(C**H**$_3$)$_2$), 1.24 (d, $^{3}J_{H-H}$ = 6.4 Hz, 24H, CH(C**H**$_3$)$_2$), 1.67 (s, 12H, NC(C**H**$_3$)), 3.14 (sept, $^{3}J_{H-H}$ = 6.6 Hz, 8H, C**H**(CH$_3$)$_2$), 4.93 (s, 2H, (CH$_3$)C(C**H**)C(CH$_3$)), 7.08-7.13 (m, 12H, Ar-**H**).

^{13}C NMR (101 MHz, C_6D_6) δ: 6.0 (Mg-**C**H$_2$), 23.5 (CH(**C**H$_3$)$_2$), 23.6 (NC**C**H$_3$), 24.7 (CH(**C**H$_3$)$_2$), 28.7 (**C**H(CH$_3$)$_2$), 28.8 (MgCH$_2$**C**H$_2$), 47.4 (MgCH$_2$CH$_2$**C**H$_2$), 95.2 ((**C**H)C(CH$_3$)$_2$), 124.0 (*para*-Ar-**C**H), 126.0 (*meta*-Ar-**C**H), 141.8 (*ortho*-Ar-**C**), 143.9 (*ipso*-Ar-**C**), 169.0 (N**C**(CH$_3$)).

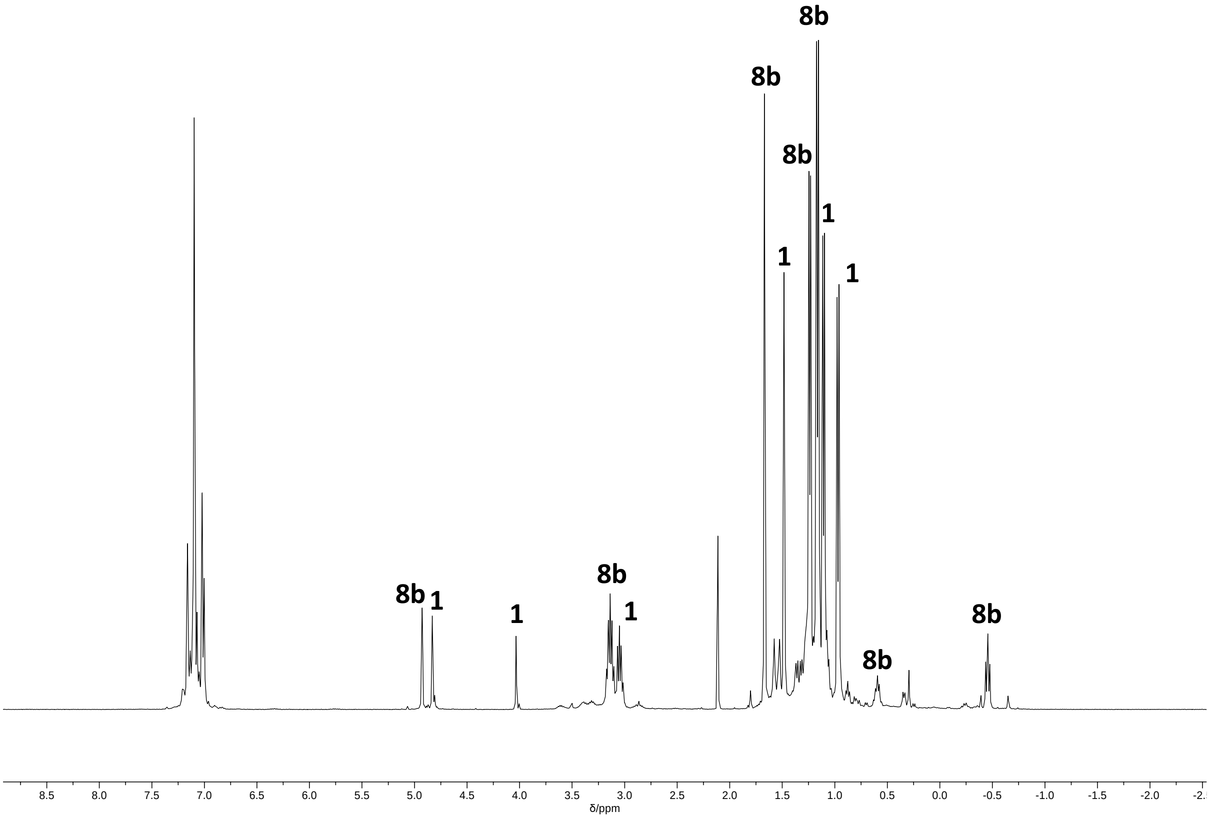

Figure S41. ^{1}H NMR spectrum of **8b** (with **1** impurity)

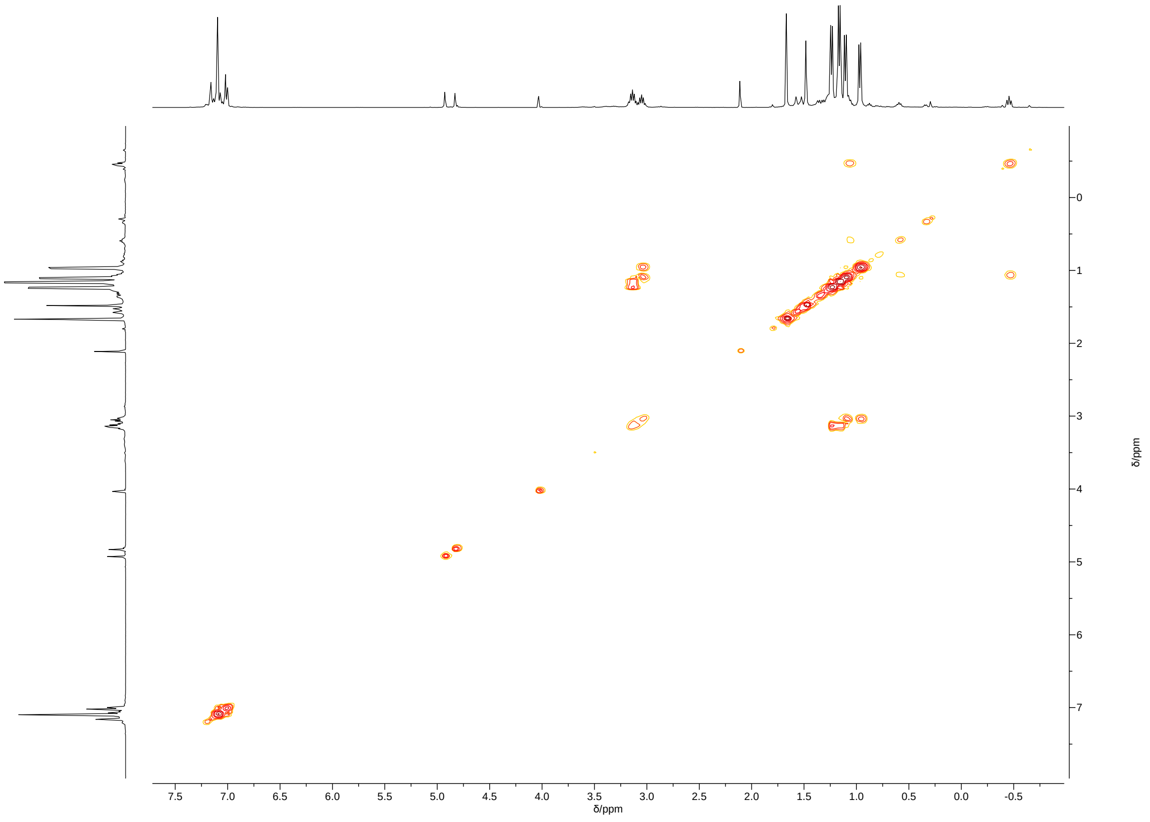

Figure S42. ^{1}H COSY NMR spectrum of **8b** (with **1** impurity)

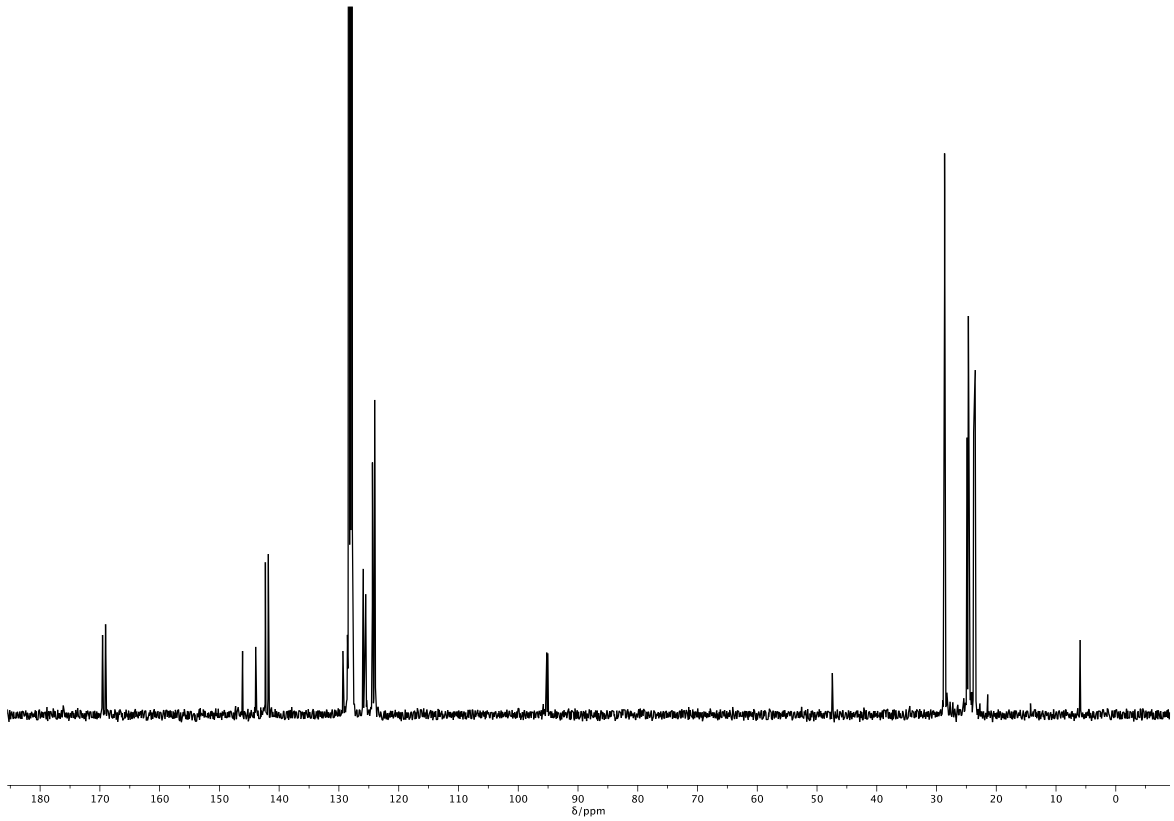

Figure S43. $^{13}C\{^{1}H\}$ NMR spectrum of **8b** (with **1** impurity)

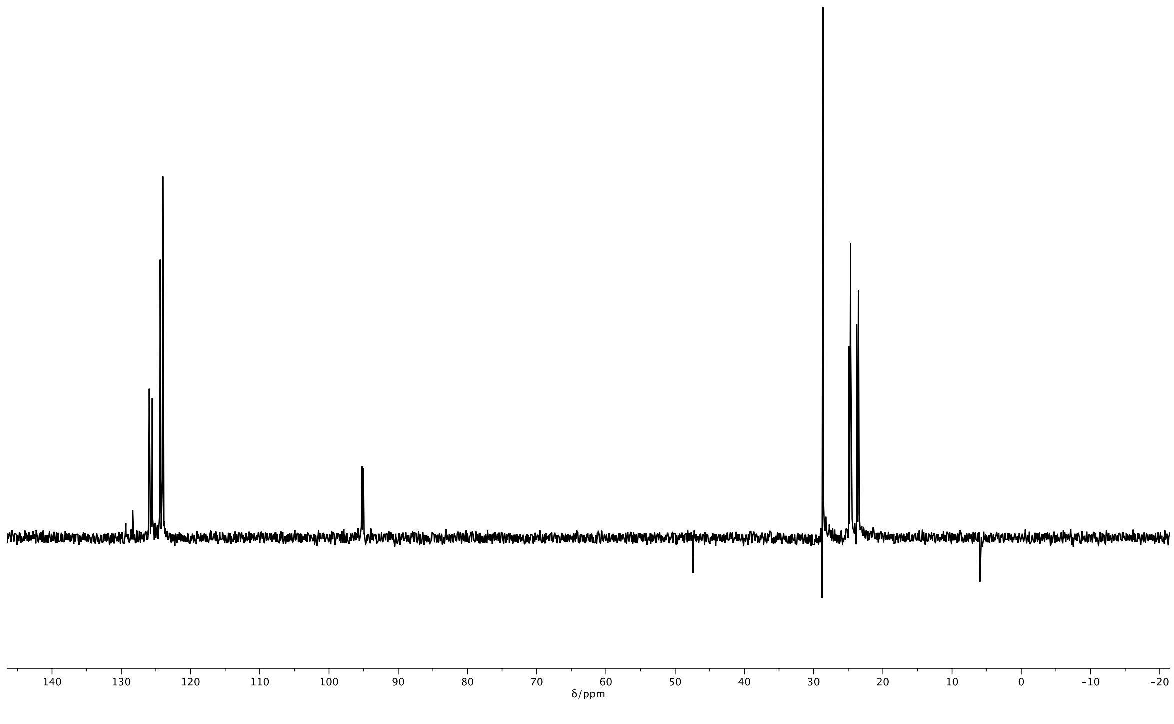

Figure S44. $^{13}C\{^{1}H\}$ DEPT-135 NMR spectrum of **8b** (with **1** impurity)

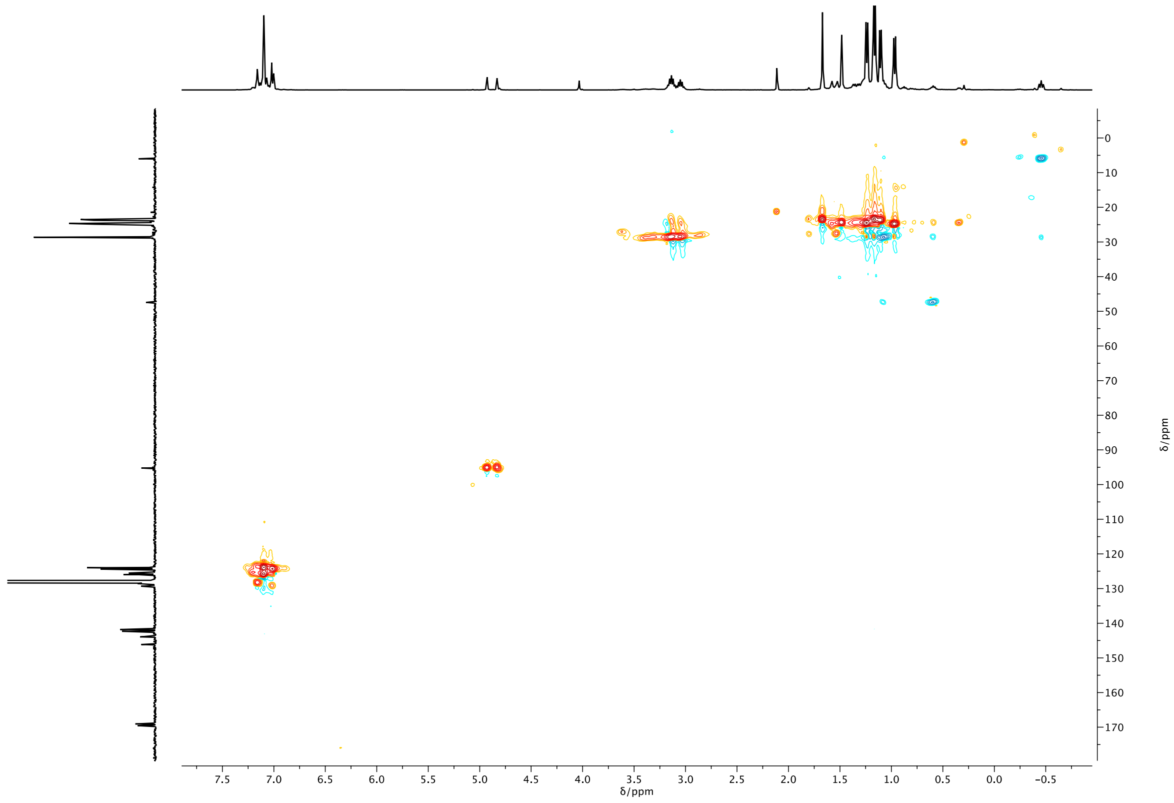

Figure S45. ^{1}H–^{13}C HSQC NMR spectrum of **8b** (with **1** impurity)

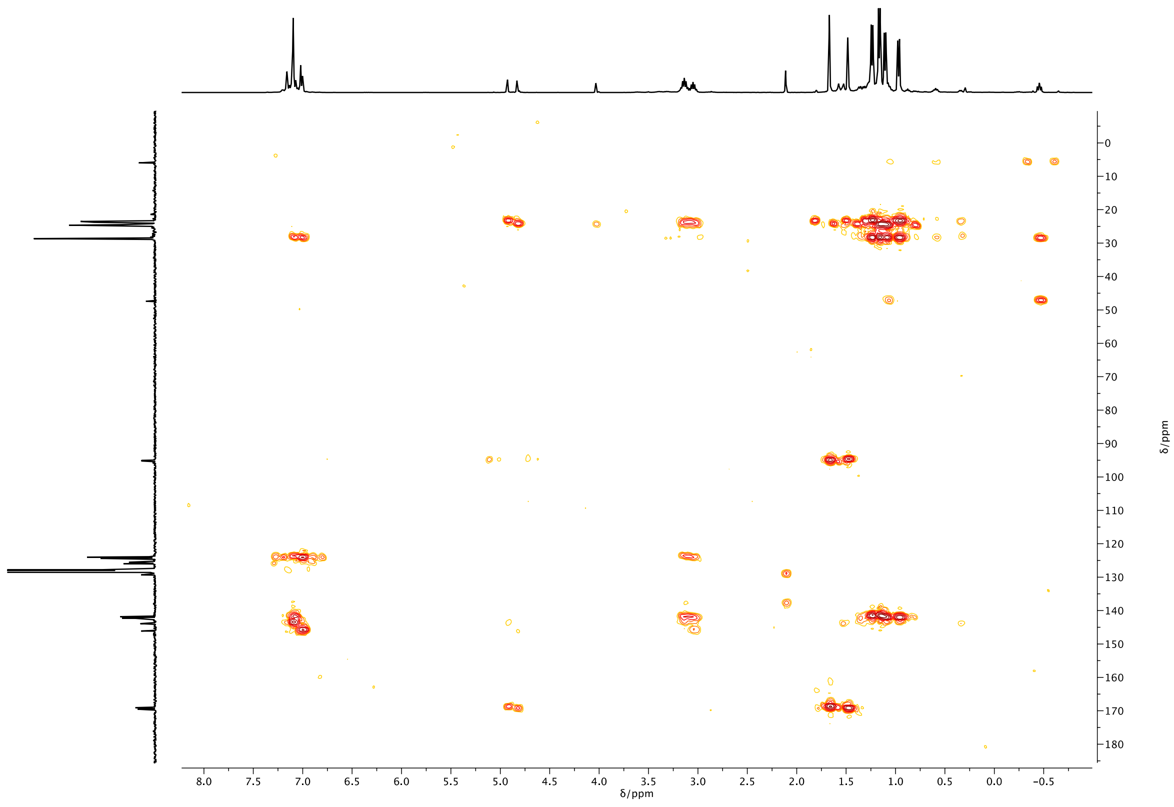

Figure S46. ^{1}H–^{13}C HMBC NMR spectrum of **8b** (with **1** impurity)

Synthesis of 9a

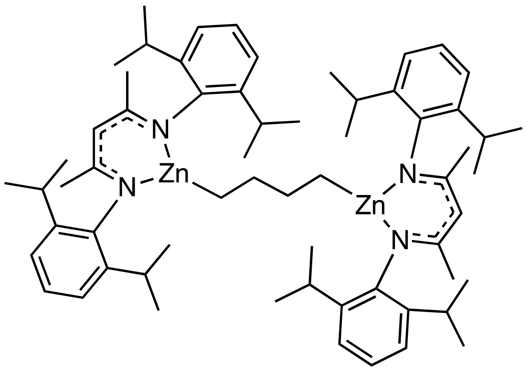

Note, **9a** can be prepared directly from **2** and **3a**, but the high volatility of **3a** (see above) makes ensuring stoichiometric control challenging. Consequently, **9a** is more conveniently prepared from **7a**.

In a N_2 filled glovebox, **7a** (34.5 mg, 0.064 mmol) in C_6D_6 (0.6 mL) was added to a J-Young NMR tube. **2** (32.6 mg, 0.067 mmol) was added as a solid and the solution heated to 100 °C for 48 hours, or until the complete consumption of **7a** was observed *via* ^{1}H NMR spectroscopy. The J-Young NMR tube was returned to the glovebox, the solution diluted with toluene (0.5 mL) and decanted into a scintillation vial. The volatiles were removed in *vacuo*, leaving an off-white crystalline solid. The solid was dissolved in n-pentane (2 x 0.5 mL) and filtered. The volatiles were removed in *vacuo*, leaving **9a** as an analytically pure white solid. Yield: 58.7 mg, 0.057 mmol, 90%.

^{1}H NMR (400 MHz, C_6D_6) δ: -0.08 (m, 4H, Zn–C**H**$_2$), 0.69 (m, 4H, Zn–CH$_2$C**H**$_2$), 1.15 (d, $^3J_{H\text{-}H}$ = 6.9 Hz, 24H, CH(C**H**$_3$)$_2$), 1.19 (d, $^3J_{H\text{-}H}$ = 7.1 Hz, 24H, CH(C**H**$_3$)$_2$), 1.69 (s, 12H, NC(C**H**$_3$)), 3.13 (sept., 8H, $^3J_{H\text{-}H}$ = 6.9 Hz, C**H**(CH$_3$)$_2$), 4.96 (s, 2H, (CH$_3$)C(C**H**)C(CH$_3$)), 7.06-7.13 (m, 12H, Ar-**H**).

^{13}C NMR (101 MHz, C_6D_6) δ: 7.0 (Zn-**C**H$_2$), 23.4 (NC(**C**H$_3$)), 23.6 (CH(**C**H$_3$)$_2$), 24.3 (CH(**C**H$_3$)$_2$), 28.5 (**C**H(CH$_3$)$_2$), 34.7 (ZnCH$_2$**C**H$_2$), 95.2 ((CH$_3$)C(**C**H)C(CH$_3$)), 123.7 (*meta*-Ar-**C**H), 125.8 (*para*-Ar-**C**H), 141.5 (*ortho*-Ar-**C**), 145.1 (*ipso*-Ar-**C**), 167.1 (N**C**(CH$_3$)).

FT-IR (cm^{-1}): 3060, 2956, 2922, 2900, 2864, 1808, 1548, 1523, 1457, 1434, 1381, 1359, 1317, 1266, 1234, 1180, 1098, 1057, 1027, 1019, 934, 859, 792, 757, 718, 704, 634, 527, 447, 434.

Anal. Calcd. ($C_{62}H_{90}N_4Zn_2$): C, 72.85; H, 8.88; N, 5.48. Found: C, 72.88; H, 8.95; N, 5.37.

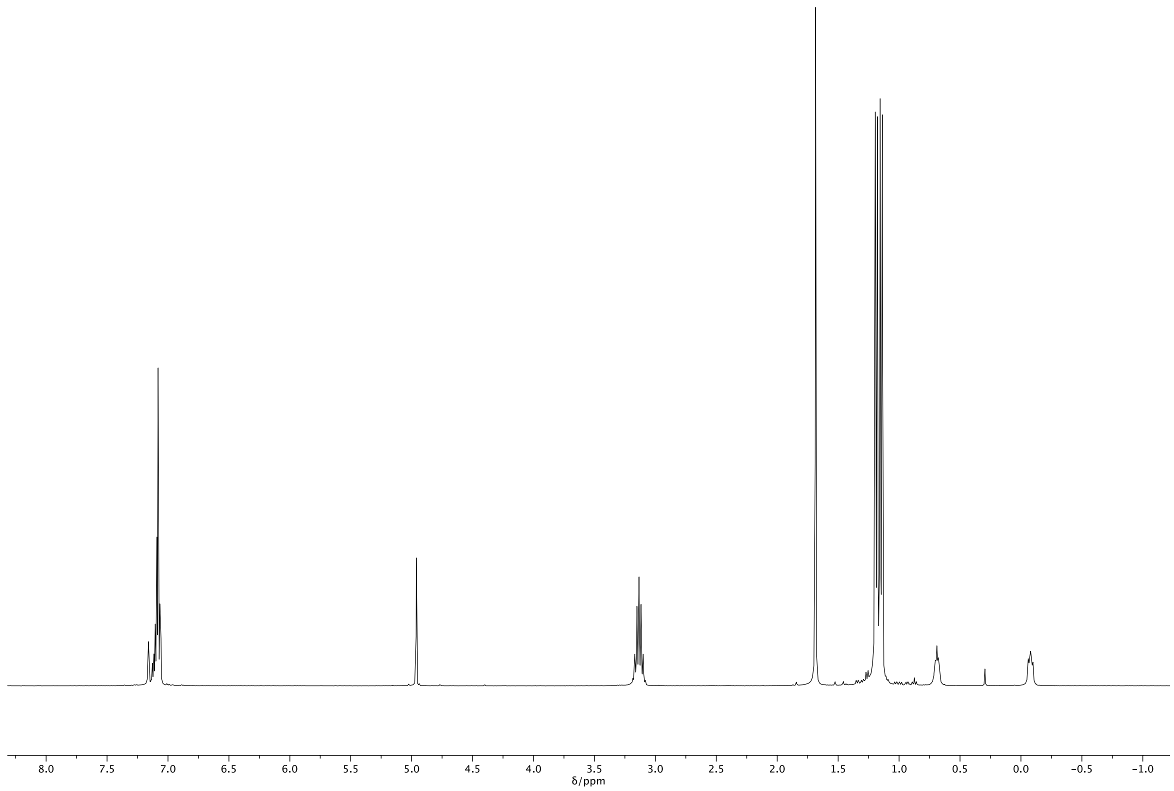

Figure S47. ^{1}H NMR spectrum of **9a**

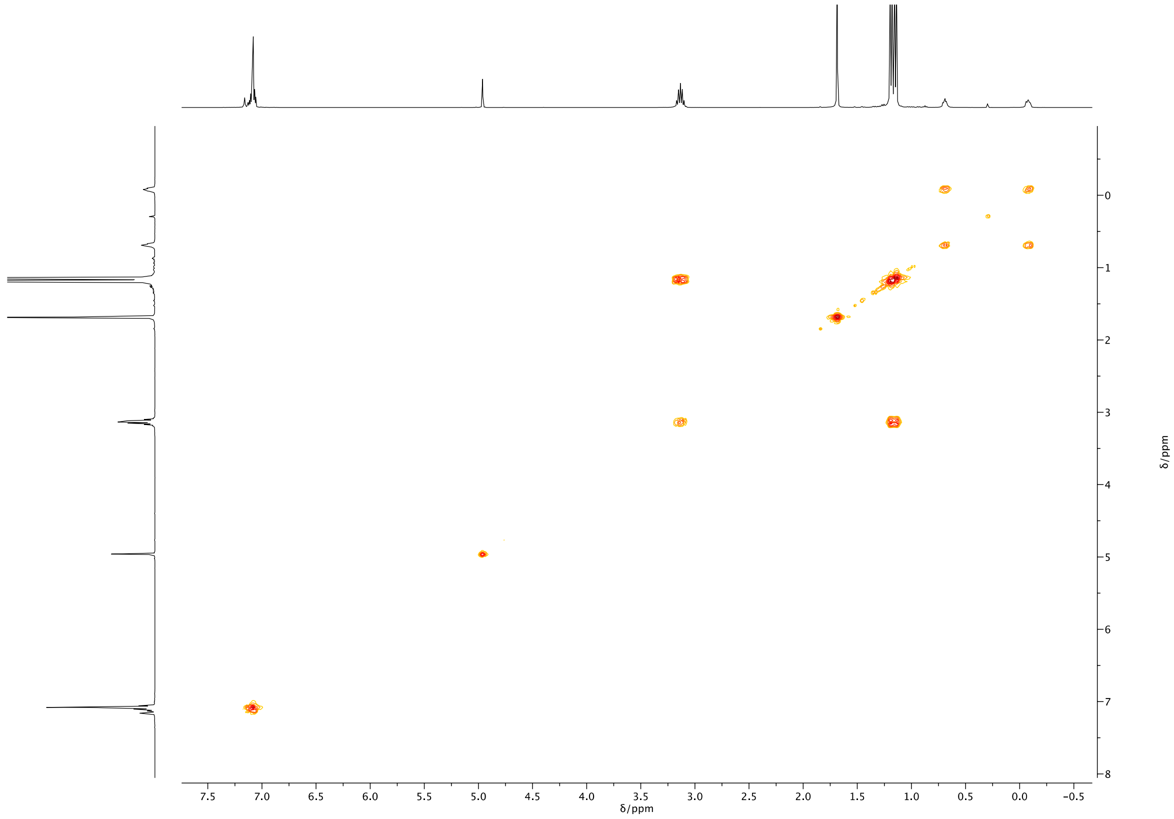

Figure S48. ^{1}H COSY NMR spectrum of **9a**

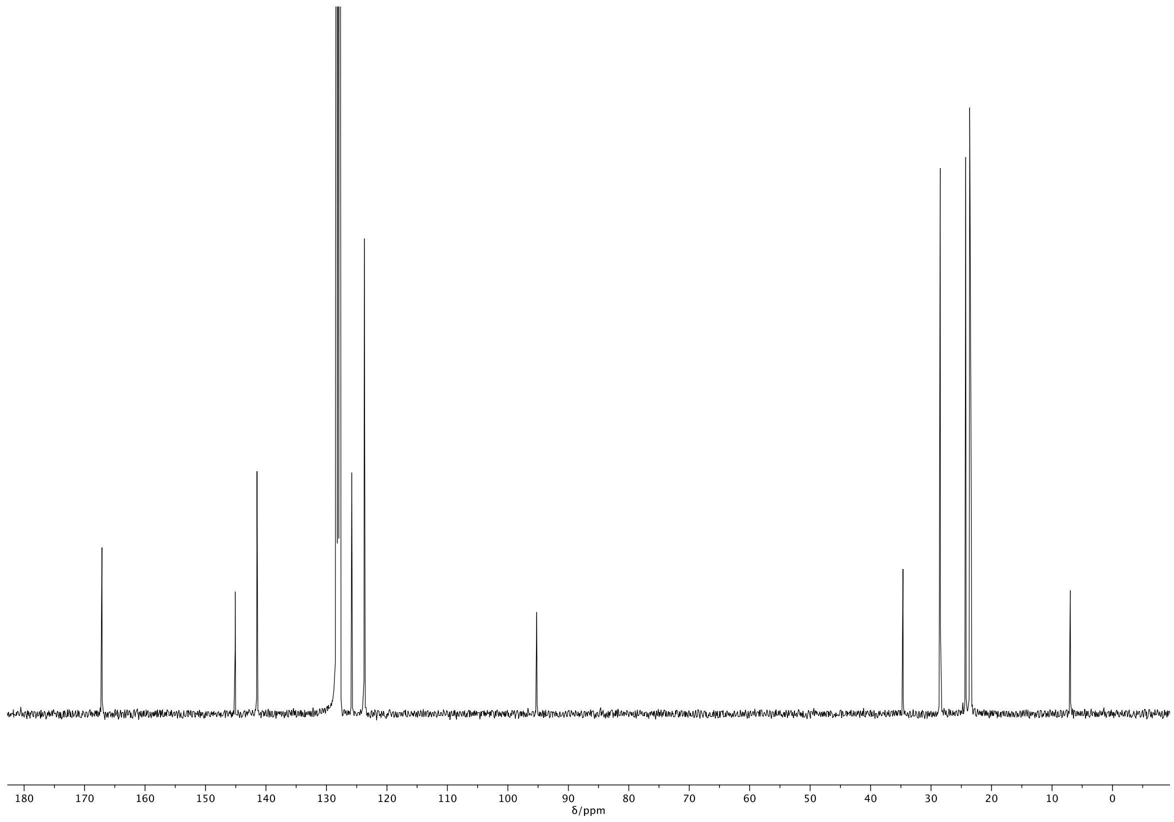

Figure S49. $^{13}C\{^{1}H\}$ NMR spectrum of **9a**

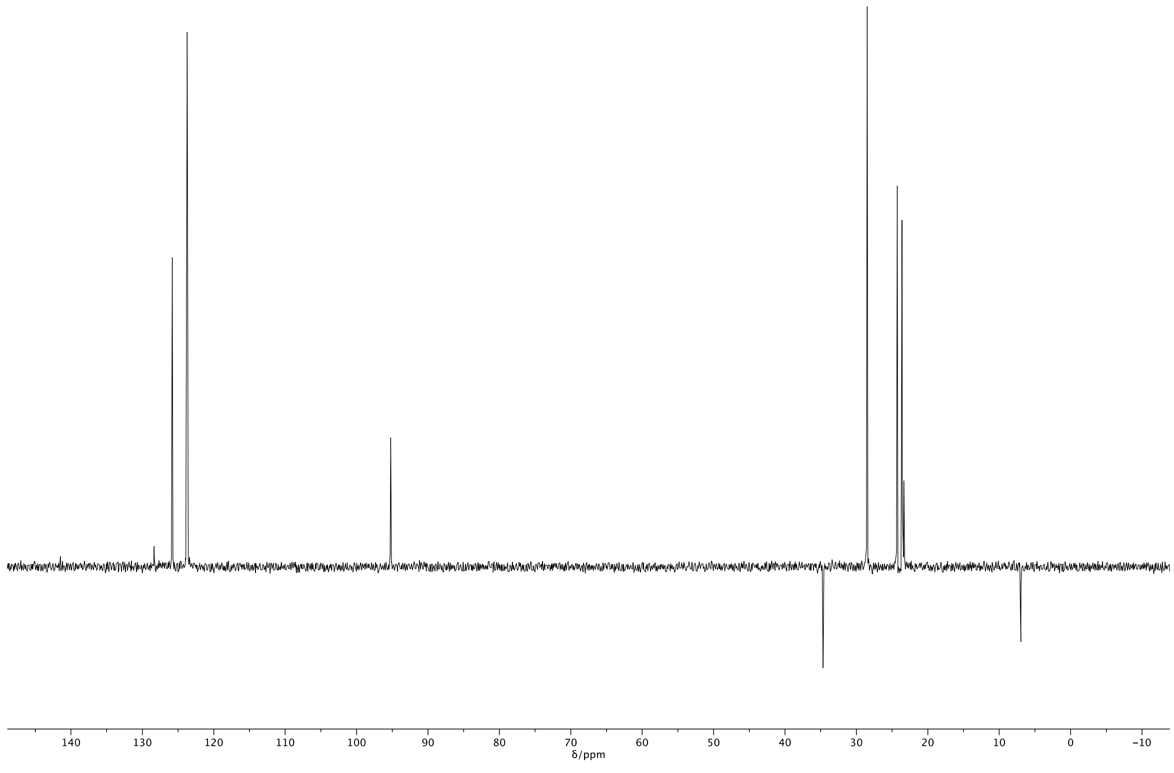

Figure S50. $^{13}C\{^{1}H\}$ DEPT-135 NMR spectrum of **9a**

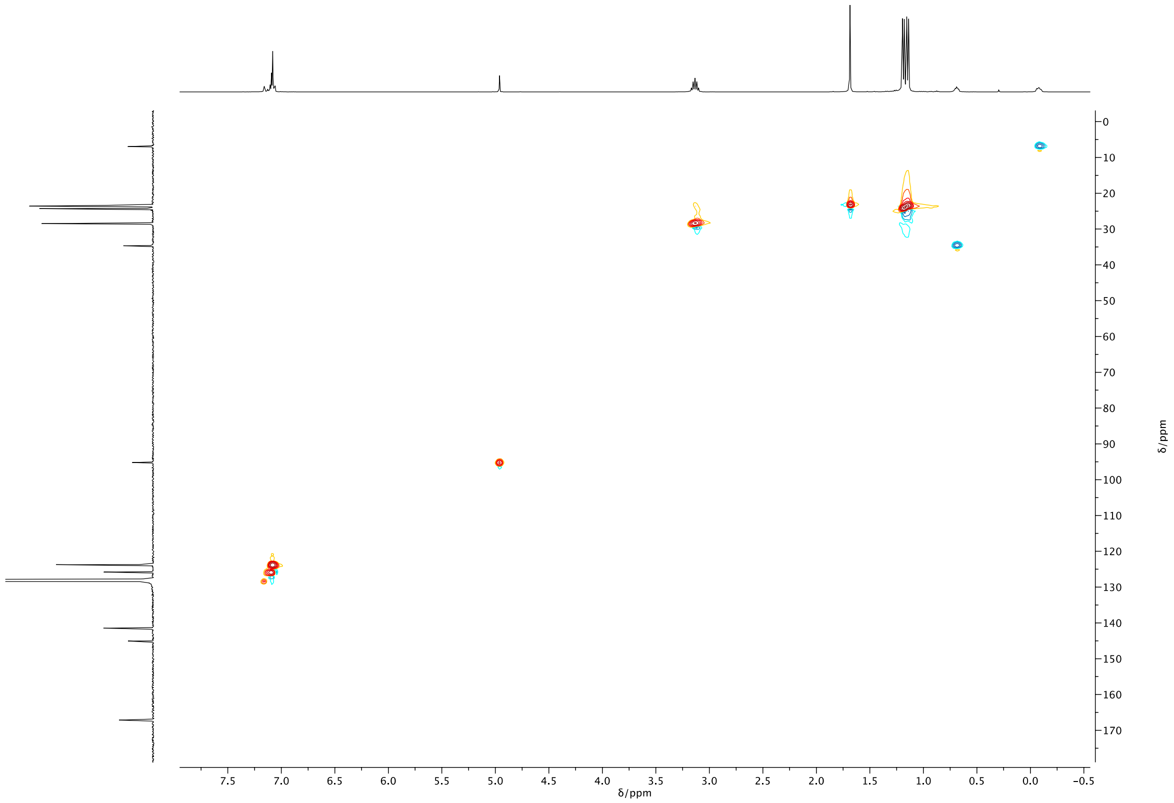

Figure S51. ^{1}H–^{13}C HSQC NMR spectrum of **9a**

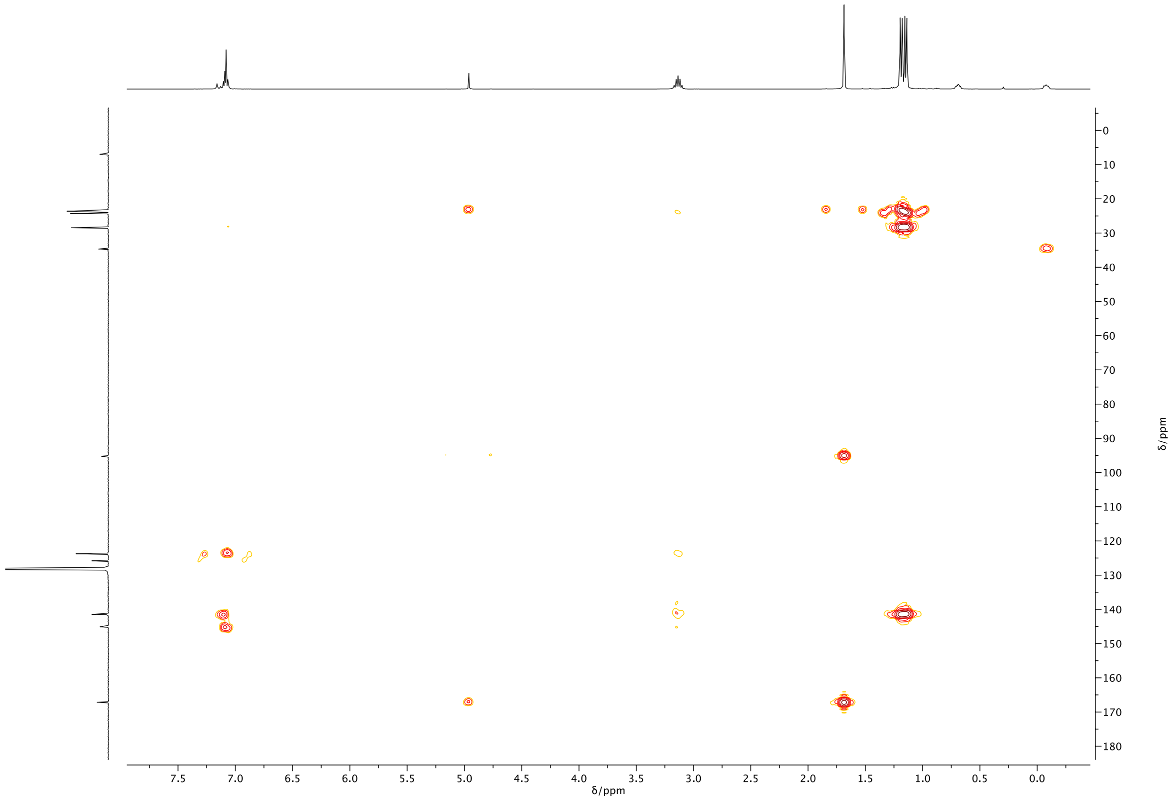

Figure S52. ^{1}H–^{13}C HMBC NMR spectrum of **9a**

Labelling reaction

A labelling study was carried out between the deuterated analogue of **1** with selective deuteration of the Mg–H group (**1-d**) and the most reactive alkylidene **3a** to determine the regioselectivity of the hydrometallation step. In a N_2 filled glovebox, a suspension of **1-d** (85% D-incorporated, 5.5 mg, 0.012 mmol) in C_6D_6 or C_6H_6 (0.6 mL) was added to a J-Young NMR tube. Methylidene cyclopropane (**3a**) was added in excess using a pre-cooled pipette and the NMR tube sealed. The resulting colourless solutions were monitored by ^1H (C_6D_6) and ^2H (C_6H_6) NMR spectroscopy over 24 h at room temperature. The ^2H NMR spectrum of the reaction mixture after 24 in C_6H_6 showed only one non-solvent resonance at 5.64 ppm (Figure S53), which corresponds perfectly to that observed for the internal alkene CH in the previously reported protio analogue.[3] The reaction in C_6D_6 was worked up as for other samples: the J-Young NMR tube was returned to the glovebox, the solution diluted with toluene (0.5 mL) and decanted into a scintillation vial. The volatiles were removed in *vacuo*, leaving an off-white solid. The solid was dissolved in n-pentane (1 mL) and filtered. The volatiles were removed in *vacuo*. ^1H NMR analysis of this product in C_6D_6 show that all the resonances for **6a** are observed with quantitative integrations, except the resonance for the internal alkene CH which is observed with *ca.* 15% intensity, due to a 15% protio impurity of **1** in **1-d** (Figure S54).

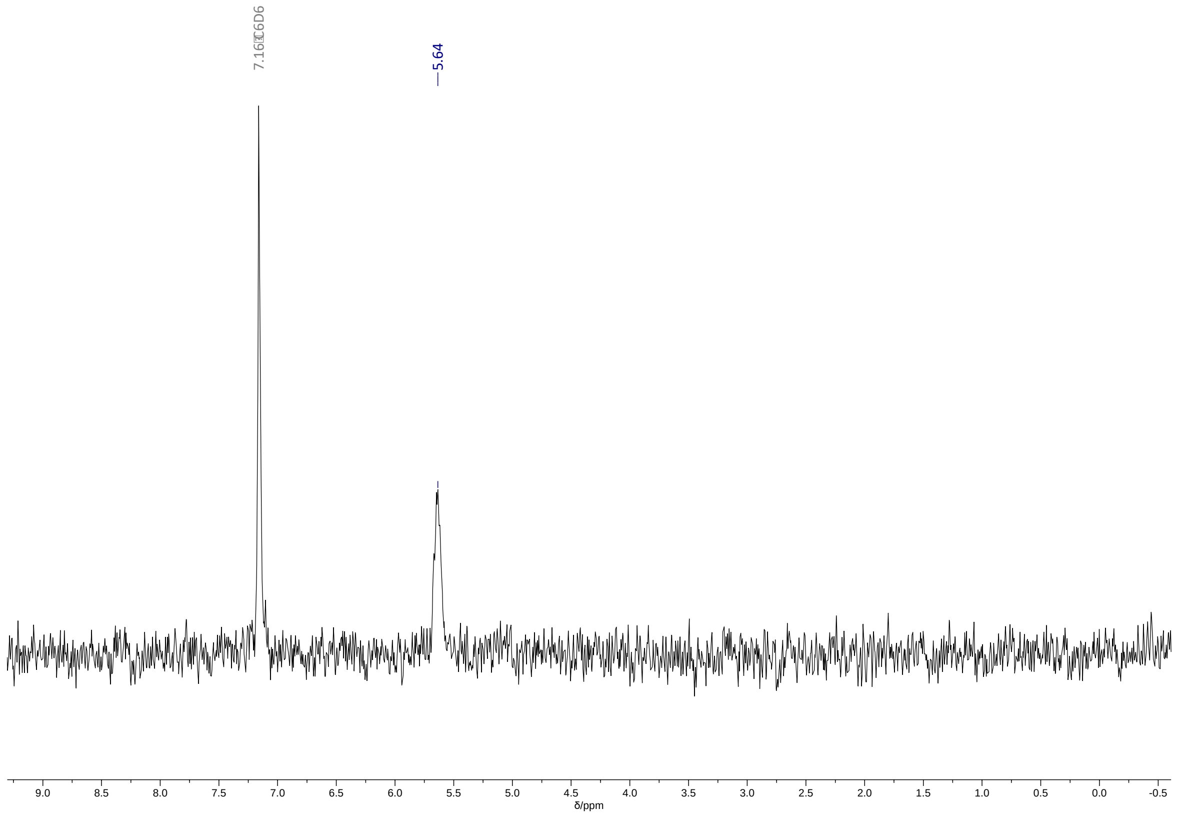

Figure S53. ^2H NMR spectrum of reaction between **1-d** and **3a** after 24 h at room temperature

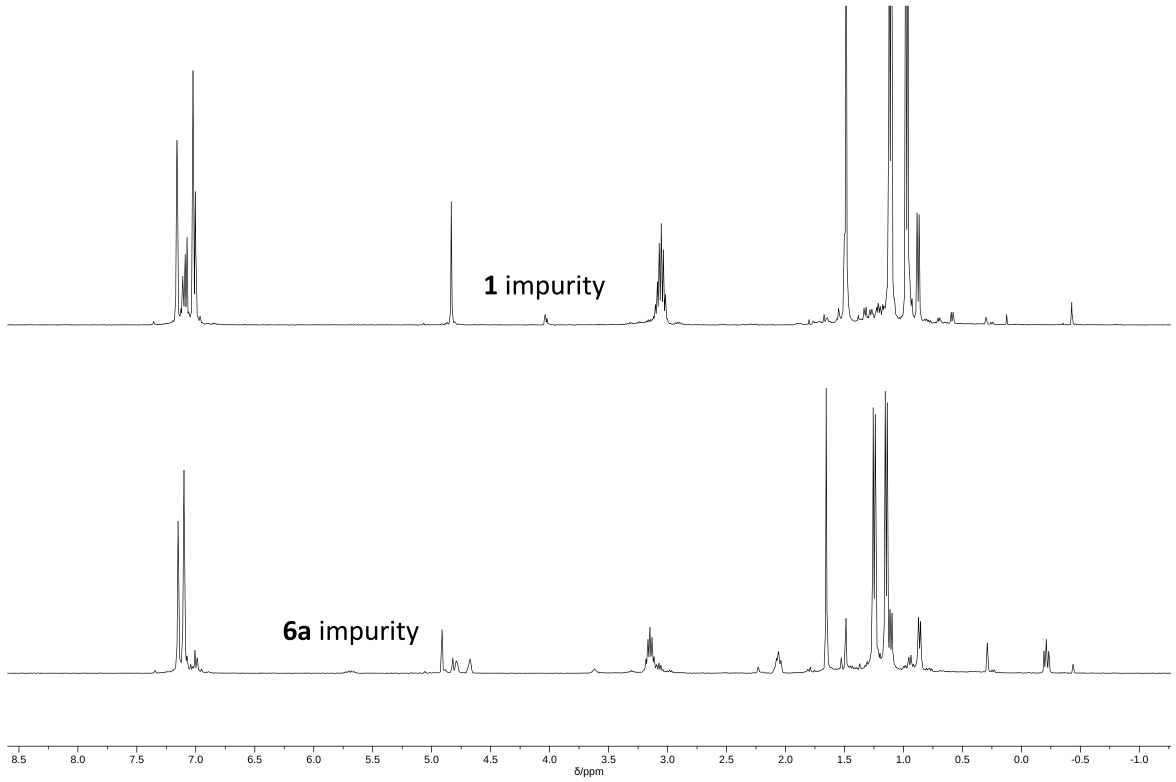

Figure S54. Stacked ^{1}H NMR spectra showing **1-d** with 15% **1** impurity (top) and isolated deuterated analogue of **6a** (bottom)

Catalytic hydrosilylation with magnesium

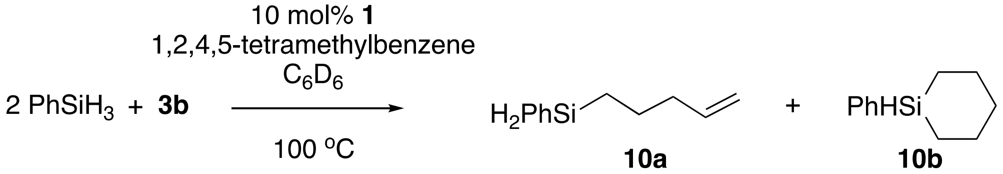

Catalytic hydrosilylation of 3b: In a glovebox, **1** (20 mg, 0.045 mmol), **3b** (30.7 mg, 41.6 μL, 0.45 mmol), and $PhSiH_3$ (111 μL, 0.90 mmol) were dissolved in C_6D_6 (0.6 mL) and transferred to a J-Young NMR tube. 1,2,4,5-tetramethylbenzene (6.0 mg, 0.045 mmol) was added as an internal standard. The tube was sealed, removed from the glovebox, and heated to 100 °C in an oil bath. The reaction was monitored at regular intervals by ^{1}H NMR spectroscopy. Upon complete consumption of **3b**, the tube was returned to the glovebox and the volatiles removed *in vacuo*. The resulting off-white oil was dissolved in n-pentane (3 x 0.5 mL) and filtered, removing the catalyst **1**. The volatiles removed *in vacuo*. The yellow oil was removed from the glovebox and subjected to NMR and TLC analysis, showing a mixture of desired product 1-phenyl-1-silacyclohexane (**10b**) and unreacted phenyl silane. **10b** was readily isolated by column chromatography (silica gel, hexanes, Rf = 0.4) and NMR spectra in $CDCl_3$ matches literature values (65.8 mg, 0.037 mmol, 83 %).[5,6]

Time (h)	**3b** (%)	**10a** (%)	**10b** (%)
1	94	5	1
3	74	12	7
4	70	14	12
5.5	63	16	20
7	58	15	27
10	46	13	39
13.5	36	8	49
17	27	7	56
19	23	6	66
21	21	4	69
25	18	4	74
28	16	2	80
33	9	1	84
36	8	1	89
42	5	1	89
50	0	<1	95

Table S1. Catalytic hydrosilylation of **3b** using **1** at 100 °C. Percentage yield calculated by integration of ^{1}H NMR spectra against internal standard.

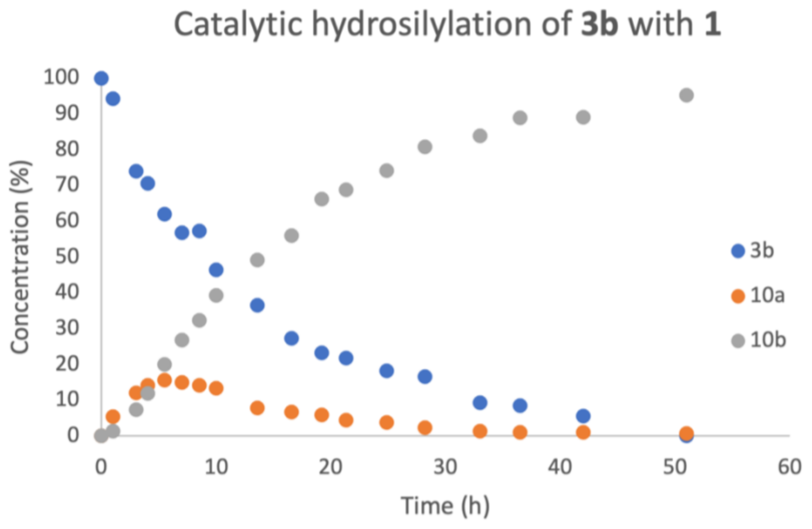

Figure S55. Catalytic hydrosilylation of **3b** using **1** at 100 °C. Percentage yield calculated by integration of ^{1}H NMR spectra against internal standard.

$$2\ PhSiH_3 + \mathbf{3a\text{-}d} \xrightarrow[100\ ^{o}C]{C_6D_6} \text{No reaction}$$

Control reactions of 3a-d with phenylsilane: In a glovebox, **3a-d** (0.04 mmol) and $PhSiH_3$ (5 μL, 0.04 mol) were dissolved in C_6D_6 (0.600 mL) and transferred to a J-Young NMR tube. The tube was sealed and removed from the glovebox. An initial ^1H NMR spectrum was taken at this timepoint. The reaction mixture was heated at 100 °C in an oil bath for 24 h and a second ^1H NMR spectrum was taken at this point. No reaction between **3a-d** and $PhSiH_3$ was observed.

Hydrosilylation with zinc

In a glovebox, **2** (21.8 mg, 0.045 mmol) and 1,2,4,5-tetramethylbenzene (6.0 mg, 0.045 mmol) were dissolved in C_6D_6 (0.6 mL) in a J-Young NMR tube and shaken to ensure complete dissolution. $PhSiH_3$ (111 μL, 0.90 mmol) was added and the solution was frozen at -35 °C. **3a** was added cold (-35 °C) to the still frozen solution in the cold tube using a pre-cooled pipette. Although 30.4 μL was attempted to be added, the extremely high volatility of **3a** makes this challenging. ^1H NMR spectra of the reaction mixture can be used to quantify the ratio of **3a** : $PhSiH_3$: **2**. **3a** was added until this ratio was found to be 1 : 1.42 : 0.10. This means the reaction mixture contained 1 equiv. **3a**, 1.4 equiv. $PhSiH_3$ and 10.5 mol % **2**. The mixture was subsequently heated to 100 °C for a total of 48 h and monitored by ^1H NMR spectroscopy:

- An initial rapid drop in **3a** (31% remaining after 1 h) and then a slow further decline (16% remaining after 48 h)
- An initial rapid drop in $PhSiH_3$ (54% remaining after 1 h) and then a slow further decline (32% remaining after 48 h)
- A sharp increase in linear silane product analogous to **10a** (8% after 1 h) which plateaus after *ca*. 3 h at 10% (NB. this is approximately 1 turnover as loading of **2** is 10.5–11.2 mol %), over 48 h this has increased to 14%.

The NMR resonances derived from the linear silane product were identified from previous reports.[3]

Time (h)	**3a** (%)	$PhSiH_3$ (%)	Linear silane (%)
0	100	100	0.7
1	31.0	53.7	8.2
2	34.3	48.8	9.6
3	31.4	46.4	10.2
5	29.9	43.7	10.9
8	23.2	39.4	12.3
12	23.6	38.8	12.3
16	24.7	37.8	13.0
20	22.5	35.2	13.6
25	21.8	35.2	13.6
30	17.3	34.1	13.6
48	16.2	32.1	14.3

Table S2. Hydrosilylation of **3a** using **2** at 100 °C. Percentage yield calculated by integration of ^{1}H NMR spectra against internal standard.

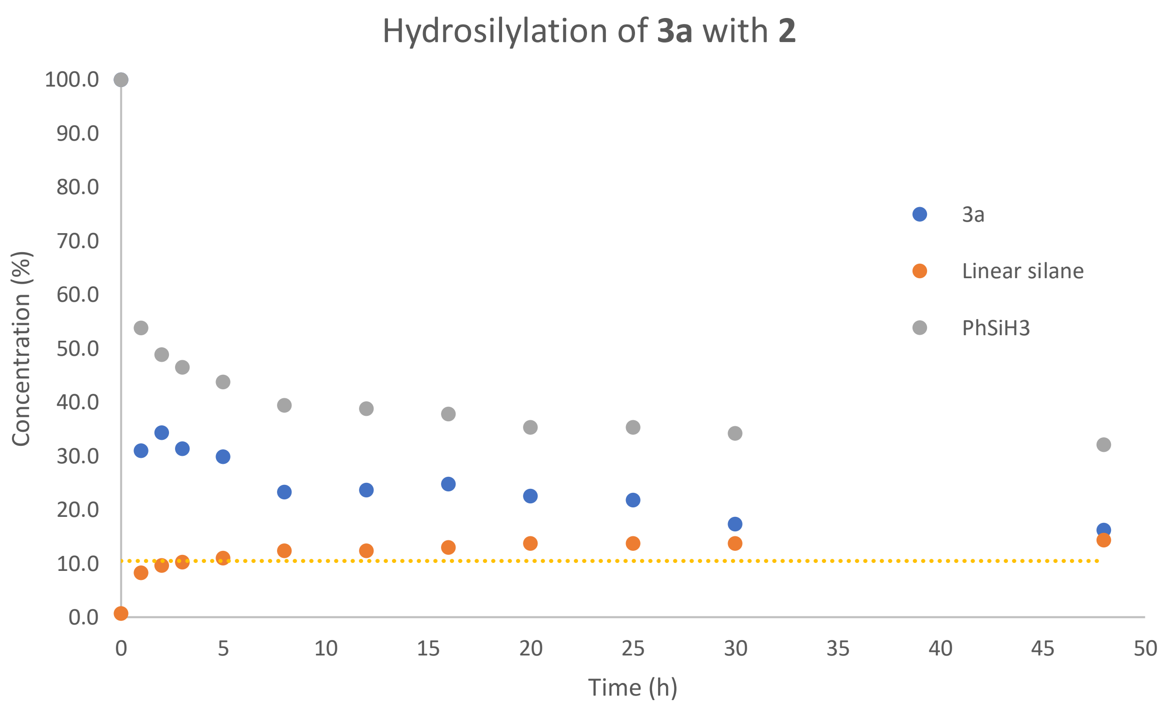

Figure S56. Hydrosilylation of **3a** using **2** at 100 °C. Percentage yield calculated by integration of ^{1}H NMR spectra against internal standard.

Kinetics

In a N_2 filled glovebox, **1** (40 mg, 0.090 mmol), **3c** (74.3 mg, 95.2 μL, 0.45 mmol), and 1,2,4,5-tetramethyl benzene (12.1 mg, 0.090 mmol; internal standard) were dissolved in C_6D_6 (3.0 mL) in a small vial. The solution was aliquoted into four J-Young tap NMR tubes (4 *x* 0.6 mL). The tubes were quickly sealed and stored in the glovebox freezer at -35 °C. The tubes were warmed to room temperature and immediately inserted into a pre-heated spectrometer (70 – 100 °C). The reaction was monitored by ^{1}H NMR spectroscopy and known resonances integrated against the internal standard. Four rate constants were determined in the temperature range of 70 to 100 °C and a plot of $\ln(k_{obs})$ against 1/T allowed calculation of the thermodynamic parameters using the Eyring equation. Data was plotted to 2 half-lives. Standard errors were calculated using regression analysis in the Microsoft Excel program. $\Delta H^{\ddagger}$ = 15.0 ±2.0 kcal mol^{-1}, $\Delta S^{\ddagger}$ = – 34.4 ± 14.7 cal K^{-1} mol^{-1} and an associated $\Delta G^{\ddagger}$(298K) = 25.2 ±2.0 kcal mol^{-1}.

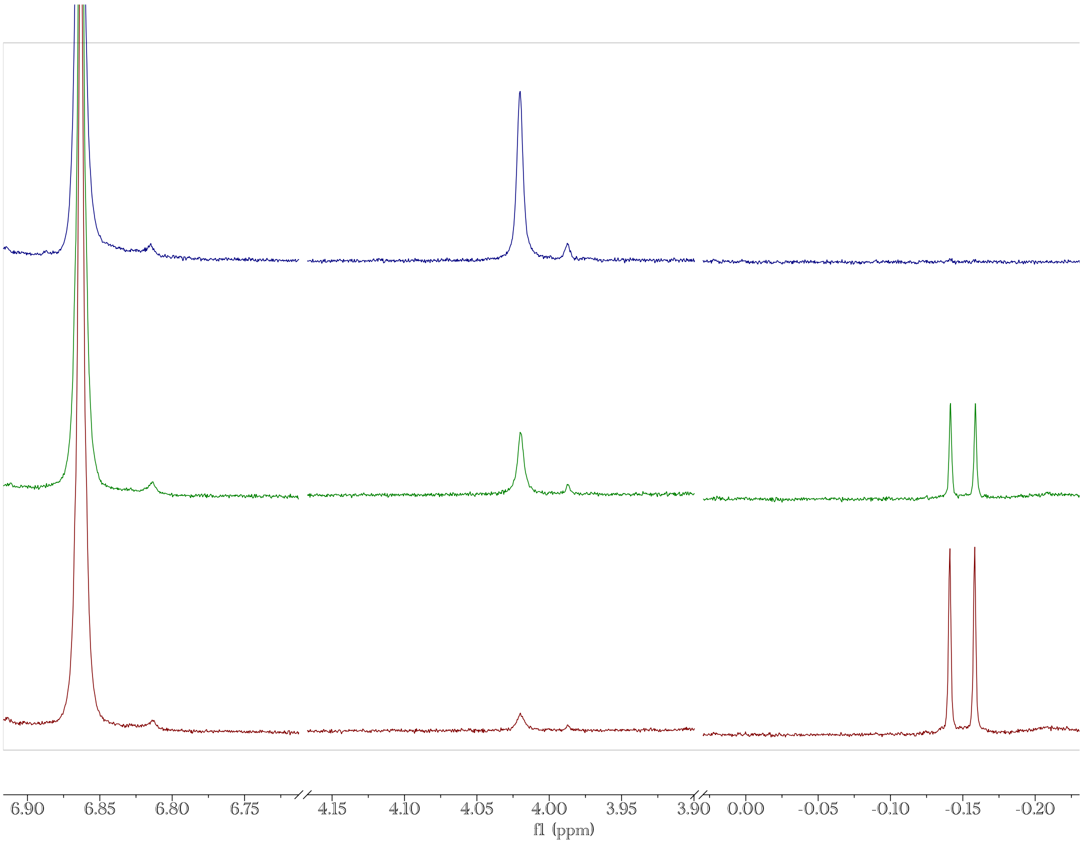

Figure S57. Representative example of ^{1}H NMR spectra for kinetic experiment. Showing t = 0 (top, blue), t = end (bottom, red) and t = intermediate timepoint (middle, green).

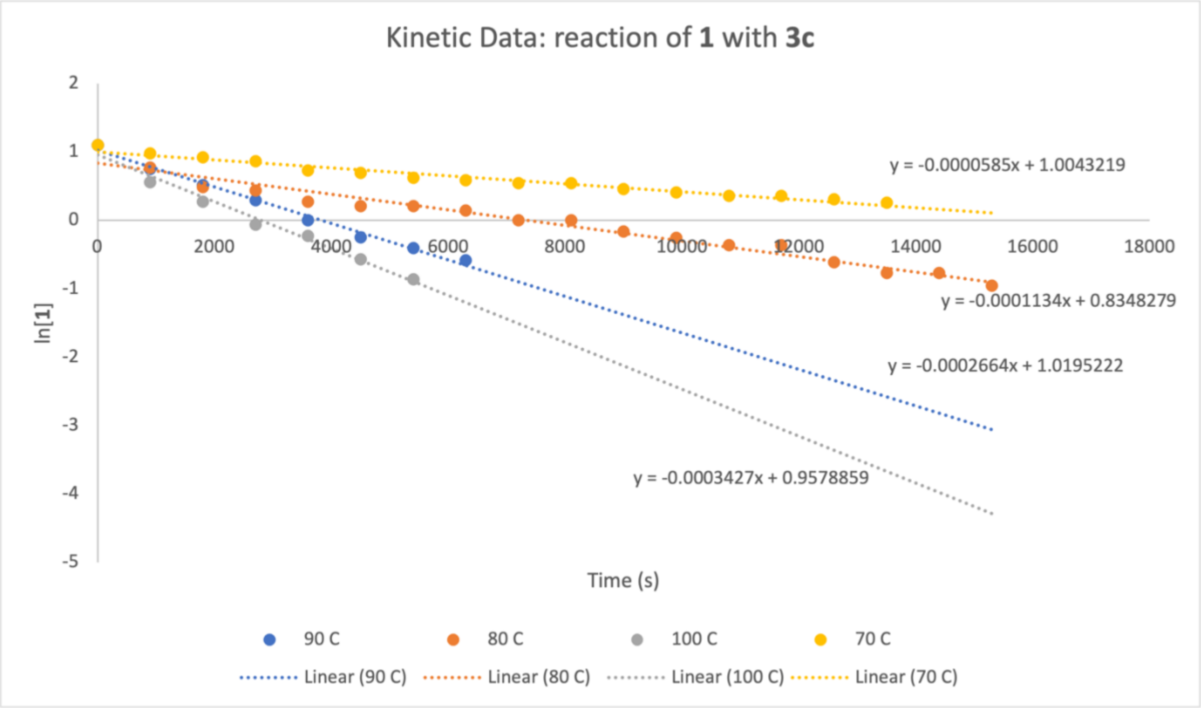

Figure S58. Kinetic data for the transformation of **1** to **4c** at 70 – 100 °C.

Temperature (K)	1/T	k (s^{-1})	ln(k/T)
343	0.00291	0.0000585	-15.585
353	0.00283	0.000113	-14.952
363	0.00275	0.000266	-14.125
373	0.00270	0.000343	-13.901

Table S3. Table of rate constants derived from kinetic experiments

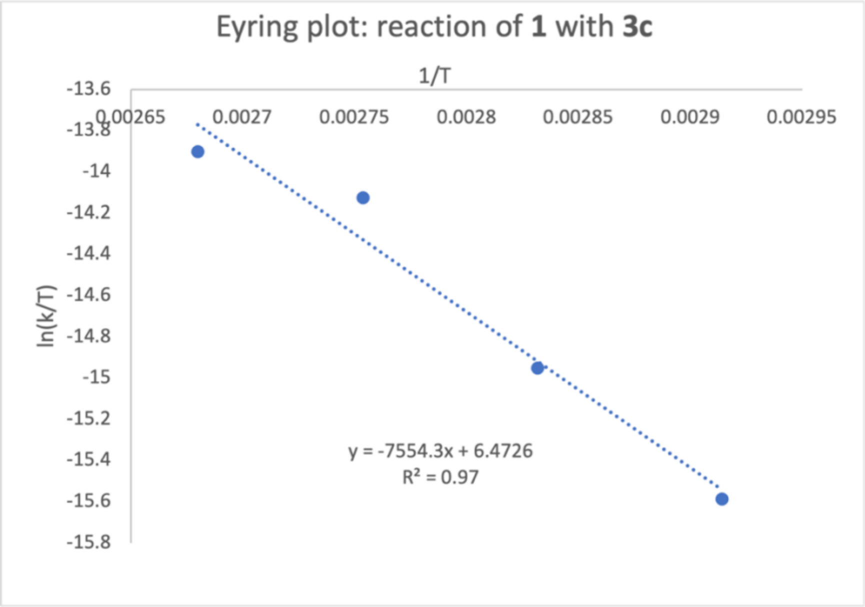

Figure S59. Eyring plot of data from Table S2.

3. X-ray Crystallography

Table S4 provides a summary of the crystallographic data for the structures of **5b**, **5c**, **7a** and **9a**. Data were collected using an Agilent Xcalibur PX Ultra A diffractometer, and the structures were solved and refined using the OLEX2,[7] SHELXTL[8] and SHELX-2013[9] program systems. CCDC 2211702–2211705.

**data**	**5b**	**5c**	**7a**	**9a**
**formula**	$C_{34}H_{50}N_2Zn$	$C_{35}H_{52}N_2Zn$	$C_{33}H_{48}N_2Zn$	$C_{62}H_{90}N_4Zn_2$
**solvent**	–	–	–	–
**formula weight**	552.13	566.15	538.10	1022.11
**colour, habit**	colourless blocks	colourless block	colourless blocks	colourless blocks
**temperature / K**	173	173	173	173
**crystal system**	orthorhombic	monoclinic	monoclinic	triclinic
**space group**	Pbca	$P12_1/n1$	$P12_1/n1$	P-1
***a* / Å**	15.0449(2)	11.2164(3)	10.7471(7)	12.4022(4)
***b* / Å**	16.2449(2)	20.8079(5)	21.2654(8)	15.9790(4)
***c* / Å**	26.5593(3)	14.1171(4)	13.8275(7)	31.6956(7)
**α / deg**	90	90	90	83.997(2)
**β / deg**	90	96.264(2)	93.143(4)	93.924(2)
**γ / deg**	90	90	90	68.783(2)
**V / Å^3**	6491.17(14)	3275.11(15)	3155.4(3)	5807.7(3)
**Z**	8	4	4	4
**D_c / g cm^{-3}**	1.130	1.148	1.133	1.169
**radiation used**	Cu-Kα	Cu-Kα	Cu-Kα	Cu-Kα
**μ / mm^{-1}**	1.199	1.199	0.800	1.302
**no. of unique reflns**				
**measured (R_{int})**	6480 (0.0319)	6283 (0.0309)	6567 (0.0366)	22157 (0.0281)
**obs, $\|F_o\| > 4\sigma(\|F_o\|)$**	5624	4761	4907	17283
**completeness (%)** [a]	100	98.6	99.7	98.4
**no. of variables**	344	374	347	1284
**R_1(obs), wR_2(all)** [b]	0.0352, 0.1009	0.0791, 0.2418	0.0457, 0.1399	0.0426, 0.1232
**CCDC code**	2211702	2211703	2211704	2211705

[a] Completeness to 0.84 Å resolution. [b] $R_1 = \Sigma||F_o| - |F_c||/\Sigma|F_o|$; $wR_2 = \{\Sigma[w(F_o^2 - F_c^2)^2] / \Sigma[w(F_o^2)^2]\}^{1/2}$; $w^{-1} = \sigma^2(F_o^2) + (aP)^2 + bP$.

Table S4. Crystal data, data collection and refinement parameters for the structures of **5b**, **5c**, **7a** and **9a**.

Crystal structure of 5b

Compound **5b** was found to crystallise in the orthorhombic space group Pbca.

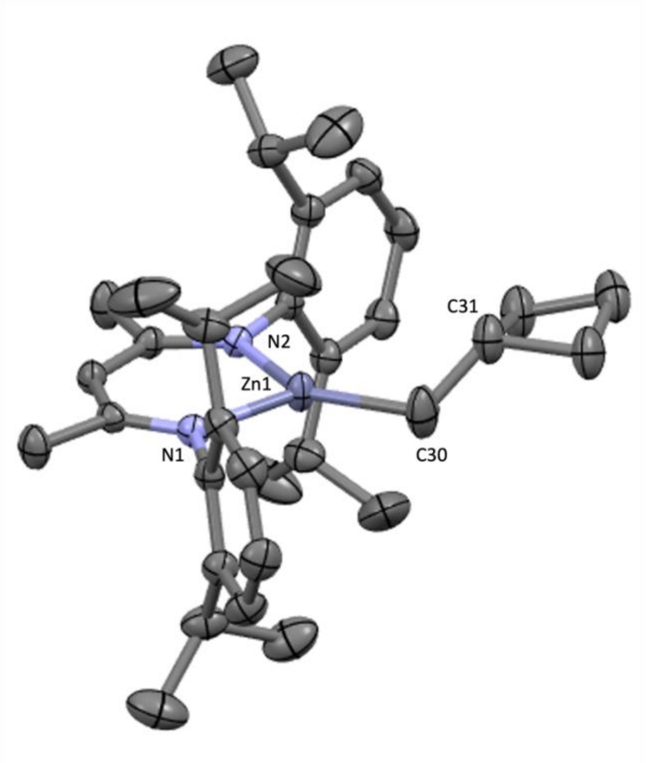

Figure S60. Structure of **5b**. Hydrogens removed for clarity. Thermal ellipsoids drawn at 50% probability.

Crystal structure of 5c

In the crystal structure of **5c** there is a large unassigned Q-peak (3.400 electrons) which sits in a chemically nonsensical position directly above C1 (0.759(4) Å). We were unable to satisfactorily assign or model this electron density. We investigated the following explanations in attempts to explain the artefact:

- *A result of unresolved twinning leading to a second crystal orientation*. Re-analysis of diffraction peaks reliably returns a peak indexing value of >98%. Even if a genuine <2% twin was present in the crystal sample, this would not manifest as such a large Q-peak.
- *Disorder in the phenyl ring containing C1*. There is no obvious disorder in the phenyl ring that would account for the unassigned electron density. If the ring were disordered over two positions with *ca.* 50:50 occupancy, an entire set of similar intensity Q-peaks should be observable to account for this alternative position. Within the 2,6-di-*iso*-propylphenyl group that contains C1 (as the *ipso*-carbon), disorder is observed in one *iso*-propyl group. In this case the CH_3 groups were both modelled anisotropically over two positions (58:42).
- *An entire secondary minor molecular orientation for **5c** that sits within the same unit cell*. In this case it is presumed that the unassigned electron density would be from the heaviest

element in this hypothetical second orientation (Zn). Assigning the Q-peak as Zn with a low (5%) occupancy, and subsequent analysis of the remaining Q-peaks should allow complete or partial identification of this second orientation *e.g.* the coordinated N atoms, adjacent phenyl rings *etc.* Unfortunately, no such clear assignment could be made, and hence the electron density remains unassigned.

Despite the unassigned electron density, the overall structure has a reasonable R_1-factor of 7.91%. Nevertheless, no detailed analysis of bond lengths or angles was undertaken due to the unreliability of these values derived from the crystal structure alone. Still, the values that are obtained for key metrics *e.g.* Zn–C and Zn–N distances all fall within reported ranges for similar reported structures.

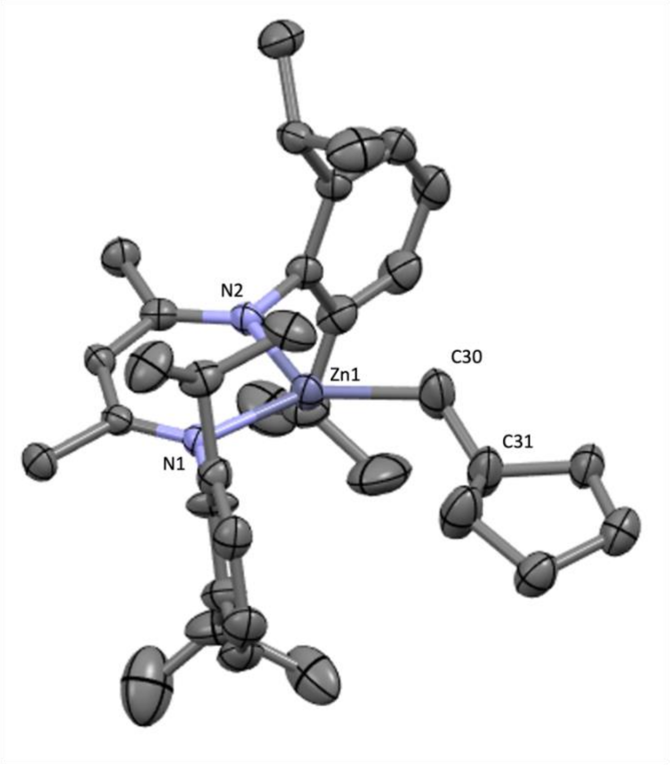

Figure S61. Structure of **5c**. Hydrogens and minor component (*iso*-propyl group) removed for clarity. Thermal ellipsoids drawn at 50% probability.

Crystal structure of 7a

The butene chain (C30–C33) was disordered and modelled over two crossing positions at slightly different orientations from the N1–Zn1–N2 plane (18.4(4) ° and 35.0(11) °). The minor component was fixed at an occupancy of 20% and refined isotropically. The major component (80%) was refined anisotropically. SIMU and SADI restraints were used over the entire length of the minor component in order to mimic the major component. An additional DFIX was used for the C32B=C33B double bond. The final major component was found to be $d_{(C32A–C33A)}$ = 1.157(9) Å.

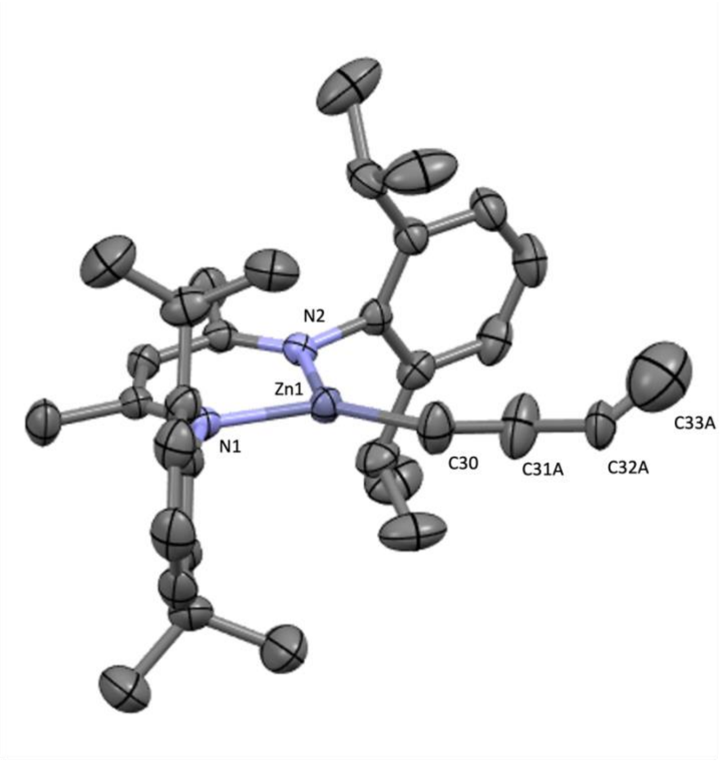

Figure S62. Structure of **7a**. Hydrogens and minor component (butene group) removed for clarity. Thermal ellipsoids drawn at 50% probability.

Crystal structure of 9a

The crystal structure of **9a** was found to crystallise with two independent molecules. The bridging butyl chain of one independent molecule was found to be disordered and was modelled over two crossing orientations (*ca.* 57:43). The geometries of each orientation were optimised, the thermal parameters of adjacent atoms were restrained to be similar, and both orientations were refined anisotropically.

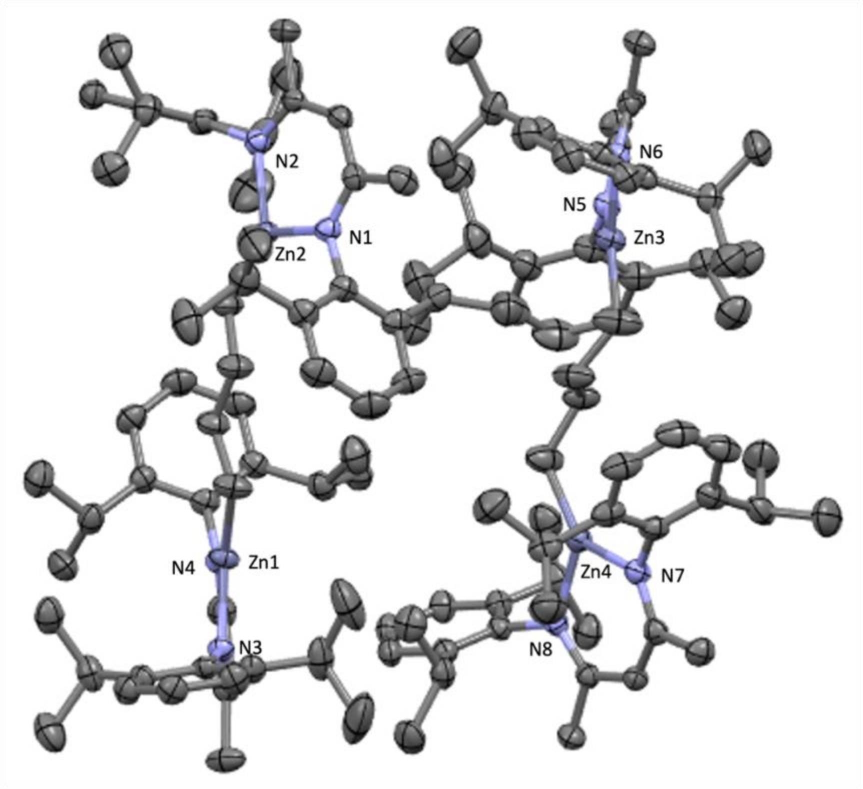

Figure S63. Structure of **9a**. Hydrogens and minor component (bridging butyl group) removed for clarity. Thermal ellipsoids drawn at 50% probability.

4. Computational Calculations

Density Functional Theory (DFT) calculations were run using Gaussian 09 (Revision D.01)[10] using the wB97X-D[11] functional and an ultrafine integrations grid (keyword int=ultrafine). Metal atoms (Mg, Zn) were described with Stuttgart SDDAll RECPs and associated basis sets, while a hybrid basis set was used for the other atoms: 6-31g**(C, H)/ 6-311+g*(N, O).[12–14] The level of theory used has previously been benchmarked in our group and shown to accurately reproduce the experimental results.[15–18]

Geometry optimisations were performed without symmetry constraints. Frequency analyses for all stationary points were performed to confirm their nature of the structures as either minima (no imaginary frequency) or transition states (only one imaginary frequency). Single point solvent corrections (benzene, epsilon = 2.2706) were applied using the polarized continuum model (PCM) to free energies.[19] Intrinsic Reaction Coordinate (IRC) calculations followed by full geometry optimisations on final points were used to connect transition states and minima located on the Potential Energy Surface.[20,21] A full energy profile was constructed (calculated at 298.15 K, 1 atm). The graphical user interface used to visualise the various properties of compounds **1-9** was GaussView 5.0.9.[22]

Functional testing was carried out and calculated bond lengths and angles compared to the single crystal X-ray data for compound **7a** (Table S5). Complete potential energy surfaces for both the zinc and magnesium system we report were calculated using the PBE0 functional,[23–25] with additional empirical dispersion=gd3bj[26] and solvent correction as reported above (Figure S13-16). Calculated data closely matched that of the wB97X-D functional.

Natural Bond Orbital analysis was carried out in NBO 6.0.[27,28] A full NBO analysis for all stationary points was carried out and the relevant NPA charges and Wiberg Bond Indices tabulated (Table S7-8). QTAIM calculations were performed using the AIMAll sotware.[29,30] Activation strain analysis were performed using input files from the AutoDIAS python tool.[31] Non-covalent interactions were analysed using the NCIPLOT 3.0 program.[32]

Computed structures, energies and analysis

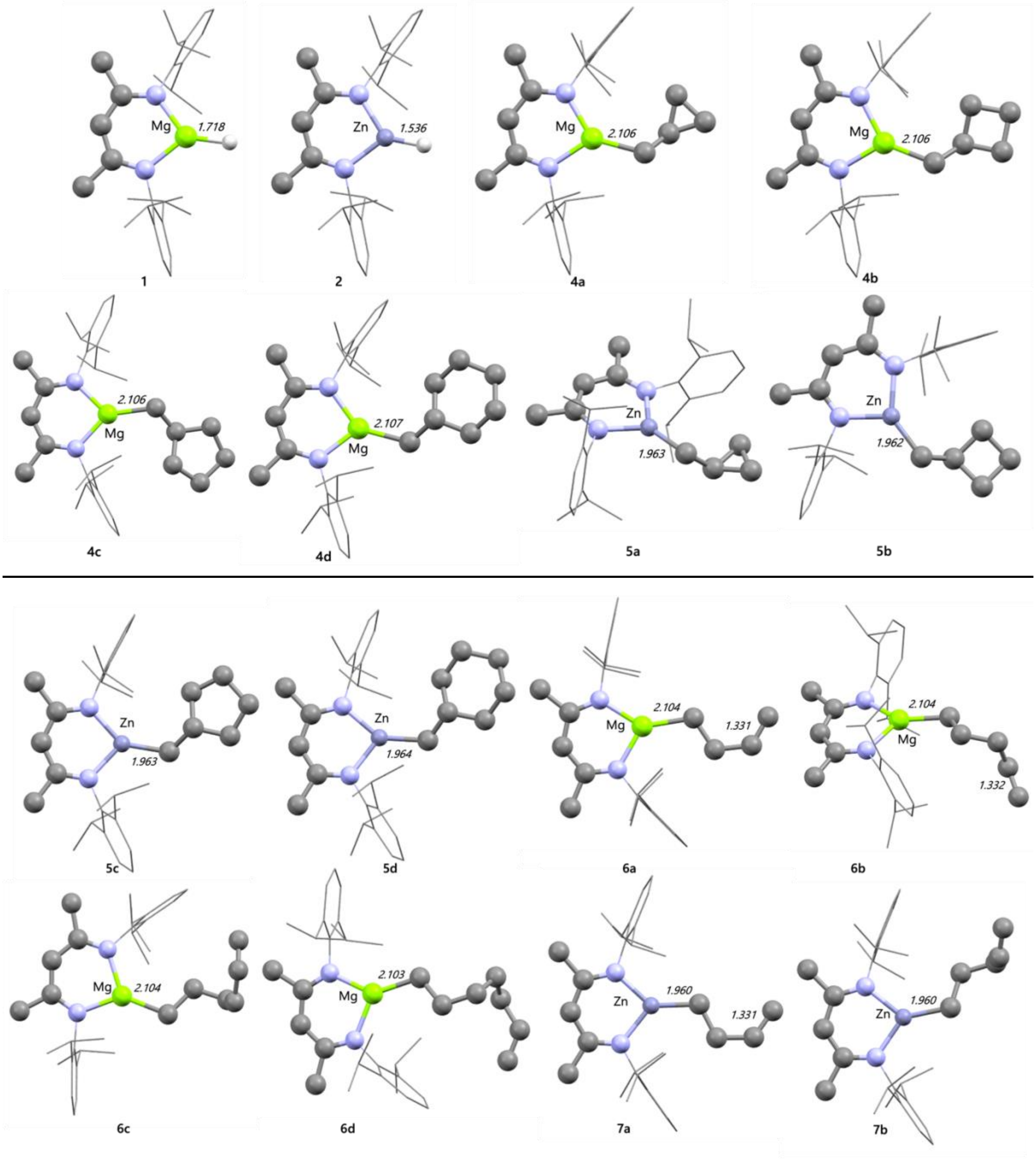

Figure S64. Structures of all stationary point minima as calculated by DFT. Select bond lengths annotated (Å), most hydrogen atoms omitted and diisopropyl units shown in wireframe for clarity.

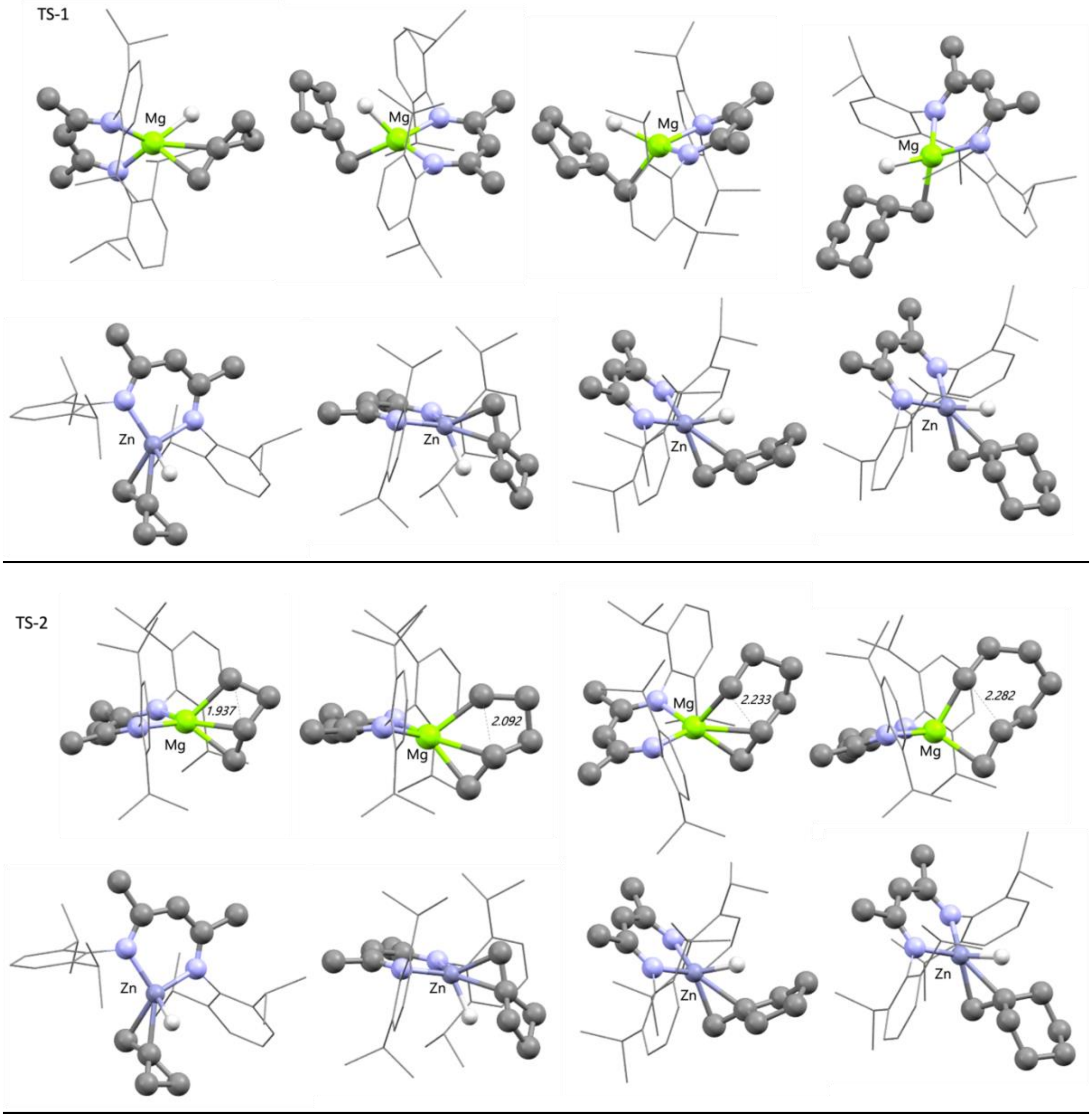

Figure S65. Structures of all transition states as calculated by DFT. Select bond lengths annotated (Å), most hydrogen atoms omitted and diisopropyl units shown in wireframe for clarity.

7a	xrd	ωB97XD	B3LYP	M06-L	M06-2X	PBE0
**C–Zn bond length (Å)**	1.960(3)	1.960	1.968	1.965	2.007	1.961
**C=C bond length (Å)**	1.157(9)	1.323	1.326	1.326	1.325	1.325
**N–Zn bond length (Å)**	1.956(2), 1.964(2)	1.964, 1.997	1.975, 2.006	1.984, 2.022	2.000, 2.020	1.967, 2.000
**N–Zn–C angle (°)**	131.84(10), 132.26(10)	123.7, 142.2	124.0, 141.7	122.2, 144.7	125.3, 141.7	124.0, 141.7
**N–Zn–N angle (°)**	95.83(9)	94.0	94.1	93.1	93.0	94.2

Table S5. Functional testing for comparison with single crystal X-ray data for compound **7a**.

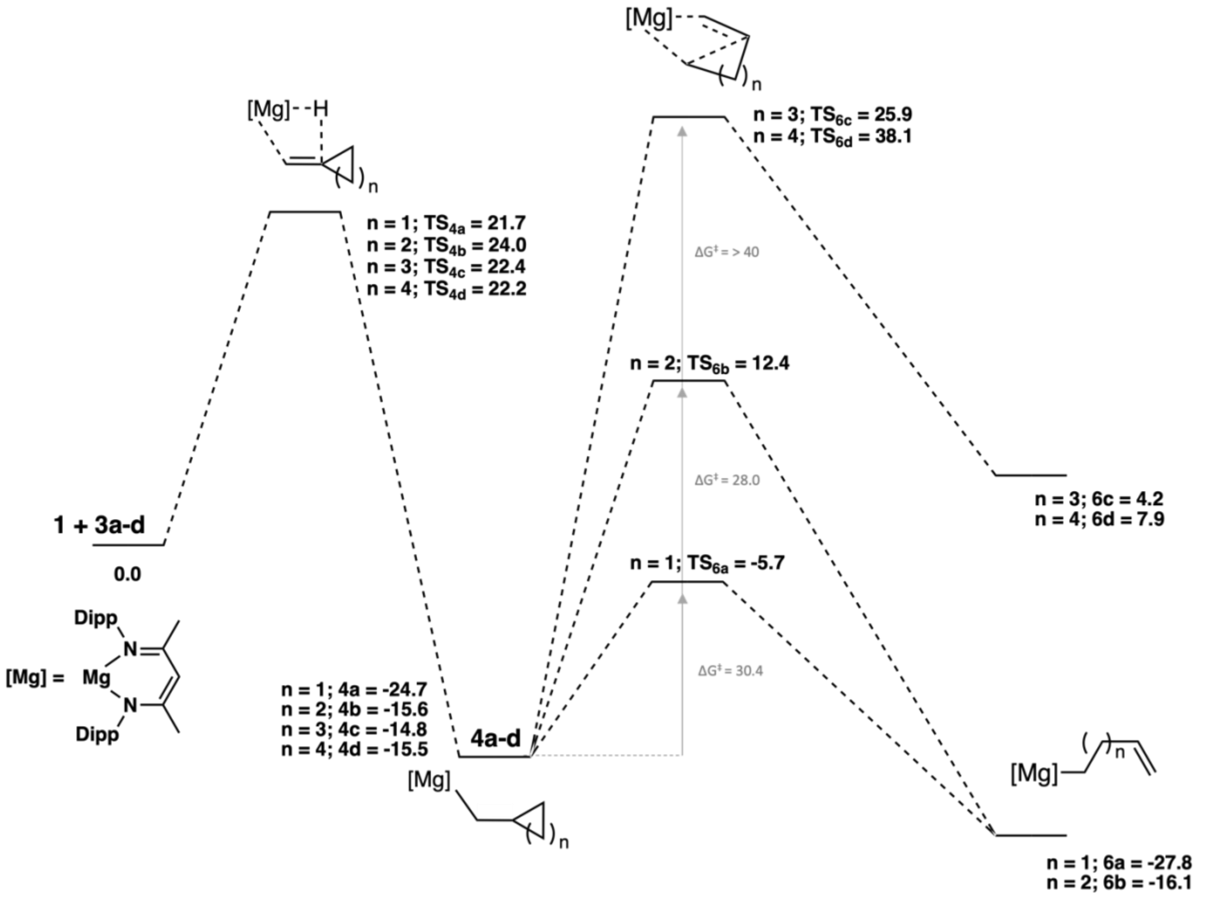

Figure S66. Potential energy surface for **1** with **3a-d** using the wB97X-D functional. Metal atoms (Mg, Zn) were described with Stuttgart SDDAll RECPs and associated basis sets, while a hybrid basis set was used for the other atoms: 6-31g**(C, H)/ 6-311+g*(N, O).

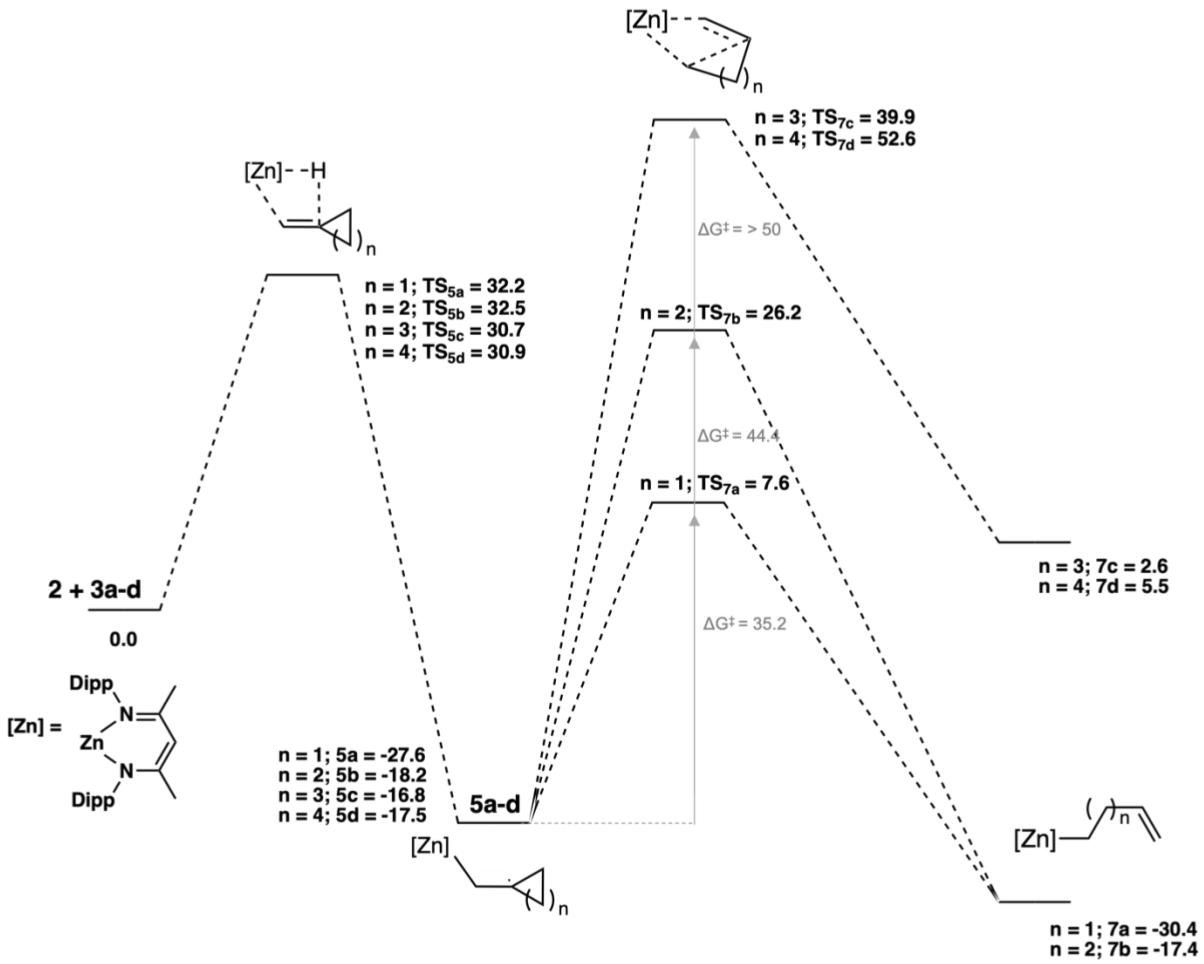

Figure S67. Potential energy surface for **2** with **3a-d** using the wB97X-D functional. Metal atoms (Mg, Zn) were described with Stuttgart SDDAll RECPs and associated basis sets, while a hybrid basis set was used for the other atoms: 6-31g**(C, H)/ 6-311+g*(N, O).

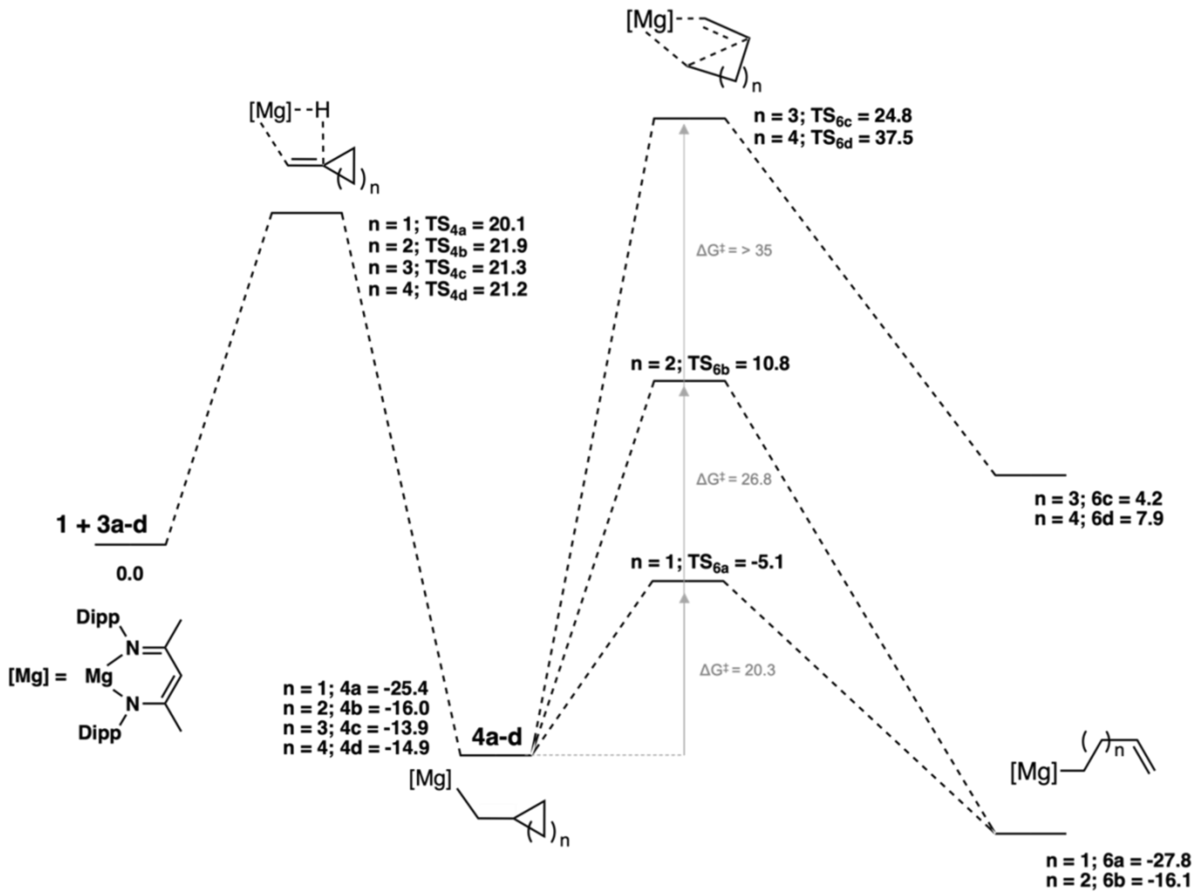

Figure S68. Potential energy surface for **1** with **3a-d** using the PBE0 functional and additional empirical dispersion (gd3bj). Metal atoms (Mg, Zn) were described with Stuttgart SDDAll RECPs and associated basis sets, while a hybrid basis set was used for the other atoms: 6-31g**(C, H)/ 6-311+g*(N, O).

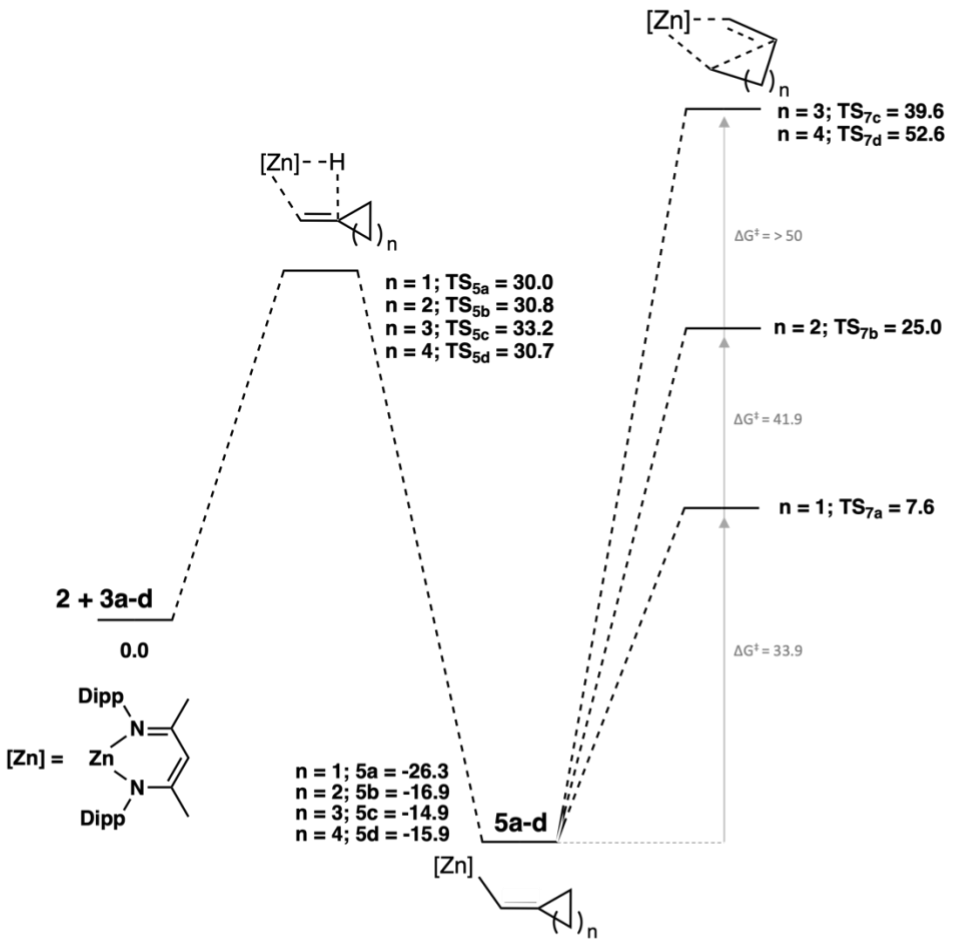

Figure S69. Potential energy surface for **2** with **3a-d** using the PBE0 functional and additional empirical dispersion (gd3bj). Metal atoms (Mg, Zn) were described with Stuttgart SDDAll RECPs and associated basis sets, while a hybrid basis set was used for the other atoms: 6-31g**(C, H)/ 6-311+g*(N, O).

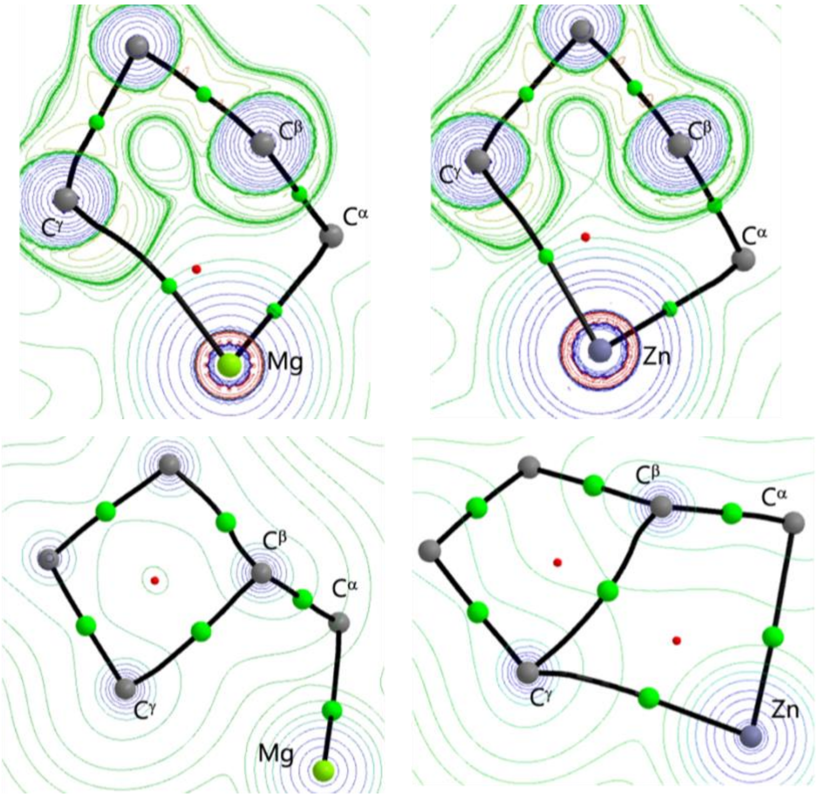

Figure S70. QTAIM contour plot of ρ for key C–C breaking transition states. Atomic charges and electron density at the bond critical point (ρ) are tabulated below. Bond (green) and ring (red) critical points are shown as spheres.

	QTAIM Charges				Bond ρ		
	M	C^{α}	C^{β}	C^{γ}	M–C^{α}	M–C^{γ}	C^{β}–C^{γ}
**4a**	1.63	-0.55	0.035	0.028	0.055	-	0.24
**TS-2(Mg, n=1)**	1.67	-0.40	-0.24	-0.32	0.062	0.047	-
**4b**	1.63	-0.058	0.092	0.035	0.055	-	0.24
**TS-2 (Mg, n=2)**	1.69	-0.40	-0.16	-0.27	0.059	-	0.135
**5a**	1.03	-0.29	0.038	-0.015	0.109	-	0.243
**TS-1 (Zn, n=1)**	1.16	-0.29	-0.17	-0.23	0.098	0.077	-
**5b**	1.03	-0.33	0.094	-0.001	0.109	-	0.240
**TS-2 (Zn, n=2)**	1.16	-0.28	0.00	-0.29	0.066	0.052	0.077

Table S6. QTAIM data for select calculated intermediates and transition states. Atomic charges and electron density at the bond critical point (ρ).

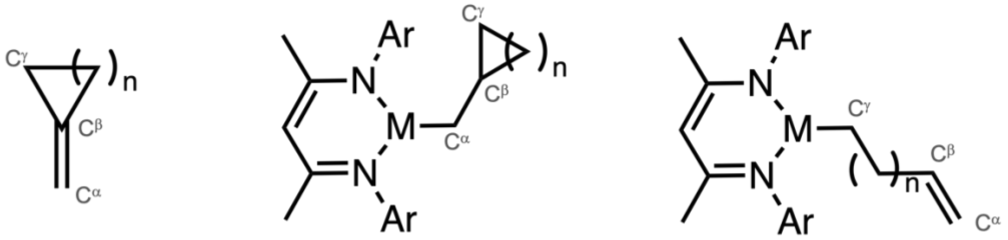

Figure S71. Schematic showing nomenclature for dft analysis.

	NPA Charges				**Wiberg Bond Indices**				Bond length (Å, dft)			
	Mg	C^{α}	C^{β}	C^{γ}	Mg–C^{α}	Mg–C^{γ}	C^{β}–C^{γ}	C^{α}–C^{β}	Mg–C^{α}	Mg–C^{γ}	C^{β}–C^{γ}	C^{α}–C^{β}
**1**	1.54	-	-	-	-	-	-	-	-	-	-	-
**TS-1 (n=1)**	1.77	-0.86	-0.08	-0.47	0.026	0.00	0.99	1.54	2.293	3.554	1.480	1.377
**4a**	1.69	-1.18	-0.27	-0.49	0.30	0.00	0.98	1.04	2.106	3.678	1.506	1.520
**TS-2 (n=2)**	1.78	-0.93	0.00	-0.47	0.045	0.00	1.01	1.50	2.261	3.384	1.528	1.390
**4b**	1.69	-1.18	-0.26	-0.47	0.31	0.01	1.04	0.98	2.106	3.647	1.550	1.522
**TS-2 (n=1)**	1.79	-1.01	-0.18	-0.94	0.09	0.043	0.53	1.36	2.218	2.263	1.937	1.423
**6a**	1.69	-0.48	-0.21	-1.19	0.00	0.31	0.023	1.97	5.106	2.104	2.598	1.331
**TS-2 (n=2)**	1.80	-1.01	-0.22	-0.88	0.096	0.022	0.57	1.37	2.182	2.273	2.092	1.423
**6b**	1.70	-0.47	-0.21	-1.19	0.00	0.31	0.002	1.97	5.936	2.104	3.199	1.332

Table S7. Select Natural Population Analysis (NPA) Charges and Wiberg Bond Indices for complexes **4a-b**, **6a-b** and associated **TS**.

	NPA Charges				**Wiberg Bond Indices**				Bond length (Å, dft)			
	Zn	C^{α}	C^{β}	C^{γ}	Zn–C^{α}	Zn–C^{γ}	C^{β}–C^{γ}	C^{α}–C^{β}	Mg–C^{α}	Mg–C^{γ}	C^{β}–C^{γ}	C^{α}–C^{β}
**2**	1.34	-	-	-	-	-	-	-	-	-	-	-
**TS-1 (n=1)**	1.61	-0.89	-0.13	-0.47	0.003	0.007	0.99	1.42	2.156	3.336	1.482	1.396
**5a**	1.45	-1.08	-0.27	-0.48	0.50	0.003	0.98	1.03	1.963	3.635	1.503	1.517
**TS-2 (n=2)**	1.63	-1.01	-0.19	-0.89	0.18	0.092	0.55	1.28	2.089	2.213	1.925	1.440
**5b**	1.45	-1.08	-0.26	-0.46	0.50	0.012	0.98	1.03	1.962	3.580	1.548	1.521
**TS-2 (n=1)**	1.63	-1.01	-0.19	-0.88	0.18	0.17	0.55	1.28	2.089	2.213	1.925	1.440
**7a**	1.46	-0.48	-0.21	-1.10	0.00	0.51	0.02	1.97	4.945	1.960	2.580	1.331
**TS-2 (n=2)**	1.64	-0.99	-0.23	-0.83	0.21	0.058	0.58	1.29	2.049	2.267	2.094	1.440
**7b**	1.46	-0.47	-0.21	-1.10	0.00	0.50	0.002	1.98	5.858	1.960	3.170	1.331

Table S8. Select Natural Population Analysis (NPA) Charges and Wiberg Bond Indices for complexes **5a-b**, **7a-b** and associated **TS**.

NBO analysis of select TS-2

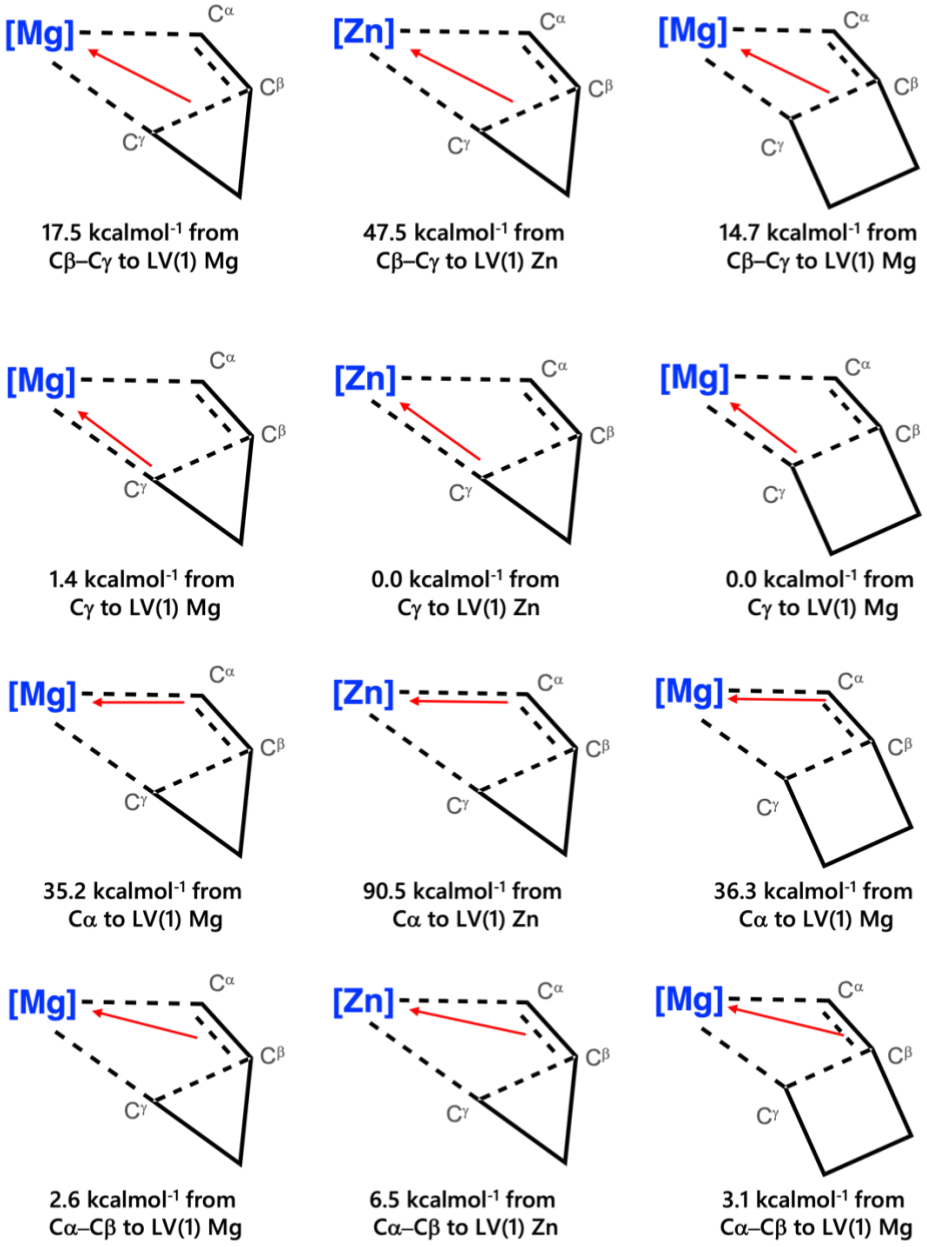

NBO analysis, specifically Second Order Perturbation, was used to give insight into the key C–C activation transition states. One stabilising interaction from the breaking C–C bond to the electropositive M (Mg, Zn) centre was found for **TS-2**. Stabilising donation of electron density from the Cb–Cg bond to LV(1), the s-orbital on M.

Activation Strain Analysis

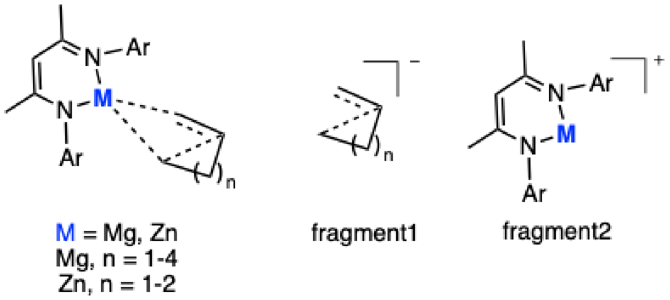

Figure S72. Fragmentation of complexes used for activation strain analysis input for **TS-2** (Mg, n = 1 - 4; Zn n = 1 – 2). Ar = 2,6-diisopropylphenyl.

Activation strain analysis were performed using input files from the AutoDIAS python tool[31] for **TS-2** (Mg, n = 1 - 4; Zn n = 1 – 2). Complexes were fragmented at the M–C^a bond (fragment1=alkyl anion; fragment2 = metal cation, Figure S20). Energy was plotted as a function of bond distance for the: a) breaking M–Ca bond; b) breaking Cb–Cg bond; and c) forming M–Cg bond (M= Mg, Zn). Transition state is noted by a vertical dashed line. The total (E(Tot), interaction (E(Int)), and distortion/strain (E(Dist)) energies at the transition state are tabulated (Table S9); energies in kcal/mol. Transition states are assigned as the geometry where E(Tot) reaches a maximum. DE(X) (X = Int, Dist) were calculated using the equation: DE(X) = $E(X)^{\ddagger}$ – E(X)initial, where E(X) initial is E(X) at the parent metallocyclocomplex (**4a-b**, **5a-b**).

		Distance (A)			Energy						
		M–Ca	Cb–Cg	M–Cg	E(Tot)	E(Int)	E(Dist)			DE(Int)	DE(Dist)
							total	frag1	frag2		
**Mg**	**n = 1**	2.218	1.937	2.263	-131.9	-145.5	13.6	14.0	-0.4	1.1	12.0
	**n = 2**	2.182	2.092	2.273	-124.7	-138.5	13.8	15.5	-1.6	7.7	11.9
	**n = 3**	2.174	2.233	2.245	-114.3	-144.2	29.9	29.9	0.0	5.8	23.1
	**n = 4**	2.161	2.250	2.232	-99.9	-142.3	42.4	42.5	-0.1	8.7	27.7
**Zn**	**n = 1**	2.089	1.925	2.213	-146.5	-159.0	12.5	13.4	-0.9	8.1	11.1
	**n = 2**	2.049	2.094	2.267	-138.9	-152.2	13.2	14.7	-1.4	19.7	12.9

Table S9. Activation strain analysis at the transition state for **TS-2** (Mg, n = 1 - 4; Zn n = 1 – 2). E(Tot), E(Int), and E(Dist) are the total, interaction, and distortion (strain) energy at the transition state, respectively. Energy in $kcalmol^{-1}$.

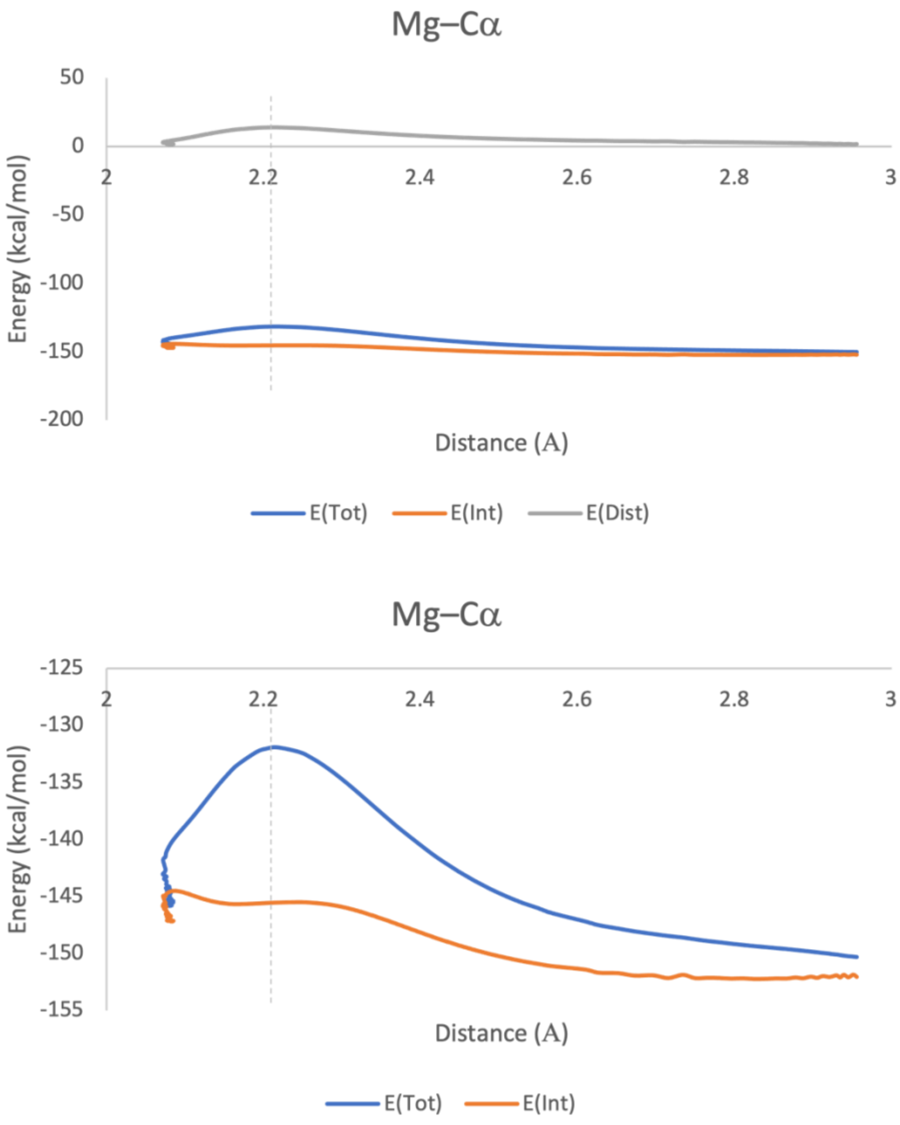

Figure S73. Activation Strain Analysis plot for **TS-2** (Mg, n = 1) along the Mg–Ca bond. Transition state is noted as a dashed vertical line.

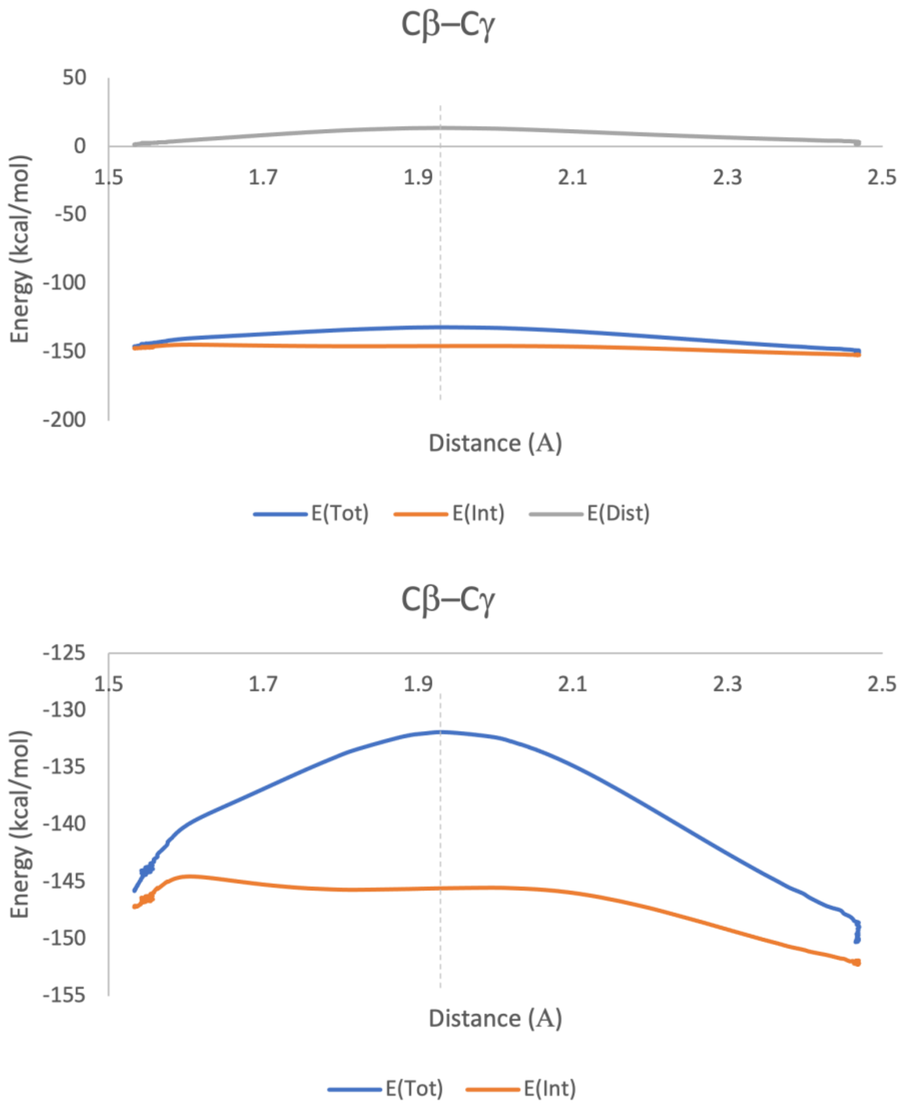

Figure S74. Activation Strain Analysis plot for **TS-2** (Mg, n = 1) along the Cb–Cg bond. Transition state is noted as a dashed vertical line.

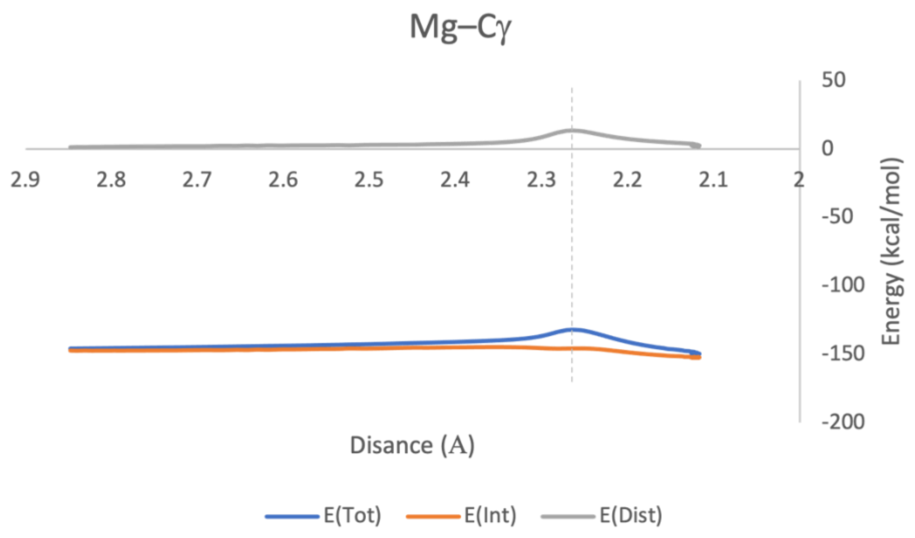

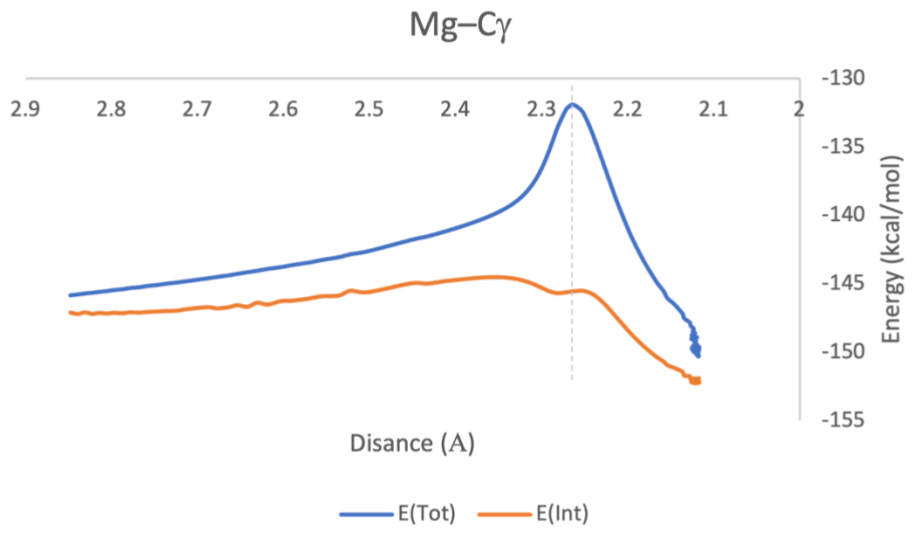

Figure S75. Activation Strain Analysis plot for **TS-2** (Mg, n = 1) along the Mg–Cg bond. Transition state is noted as a dashed vertical line.

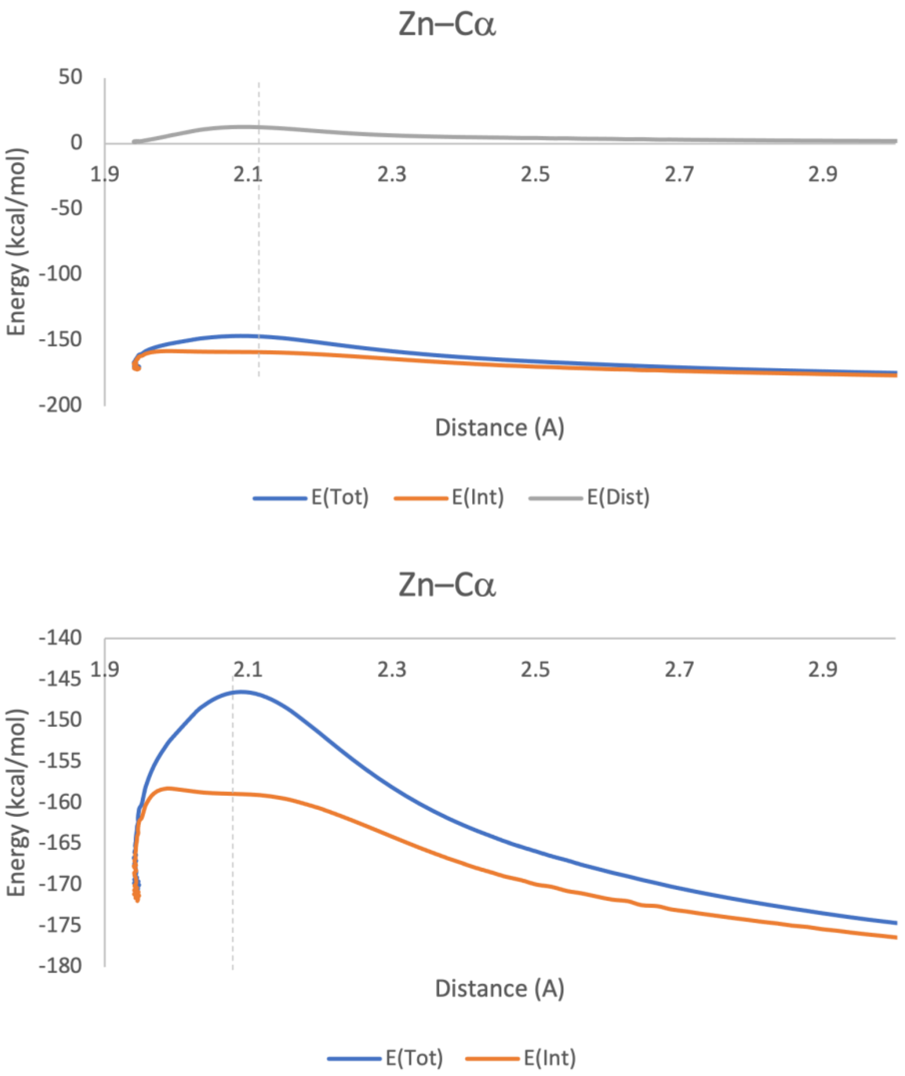

Figure S76. Activation Strain Analysis plot for **TS-2** (Zn, n = 1) along the Zn–Ca bond. Transition state is noted as a dashed vertical line.

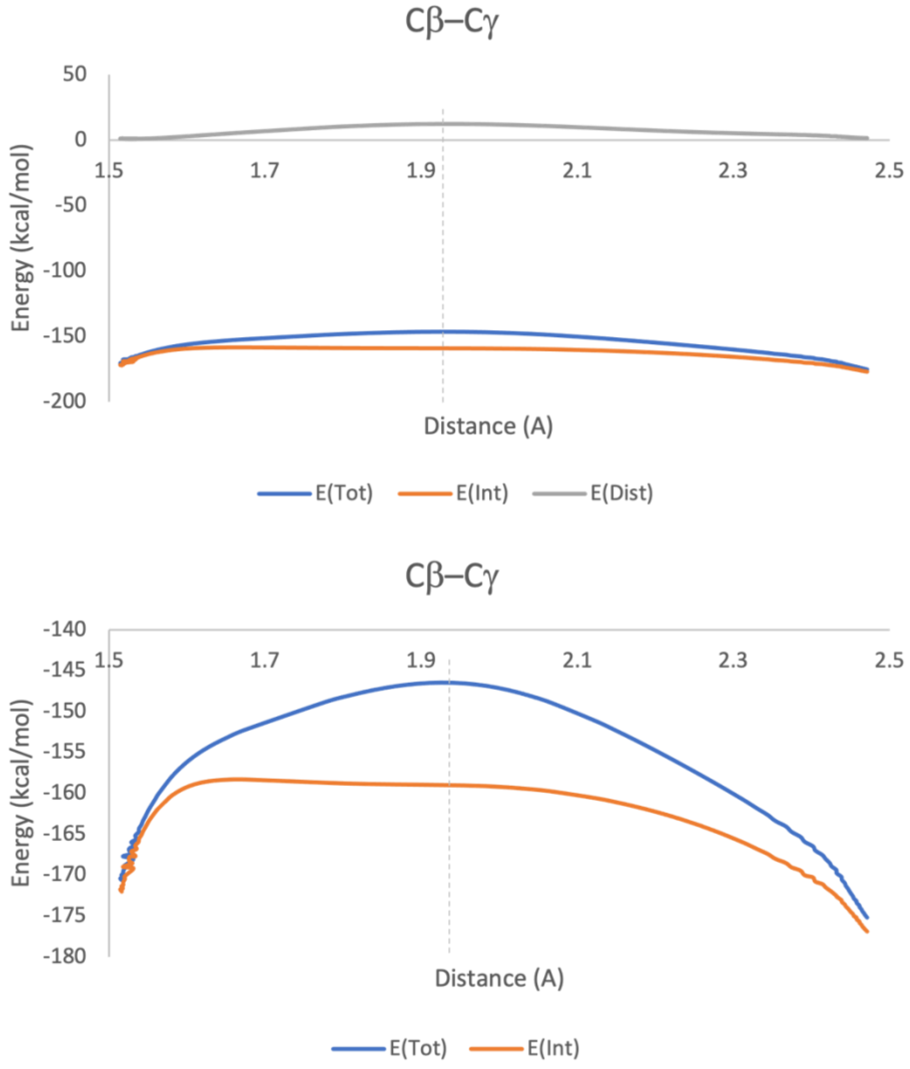

Figure S77. Activation Strain Analysis plot for **TS-2** (Zn, n = 1) along the Cb–Cg bond. Transition state is noted as a dashed vertical line.

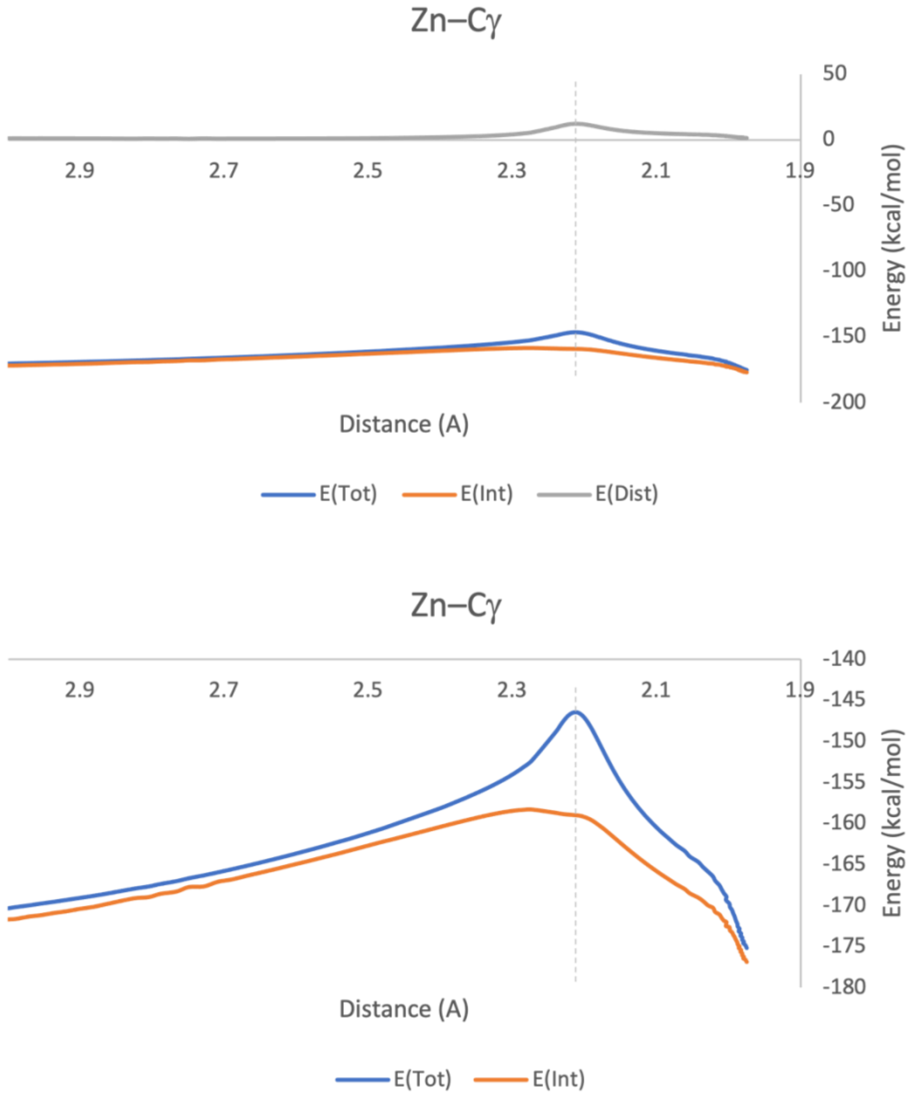

Figure S78. Activation Strain Analysis plot for **TS-2** (Zn, n = 1) along the Zn–Cg bond. Transition state is noted as a dashed vertical line.

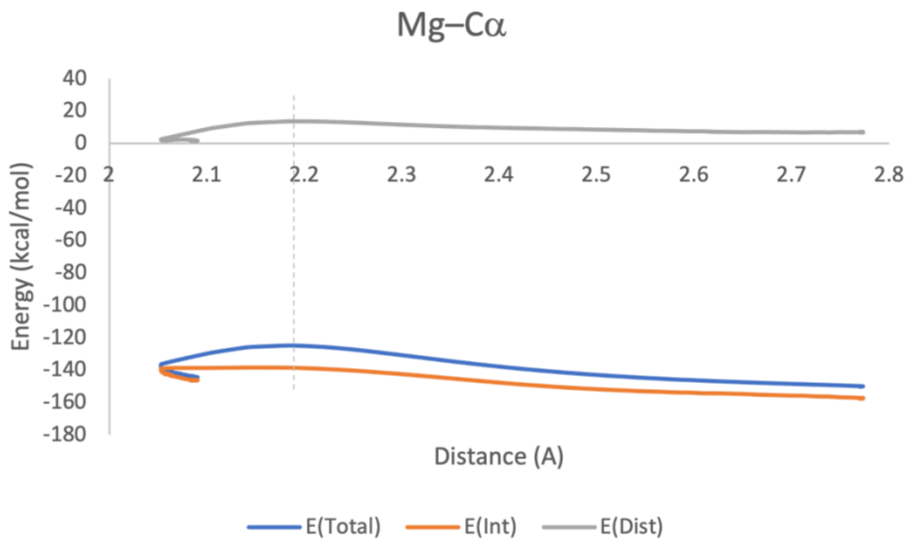

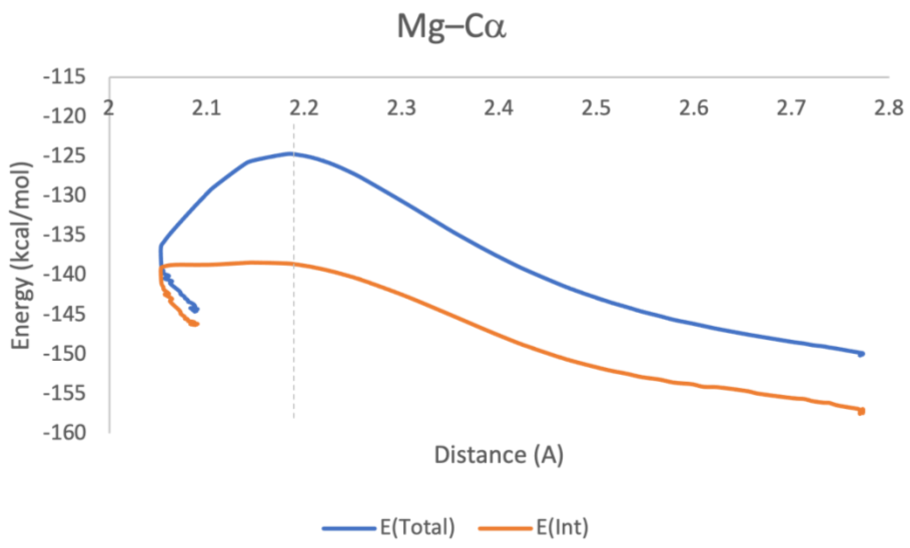

Figure S79. Activation Strain Analysis plot for **TS-2** (Mg, n = 2) along the Mg–Ca bond. Transition state is noted as a dashed vertical line.

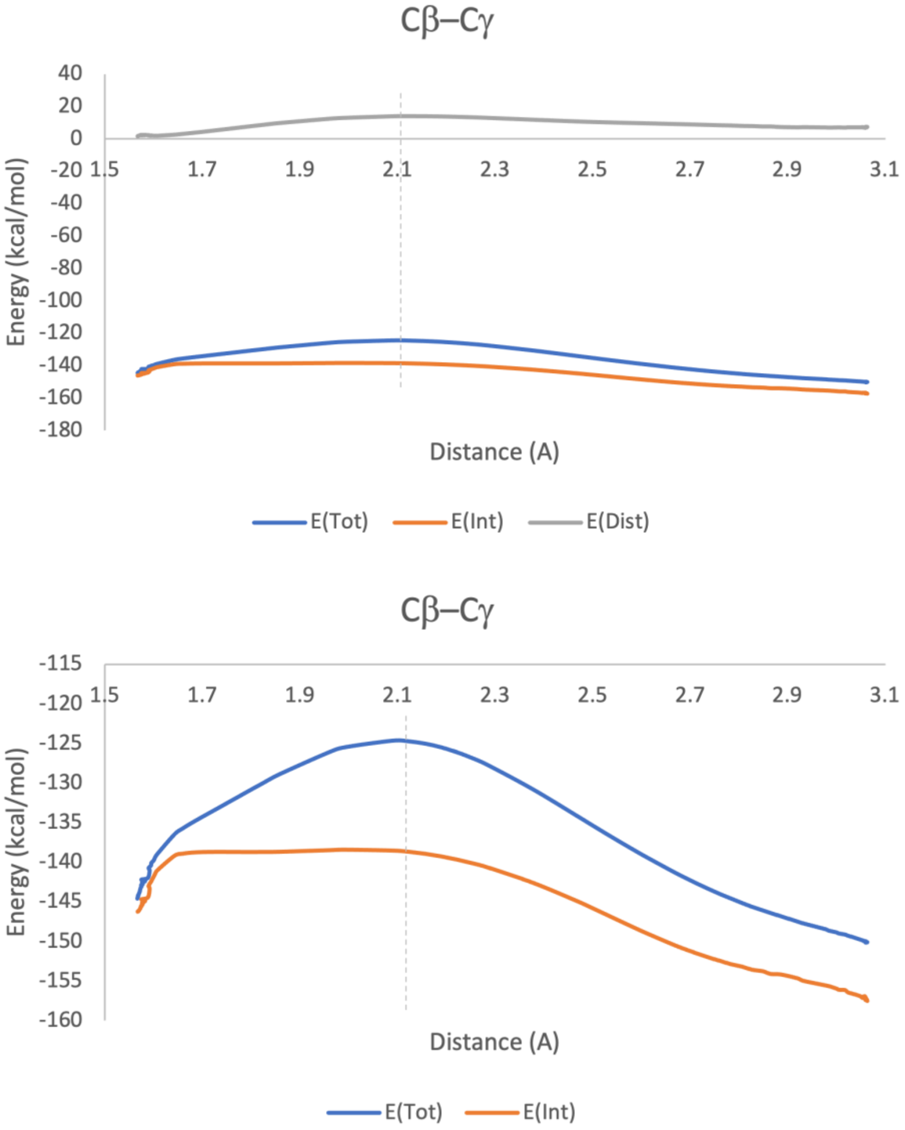

Figure S80. Activation Strain Analysis plot for **TS-2** (Mg, n = 2) along the Cb–Cg bond. Transition state is noted as a dashed vertical line.

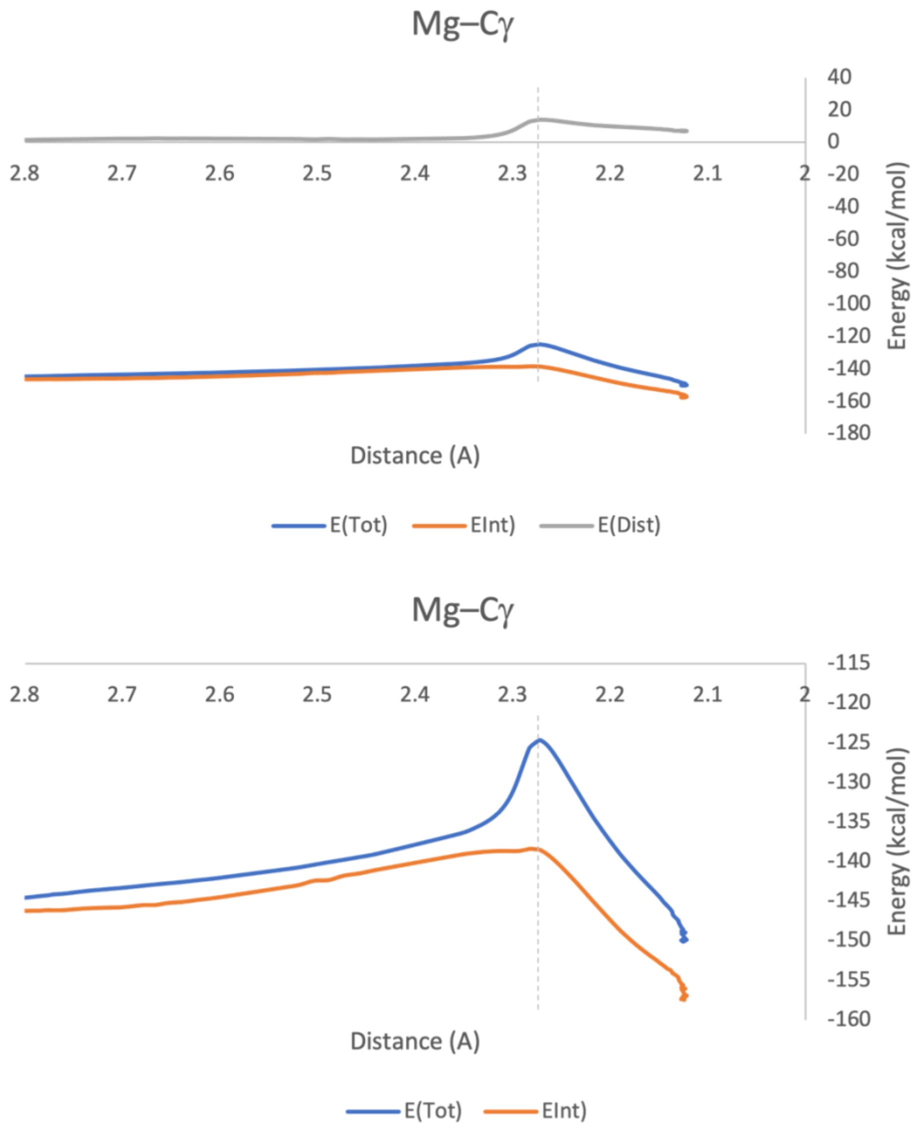

Figure S81. Activation Strain Analysis plot for **TS-2** (Mg, n = 2) along the Mg–Cg bond. Transition state is noted as a dashed vertical line.

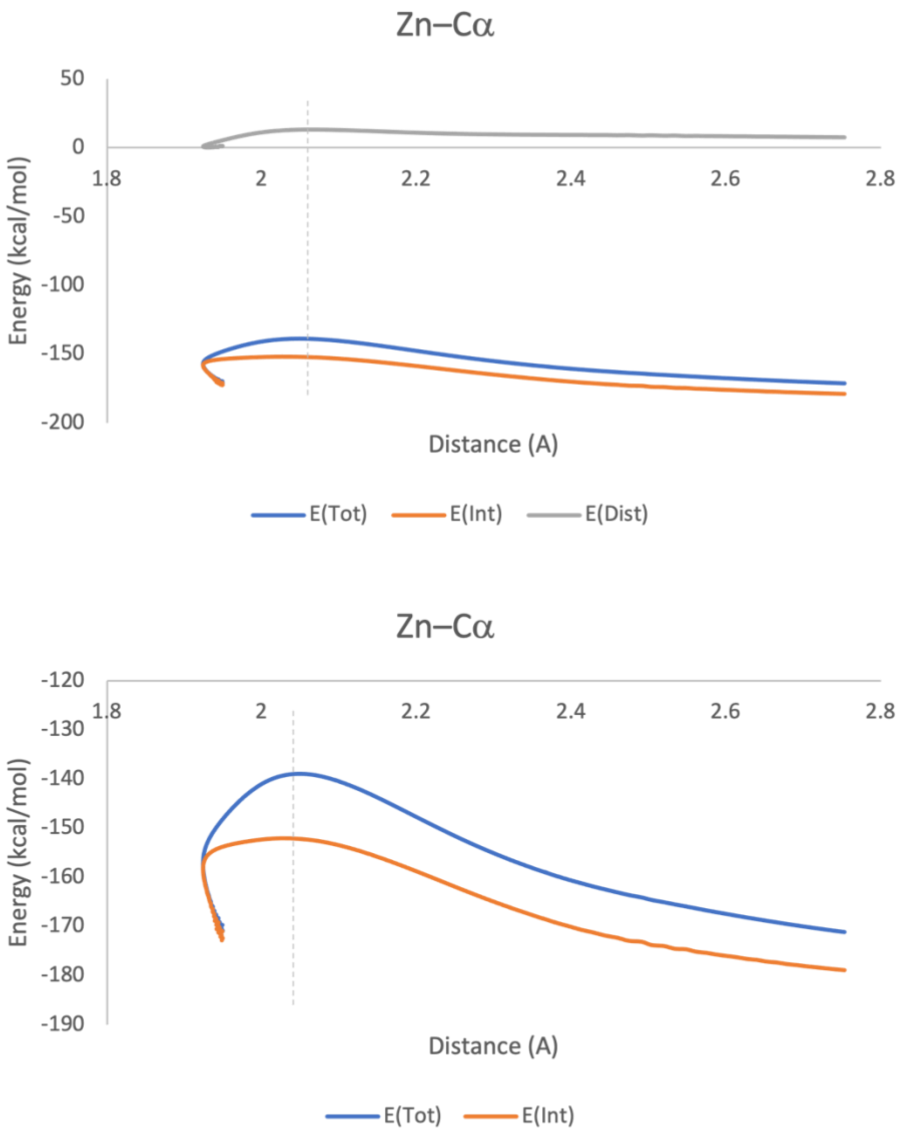

Figure S82. Activation Strain Analysis plot for **TS-2** (Zn, n = 2) along the Zn–Ca bond. Transition state is noted as a dashed vertical line.

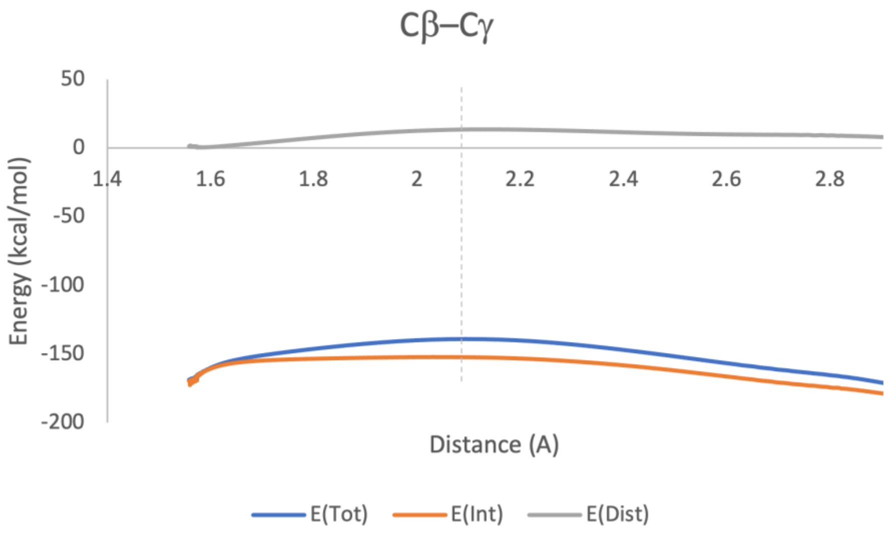

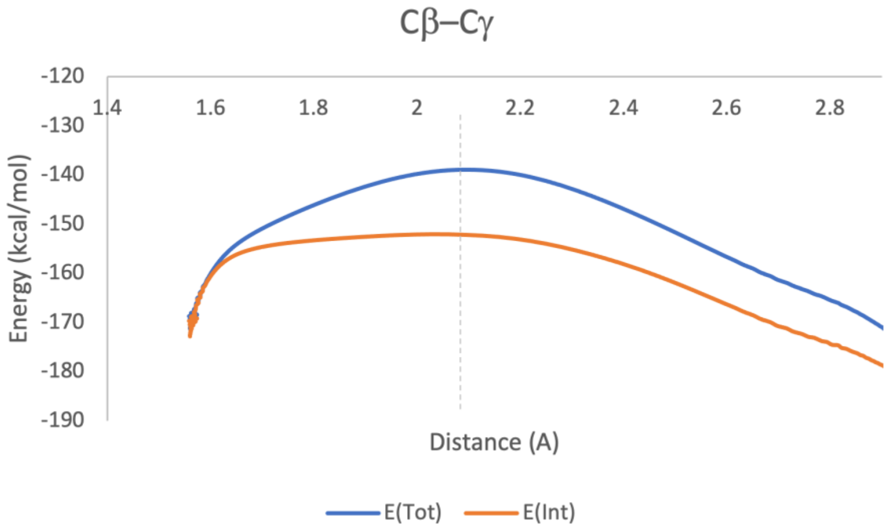

Figure S83. Activation Strain Analysis plot for **TS-2** (Zn, n = 2) along the Cb–Cg bond. Transition state is noted as a dashed vertical line.

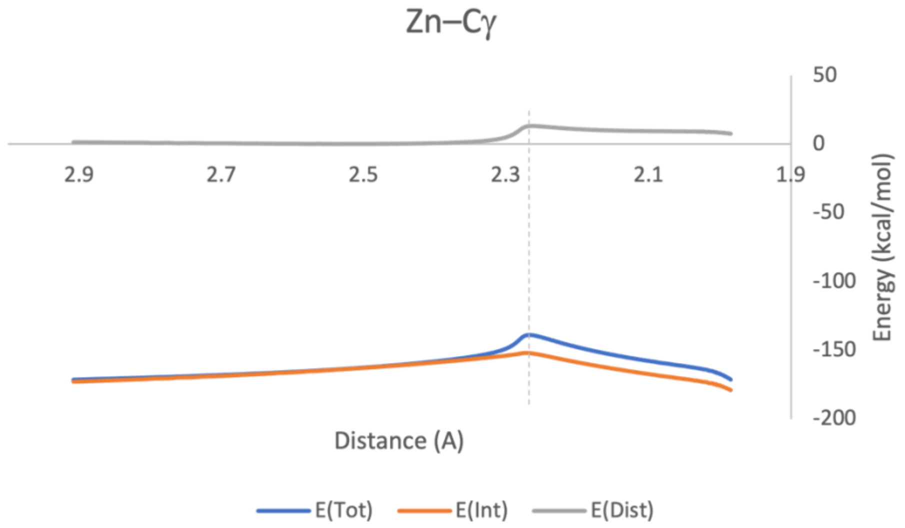

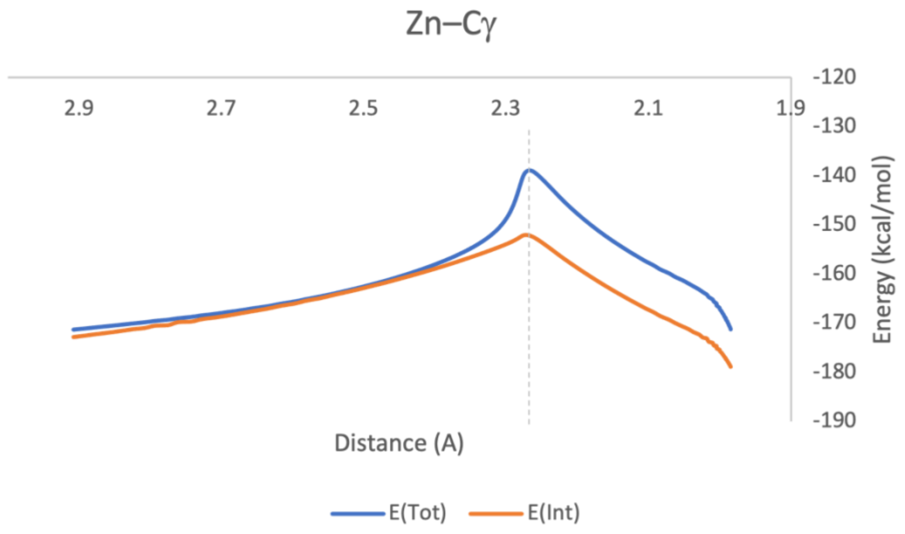

Figure S84. Activation Strain Analysis plot for **TS-2** (Zn, n = 2) along the Zn–Cg bond. Transition state is noted as a dashed vertical line.

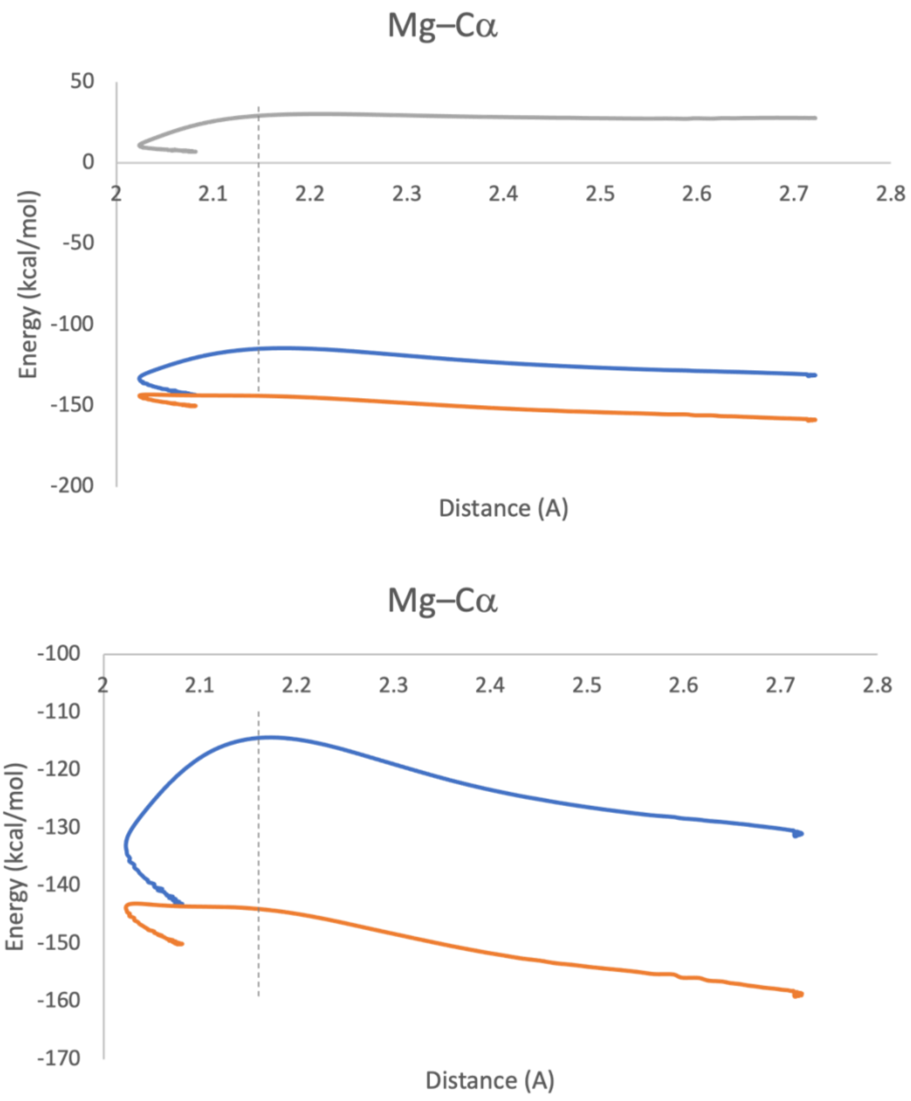

Figure S85. Activation Strain Analysis plot for **TS-2** (Mg, n = 3) along the Mg–Ca bond. Transition state is noted as a dashed vertical line.

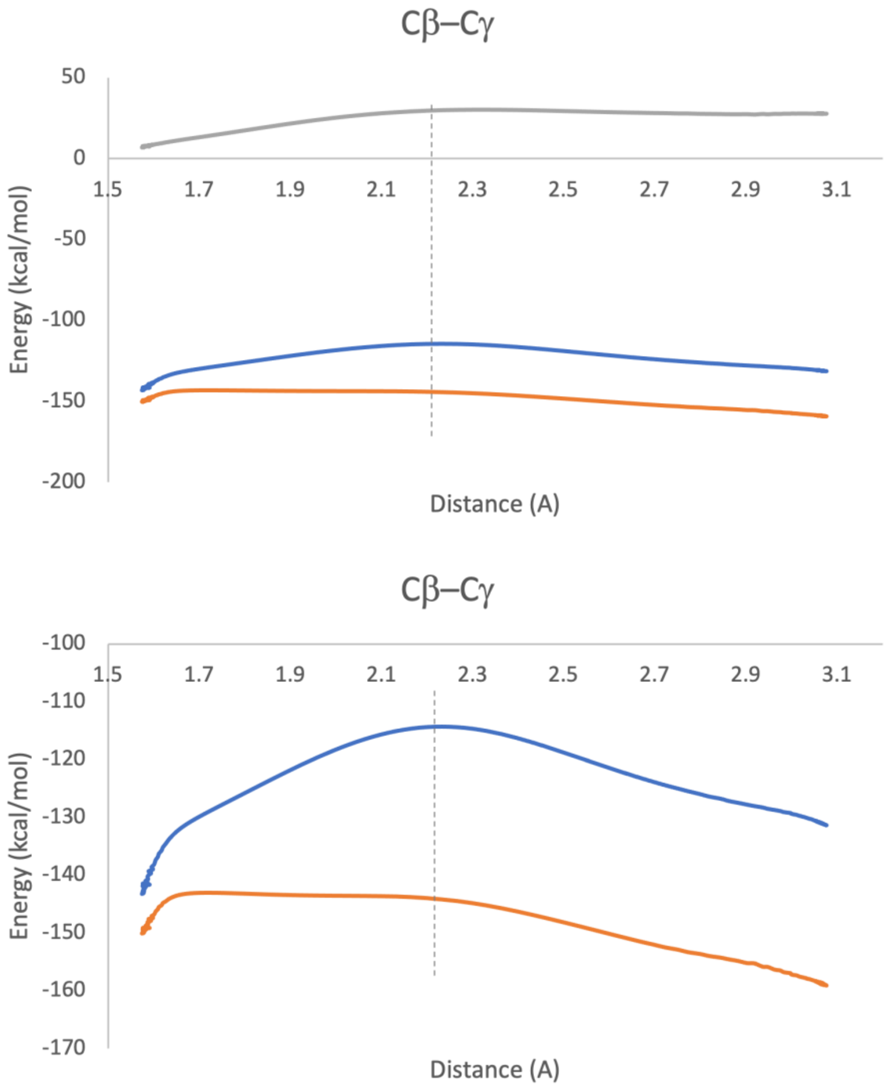

Figure S86. Activation Strain Analysis plot for **TS-2** (Mg, n = 3) along the Cb–Cg bond. Transition state is noted as a dashed vertical line.

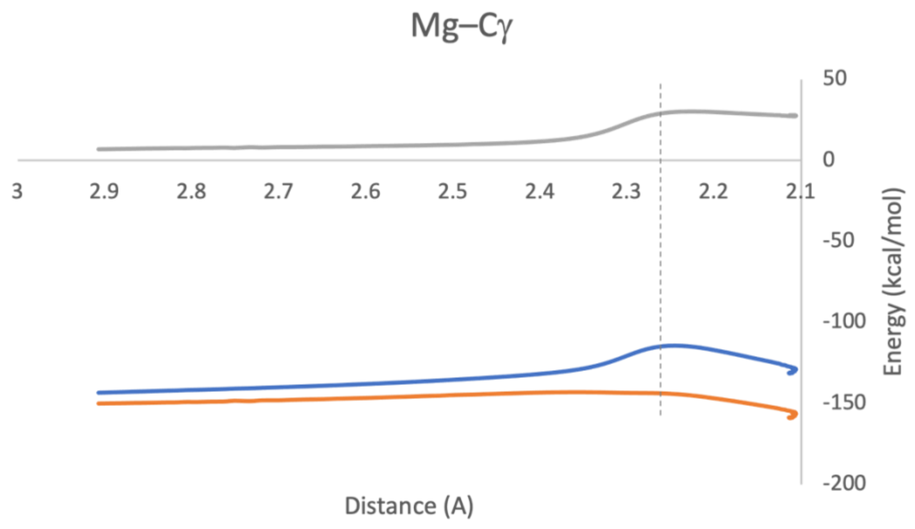

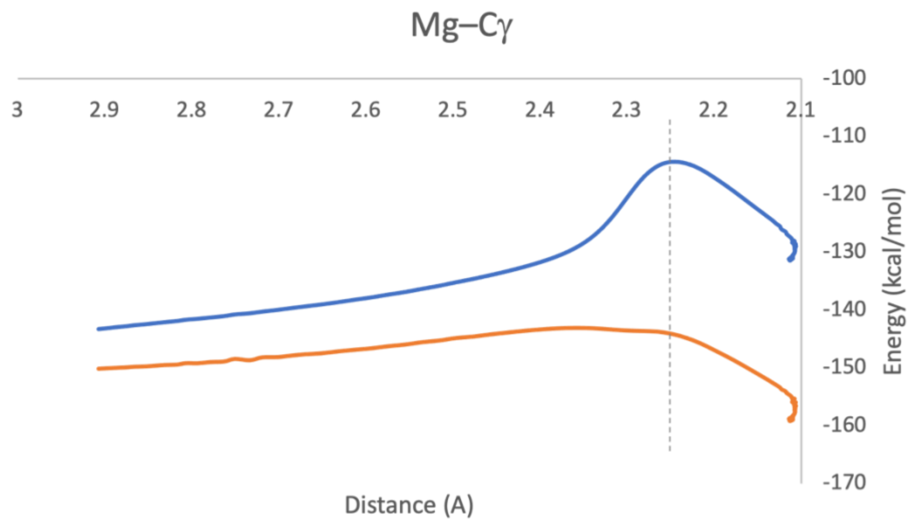

Figure S87. Activation Strain Analysis plot for **TS-2** (Mg, n = 3) along the Mg–Cg bond. Transition state is noted as a dashed vertical line.

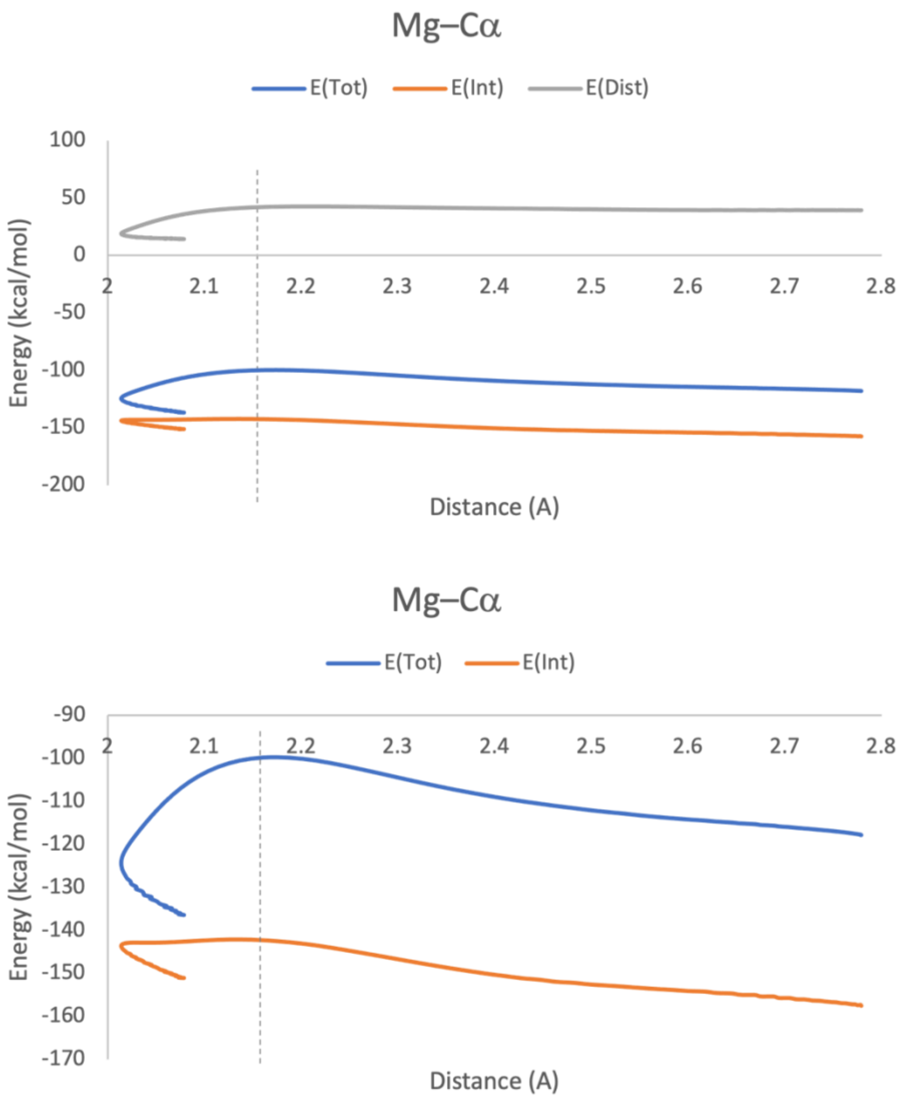

Figure S88. Activation Strain Analysis plot for **TS-2** (Mg, n = 4) along the Mg–Ca bond. Transition state is noted as a dashed vertical line.

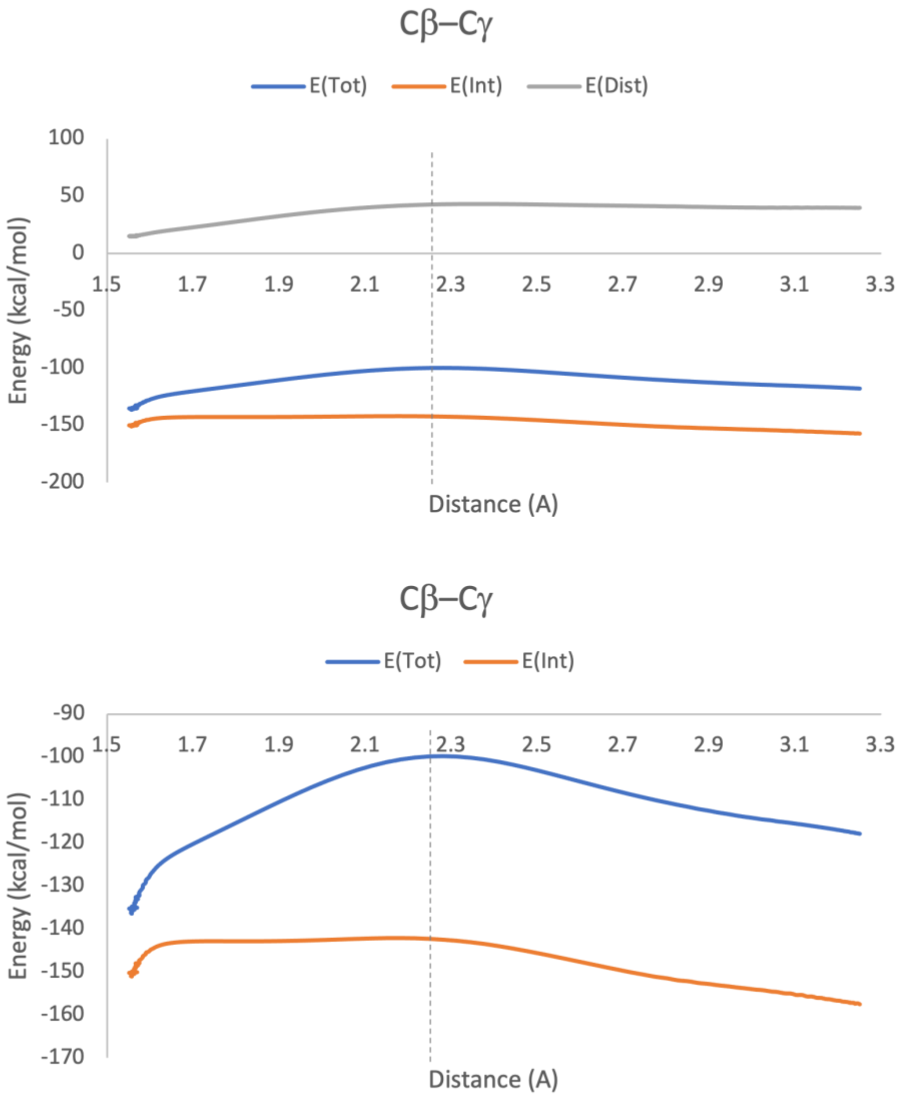

Figure S89. Activation Strain Analysis plot for **TS-2** (Mg, n = 4) along the Cb–Cg bond. Transition state is noted as a dashed vertical line.

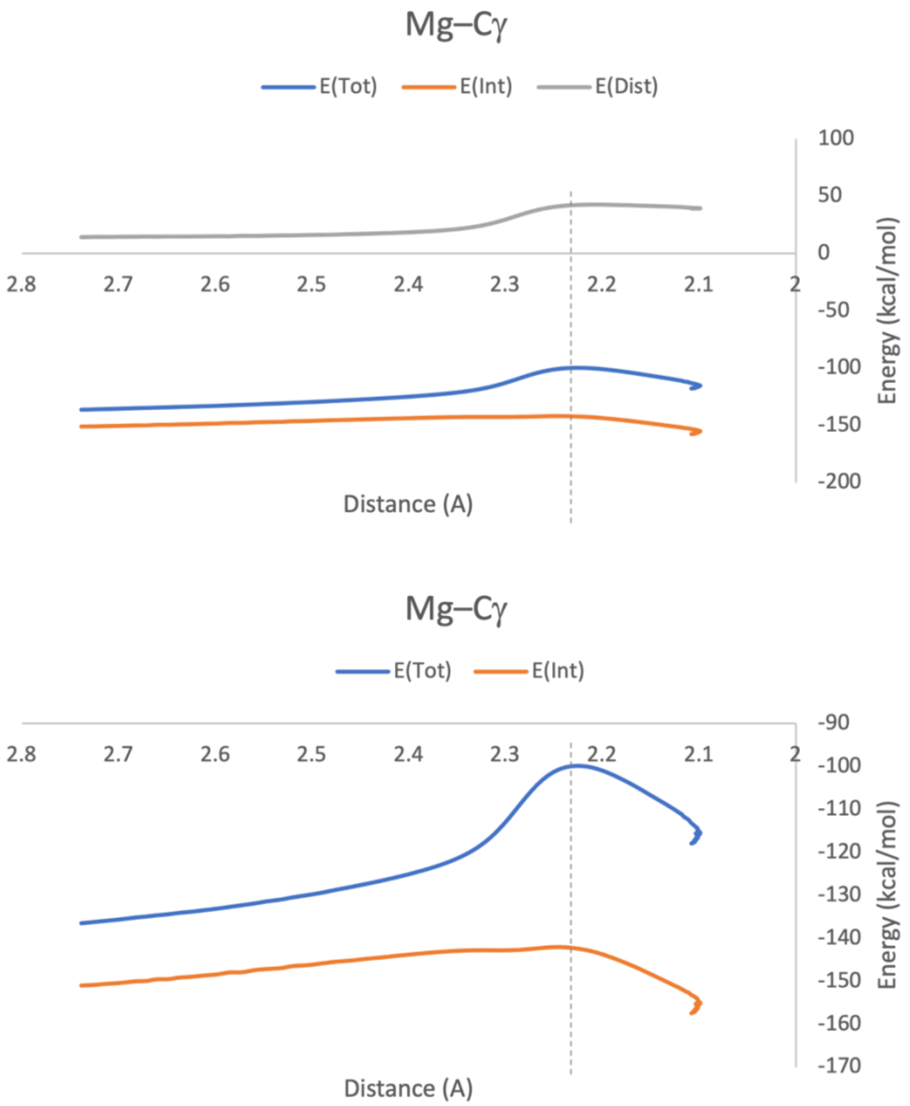

Figure S90. Activation Strain Analysis plot for **TS-2** (Mg, n = 4) along the Mg–Cg bond. Transition state is noted as a dashed vertical line.

Non-covalent interactions (NCI) plots

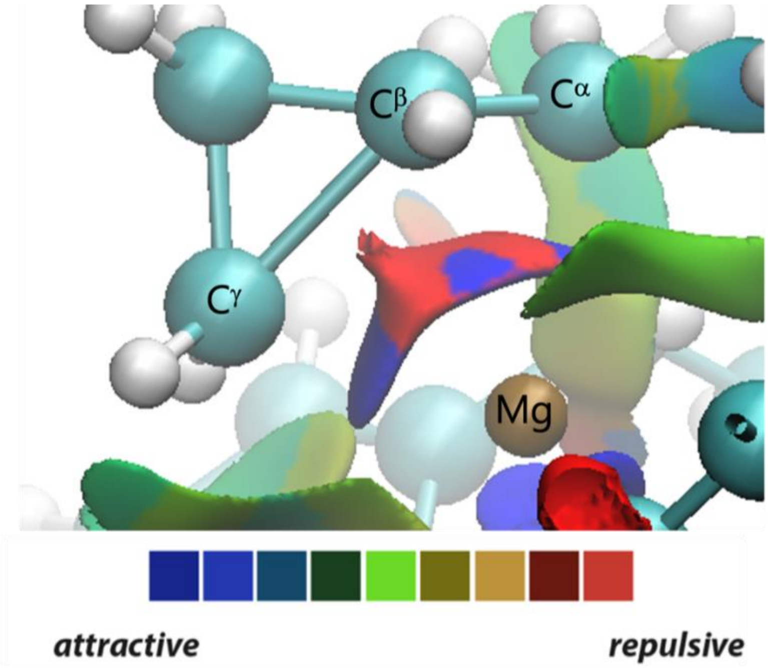

Figure S91. NCI plot for **TS-2** (Mg, n = 1).

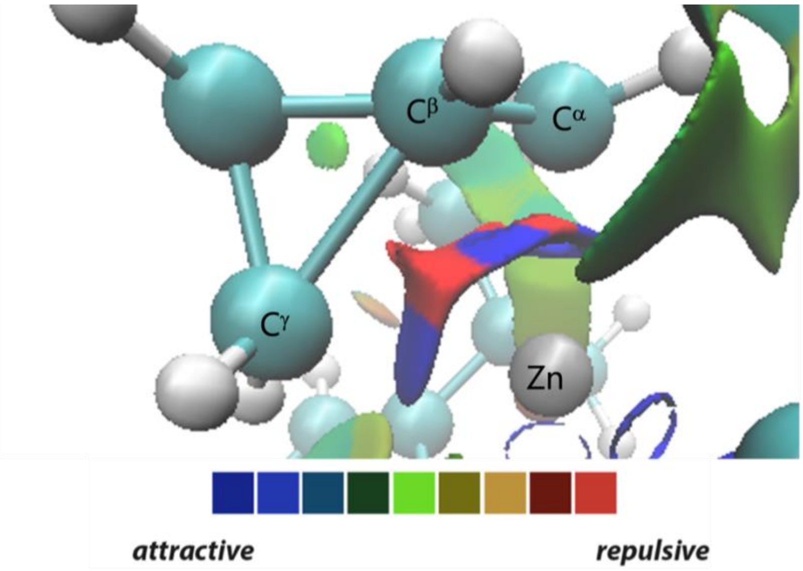

Figure S92. NCI plot for **TS-2** (Zn, n = 1).

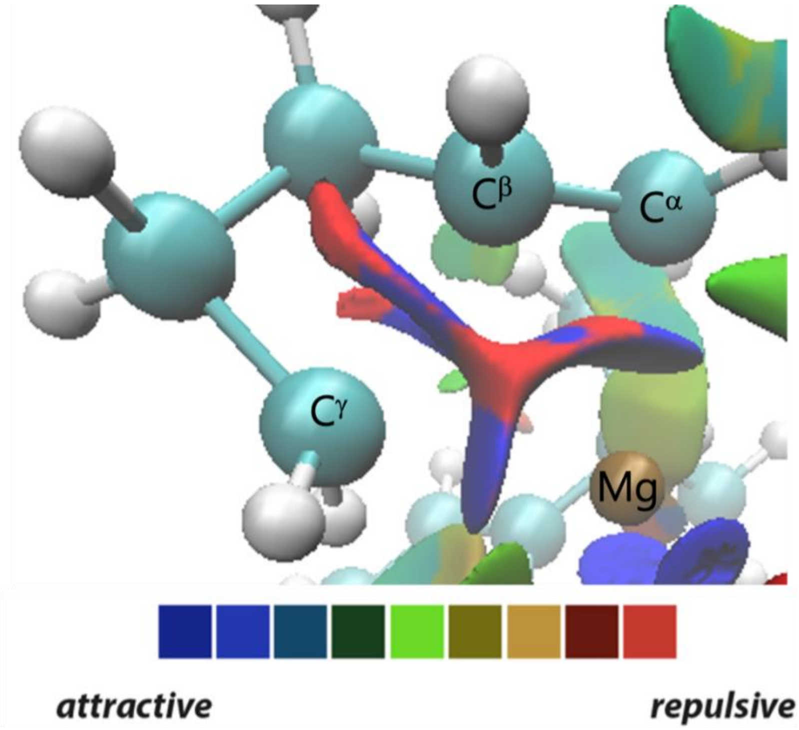

Figure S93. NCI plot for **TS-2** (Mg, n = 2).

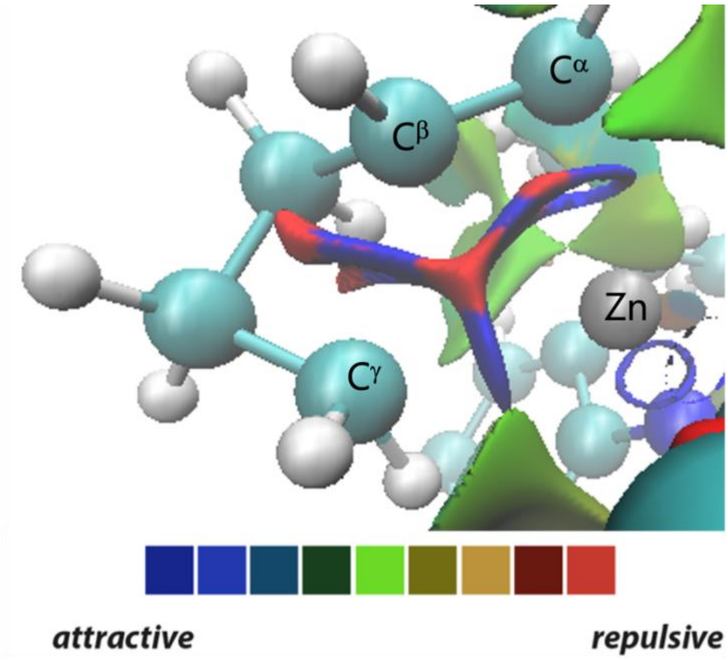

Figure S94. NCI plot for **TS-2** (Zn, n = 2).

Computational coordinates

1.log

SCF (wB97x) = -1240.52016510
E(SCF)+ZPE(0 K)= -1239.870609
H(298 K)= -1239.833784
G(298 K)= -1239.938913
Lowest Frequency = 11.9394cm-1

Mg	5.933331	1.002748	0.938104
N	6.027005	2.968153	1.423556
C	5.180099	5.244226	1.788126
H	5.931262	5.423272	2.561262
H	4.249685	5.735771	2.073414
H	5.552380	5.713270	0.871286
N	3.938367	0.929866	1.260672
C	4.971225	3.761264	1.562780
C	3.638062	3.312384	1.512816
H	2.881439	4.078998	1.622900
C	3.161092	1.992078	1.439737
C	1.672656	1.787641	1.611886
H	1.260926	1.222141	0.770558
H	1.145867	2.738525	1.693337
H	1.475662	1.198041	2.513024
C	7.337382	3.494433	1.631496
C	7.961651	3.264101	2.874525
C	9.247080	3.769875	3.071528
H	9.745298	3.617520	4.023149
C	9.911554	4.454760	2.062254
H	10.912260	4.838483	2.234301
C	9.303575	4.624958	0.826269
H	9.843576	5.131530	0.031956
C	8.015021	4.147462	0.586152
C	7.249898	2.482232	3.972634
H	6.600786	1.744972	3.483417
C	8.209328	1.701543	4.875431
H	8.793481	2.366063	5.520792
H	8.904595	1.093596	4.289740
H	7.642724	1.034845	5.532386
C	6.329234	3.378040	4.813454
H	6.901757	4.186383	5.281287
H	5.852027	2.794401	5.608082
H	5.536683	3.823729	4.207532
C	7.395157	4.263537	-0.797896
H	6.317646	4.103199	-0.698689
C	7.594705	5.642127	-1.436838
H	7.256658	6.444105	-0.773009
H	7.029101	5.709948	-2.371262
H	8.645519	5.830513	-1.679824
C	7.938915	3.150875	-1.707612
H	9.018934	3.264152	-1.850514
H	7.459312	3.183060	-2.691628
H	7.770693	2.159276	-1.273081
C	3.380790	-0.380836	1.372898
C	3.389964	-1.012892	2.631072
C	2.931185	-2.327176	2.719453
H	2.938437	-2.833079	3.680793
C	2.474895	-3.003373	1.595500
H	2.124845	-4.027307	1.680781
C	2.474568	-2.367720	0.360954
H	2.125502	-2.905610	-0.515773
C	2.923076	-1.053490	0.225926
C	3.935106	-0.303373	3.862322
H	4.039789	0.758618	3.620724
C	2.994830	-0.401577	5.068866
H	2.902017	-1.430645	5.431226
H	1.991619	-0.042713	4.819722
H	3.377663	0.203755	5.896702
C	5.332911	-0.834506	4.211614
H	5.292043	-1.897189	4.473297
H	5.753286	-0.289399	5.063385
H	6.027592	-0.738142	3.368571
C	2.958099	-0.387123	-1.140070
H	3.115100	0.684134	-0.980831
C	1.642590	-0.545629	-1.911081
H	1.456073	-1.588794	-2.186599
H	1.675300	0.035435	-2.838007
H	0.789123	-0.198598	-1.320255
C	4.145389	-0.912905	-1.960754
H	5.093876	-0.777392	-1.429459
H	4.211714	-0.396025	-2.923985
H	4.033136	-1.984679	-2.157506
H	7.112800	-0.145412	0.447202

2.log

SCF (wB97x) = -1466.80367503
E(SCF)+ZPE(0 K)= -1466.152900
H(298 K)= -1466.116143
G(298 K)= -1466.220198
Lowest Frequency = 20.1802cm-1

Zn	5.883372	1.048903	1.011224
N	6.015087	2.970617	1.434656
C	5.185782	5.246498	1.815577
H	5.931215	5.416874	2.596154
H	4.256549	5.743881	2.094589
H	5.569829	5.715119	0.903550
N	3.937952	0.944523	1.278898
C	4.964467	3.766741	1.585871
C	3.631359	3.322144	1.544308
H	2.875623	4.087733	1.665390
C	3.158029	2.003142	1.462803
C	1.670789	1.788923	1.627900
H	1.266213	1.222163	0.784107
H	1.140223	2.738019	1.706459
H	1.471978	1.198934	2.528207
C	7.332633	3.484298	1.623664
C	7.971264	3.254867	2.858902
C	9.259484	3.760637	3.036142
H	9.771091	3.609014	3.980753
C	9.909923	4.443545	2.016208
H	10.912775	4.827997	2.173974
C	9.285678	4.611209	0.787966
H	9.814889	5.116007	-0.014582
C	7.993843	4.133309	0.566534
C	7.276394	2.467090	3.963417
H	6.639546	1.718705	3.476920
C	8.251302	1.700179	4.861353
H	8.827704	2.371472	5.507037
H	8.953310	1.104235	4.271457
H	7.696188	1.022406	5.516965
C	6.349870	3.350929	4.810494
H	6.913617	4.168223	5.273689
H	5.887709	2.760823	5.609247
H	5.544937	3.784368	4.212012
C	7.353631	4.240325	-0.808929
H	6.274906	4.103733	-0.691326

C 7.571925 5.602580 -1.475483
H 7.264324 6.423593 -0.820010
H 6.990013 5.666484 -2.400119
H 8.621653 5.763129 -1.742205
C 7.857429 3.098853 -1.705453
H 8.938765 3.180120 -1.860507
H 7.368195 3.128730 -2.684780
H 7.660452 2.122805 -1.250270
C 3.391446 -0.371452 1.376180
C 3.382931 -1.007926 2.631209
C 2.926207 -2.323904 2.703749
H 2.919823 -2.835768 3.661891
C 2.489455 -2.994211 1.568518
H 2.140496 -4.019480 1.642324
C 2.505054 -2.351024 0.337855
H 2.169681 -2.884059 -0.547071
C 2.952102 -1.034924 0.217821
C 3.906160 -0.304160 3.875182
H 4.009443 0.759834 3.642676
C 2.948225 -0.416094 5.066544
H 2.857301 -1.447990 5.421356
H 1.945899 -0.062635 4.805820
H 3.315106 0.186331 5.903682
C 5.302454 -0.828529 4.239973
H 5.263724 -1.892572 4.497329
H 5.706703 -0.284359 5.100512
H 6.002537 -0.718314 3.404654
C 3.008081 -0.356939 -1.141517
H 3.144600 0.715084 -0.970688
C 1.715285 -0.531518 -1.946268
H 1.555011 -1.574612 -2.238273
H 1.760259 0.060506 -2.865742
H 0.840971 -0.206904 -1.373335
C 4.225503 -0.854685 -1.934378
H 5.153902 -0.692662 -1.377469
H 4.302892 -0.334220 -2.894834
H 4.142138 -1.928561 -2.134687
H 6.951621 0.006322 0.650400

3a.log

SCF (wB97x) = -155.913909931
E(SCF)+ZPE(0 K)= -155.827768
H(298 K)= -155.822589
G(298 K)= -155.853798
Lowest Frequency = 300.6872cm-1

C -2.724443 0.393765 -1.756470
C -3.968566 0.476932 -2.186631
C -5.038740 1.296625 -2.765243
C -5.256158 -0.185095 -2.422784
H -2.324849 -0.537543 -1.365827
H -4.977650 1.569504 -3.815761
H -5.526311 2.038026 -2.137227
H -5.338295 -0.888329 -3.247723
H -5.886977 -0.419955 -1.569155
H -2.061897 1.254038 -1.779846

3b.log

SCF (wB97x) = -195.240200641
E(SCF)+ZPE(0 K)= -195.124007
H(298 K)= -195.117990
G(298 K)= -195.151960
Lowest Frequency = 94.6863cm-1

C -2.921105 0.394177 -1.879645
C -4.184434 0.489308 -2.269846
C -5.009484 1.595000 -2.904738
C -6.012704 0.503469 -3.370425
C -5.318574 -0.509894 -2.418367
H -2.513678 -0.541605 -1.507796
H -4.522835 2.197351 -3.676834
H -5.444440 2.270225 -2.159537
H -5.873962 0.240196 -4.421609
H -7.071889 0.700255 -3.191813
H -5.064670 -1.493125 -2.824041
H -5.873467 -0.651718 -1.484349
H -2.251200 1.248249 -1.921515

3c.log

SCF (wB97x) = -234.578529852
E(SCF)+ZPE(0 K)= -234.431900
H(298 K)= -234.425141
G(298 K)= -234.461288
Lowest Frequency = 69.6322cm-1

C -5.084546 -0.160710 -0.050762
C -3.575444 -0.000886 -0.144441
C -3.248939 1.483619 -0.180114
C -4.562503 2.177755 0.213642
C -5.641073 1.240676 -0.348719
H -5.466862 -0.934841 -0.722031
H -5.354153 -0.457357 0.971198
H -2.405921 1.743461 0.466268
H -2.971296 1.768409 -1.203316
H -4.648017 2.223359 1.305969
H -4.638400 3.199852 -0.166656
H -6.629565 1.411011 0.085912
H -5.725974 1.386259 -1.432314
C -2.690300 -0.992471 -0.187474
H -1.622269 -0.799079 -0.234922
H -3.001991 -2.033192 -0.176725

3d.log

SCF (wB97x) = -273.893114480
E(SCF)+ZPE(0 K)= -273.716265
H(298 K)= -273.708769
G(298 K)= -273.746421
Lowest Frequency = 123.6942cm-1

C -4.142119 -1.314162 -0.178892
C -2.637334 -1.309963 -0.296781
C -2.013629 0.059714 -0.181358
C -2.470187 0.764319 1.106583
C -3.997954 0.800592 1.206950
C -4.594215 -0.606669 1.109043
H -0.921982 -0.009331 -0.218830
H -4.567728 -0.776714 -1.039156
H -4.528782 -2.337436 -0.214689
H -2.063861 0.222925 1.970544
H -2.060410 1.779449 1.142962
H -4.304272 1.276867 2.144531
H -4.398644 1.419878 0.392289

H	-4.266859	-1.199000	1.973116
H	-5.687963	-0.561989	1.147067
H	-2.329500	0.667929	-1.041796
C	-1.919547	-2.422188	-0.445767
H	-0.835279	-2.394004	-0.512999
H	-2.391697	-3.398783	-0.511241

4a.log

SCF (wB97x) = -1396.50748940
E(SCF)+ZPE(0 K)= -1395.763733
H(298 K)= -1395.722260
G(298 K)= -1395.836889
Lowest Frequency = 15.3103cm-1

Mg	0.347522	-0.001801	-0.218917
N	0.376553	1.966432	0.251850
C	-0.473349	4.147600	0.964497
H	0.255505	4.249172	1.774684
H	-1.408153	4.618938	1.268415
H	-0.065324	4.690692	0.106204
N	-1.634143	-0.152331	0.183556
C	-0.672722	2.687875	0.622654
C	-1.980246	2.177208	0.723179
H	-2.741902	2.891143	1.010440
C	-2.425986	0.858586	0.522490
C	-3.903055	0.594580	0.714583
H	-4.442448	1.506417	0.971309
H	-4.062371	-0.144999	1.505545
H	-4.333177	0.173304	-0.199516
C	1.671513	2.563978	0.190330
C	2.538327	2.437071	1.290317
C	3.833267	2.945977	1.180295
H	4.517895	2.850998	2.018465
C	4.263929	3.561176	0.013189
H	5.274694	3.950793	-0.056436
C	3.400807	3.666982	-1.070918
H	3.750922	4.136417	-1.985181
C	2.099256	3.171263	-1.006024
C	2.104188	1.709533	2.553691
H	1.022804	1.552202	2.492065
C	2.770866	0.327212	2.626473
H	3.858280	0.425003	2.714660
H	2.572084	-0.261832	1.723909
H	2.407931	-0.236518	3.492269
C	2.373077	2.521037	3.825909
H	3.444941	2.666677	3.995771
H	1.970446	2.000200	4.700436
H	1.905681	3.509028	3.771902
C	1.183513	3.219189	-2.219861
H	0.152933	3.124946	-1.864874
C	1.275839	4.534881	-2.999350
H	1.125148	5.397771	-2.343409
H	0.511310	4.562664	-3.781915
H	2.246832	4.652056	-3.491625
C	1.463455	2.019440	-3.138776
H	2.480138	2.074426	-3.542370
H	0.761421	1.997888	-3.979037
H	1.385072	1.067183	-2.601769
C	-2.170184	-1.469885	0.058692
C	-2.202428	-2.308874	1.190144
C	-2.609919	-3.631452	1.024014
H	-2.635290	-4.296862	1.881244
C	-2.978149	-4.118032	-0.224789
H	-3.283480	-5.153571	-0.336293
C	-2.955846	-3.275816	-1.327007
H	-3.246242	-3.661166	-2.300349
C	-2.558789	-1.942786	-1.206697
C	-1.763887	-1.787525	2.551418
H	-1.922970	-0.704777	2.560357
C	-2.573772	-2.371970	3.713279
H	-2.362195	-3.435409	3.866073
H	-3.649392	-2.262926	3.544621
H	-2.319220	-1.855709	4.643969
C	-0.263188	-2.025012	2.768435
H	-0.033283	-3.095490	2.756236
H	0.061815	-1.610770	3.728643
H	0.341118	-1.564393	1.980971
C	-2.519686	-1.041545	-2.431070
H	-2.360588	-0.016444	-2.082449
C	-3.840010	-1.057734	-3.210533
H	-4.035210	-2.038280	-3.656982
H	-3.808747	-0.327044	-4.024914
H	-4.687123	-0.811458	-2.562987
C	-1.338347	-1.409860	-3.339941
H	-0.387762	-1.362515	-2.798846
H	-1.279230	-0.731133	-4.197301
H	-1.444726	-2.431217	-3.721665
H	2.183451	-2.588235	0.891031
C	1.856126	-1.312733	-0.882512
C	1.853054	-2.643251	-0.147260
C	2.181566	-3.945993	-0.832949
C	0.763578	-3.651427	-0.399988
H	1.716780	-1.514001	-1.957354
H	2.731373	-4.709735	-0.289697
H	2.405028	-3.903462	-1.895585
H	0.350986	-4.212527	0.433187
H	0.032312	-3.415391	-1.169705
H	2.855735	-0.857489	-0.812783

4b.log

SCF (wB97x) = -1435.82069030
E(SCF)+ZPE(0 K)= -1435.047329
H(298 K)= -1435.004931
G(298 K)= -1435.122025
Lowest Frequency = 11.1541cm-1

Mg	0.292155	-0.021593	-0.274071
N	0.354024	1.941341	0.216516
C	-0.460443	4.132937	0.940058
H	0.273755	4.216967	1.747202
H	-1.386872	4.615164	1.252428
H	-0.048938	4.677578	0.084345
N	-1.683365	-0.151479	0.161666
C	-0.682742	2.679221	0.586818
C	-1.999388	2.188967	0.678502
H	-2.751942	2.915109	0.959101
C	-2.460708	0.873892	0.494876
C	-3.938233	0.628277	0.707793
H	-4.469085	1.553994	0.930439
H	-4.095744	-0.075166	1.531565
H	-4.378918	0.170972	-0.183565
C	1.663914	2.507452	0.184030
C	2.518316	2.317907	1.284418
C	3.825435	2.801358	1.204053

H	4.499378	2.660212	2.044640
C	4.280995	3.448593	0.064499
H	5.300850	3.817356	0.017790
C	3.430759	3.612119	-1.022852
H	3.800488	4.104281	-1.917202
C	2.117764	3.145184	-0.986846
C	2.061117	1.561570	2.522809
H	0.989672	1.363286	2.416741
C	2.777389	0.206356	2.620378
H	3.857134	0.342636	2.743649
H	2.625144	-0.391454	1.715131
H	2.408904	-0.367353	3.477125
C	2.245598	2.380010	3.806166
H	3.303081	2.580628	4.007733
H	1.842979	1.835880	4.666521
H	1.729737	3.342815	3.739772
C	1.217089	3.252719	-2.207956
H	0.180506	3.172707	-1.867344
C	1.349795	4.588414	-2.946411
H	1.214017	5.434912	-2.266202
H	0.594147	4.658769	-3.734895
H	2.328262	4.695937	-3.425888
C	1.478758	2.073804	-3.158515
H	2.501762	2.114461	-3.547533
H	0.787590	2.095041	-4.007764
H	1.369005	1.109399	-2.649783
C	-2.235182	-1.466330	0.089481
C	-2.271796	-2.260376	1.253249
C	-2.713345	-3.578109	1.144801
H	-2.745746	-4.208548	2.027727
C	-3.097972	-4.106699	-0.082120
H	-3.426558	-5.138879	-0.150076
C	-3.058425	-3.312168	-1.218537
H	-3.358220	-3.732082	-2.174431
C	-2.638115	-1.982546	-1.153952
C	-1.777085	-1.705281	2.581019
H	-1.904228	-0.618595	2.558719
C	-2.558868	-2.227777	3.790222
H	-2.372338	-3.291544	3.970647
H	-3.636412	-2.091901	3.657194
H	-2.255284	-1.689509	4.693295
C	-0.276019	-1.982969	2.748550
H	-0.080102	-3.060340	2.768925
H	0.101845	-1.544194	3.677893
H	0.311715	-1.570738	1.921304
C	-2.595630	-1.127130	-2.410755
H	-2.449238	-0.088749	-2.097554
C	-3.908751	-1.182031	-3.200522
H	-4.088860	-2.176895	-3.620824
H	-3.878244	-0.474883	-4.035408
H	-4.764149	-0.926820	-2.567509
C	-1.403747	-1.516213	-3.297023
H	-0.455072	-1.432491	-2.756351
H	-1.351319	-0.874419	-4.182838
H	-1.493497	-2.554782	-3.634150
H	2.073656	-2.598577	0.759577
C	1.858731	-1.264171	-0.934609
C	1.905135	-2.658776	-0.326713
C	2.848806	-3.758455	-0.893758
C	1.736992	-4.796606	-0.624905
C	0.724361	-3.630118	-0.582619
H	1.821068	-1.370013	-2.032087
H	3.822154	-3.903518	-0.414972
H	3.003568	-3.607853	-1.968305
H	1.851596	-5.262124	0.359406
H	1.579176	-5.584458	-1.366566
H	-0.088714	-3.671024	0.148044
H	0.288366	-3.448937	-1.571968
H	2.808779	-0.741315	-0.735145

4c.log

SCF (wB97x) = -1475.15526088
E(SCF)+ZPE(0 K)= -1474.351952
H(298 K)= -1474.308454
G(298 K)= -1474.428378
Lowest Frequency = 15.1988cm-1

Mg	6.014093	14.904442	4.377551
N	7.178024	16.433985	3.724137
N	7.241073	13.520853	3.552835
C	8.351281	16.297126	3.115088
C	8.916526	15.061037	2.757210
H	9.875486	15.110669	2.256862
C	8.382653	13.769273	2.927508
C	9.138703	17.540674	2.765715
H	8.573455	18.170607	2.071480
H	10.098228	17.291942	2.312014
H	9.314707	18.142193	3.663008
C	9.171564	12.613917	2.352857
H	9.458755	11.914170	3.144150
H	10.071526	12.957971	1.842845
H	8.555221	12.050785	1.645372
C	6.649699	17.737247	3.968806
C	6.826212	18.330324	5.230753
C	6.177026	19.537067	5.496629
H	6.287641	19.999516	6.473258
C	5.385170	20.151050	4.537137
H	4.882770	21.086413	4.762190
C	5.233561	19.563021	3.286811
H	4.608450	20.048849	2.544391
C	5.854337	18.352680	2.981275
C	7.665888	17.656788	6.304622
H	8.231334	16.851754	5.825242
C	6.767361	17.019600	7.374679
H	6.177055	17.784674	7.890791
H	7.367176	16.490181	8.122514
H	6.058828	16.308708	6.936136
C	8.684320	18.611846	6.937979
H	9.313937	19.082618	6.176735
H	9.334199	18.067545	7.630376
H	8.194741	19.409040	7.506851
C	5.616339	17.667496	1.643613
H	6.471791	17.015479	1.442219
C	6.750429	12.184102	3.650785
C	5.798987	11.734756	2.718219
C	5.261547	10.456383	2.878181
H	4.522847	10.095235	2.167798
C	5.646438	9.644562	3.935480
H	5.217560	8.653562	4.046239
C	6.574034	10.109390	4.860335
H	6.856900	9.474541	5.694448
C	7.136814	11.379055	4.739896
C	5.317882	12.625789	1.582728
H	5.929239	13.533910	1.588217
C	5.497865	11.966590	0.210088

H	6.539307	11.674361	0.044154
H	5.206763	12.658753	-0.586521
H	4.878922	11.068752	0.110177
C	3.858380	13.046929	1.809711
H	3.193285	12.176814	1.795657
H	3.527379	13.738145	1.027629
H	3.731747	13.539366	2.779857
C	8.088836	11.914392	5.798669
H	8.666831	12.727146	5.348894
C	7.291031	12.512653	6.968065
H	6.581814	13.275852	6.628262
H	7.960109	12.969003	7.705280
H	6.705759	11.735234	7.470651
C	9.090561	10.869351	6.300122
H	8.601077	10.074187	6.871953
H	9.824182	11.339121	6.962541
H	9.629156	10.403014	5.469393
C	4.213883	14.584237	5.422374
H	3.911340	13.540657	5.234157
H	4.384165	14.629666	6.512061
C	1.922146	17.668032	5.299519
H	1.760102	18.051710	4.285855
H	1.832255	18.523642	5.975175
C	0.893733	16.545274	5.622430
H	0.273740	16.791153	6.489724
H	0.209625	16.403221	4.779244
C	1.733552	15.273932	5.855563
H	1.223710	14.357392	5.541551
H	1.972676	15.162131	6.921764
C	4.370706	16.772572	1.720458
H	4.458292	16.015901	2.507511
H	4.204889	16.248798	0.773324
H	3.479251	17.366050	1.950268
C	5.504962	18.644922	0.469400
H	4.589221	19.243130	0.519289
H	5.478779	18.093461	-0.475384
H	6.355905	19.332160	0.440904
C	3.045787	15.516428	5.090432
H	2.807829	15.437446	4.015004
C	3.302805	16.998839	5.391572
H	4.036772	17.456807	4.722749
H	3.702506	17.082267	6.411876

4d.log

SCF (wB97x) = -1514.47257144
E(SCF)+ZPE(0 K)= -1513.639049
H(298 K)= -1513.595042
G(298 K)= -1513.714661
Lowest Frequency = 15.6656cm-1

Mg	5.740881	14.557472	4.397323
N	6.694248	16.265726	3.852792
N	7.149462	13.404763	3.509219
C	7.878327	16.325948	3.252906
C	8.607202	15.201167	2.830081
H	9.553460	15.409259	2.346618
C	8.250432	13.841509	2.915116
C	8.490353	17.683712	2.988502
H	7.847777	18.271644	2.325339
H	9.477299	17.596112	2.533865
H	8.577483	18.247885	3.922382
C	9.190342	12.839630	2.282337
H	9.568764	12.140017	3.034415
H	10.036111	13.332092	1.802152
H	8.657769	12.240652	1.537313
C	5.986247	17.465490	4.163017
C	6.066821	18.001603	5.459752
C	5.239155	19.076008	5.789467
H	5.275297	19.489892	6.793107
C	4.361146	19.615072	4.860190
H	3.718474	20.445417	5.135195
C	4.304063	19.086834	3.575753
H	3.609896	19.511890	2.857811
C	5.108180	18.010358	3.204529
C	6.987148	17.391993	6.504845
H	7.664733	16.705489	5.987786
C	6.179120	16.570655	7.520565
H	5.487915	17.213710	8.076269
H	6.842077	16.080222	8.241144
H	5.574984	15.800636	7.028597
C	7.853276	18.442662	7.209053
H	8.418409	19.039759	6.486788
H	8.565834	17.956743	7.882967
H	7.249318	19.129559	7.811027
C	4.983354	17.381133	1.824807
H	5.925308	16.871390	1.600590
C	6.843115	12.010807	3.522766
C	5.961162	11.494254	2.557316
C	5.601934	10.147561	2.636197
H	4.919151	9.733400	1.899274
C	6.092721	9.332935	3.646433
H	5.802111	8.288162	3.693965
C	6.947555	9.862550	4.605973
H	7.312620	9.223114	5.403880
C	7.332956	11.201568	4.566203
C	5.363263	12.378654	1.473318
H	5.839584	13.361920	1.543522
C	5.641657	11.840077	0.064915
H	6.714895	11.710455	-0.105022
H	5.257837	12.532627	-0.691052
H	5.158083	10.871230	-0.098384
C	3.857680	12.573788	1.706197
H	3.322661	11.621206	1.628965
H	3.435694	13.259422	0.964012
H	3.658198	12.981438	2.703006
C	8.199557	11.797517	5.665086
H	8.660242	12.708116	5.270775
C	7.323492	12.208463	6.859075
H	6.516320	12.885387	6.557026
H	7.920886	12.707859	7.629235
H	6.850108	11.329014	7.308515
C	9.334847	10.872201	6.114250
H	8.958657	9.983725	6.631888
H	9.994447	11.397563	6.811826
H	9.935229	10.536257	5.263154
C	3.995947	13.918572	5.389572
H	3.862595	12.851362	5.143560
H	4.147263	13.928180	6.483135
C	3.878226	16.315205	1.825160
H	4.063135	15.533468	2.569854
H	3.800771	15.829622	0.846837
H	2.908312	16.761224	2.069793
C	4.744594	18.403829	0.709499
H	3.753141	18.863113	0.779748
H	4.804680	17.913813	-0.267100

H	5.489960	19.204359	0.737120
C	0.167469	14.801585	5.455314
C	1.460632	14.039598	5.763390
C	2.690249	14.669391	5.088588
C	2.783399	16.150260	5.479059
C	1.501109	16.926850	5.176415
C	0.285100	16.278523	5.843203
H	1.629569	14.029557	6.850675
H	1.368867	12.991841	5.452743
H	-0.043468	14.730315	4.378697
H	-0.681772	14.339264	5.971802
H	3.002842	16.213978	6.555936
H	3.626585	16.624839	4.966900
H	1.608952	17.969739	5.495671
H	1.345781	16.947837	4.087689
H	0.393199	16.354565	6.934285
H	-0.631515	16.818296	5.578539
H	2.499363	14.636235	4.001139

5a.log

SCF (wB97x) = -1622.79044855
E(SCF)+ZPE(0 K)= -1622.046223
H(298 K)= -1622.004582
G(298 K)= -1622.120208
Lowest Frequency = 11.4574cm-1

Zn	0.309053	0.009222	-0.195395
N	0.382391	1.935632	0.256184
C	-0.446337	4.123789	0.961447
H	0.289039	4.224640	1.765548
H	-1.376585	4.601123	1.270208
H	-0.042673	4.660341	0.097079
N	-1.620264	-0.163823	0.184002
C	-0.659285	2.664899	0.626879
C	-1.968043	2.161777	0.729255
H	-2.727615	2.876946	1.018394
C	-2.412413	0.845561	0.525097
C	-3.888366	0.576476	0.715540
H	-4.429059	1.489609	0.965019
H	-4.047742	-0.156923	1.512050
H	-4.316195	0.148469	-0.196099
C	1.683663	2.515663	0.184049
C	2.549093	2.397550	1.285706
C	3.849271	2.889827	1.164533
H	4.534177	2.800668	2.003180
C	4.284999	3.480709	-0.013492
H	5.300516	3.856229	-0.091601
C	3.419616	3.582937	-1.095764
H	3.771690	4.035417	-2.017990
C	2.111713	3.104984	-1.020131
C	2.108095	1.702996	2.564948
H	1.027873	1.541779	2.501977
C	2.772779	0.323364	2.678826
H	3.860539	0.420803	2.764728
H	2.565049	-0.287250	1.794467
H	2.407620	-0.213378	3.560719
C	2.371660	2.550049	3.815141
H	3.442909	2.701070	3.984663
H	1.965995	2.053385	4.702293
H	1.904446	3.536285	3.732887
C	1.192227	3.160596	-2.230605
H	0.165072	3.034808	-1.876118
C	1.256304	4.498716	-2.974369
H	1.084446	5.340521	-2.296379
H	0.493434	4.530145	-3.758529
H	2.225989	4.651314	-3.459523
C	1.493336	1.988753	-3.177203
H	2.513097	2.063638	-3.570244
H	0.799316	1.983453	-4.024415
H	1.410256	1.028103	-2.659006
C	-2.149496	-1.483277	0.054736
C	-2.190791	-2.319429	1.187322
C	-2.595295	-3.642660	1.017725
H	-2.625792	-4.307878	1.874981
C	-2.956662	-4.129255	-0.233105
H	-3.261635	-5.164796	-0.346265
C	-2.931459	-3.285954	-1.334440
H	-3.219720	-3.669775	-2.309184
C	-2.533586	-1.953280	-1.212410
C	-1.771152	-1.798156	2.554793
H	-1.916788	-0.713570	2.557447
C	-2.612997	-2.368514	3.701003
H	-2.416494	-3.433533	3.862863
H	-3.683787	-2.248746	3.509492
H	-2.372211	-1.851134	4.634774
C	-0.277605	-2.049523	2.800816
H	-0.056943	-3.122205	2.784865
H	0.028550	-1.647648	3.772558
H	0.337998	-1.579691	2.029117
C	-2.496317	-1.052787	-2.437158
H	-2.287982	-0.034855	-2.094835
C	-3.841160	-1.023419	-3.173911
H	-4.087573	-1.999942	-3.603847
H	-3.808648	-0.300827	-3.995538
H	-4.657806	-0.740033	-2.502708
C	-1.358574	-1.462134	-3.382345
H	-0.394417	-1.440359	-2.866790
H	-1.303792	-0.784724	-4.240961
H	-1.510958	-2.478715	-3.761460
H	2.129835	-2.487232	0.909901
C	1.739211	-1.180172	-0.823225
C	1.802525	-2.526920	-0.128424
C	2.212624	-3.774549	-0.869485
C	0.780593	-3.587375	-0.428019
H	1.592590	-1.346733	-1.899913
H	2.813814	-4.518997	-0.355584
H	2.432035	-3.675910	-1.929226
H	0.403419	-4.200619	0.384405
H	0.035665	-3.362543	-1.186899
H	2.706231	-0.666444	-0.737253

5b.log

SCF (wB97x) = -1662.10368398
E(SCF)+ZPE(0 K)= -1661.329203
H(298 K)= -1661.286873
G(298 K)= -1661.403336
Lowest Frequency = 17.0553cm-1

Zn	0.254160	-0.025913	-0.252234
N	0.364545	1.901950	0.217404
C	-0.425781	4.100733	0.936646
H	0.315997	4.182059	1.736808
H	-1.346850	4.588765	1.256058
H	-0.019633	4.640586	0.075462

N	-1.666462	-0.169867	0.157104
C	-0.662779	2.648778	0.587959
C	-1.981789	2.167744	0.679492
H	-2.731624	2.895765	0.961890
C	-2.442747	0.856267	0.491287
C	-3.920176	0.607632	0.698549
H	-4.451025	1.535024	0.914311
H	-4.081568	-0.090916	1.525525
H	-4.357056	0.146693	-0.192478
C	1.680886	2.448943	0.174780
C	2.534215	2.263570	1.276428
C	3.847466	2.727411	1.184993
H	4.521745	2.588792	2.025786
C	4.308719	3.352161	0.034923
H	5.334019	3.704366	-0.020523
C	3.455682	3.517811	-1.049510
H	3.827622	3.995463	-1.950947
C	2.135332	3.072043	-1.002722
C	2.068007	1.539627	2.530472
H	0.997558	1.339890	2.423393
C	2.777844	0.185429	2.668088
H	3.857753	0.318990	2.793994
H	2.621505	-0.432421	1.778542
H	2.400884	-0.363457	3.537344
C	2.250022	2.391664	3.792409
H	3.307412	2.594894	3.992290
H	1.842730	1.871578	4.665403
H	1.737436	3.354114	3.699622
C	1.229801	3.196323	-2.218583
H	0.195357	3.094460	-1.877970
C	1.345849	4.553409	-2.920491
H	1.198653	5.379977	-2.218293
H	0.590343	4.635229	-3.708099
H	2.323738	4.686532	-3.394769
C	1.500563	2.044096	-3.197595
H	2.527191	2.094216	-3.576428
H	0.817369	2.089792	-4.052343
H	1.376761	1.072460	-2.709395
C	-2.215202	-1.485750	0.083497
C	-2.264476	-2.273181	1.250643
C	-2.707196	-3.590707	1.141693
H	-2.748460	-4.218231	2.026328
C	-3.083476	-4.122035	-0.086634
H	-3.413449	-5.153878	-0.154106
C	-3.038333	-3.329356	-1.224158
H	-3.336214	-3.749377	-2.180765
C	-2.615800	-2.000407	-1.160486
C	-1.790659	-1.714058	2.584721
H	-1.911533	-0.626844	2.555328
C	-2.600628	-2.226708	3.779627
H	-2.421082	-3.289917	3.970412
H	-3.674940	-2.088785	3.623491
H	-2.314148	-1.684032	4.685664
C	-0.294213	-1.994589	2.782672
H	-0.100346	-3.072614	2.799086
H	0.061007	-1.565181	3.725409
H	0.304057	-1.568063	1.971823
C	-2.579404	-1.146011	-2.418012
H	-2.391783	-0.113068	-2.110327
C	-3.916977	-1.164102	-3.168164
H	-4.139674	-2.155678	-3.575895
H	-3.889991	-0.462586	-4.007994
H	-4.745714	-0.879799	-2.512308
C	-1.425583	-1.568153	-3.337565
H	-0.463895	-1.491551	-2.822346
H	-1.388223	-0.933614	-4.229283
H	-1.546641	-2.606947	-3.664481
H	2.018733	-2.486639	0.787575
C	1.742360	-1.137869	-0.883997
C	1.845842	-2.537138	-0.296503
C	2.844427	-3.562585	-0.900681
C	1.793213	-4.668104	-0.655864
C	0.717528	-3.561736	-0.570050
H	1.674549	-1.223976	-1.978787
H	3.826845	-3.665958	-0.430426
H	2.984619	-3.375406	-1.971505
H	1.942791	-5.157091	0.311996
H	1.673640	-5.439126	-1.421611
H	-0.079769	-3.665024	0.170239
H	0.256555	-3.377631	-1.546741
H	2.668145	-0.576921	-0.689808

5c.log

SCF (wB97x) = -1701.43829697
E(SCF)+ZPE(0 K)= -1700.634139
H(298 K)= -1700.590591
G(298 K)= -1700.710408
Lowest Frequency = 13.4972cm-1

Zn	6.028963	14.947147	4.348040
N	7.159550	16.439086	3.733111
N	7.199883	13.544532	3.561121
C	8.332938	16.295191	3.125047
C	8.889667	15.059714	2.763497
H	9.848350	15.102056	2.262275
C	8.342283	13.774792	2.935456
C	9.125908	17.537026	2.783902
H	8.568761	18.171298	2.087327
H	10.086363	17.282697	2.335232
H	9.300767	18.135099	3.683456
C	9.116473	12.606683	2.367873
H	9.404008	11.913880	3.165034
H	10.015748	12.939536	1.849110
H	8.491798	12.041045	1.670118
C	6.635416	17.744011	3.977556
C	6.815178	18.336385	5.238512
C	6.174748	19.548755	5.499949
H	6.286824	20.011796	6.476240
C	5.391347	20.167718	4.536696
H	4.895608	21.107468	4.758576
C	5.243360	19.581631	3.285010
H	4.629092	20.074017	2.537902
C	5.856065	18.366203	2.982323
C	7.654064	17.665090	6.314172
H	8.193109	16.836660	5.845291
C	6.759368	17.069925	7.410210
H	6.184595	17.855494	7.913183
H	7.361379	16.552514	8.164601
H	6.045621	16.355281	6.990351
C	8.701850	18.613049	6.909902
H	9.331648	19.051588	6.129616
H	9.349117	18.071923	7.607304
H	8.236964	19.434860	7.464469
C	5.631788	17.692339	1.636150
H	6.484649	17.034539	1.442496

C	6.689307	12.218097	3.675917
C	5.735639	11.769310	2.745737
C	5.178995	10.501817	2.924274
H	4.437711	10.140525	2.216588
C	5.548268	9.700826	3.995549
H	5.103200	8.718718	4.121041
C	6.484139	10.163800	4.912588
H	6.758318	9.536764	5.755603
C	7.068082	11.422261	4.773519
C	5.278399	12.644364	1.588380
H	5.887936	13.552950	1.593410
C	5.487046	11.961940	0.230924
H	6.531724	11.667902	0.089990
H	5.211299	12.639991	-0.583160
H	4.871315	11.061425	0.134405
C	3.815582	13.070436	1.775934
H	3.148045	12.202070	1.760437
H	3.504583	13.750576	0.976115
H	3.674585	13.580591	2.733517
C	8.039941	11.948143	5.818962
H	8.607428	12.766930	5.367213
C	7.267368	12.535017	7.009810
H	6.574876	13.317991	6.685591
H	7.954153	12.965977	7.746042
H	6.676585	11.757423	7.506084
C	9.051886	10.896125	6.284712
H	8.573746	10.092540	6.854448
H	9.798693	11.356536	6.939020
H	9.573822	10.441276	5.436979
C	4.366195	14.594007	5.329279
H	4.110993	13.542487	5.132440
H	4.570281	14.645101	6.409806
C	1.919085	17.551978	5.316369
H	1.720257	17.944621	4.312948
H	1.788259	18.386854	6.010742
C	0.964105	16.363967	5.628305
H	0.356995	16.546125	6.519865
H	0.265224	16.209536	4.799827
C	1.878503	15.134580	5.799572
H	1.416699	14.203504	5.455630
H	2.138382	14.995372	6.857638
C	4.379909	16.804706	1.684715
H	4.467056	16.031182	2.453774
H	4.218321	16.305271	0.723612
H	3.490852	17.400118	1.919720
C	5.549512	18.681570	0.469306
H	4.636685	19.285160	0.507435
H	5.536964	18.138408	-0.480573
H	6.405068	19.363823	0.462197
C	3.160521	15.484165	5.026406
H	2.918889	15.423231	3.952928
C	3.338746	16.966423	5.374889
H	4.036843	17.485346	4.714123
H	3.743176	17.041428	6.393679

5d.log

SCF (wB97x) = -1740.75551970
E(SCF)+ZPE(0 K)= -1739.921186
H(298 K)= -1739.877084
G(298 K)= -1739.996845
Lowest Frequency = 16.9919cm-1

Zn	5.743374	14.605449	4.367890
N	6.671634	16.269333	3.858432
N	7.100023	13.421809	3.519981
C	7.857260	16.319607	3.259469
C	8.576736	15.193184	2.834970
H	9.524001	15.392545	2.350160
C	8.204938	13.838820	2.924487
C	8.477277	17.674630	3.001541
H	7.842210	18.269191	2.337357
H	9.464875	17.579035	2.549887
H	8.566746	18.235340	3.937031
C	9.131799	12.820144	2.300525
H	9.508349	12.128173	3.060466
H	9.979232	13.300455	1.810872
H	8.591497	12.216050	1.565598
C	5.971879	17.474088	4.168114
C	6.059644	18.011142	5.463219
C	5.241936	19.094026	5.789938
H	5.281754	19.509292	6.792938
C	4.369547	19.639504	4.858998
H	3.733959	20.475942	5.132200
C	4.312703	19.113356	3.573695
H	3.627209	19.547132	2.852656
C	5.107796	18.029418	3.203861
C	6.978754	17.401137	6.508893
H	7.635009	16.690041	5.998436
C	6.167772	16.614863	7.548601
H	5.488309	17.278439	8.094850
H	6.830534	16.132927	8.275013
H	5.559449	15.841411	7.070729
C	7.873046	18.449303	7.181431
H	8.442143	19.020823	6.441634
H	8.583327	17.963509	7.857914
H	7.288900	19.160351	7.775005
C	4.990025	17.412495	1.817655
H	5.928499	16.893958	1.598804
C	6.771469	12.034621	3.552097
C	5.889204	11.517645	2.587546
C	5.507844	10.178539	2.686289
H	4.824016	9.763450	1.950775
C	5.978411	9.372635	3.713229
H	5.669384	8.333965	3.776583
C	6.840164	9.902422	4.666103
H	7.193362	9.269541	5.474636
C	7.249546	11.233881	4.606522
C	5.321847	12.389564	1.477544
H	5.794827	13.373630	1.549073
C	5.640108	11.829894	0.085572
H	6.717729	11.699366	-0.053627
H	5.276255	12.509876	-0.691461
H	5.162948	10.857353	-0.075257
C	3.811235	12.590071	1.662747
H	3.274894	11.638411	1.581776
H	3.414227	13.267692	0.899716
H	3.588847	13.014293	2.646410
C	8.136196	11.824222	5.692421
H	8.589879	12.736787	5.295057
C	7.285131	12.232192	6.904333
H	6.494649	12.931888	6.615738
H	7.903399	12.708955	7.672381
H	6.802420	11.354910	7.348553
C	9.278517	10.893004	6.111318
H	8.910456	10.001342	6.629415

H	9.953112	11.411660	6.799595
H	9.861243	10.561552	5.246143
C	4.136185	13.956693	5.292177
H	4.043819	12.892775	5.027636
H	4.321279	13.966886	6.377436
C	3.875645	16.356888	1.795278
H	4.063850	15.560456	2.521799
H	3.794531	15.895391	0.805483
H	2.909584	16.807259	2.048112
C	4.777073	18.449538	0.710285
H	3.788770	18.916963	0.771295
H	4.846052	17.968711	-0.270303
H	5.528968	19.243272	0.756057
C	0.288692	14.659348	5.427606
C	1.621606	13.951812	5.692372
C	2.804489	14.670350	5.024517
C	2.834547	16.137137	5.468809
C	1.511098	16.856630	5.206676
C	0.338080	16.126202	5.865217
H	1.804637	13.910552	6.776605
H	1.576078	12.912970	5.343448
H	0.066668	14.614005	4.351985
H	-0.527984	14.136597	5.938480
H	3.062224	16.173238	6.545067
H	3.646419	16.666880	4.964119
H	1.571598	17.892165	5.559985
H	1.341599	16.906244	4.121195
H	0.456717	16.170525	6.956930
H	-0.608028	16.626963	5.629939
H	2.604649	14.664064	3.940474

6a.log

SCF (wB97x) = -1396.50917707
E(SCF)+ZPE(0 K)= -1395.766269
H(298 K)= -1395.724170
G(298 K)= -1395.841164
Lowest Frequency = 14.0665cm-1

N	-2.678086	18.613037	3.734096
N	-1.960732	18.378729	6.545712
C	-1.933159	18.685891	7.940238
C	-1.878008	17.562280	3.629368
C	-0.421738	16.543731	7.043919
H	0.302415	17.183925	7.557693
H	0.113967	15.741904	6.535617
H	-1.060122	16.107682	7.818891
C	-2.902894	18.121633	8.790257
C	-1.656575	16.946871	2.266484
H	-2.609789	16.620317	1.838435
H	-0.980490	16.093084	2.315701
H	-1.240958	17.689317	1.577681
C	-1.212640	16.977521	4.722847
H	-0.588618	16.123313	4.491955
C	-2.927440	18.518739	10.127232
H	-3.673306	18.100269	10.796554
C	-4.618893	18.741492	2.252714
C	-1.250202	17.358790	6.076054
C	-3.320019	19.167080	2.587012
C	-4.706260	20.475142	0.561411
H	-5.251610	20.993166	-0.221117
C	-5.296451	19.413951	1.235490
H	-6.305381	19.110495	0.972168
C	-1.005265	19.628378	8.421083
C	-2.700257	20.226303	1.898358
C	-1.302071	20.690995	2.279289
H	-0.832112	19.889128	2.856532
C	-3.935542	17.143050	8.251709
H	-3.565416	16.763096	7.294792
C	-4.390025	21.267465	5.705585
H	-5.194977	21.019313	6.416035
H	-3.837346	22.086321	6.193396
C	0.007926	20.272135	7.486792
H	0.056295	19.662774	6.579364
C	-1.066958	19.996514	9.765353
H	-0.364475	20.728694	10.152690
C	-3.415447	20.869919	0.888439
H	-2.960764	21.699196	0.354676
C	-5.288318	17.608228	3.016385
H	-4.504438	17.054285	3.541516
C	-6.249945	18.158396	4.080089
H	-5.745115	18.837189	4.776703
H	-6.693655	17.344043	4.662316
H	-7.061456	18.726397	3.612512
C	-4.150373	15.933789	9.168215
H	-3.203688	15.433833	9.394813
H	-4.813159	15.208397	8.686192
H	-4.615437	16.217937	10.117833
C	-5.262822	17.866127	7.977108
H	-5.691089	18.253383	8.907892
H	-5.991438	17.187803	7.520996
H	-5.127967	18.722128	7.306435
C	-2.018448	19.448457	10.615569
H	-2.054272	19.748870	11.658024
C	-0.411329	20.953534	1.059827
H	-0.390174	20.088519	0.389996
H	0.613930	21.163762	1.380011
H	-0.754496	21.817481	0.481354
C	1.420820	20.318708	8.078697
H	1.479231	20.987108	8.943933
H	2.130635	20.689398	7.332618
H	1.749914	19.325617	8.399646
C	-5.940741	22.992152	4.535128
H	-6.370699	23.342514	3.594501
C	-5.022134	21.801320	4.412742
H	-4.242687	22.067388	3.683298
H	-5.587641	21.007235	3.902805
C	-1.364412	21.929544	3.185242
H	-1.828667	22.771219	2.660042
H	-0.360330	22.233571	3.498996
H	-1.960277	21.748169	4.086925
C	-6.261318	23.631753	5.656937
H	-5.861343	23.323992	6.618953
H	-6.934140	24.484084	5.650067
C	-0.462047	21.675430	7.074850
H	-1.464522	21.653912	6.632973
H	0.222760	22.119003	6.344544
H	-0.509828	22.338054	7.945713
C	-6.005946	16.615258	2.095271
H	-6.876001	17.066366	1.607169
H	-6.365625	15.757681	2.672401
H	-5.336949	16.244824	1.312563
Mg	-3.131240	19.603595	5.437358

6b.log

SCF (wB97x) = -1435.81449796
E(SCF)+ZPE(0 K)= -1435.043320
H(298 K)= -1434.999690
G(298 K)= -1435.121099
Lowest Frequency = 10.0854cm-1

Mg	-2.993080	19.262786	5.495223
N	-2.753317	18.298503	3.730449
N	-1.888957	17.886059	6.483400
C	-1.729519	18.149491	7.877477
C	-2.090862	17.165483	3.548280
C	-0.498487	15.900901	6.817772
H	0.309639	16.460801	7.298904
H	-0.067704	15.078028	6.247134
H	-1.115969	15.488260	7.621955
C	-2.652908	17.610919	8.794510
C	-2.033416	16.569650	2.159969
H	-3.045221	16.375581	1.789877
H	-1.466716	15.638458	2.145309
H	-1.573615	17.275723	1.461140
C	-1.423003	16.475455	4.577619
H	-0.914334	15.566757	4.281119
C	-2.539377	17.966981	10.137941
H	-3.244700	17.566833	10.859991
C	-4.732571	18.728865	2.355978
C	-1.318257	16.817718	5.937347
C	-3.374220	18.967121	2.632847
C	-4.668935	20.512408	0.716184
H	-5.177617	21.124072	-0.022232
C	-5.361981	19.517427	1.392290
H	-6.415441	19.362677	1.179423
C	-0.718239	19.031694	8.298863
C	-2.652790	19.958172	1.940728
C	-1.188699	20.221475	2.262191
H	-0.794073	19.334321	2.766392
C	-3.782974	16.710399	8.318212
H	-3.492227	16.296147	7.348293
C	-4.157705	20.962137	5.924399
H	-5.052286	20.603755	6.460027
H	-3.681209	21.660804	6.628525
C	0.243268	19.656641	7.299578
H	0.135406	19.116353	6.353990
C	-0.643147	19.361522	9.652842
H	0.127147	20.047290	9.994231
C	-3.323030	20.723543	0.987108
H	-2.788494	21.503241	0.452828
C	-5.520091	17.685170	3.132988
H	-4.799884	17.025452	3.626896
C	-6.362792	18.353880	4.229255
H	-5.742208	18.933906	4.920265
H	-6.910446	17.604235	4.810428
H	-7.084939	19.055111	3.798486
C	-4.049768	15.528313	9.255827
H	-3.134060	14.962874	9.454127
H	-4.780440	14.849072	8.805952
H	-4.459185	15.853128	10.217987
C	-5.061923	17.532842	8.098467
H	-5.409319	17.964568	9.043257
H	-5.863560	16.908240	7.690728
H	-4.895612	18.365883	7.406682
C	-1.542890	18.834413	10.568902
H	-1.472175	19.102956	11.618253
C	-0.333851	20.447751	1.010335
H	-0.460719	19.633287	0.290765
H	0.724934	20.502738	1.282061
H	-0.588071	21.385055	0.504747
C	1.708727	19.530554	7.730875
H	1.916368	20.106776	8.638503
H	2.369376	19.909032	6.944513
H	1.975360	18.487807	7.928377
C	-5.755468	22.730315	4.861557
H	-5.859283	23.357357	3.966795
C	-4.592019	21.735525	4.665733
H	-3.733375	22.295267	4.272634
H	-4.887579	21.052955	3.858221
C	-1.047044	21.399786	3.236419
H	-1.431824	22.321699	2.787378
H	0.002215	21.560224	3.505832
H	-1.611880	21.236868	4.161341
C	-7.062122	22.040961	5.127887
H	-7.134228	21.485764	6.062968
C	-0.131818	21.123859	7.041056
H	-1.173858	21.223623	6.715840
H	0.510623	21.561193	6.269615
H	-0.021431	21.718560	7.954325
C	-6.393661	16.808688	2.228427
H	-7.208272	17.379915	1.771484
H	-6.849446	16.002145	2.811252
H	-5.806073	16.358576	1.422286
C	-8.104227	22.034570	4.299043
H	-9.018811	21.498010	4.534239
H	-8.079520	22.574025	3.354622
H	-5.513212	23.396206	5.701483

6c.log

SCF (wB97x) = -1475.12131661
E(SCF)+ZPE(0 K)= -1474.320631
H(298 K)= -1474.276113
G(298 K)= -1474.398412
Lowest Frequency = 12.5080cm-1

Mg	-3.013778	19.347600	5.402265
N	-2.784549	18.412260	3.621652
N	-1.886102	17.963303	6.355907
C	-1.711969	18.203740	7.752447
C	-2.115581	17.288310	3.411826
C	-0.484989	15.978513	6.644030
H	0.329764	16.531553	7.122004
H	-0.061993	15.163021	6.057200
H	-1.092454	15.555925	7.450657
C	-2.633661	17.661519	8.668608
C	-2.071922	16.715573	2.013341
H	-3.086877	16.516289	1.654735
H	-1.495525	15.791004	1.975333
H	-1.630020	17.437875	1.319550
C	-1.430618	16.586913	4.422033
H	-0.918962	15.686696	4.105433
C	-2.505972	17.995983	10.016501
H	-3.210779	17.593087	10.737665
C	-4.796472	18.836385	2.296768
C	-1.315807	16.906898	5.786355
C	-3.435746	19.090130	2.548120
C	-4.791786	20.645311	0.684088
H	-5.325201	21.260737	-0.033726
C	-5.456383	19.629293	1.358030

H	-6.512067	19.464489	1.165998
C	-0.686445	19.067047	8.179355
C	-2.741954	20.100235	1.855660
C	-1.271851	20.370671	2.142832
H	-0.857417	19.477427	2.619735
C	-3.776815	16.779411	8.189534
H	-3.508573	16.395213	7.200975
C	-4.148714	21.058419	5.863550
H	-5.013047	20.734562	6.467386
H	-3.608799	21.763069	6.515739
C	0.273601	19.695636	7.180962
H	0.164951	19.158648	6.233734
C	-0.597019	19.375071	9.537420
H	0.184066	20.046100	9.883356
C	-3.443521	20.871729	0.929606
H	-2.931293	21.666431	0.395562
C	-5.547776	17.763520	3.070744
H	-4.805069	17.093071	3.514295
C	-6.353648	18.388212	4.219452
H	-5.714104	18.954581	4.905321
H	-6.869700	17.614017	4.797356
H	-7.097118	19.094014	3.835219
C	-4.024631	15.568920	9.095745
H	-3.106771	14.993545	9.250825
H	-4.770665	14.907436	8.644512
H	-4.405703	15.864602	10.078722
C	-5.057692	17.611559	8.024832
H	-5.384720	18.010782	8.990998
H	-5.869317	17.003360	7.612055
H	-4.902519	18.467981	7.359432
C	-1.496265	18.844982	10.452605
H	-1.414261	19.096918	11.505263
C	-0.453923	20.625453	0.871844
H	-0.596096	19.823665	0.140963
H	0.611741	20.682509	1.114749
H	-0.728072	21.570282	0.391228
C	1.740061	19.571104	7.609012
H	1.950310	20.149255	8.514803
H	2.398236	19.948719	6.820188
H	2.008037	18.528880	7.807591
C	-5.644094	22.956410	4.909951
H	-5.740950	23.608212	4.031126
C	-4.651476	21.816470	4.620942
H	-3.794184	22.244551	4.085586
H	-5.114788	21.123046	3.907909
C	-1.108854	21.531652	3.134206
H	-1.514483	22.458855	2.715347
H	-0.052641	21.695560	3.372565
H	-1.641335	21.345866	4.073732
C	-7.037242	22.486126	5.334563
H	-6.943208	21.737963	6.133833
C	-0.104183	21.162955	6.927229
H	-1.147091	21.262203	6.605000
H	0.535508	21.603524	6.155336
H	0.007336	21.754971	7.842118
C	-6.451511	16.910128	2.173882
H	-7.290571	17.488400	1.773506
H	-6.874343	16.079126	2.747099
H	-5.895511	16.494602	1.327775
C	-7.916021	21.914061	4.252885
H	-8.875299	21.530807	4.603456
H	-5.229457	23.581083	5.711104
C	-7.655765	21.851264	2.948508
H	-7.585332	23.320987	5.793678
H	-8.379319	21.433972	2.254055
H	-6.718710	22.198347	2.523805

6d.log

SCF (wB97x) = -1514.42781912
E(SCF)+ZPE(0 K)= -1513.597989
H(298 K)= -1513.552251
G(298 K)= -1513.677167
Lowest Frequency = 14.2344cm-1

Mg	-3.128438	19.289700	5.234071
N	-2.825929	18.333310	3.476743
N	-1.998792	17.944298	6.240704
C	-1.875509	18.208236	7.638525
C	-2.126907	17.220862	3.303446
C	-0.564389	15.995839	6.601643
H	0.226395	16.572056	7.092391
H	-0.110070	15.178467	6.041440
H	-1.186928	15.576220	7.398258
C	-2.823134	17.669891	8.529994
C	-2.039982	16.622158	1.917895
H	-3.041575	16.378826	1.548872
H	-1.431082	15.717938	1.908273
H	-1.613568	17.344910	1.215068
C	-1.452585	16.552582	4.342213
H	-0.911451	15.660018	4.054289
C	-2.748811	18.029268	9.875545
H	-3.474558	17.630017	10.577710
C	-4.823948	18.663866	2.105811
C	-1.387386	16.893757	5.705031
C	-3.477868	18.967771	2.377806
C	-4.843730	20.427457	0.443444
H	-5.381227	21.006009	-0.301022
C	-5.487554	19.405368	1.127935
H	-6.528473	19.187999	0.905619
C	-0.874661	19.089422	8.087213
C	-2.801977	19.980139	1.670467
C	-1.343131	20.294991	1.971065
H	-0.909036	19.416306	2.457565
C	-3.935531	16.762357	8.026347
H	-3.626059	16.364751	7.055216
C	-4.278258	20.997846	5.661413
H	-5.152913	20.693551	6.259935
H	-3.728656	21.690408	6.320156
C	0.115809	19.709435	7.113364
H	0.056252	19.147060	6.176643
C	-0.838714	19.421902	9.441893
H	-0.078064	20.107310	9.804581
C	-3.509327	20.704995	0.711763
H	-3.012262	21.501903	0.167077
C	-5.558672	17.587999	2.891001
H	-4.808671	16.992017	3.419502
C	-6.472791	18.220377	3.950844
H	-5.918019	18.895610	4.612275
H	-6.946556	17.450072	4.568362
H	-7.265919	18.810734	3.478475
C	-4.197054	15.564068	8.944965
H	-3.276598	15.006297	9.142824
H	-4.916296	14.882958	8.479646
H	-4.617534	15.870841	9.908251
C	-5.222746	17.570408	7.801631

H	-5.589339	17.982388	8.747998
H	-6.010165	16.941048	7.373780
H	-5.058860	18.417229	7.125988
C	-1.765698	18.897964	10.332762
H	-1.725402	19.169486	11.382960
C	-0.519851	20.564700	0.706590
H	-0.636098	19.757557	-0.022986
H	0.541727	20.647622	0.959494
H	-0.811311	21.501566	0.220627
C	1.565112	19.622486	7.604508
H	1.728873	20.230106	8.500544
H	2.248901	19.988732	6.832312
H	1.841604	18.591223	7.844409
C	-5.560122	23.053102	4.711820
H	-5.842261	23.520398	3.759327
C	-4.762072	21.771141	4.421857
H	-3.906639	22.057447	3.796636
H	-5.361493	21.114559	3.775969
C	-1.219144	21.465811	2.956417
H	-1.650408	22.378333	2.531198
H	-0.168967	21.661762	3.197043
H	-1.747621	21.269463	3.895924
C	-6.818237	22.851826	5.561801
H	-6.534461	22.488831	6.556070
C	-0.275237	21.162743	6.805313
H	-1.305106	21.235282	6.437742
H	0.388814	21.594481	6.049167
H	-0.212228	21.779945	7.708080
C	-6.346433	16.631316	1.988947
H	-7.179082	17.136336	1.488562
H	-6.769814	15.814751	2.582182
H	-5.705558	16.195957	1.216072
C	-7.832077	21.867411	4.953498
H	-7.362547	20.885538	4.830403
H	-4.904751	23.765139	5.231204
C	-8.376264	22.337671	3.634960
H	-7.307910	23.822462	5.715429
H	-9.023157	23.215506	3.673734
C	-8.096879	21.794625	2.452921
H	-8.661507	21.734340	5.659920
H	-7.432259	20.938355	2.368873
H	-8.504336	22.194357	1.528999

7a.log

SCF (wB97x) = -1622.79186344
E(SCF)+ZPE(0 K)= -1622.048620
H(298 K)= -1622.006357
G(298 K)= -1622.124307
Lowest Frequency = 6.9653cm-1

N	-2.701069	18.608350	3.729904
N	-2.014881	18.402775	6.532031
C	-1.991582	18.753141	7.915996
C	-1.906487	17.553268	3.639102
C	-0.457419	16.599680	7.071604
H	0.251973	17.260659	7.578834
H	0.095130	15.800078	6.577913
H	-1.092407	16.163643	7.849081
C	-2.934760	18.179869	8.788939
C	-1.682378	16.927957	2.281554
H	-2.636091	16.609409	1.848913
H	-1.017207	16.066410	2.343782
H	-1.251202	17.659240	1.590616
C	-1.250252	16.977177	4.740325
H	-0.625390	16.119950	4.524090
C	-2.962928	18.616418	10.113352
H	-3.690008	18.193355	10.800355
C	-4.619149	18.773555	2.225849
C	-1.292559	17.383024	6.084463
C	-3.317949	19.174567	2.576407
C	-4.652977	20.516267	0.541731
H	-5.179214	21.049361	-0.243843
C	-5.271536	19.463877	1.203713
H	-6.283543	19.181200	0.928528
C	-1.089716	19.736500	8.360487
C	-2.663913	20.217219	1.894565
C	-1.253145	20.639687	2.280246
H	-0.803052	19.818444	2.845673
C	-3.934003	17.144674	8.294833
H	-3.574883	16.766228	7.333474
C	-4.348567	21.075340	5.719018
H	-5.185278	20.737638	6.346861
H	-3.822093	21.823162	6.327588
C	-0.088734	20.378665	7.411981
H	-0.101302	19.809284	6.478083
C	-1.154520	20.141797	9.694111
H	-0.472042	20.907145	10.053067
C	-3.355762	20.879975	0.881037
H	-2.877061	21.698903	0.352389
C	-5.321008	17.646757	2.969179
H	-4.564094	17.108521	3.547176
C	-6.347034	18.201224	3.968058
H	-5.876855	18.870630	4.695207
H	-6.830145	17.387333	4.518779
H	-7.125738	18.771567	3.449891
C	-4.060651	15.943689	9.238846
H	-3.084353	15.494749	9.446546
H	-4.700652	15.177419	8.790311
H	-4.510722	16.223386	10.197031
C	-5.301959	17.796104	8.045281
H	-5.715456	18.197307	8.976979
H	-6.012735	17.067395	7.641532
H	-5.222069	18.625028	7.335485
C	-2.082594	19.590457	10.567067
H	-2.121774	19.920739	11.600378
C	-0.359200	20.894309	1.061307
H	-0.368614	20.041550	0.375553
H	0.673432	21.065305	1.381139
H	-0.675684	21.779559	0.499966
C	1.343884	20.332475	7.956796
H	1.453792	20.941576	8.860126
H	2.045683	20.720710	7.211843
H	1.642661	19.309616	8.206237
C	-5.843520	22.898857	4.671410
H	-6.229677	23.353461	3.757611
C	-4.903596	21.740918	4.455025
H	-4.083791	22.094616	3.814979
H	-5.424058	21.003719	3.828302
C	-1.275663	21.864393	3.205765
H	-1.739520	22.721357	2.705149
H	-0.259173	22.148248	3.497949
H	-1.845718	21.667366	4.119404
C	-6.231575	23.398283	5.842011
H	-5.880118	22.987501	6.784247
H	-6.916378	24.238954	5.897298

C	-0.505856	21.817733	7.077240
H	-1.514384	21.848382	6.653858
H	0.183014	22.262996	6.351746
H	-0.504617	22.443550	7.976337
C	-5.972221	16.636184	2.017719
H	-6.808760	17.077242	1.465812
H	-6.366548	15.785457	2.582319
H	-5.251987	16.257128	1.286048
Zn	-3.156309	19.550449	5.410071

7b.log

SCF (wB97x) = -1662.09710684
E(SCF)+ZPE(0 K)= -1661.324700
H(298 K)= -1661.281190
G(298 K)= -1661.401838
Lowest Frequency = 8.0362cm-1

Zn	-3.084182	19.161186	5.476538
N	-2.806353	18.271120	3.728801
N	-1.956827	17.891122	6.472090
C	-1.782532	18.200519	7.854524
C	-2.130750	17.145925	3.553032
C	-0.507720	15.956078	6.830645
H	0.280603	16.547960	7.305400
H	-0.049448	15.143314	6.266885
H	-1.110561	15.529722	7.638594
C	-2.658230	17.641925	8.804748
C	-2.055029	16.553836	2.164333
H	-3.061708	16.376991	1.773182
H	-1.502940	15.613724	2.161201
H	-1.567640	17.252071	1.476506
C	-1.462803	16.467454	4.587383
H	-0.940935	15.563439	4.300178
C	-2.524925	18.030222	10.137367
H	-3.195017	17.616456	10.884993
C	-4.748035	18.756596	2.321628
C	-1.358312	16.832826	5.939520
C	-3.391054	18.959564	2.624805
C	-4.606411	20.542778	0.689363
H	-5.084025	21.170279	-0.056570
C	-5.338061	19.565372	1.348993
H	-6.391160	19.436969	1.115743
C	-0.799271	19.132939	8.229335
C	-2.626804	19.927939	1.945819
C	-1.159430	20.144642	2.288823
H	-0.787977	19.230971	2.762263
C	-3.761890	16.684562	8.380974
H	-3.497841	16.284648	7.397728
C	-4.178301	20.722003	5.931925
H	-5.101188	20.333060	6.384858
H	-3.701743	21.311841	6.726679
C	0.117968	19.776835	7.200654
H	-0.053948	19.279697	6.241286
C	-0.701947	19.490679	9.574945
H	0.048803	20.213813	9.881750
C	-3.258820	20.713250	0.982915
H	-2.692968	21.476589	0.457672
C	-5.579710	17.721186	3.061870
H	-4.894229	17.102567	3.649013
C	-6.545422	18.399163	4.044065
H	-6.004815	19.007481	4.774198
H	-7.132403	17.651110	4.587602
H	-7.238356	19.066583	3.521165
C	-3.920709	15.492161	9.330269
H	-2.968731	14.974406	9.483769
H	-4.636946	14.775314	8.916865
H	-4.298028	15.797781	10.311675
C	-5.088658	17.441713	8.221055
H	-5.405479	17.870854	9.177858
H	-5.879519	16.772034	7.867069
H	-4.991261	18.264317	7.505793
C	-1.554200	18.945789	10.525072
H	-1.466601	19.237345	11.567026
C	-0.288330	20.399082	1.053827
H	-0.434431	19.623035	0.296293
H	0.769102	20.407739	1.335905
H	-0.507216	21.366666	0.590348
C	1.599989	19.595556	7.550372
H	1.862644	20.115305	8.477794
H	2.231362	20.002629	6.754117
H	1.852035	18.538287	7.677967
C	-5.616211	22.681024	5.033844
H	-5.675294	23.385852	4.195470
C	-4.523792	21.632987	4.743706
H	-3.620201	22.161113	4.416014
H	-4.852777	21.043667	3.879917
C	-0.997830	21.277690	3.311537
H	-1.374678	22.222703	2.905554
H	0.055721	21.413511	3.578209
H	-1.553816	21.069512	4.231185
C	-6.968165	22.062605	5.249100
H	-7.090432	21.462307	6.150433
C	-0.230763	21.261013	7.019509
H	-1.280170	21.387283	6.734966
H	0.391608	21.713196	6.240320
H	-0.068754	21.817624	7.948992
C	-6.330726	16.789442	2.102903
H	-7.093822	17.327238	1.530639
H	-6.838614	15.998180	2.663394
H	-5.648429	16.318328	1.388394
C	-7.995606	22.170622	4.409936
H	-8.945584	21.681325	4.603295
H	-7.921726	22.758632	3.497692
H	-5.329714	23.258715	5.923714

7c.log

SCF (wB97x) = -1701.40423927
E(SCF)+ZPE(0 K)= -1700.602321
H(298 K)= -1700.557896
G(298 K)= -1700.679511
Lowest Frequency = 11.9280cm-1

Zn	-3.075082	19.270908	5.387900
N	-2.824300	18.399840	3.626449
N	-1.940761	17.979677	6.350161
C	-1.763381	18.259616	7.738413
C	-2.147166	17.280778	3.422614
C	-0.492381	16.036896	6.664266
H	0.303839	16.617486	7.139931
H	-0.043868	15.229865	6.084621
H	-1.090121	15.602495	7.471684
C	-2.650736	17.698569	8.675930
C	-2.098474	16.705763	2.025602
H	-3.112328	16.509248	1.662909

H	-1.525673	15.778606	1.995438
H	-1.648701	17.422604	1.331430
C	-1.459055	16.591018	4.436147
H	-0.936663	15.694250	4.127906
C	-2.518687	18.065238	10.014962
H	-3.198644	17.649923	10.752894
C	-4.819118	18.855662	2.289636
C	-1.346441	16.931051	5.794105
C	-3.457540	19.087607	2.549745
C	-4.773758	20.666716	0.680065
H	-5.292052	21.293686	-0.038891
C	-5.459962	19.662517	1.349025
H	-6.518002	19.516239	1.152914
C	-0.765319	19.168318	8.132590
C	-2.738268	20.080820	1.858536
C	-1.262713	20.318801	2.148601
H	-0.860076	19.405402	2.596558
C	-3.762302	16.758281	8.234822
H	-3.507865	16.384949	7.238693
C	-4.142009	20.841163	5.875933
H	-5.030414	20.474094	6.410163
H	-3.602864	21.450971	6.614366
C	0.168894	19.807936	7.116684
H	0.008701	19.309302	6.156182
C	-0.670074	19.505150	9.483526
H	0.090555	20.211296	9.805083
C	-3.421220	20.865103	0.929728
H	-2.891197	21.648087	0.395983
C	-5.597117	17.787682	3.042758
H	-4.873646	17.159371	3.570919
C	-6.515007	18.421210	4.097912
H	-5.941996	19.012245	4.818565
H	-7.062440	17.648032	4.647583
H	-7.239289	19.097315	3.632322
C	-3.915207	15.540629	9.152776
H	-2.964045	15.014145	9.279162
H	-4.640708	14.838939	8.729543
H	-4.277249	15.821293	10.147328
C	-5.088484	17.522364	8.107828
H	-5.395823	17.926422	9.078571
H	-5.884058	16.864047	7.743208
H	-4.995779	18.363298	7.413733
C	-1.537042	18.960819	10.420924
H	-1.451184	19.236470	11.467354
C	-0.444783	20.599794	0.883279
H	-0.608454	19.829375	0.123591
H	0.622687	20.622717	1.123700
H	-0.697103	21.568441	0.439367
C	1.645519	19.624607	7.487206
H	1.898851	20.151530	8.413149
H	2.288160	20.023077	6.695662
H	1.892582	18.567679	7.626965
C	-5.547626	22.849853	5.077155
H	-5.584430	23.604463	4.280858
C	-4.572557	21.724773	4.694176
H	-3.684668	22.177147	4.237206
H	-5.026408	21.113826	3.905936
C	-1.076045	21.442612	3.177260
H	-1.483123	22.386384	2.798344
H	-0.014383	21.591794	3.401105
H	-1.589450	21.215300	4.116854
C	-6.971946	22.373716	5.374338
H	-6.940765	21.531591	6.079844
C	-0.173453	21.292501	6.926712
H	-1.218754	21.421250	6.628965
H	0.459989	21.741008	6.154243
H	-0.021902	21.850631	7.857117
C	-6.391031	16.875986	2.099449
H	-7.195383	17.419203	1.592607
H	-6.852749	16.058805	2.662676
H	-5.746207	16.439665	1.330108
C	-7.805778	21.963522	4.188540
H	-8.792897	21.574983	4.442006
H	-5.158570	23.362902	5.965761
C	-7.474650	22.042522	2.901599
H	-7.520776	23.164273	5.904847
H	-8.169189	21.732416	2.126394
H	-6.505880	22.402022	2.568369

7d.log

SCF (wB97x) = -1740.71109336
E(SCF)+ZPE(0 K)= -1739.880475
H(298 K)= -1739.834745
G(298 K)= -1739.959676
Lowest Frequency = 8.6577cm-1

Zn	-3.183416	19.226260	5.203955
N	-2.867785	18.321912	3.471529
N	-2.062606	17.967081	6.226747
C	-1.936812	18.275219	7.614694
C	-2.162365	17.212914	3.308867
C	-0.600752	16.050369	6.625812
H	0.174786	16.648071	7.114277
H	-0.126779	15.235893	6.077692
H	-1.220378	15.627286	7.422680
C	-2.856169	17.729413	8.529891
C	-2.060654	16.613534	1.924960
H	-3.058037	16.375317	1.541996
H	-1.456983	15.705698	1.926866
H	-1.618413	17.331643	1.227334
C	-1.494096	16.554442	4.355191
H	-0.945928	15.662351	4.079940
C	-2.776916	18.125945	9.864637
H	-3.482580	17.723078	10.585156
C	-4.838817	18.674860	2.071029
C	-1.434832	16.918153	5.710841
C	-3.496206	18.967439	2.366694
C	-4.818758	20.458393	0.429910
H	-5.340401	21.051515	-0.314484
C	-5.481939	19.434849	1.093402
H	-6.521755	19.229502	0.854389
C	-0.956963	19.195866	8.026661
C	-2.795512	19.972459	1.673683
C	-1.332367	20.257847	1.985060
H	-0.913368	19.362632	2.454364
C	-3.943813	16.770903	8.068738
H	-3.652815	16.383243	7.088166
C	-4.255236	20.805050	5.649922
H	-5.139526	20.462097	6.206193
H	-3.700009	21.428666	6.365557
C	0.015455	19.814704	7.033937
H	-0.104290	19.292628	6.080106
C	-0.914853	19.562532	9.372439
H	-0.169387	20.278583	9.707282
C	-3.483083	20.714716	0.714002

H	-2.969219	21.508334	0.180323
C	-5.591776	17.587296	2.821805
H	-4.856364	16.991930	3.370644
C	-6.546158	18.198633	3.857101
H	-6.007933	18.844813	4.557972
H	-7.049527	17.415204	4.433229
H	-7.315754	18.806655	3.368145
C	-4.114029	15.567482	9.002382
H	-3.162434	15.052464	9.166916
H	-4.819742	14.851704	8.569355
H	-4.508932	15.861679	9.980349
C	-5.272870	17.518275	7.885446
H	-5.616294	17.935473	8.838348
H	-6.049345	16.845222	7.506705
H	-5.166133	18.348396	7.180486
C	-1.815463	19.035299	10.287734
H	-1.770978	19.334488	11.330267
C	-0.503035	20.538775	0.726952
H	-0.636341	19.752561	-0.022419
H	0.559819	20.592241	0.982378
H	-0.772860	21.493678	0.263975
C	1.476380	19.646131	7.468402
H	1.690279	20.196785	8.390467
H	2.149479	20.027438	6.693917
H	1.720858	18.594024	7.644486
C	-5.453651	22.944194	4.835955
H	-5.715212	23.484643	3.917682
C	-4.698709	21.662133	4.454860
H	-3.829343	21.955943	3.856170
H	-5.315303	21.064209	3.771735
C	-1.187118	21.402910	2.996787
H	-1.617587	22.328813	2.600194
H	-0.131661	21.585623	3.224879
H	-1.699020	21.176069	3.937283
C	-6.723101	22.726455	5.665766
H	-6.460052	22.291813	6.637041
C	-0.322279	21.292960	6.793599
H	-1.354854	21.411079	6.451037
H	0.340744	21.724109	6.036187
H	-0.209510	21.874522	7.715120
C	-6.338831	16.635865	1.879951
H	-7.156020	17.141648	1.355262
H	-6.777735	15.809262	2.447518
H	-5.667503	16.214322	1.125356
C	-7.763582	21.818107	4.988337
H	-7.330259	20.827887	4.812191
H	-4.777693	23.597254	5.403999
C	-8.270535	22.385096	3.693099
H	-7.181471	23.700683	5.879781
H	-8.878917	23.287151	3.774742
C	-8.000595	21.895912	2.485632
H	-8.606726	21.675196	5.676138
H	-7.374502	21.015926	2.359697
H	-8.379393	22.363744	1.581754

ts4a.log

SCF (wB97x) = -1396.43036181
E(SCF)+ZPE(0 K)= -1395.692007
H(298 K)= -1395.651060
G(298 K)= -1395.763338
Lowest Frequency = -727.4785cm-1

Mg	5.858888	0.762801	1.609651
N	5.930566	2.780197	1.659706
C	5.141416	4.981475	2.384407
H	5.579058	5.491978	1.523112
H	5.859818	5.081118	3.205128
H	4.216165	5.485980	2.663557
N	3.833323	0.744082	1.492326
C	4.910916	3.515902	2.087592
C	3.610064	3.018160	2.277038
H	2.885523	3.728703	2.655188
C	3.090276	1.759143	1.906320
C	1.587972	1.594422	2.001251
H	1.089462	2.560662	2.083909
H	1.333086	0.998716	2.883711
H	1.195273	1.061951	1.131752
C	7.121480	3.407165	1.192369
C	8.234877	3.544013	2.039922
C	9.409535	4.087543	1.517011
H	10.279240	4.194155	2.159626
C	9.485894	4.492787	0.191674
H	10.405723	4.918870	-0.196660
C	8.381044	4.343898	-0.637350
H	8.446747	4.657685	-1.675772
C	7.190724	3.793551	-0.162627
C	8.186981	3.082041	3.487009
H	7.149027	2.821128	3.716462
C	9.033416	1.815519	3.676445
H	10.088465	2.014911	3.457792
H	8.693604	1.014501	3.012989
H	8.961177	1.452221	4.706898
C	8.614116	4.183510	4.464876
H	9.669975	4.446176	4.340001
H	8.478646	3.848266	5.497991
H	8.026323	5.095453	4.320966
C	6.002153	3.627945	-1.100567
H	5.221700	3.086457	-0.558216
C	5.418416	4.985321	-1.516272
H	5.105070	5.571144	-0.647729
H	4.543288	4.844309	-2.159257
H	6.153511	5.577358	-2.072542
C	6.358968	2.796512	-2.338484
H	7.137853	3.276638	-2.940049
H	5.477016	2.679179	-2.977109
H	6.713145	1.799874	-2.060487
C	3.230895	-0.458219	1.021023
C	2.818020	-1.454315	1.925416
C	2.286986	-2.639050	1.412052
H	1.966585	-3.419396	2.096535
C	2.167799	-2.840531	0.045235
H	1.746615	-3.765785	-0.335507
C	2.606652	-1.860790	-0.836955
H	2.528761	-2.038628	-1.904194
C	3.156143	-0.665296	-0.373763
C	2.980519	-1.293080	3.429607
H	3.237592	-0.249596	3.633017
C	1.690771	-1.613239	4.196558
H	1.440212	-2.677647	4.138160
H	0.837156	-1.051243	3.804923
H	1.808938	-1.364082	5.255707
C	4.147235	-2.151688	3.939500
H	3.974400	-3.211889	3.722968
H	4.262069	-2.040327	5.022987
H	5.086545	-1.845825	3.467671

C	3.665884	0.392043	-1.346326
H	4.539691	0.864304	-0.882863
C	2.637930	1.507928	-1.580947
H	1.705859	1.097368	-1.984531
H	3.028120	2.237303	-2.299533
H	2.407323	2.045467	-0.658195
C	4.125481	-0.195567	-2.684097
H	4.811644	-1.036286	-2.541800
H	4.643308	0.568946	-3.269216
H	3.280844	-0.546180	-3.286920
H	6.707550	-0.438389	2.636079
C	7.173806	-0.177127	-0.016612
C	7.460531	-0.956587	1.081493
C	8.662126	-1.528689	1.732127
C	7.456811	-2.404457	1.390250
H	6.433649	-0.515846	-0.738703
H	8.844912	-1.334255	2.781676
H	9.547944	-1.601385	1.107739
H	6.864347	-2.770128	2.219640
H	7.525060	-3.072808	0.536884
H	7.840501	0.634826	-0.301216

ts4b.log

SCF (wB97x) =	-1435.77842766
E(SCF)+ZPE(0 K)=	-1435.006071
H(298 K)=	-1434.964557
G(298 K)=	-1435.077367

Lowest Frequency = -422.7056cm-1

N	-1.708743	-1.086297	-0.793867
N	1.240079	-1.338782	-0.742330
C	0.983470	-2.037806	-1.844415
C	2.512005	-1.470401	-0.110624
C	-2.971149	-0.984006	-0.140990
C	3.586112	-0.631214	-0.460354
C	-1.548925	-1.844443	-1.865433
C	-3.373546	-1.996648	0.753092
C	0.056213	2.121107	0.014370
C	-3.752560	0.175922	-0.317259
C	2.649814	-2.426334	0.920850
C	-3.342365	1.254061	-1.307861
H	-2.264759	1.159034	-1.470873
C	3.438116	0.491589	-1.475131
H	2.415685	0.456393	-1.865155
C	-0.292887	-2.173463	-2.418136
H	-0.334219	-2.750060	-3.335036
C	-2.739492	-2.470561	-2.563884
H	-2.694241	-3.559243	-2.467312
H	-2.698975	-2.238671	-3.632044
H	-3.691694	-2.125446	-2.160989
C	3.898913	-2.578799	1.522162
H	4.031054	-3.317741	2.305406
C	-0.236891	-0.000085	2.277143
H	0.463765	-0.814647	2.497094
H	-1.224903	-0.427763	2.023003
C	-2.550626	-3.258992	0.968785
H	-1.636476	-3.176482	0.373363
C	4.814829	-0.815867	0.177549
H	5.654370	-0.180185	-0.090865
C	-4.944986	0.291629	0.397769
H	-5.564037	1.173477	0.264267
C	1.461719	-3.265870	1.377701
H	0.561475	-2.662405	1.216478
C	2.115485	-2.756341	-2.549457
H	2.768187	-2.026203	-3.036326
H	1.738136	-3.442488	-3.308102
H	2.733260	-3.313636	-1.841447
C	4.981269	-1.792027	1.147237
H	5.945863	-1.928794	1.626073
C	-4.574157	-1.834773	1.446344
H	-4.900086	-2.610686	2.133826
C	-5.358314	-0.703179	1.274305
H	-6.289397	-0.595402	1.821807
C	4.405510	0.349482	-2.658399
H	5.446258	0.445508	-2.330893
H	4.218608	1.132492	-3.400126
H	4.306722	-0.619542	-3.154754
C	-2.128689	-3.423981	2.433895
H	-1.517460	-2.582162	2.772627
H	-1.544331	-4.342347	2.553593
H	-2.997699	-3.498489	3.095993
C	3.639019	1.855660	-0.797825
H	2.992267	1.966483	0.076568
H	3.417261	2.671121	-1.494098
H	4.673997	1.975987	-0.459911
C	-3.605902	2.673507	-0.794100
H	-4.676065	2.903466	-0.756619
H	-3.140756	3.405736	-1.461552
H	-3.195272	2.819108	0.209544
C	1.286858	-4.544168	0.545246
H	1.071601	-4.319924	-0.502177
H	0.450197	-5.135504	0.934080
H	2.189768	-5.163271	0.588696
C	-3.310318	-4.504378	0.489378
H	-4.197038	-4.685931	1.106586
H	-2.671320	-5.391237	0.553276
H	-3.645118	-4.396321	-0.545622
C	1.526701	-3.610494	2.869575
H	2.314977	-4.339615	3.085258
H	0.582026	-4.053859	3.193590
H	1.710976	-2.721048	3.479742
C	-4.028683	1.035592	-2.664167
H	-3.774236	0.060139	-3.086782
H	-3.721976	1.805486	-3.379760
H	-5.118148	1.083288	-2.557567
H	0.918321	2.394235	-0.589868
H	-0.873506	2.624197	-0.242599
C	0.270460	1.824870	1.389269
H	1.311467	1.746399	1.711364
Mg	-0.140167	-0.051852	0.006670
C	-0.653462	2.234725	2.518485
C	-0.415345	1.016874	3.403854
H	0.521289	1.134910	3.959959
H	-1.205844	0.791107	4.125937
H	-0.425429	3.227522	2.925549
H	-1.688592	2.245114	2.155375

ts4c.log

SCF (wB97x) =	-1475.09642768
E(SCF)+ZPE(0 K)=	-1474.297595
H(298 K)=	-1474.255096
G(298 K)=	-1474.370455

Lowest Frequency = -764.4157cm-1

Mg	6.135212	1.060851	1.328688
N	6.233059	3.056866	1.639083
C	5.417438	5.369500	1.577586
H	6.188115	5.640659	2.302490
H	4.496767	5.903128	1.816907
H	5.765181	5.710265	0.596143
N	4.120621	1.037418	1.508896
C	5.193549	3.872088	1.542558
C	3.863280	3.433306	1.389164
H	3.120858	4.216207	1.291875
C	3.358910	2.118235	1.429852
C	1.854002	1.962874	1.360192
H	1.561553	1.683469	0.342029
H	1.346284	2.893247	1.617573
H	1.504624	1.168122	2.023054
C	7.538814	3.532138	1.947508
C	7.992461	3.408766	3.279895
C	9.304953	3.778011	3.570508
H	9.672559	3.696396	4.588293
C	10.159708	4.236071	2.574290
H	11.180758	4.512128	2.818610
C	9.706858	4.328989	1.266399
H	10.384519	4.672685	0.489863
C	8.396459	3.983629	0.928174
C	7.059840	2.889123	4.365867
H	6.391671	2.163601	3.890447
C	7.785023	2.161352	5.501280
H	8.353125	2.852779	6.133291
H	8.475520	1.402306	5.122383
H	7.054143	1.662445	6.145310
C	6.171881	4.005116	4.934968
H	6.784768	4.807452	5.360383
H	5.527674	3.611014	5.728188
H	5.524946	4.436814	4.168111
C	7.940779	4.064120	-0.520805
H	6.856472	3.922727	-0.539523
C	8.241477	5.431116	-1.148219
H	7.841578	6.247421	-0.538611
H	7.795869	5.496678	-2.145807
H	9.318131	5.596991	-1.259803
C	8.554305	2.926542	-1.349069
H	9.649103	2.965765	-1.321022
H	8.238543	2.997286	-2.395450
H	8.232131	1.952621	-0.965750
C	3.568697	-0.260188	1.702895
C	3.585018	-0.800274	3.008355
C	3.136834	-2.107801	3.190515
H	3.135096	-2.541251	4.185342
C	2.704564	-2.875519	2.115052
H	2.365716	-3.894263	2.275776
C	2.718167	-2.339583	0.835656
H	2.394774	-2.950373	-0.002595
C	3.145474	-1.030109	0.604420
C	4.078270	0.035699	4.181982
H	4.864712	0.692609	3.796092
C	2.972814	0.944317	4.738929
H	2.115385	0.349527	5.072718
H	2.624030	1.660749	3.991981
H	3.346038	1.515044	5.595975
C	4.695886	-0.795000	5.310424
H	3.938523	-1.363088	5.861724
H	5.188059	-0.132227	6.029003
H	5.442683	-1.500332	4.934591
C	3.182898	-0.483534	-0.814610
H	3.370505	0.592252	-0.755008
C	1.850929	-0.681562	-1.549012
H	1.648274	-1.740328	-1.741554
H	1.872937	-0.172073	-2.517507
H	1.011998	-0.283129	-0.969890
C	4.347851	-1.097386	-1.603727
H	5.306028	-0.845307	-1.137185
H	4.362848	-0.715096	-2.630013
H	4.260379	-2.188811	-1.648755
H	7.024548	0.226897	-0.017446
C	7.444118	-2.000309	0.563316
C	7.821423	-0.692745	1.229831
C	9.187421	-0.324229	0.686611
C	9.243705	-0.905305	-0.738149
C	8.118492	-1.975594	-0.819947
H	7.875027	-2.791794	1.192091
H	6.364154	-2.168064	0.530847
H	9.393401	0.748319	0.739432
H	9.915000	-0.824942	1.340261
H	9.070576	-0.119854	-1.477537
H	10.228408	-1.332082	-0.947831
H	7.394483	-1.701808	-1.590989
H	8.510899	-2.961815	-1.082835
C	7.382242	-0.329872	2.512114
H	8.033674	0.288403	3.126310
H	6.693812	-0.994369	3.030223

ts4d.log

SCF (wB97x) = -1514.41023348
E(SCF)+ZPE(0 K)= -1513.581787
H(298 K)= -1513.538299
G(298 K)= -1513.655546
Lowest Frequency = -786.2026cm-1

Mg	5.930437	0.979328	1.296479
N	5.991081	2.965044	1.662329
C	5.255449	5.081980	2.638833
H	6.105084	5.145719	3.326260
H	4.380373	5.523585	3.116118
H	5.516521	5.678940	1.760652
N	3.907839	0.932844	1.490449
C	5.010173	3.641883	2.247362
C	3.737503	3.108997	2.512584
H	3.047254	3.766975	3.026642
C	3.201288	1.870937	2.095590
C	1.734347	1.639653	2.391819
H	1.188271	2.583606	2.429759
H	1.633479	1.152028	3.367528
H	1.272341	0.985974	1.649394
C	7.163172	3.623120	1.191533
C	8.330957	3.630742	1.976582
C	9.504441	4.144753	1.421871
H	10.416303	4.147646	2.012928
C	9.526299	4.653447	0.130299
H	10.448121	5.048931	-0.284789
C	8.360911	4.659584	-0.626053
H	8.379995	5.066451	-1.633775
C	7.167249	4.147316	-0.118263
C	8.331228	3.092806	3.399306
H	7.292288	2.884042	3.672654
C	9.104797	1.771099	3.498710

H	10.143269	1.897907	3.172718
H	8.641783	0.994605	2.881519
H	9.115775	1.408786	4.532111
C	8.873754	4.123589	4.398136
H	9.938740	4.320817	4.235960
H	8.759779	3.755916	5.422791
H	8.343680	5.077586	4.315478
C	5.906680	4.174166	-0.972977
H	5.096704	3.724107	-0.391644
C	5.491940	5.612721	-1.313196
H	5.359645	6.218521	-0.412075
H	4.546601	5.617031	-1.865492
H	6.245488	6.105945	-1.936788
C	6.061599	3.345494	-2.254451
H	6.866240	3.733628	-2.888263
H	5.134724	3.376970	-2.837602
H	6.284333	2.300186	-2.026503
C	3.325513	-0.265395	0.991708
C	3.113297	-1.366498	1.841654
C	2.680369	-2.568216	1.276772
H	2.520628	-3.430023	1.918901
C	2.461433	-2.684297	-0.087428
H	2.127568	-3.627486	-0.508519
C	2.675947	-1.588844	-0.916546
H	2.507198	-1.692934	-1.983049
C	3.114445	-0.369375	-0.402096
C	3.365510	-1.292267	3.339971
H	3.606032	-0.255627	3.591533
C	2.120097	-1.692002	4.143288
H	1.883656	-2.753411	4.013250
H	1.241419	-1.117609	3.834257
H	2.285327	-1.521102	5.211817
C	4.576874	-2.143861	3.743679
H	4.427961	-3.195097	3.472439
H	4.734697	-2.094297	4.826366
H	5.483692	-1.781814	3.249613
C	3.352597	0.829738	-1.311511
H	4.214643	1.375907	-0.911416
C	2.163697	1.801435	-1.287356
H	1.249409	1.301364	-1.625172
H	2.354172	2.650455	-1.952765
H	1.986716	2.198375	-0.285348
C	3.684526	0.443869	-2.755692
H	4.483773	-0.302041	-2.806106
H	4.012809	1.327695	-3.309735
H	2.811161	0.039607	-3.279021
H	6.849840	-0.380787	2.065469
C	6.599789	-2.183470	0.465707
C	7.309058	-0.841890	0.433502
C	8.782301	-0.939726	0.776709
C	9.456418	-1.864126	-0.252964
C	8.772539	-3.235160	-0.295853
C	7.269223	-3.108136	-0.565586
H	9.244383	0.052608	0.766390
H	6.688023	-2.626768	1.462622
H	5.533089	-2.066789	0.248319
H	9.405051	-1.396937	-1.243578
H	10.516869	-1.974591	-0.002330
H	9.242068	-3.867230	-1.057392
H	8.920883	-3.739242	0.669091
H	7.108539	-2.703250	-1.571931
H	6.790383	-4.092534	-0.533424
H	8.904696	-1.354850	1.782819
C	6.897069	0.107051	-0.515939
H	7.623167	0.829191	-0.885968
H	6.063020	-0.138176	-1.169962

ts5a.log

SCF (wB97x) = -1622.69435025
E(SCF)+ZPE(0 K)= -1621.955379
H(298 K)= -1621.914451
G(298 K)= -1622.025788
Lowest Frequency = -778.5513cm-1

Zn	5.719909	0.730969	1.510310
N	5.968471	2.700385	1.477527
C	5.306672	4.988886	2.071788
H	6.160448	5.310408	1.473432
H	5.536391	5.195171	3.122752
H	4.431591	5.582356	1.799862
N	3.766723	0.763003	1.600279
C	5.021618	3.511370	1.903577
C	3.715767	3.089639	2.244170
H	3.062552	3.867349	2.621265
C	3.117286	1.840111	2.026720
C	1.629947	1.726836	2.277266
H	1.219313	2.660316	2.662871
H	1.432011	0.925563	2.996379
H	1.099939	1.462359	1.358613
C	7.268618	3.139752	1.109289
C	8.243410	3.371754	2.096550
C	9.543366	3.672354	1.684183
H	10.311058	3.849258	2.432762
C	9.872144	3.747620	0.339038
H	10.888233	3.983486	0.038269
C	8.896078	3.520013	-0.624501
H	9.164665	3.580349	-1.673915
C	7.586230	3.206694	-0.264255
C	7.922882	3.308725	3.583159
H	6.847047	3.149734	3.692681
C	8.615429	2.121547	4.264558
H	9.701470	2.158696	4.123597
H	8.238437	1.178111	3.859518
H	8.415158	2.128104	5.341147
C	8.277112	4.626373	4.287169
H	9.359446	4.792879	4.303538
H	7.931069	4.609083	5.325636
H	7.818554	5.483729	3.785480
C	6.510996	2.963900	-1.315170
H	5.840966	2.193421	-0.919234
C	5.658931	4.220068	-1.547737
H	5.157518	4.542055	-0.631982
H	4.886388	4.021558	-2.298703
H	6.279674	5.048340	-1.906858
C	7.066027	2.449372	-2.646177
H	7.621757	3.225397	-3.183859
H	6.243960	2.132916	-3.294433
H	7.733413	1.593529	-2.504226
C	3.060680	-0.357168	1.078722
C	2.856709	-1.495133	1.877306
C	2.233597	-2.606597	1.307894
H	2.073428	-3.495611	1.911893
C	1.819079	-2.596171	-0.017107
H	1.333198	-3.468299	-0.443573
C	2.034773	-1.466732	-0.796804

H	1.716068	-1.465835	-1.835991
C	2.661231	-0.335945	-0.273177
C	3.338572	-1.537690	3.317517
H	3.669032	-0.529306	3.583384
C	2.226258	-1.936731	4.294271
H	1.888120	-2.964576	4.124426
H	1.355909	-1.280258	4.196698
H	2.586347	-1.875228	5.326194
C	4.552670	-2.467537	3.447407
H	4.282407	-3.500798	3.201823
H	4.945112	-2.451771	4.469705
H	5.353806	-2.155917	2.771062
C	2.906521	0.875514	-1.163278
H	3.424101	1.632547	-0.567739
C	1.591944	1.492270	-1.660466
H	1.038143	0.790363	-2.293468
H	1.792793	2.390388	-2.253695
H	0.939633	1.776913	-0.829982
C	3.818275	0.531491	-2.347988
H	4.781437	0.145665	-2.004913
H	4.004430	1.426677	-2.951595
H	3.362733	-0.221079	-3.000682
H	6.721729	-0.011293	2.604997
C	6.596880	-0.494123	-0.031576
C	7.333474	-0.696697	1.137288
C	8.777848	-0.600064	1.452883
C	7.979932	-1.850781	1.813050
H	5.857052	-1.229623	-0.336948
H	9.107629	0.033868	2.266147
H	9.447586	-0.634102	0.599082
H	7.790017	-2.029813	2.864637
H	8.107088	-2.741342	1.204468
H	6.992218	0.180260	-0.784778

ts5b.log

SCF (wB97x) = -1662.01968132
E(SCF)+ZPE(0 K)= -1661.251107
H(298 K)= -1661.209299
G(298 K)= -1661.323860
Lowest Frequency = -743.3706cm-1

Zn	5.663557	0.954997	0.763718
N	5.917035	2.855924	1.190358
C	5.199230	5.009850	2.121566
H	6.138705	5.405416	1.733621
H	5.262600	5.003875	3.214763
H	4.382899	5.679375	1.841726
N	3.733329	0.839131	1.116587
C	4.921903	3.603923	1.631844
C	3.586677	3.166540	1.741599
H	2.886836	3.910876	2.104690
C	3.050453	1.874480	1.570259
C	1.611348	1.692649	2.005225
H	1.015975	2.566089	1.730201
H	1.574954	1.596954	3.095476
H	1.159366	0.798133	1.574822
C	7.276116	3.273401	1.203702
C	8.038618	3.143684	2.380033
C	9.397497	3.460290	2.326904
H	10.000564	3.366881	3.226200
C	9.991498	3.886677	1.147608
H	11.049290	4.130009	1.126663
C	9.228904	3.994081	-0.009293
H	9.703635	4.318913	-0.929669
C	7.869501	3.682497	-0.008383
C	7.430568	2.657535	3.687474
H	6.363148	2.487216	3.521151
C	8.037073	1.316451	4.122224
H	9.114297	1.405750	4.301477
H	7.872037	0.555762	3.354027
H	7.569723	0.968705	5.049737
C	7.574611	3.706988	4.798355
H	8.625534	3.858712	5.067497
H	7.045256	3.384594	5.700793
H	7.170430	4.675593	4.489864
C	7.032951	3.801599	-1.273560
H	6.219805	3.073508	-1.188746
C	6.391959	5.192754	-1.384472
H	5.730482	5.398307	-0.539490
H	5.798283	5.269931	-2.301346
H	7.162511	5.971376	-1.410314
C	7.815442	3.475992	-2.549974
H	8.542691	4.257973	-2.794630
H	7.128389	3.398241	-3.398109
H	8.357389	2.529331	-2.462138
C	3.211109	-0.482176	1.064944
C	3.237363	-1.293750	2.214982
C	2.819927	-2.621136	2.098599
H	2.832767	-3.261175	2.976833
C	2.394400	-3.137863	0.883127
H	2.072183	-4.172182	0.813046
C	2.388709	-2.328260	-0.246529
H	2.063365	-2.742677	-1.195426
C	2.802680	-0.998127	-0.182258
C	3.718158	-0.772216	3.561202
H	3.977618	0.283504	3.441828
C	2.618009	-0.871362	4.627052
H	2.373539	-1.915740	4.849296
H	1.697066	-0.379881	4.299901
H	2.945716	-0.402466	5.560490
C	4.989744	-1.498092	4.020954
H	4.809667	-2.570316	4.156556
H	5.336877	-1.092036	4.977107
H	5.789948	-1.367751	3.287080
C	2.793731	-0.109323	-1.416909
H	3.582247	0.637487	-1.278492
C	1.463685	0.647958	-1.543763
H	0.626061	-0.054212	-1.621336
H	1.464554	1.278114	-2.439283
H	1.286084	1.293785	-0.680555
C	3.095853	-0.869241	-2.712455
H	3.992871	-1.489074	-2.618599
H	3.254896	-0.160797	-3.531122
H	2.266185	-1.521424	-3.006676
H	6.576766	-0.075449	1.692821
C	6.529137	0.075308	-0.949613
C	7.113416	-0.607900	0.138233
C	8.562588	-0.604073	0.626585
C	8.266501	-1.923575	1.386590
C	6.954430	-2.065949	0.571316
H	5.767553	-0.442019	-1.525866
H	8.916654	0.262110	1.187970
H	9.220756	-0.764382	-0.235284
H	8.077022	-1.755080	2.449049
H	8.999218	-2.725443	1.278831

H 6.038087 -2.354394 1.089004
H 7.084137 -2.706701 -0.308629
H 7.135528 0.806188 -1.476746

ts5c.log

SCF (wB97x) = -1701.36000459
E(SCF)+ZPE(0 K)= -1700.561525
H(298 K)= -1700.518699
G(298 K)= -1700.634972
Lowest Frequency = -857.5434cm-1

Zn 6.071030 1.034760 1.512264
N 6.258357 2.991088 1.726540
C 5.463332 5.306244 1.593304
H 6.373344 5.584970 2.127186
H 4.614611 5.839499 2.025306
H 5.574508 5.637052 0.554848
N 4.103832 0.982665 1.572072
C 5.228981 3.810349 1.617088
C 3.891160 3.386051 1.485126
H 3.159959 4.180505 1.394949
C 3.368276 2.079825 1.492850
C 1.864613 1.950122 1.366973
H 1.603087 1.760473 0.320021
H 1.363399 2.866538 1.681532
H 1.480940 1.111883 1.951689
C 7.595849 3.436363 1.904295
C 8.158314 3.357013 3.196694
C 9.508886 3.666635 3.350322
H 9.962939 3.613109 4.334473
C 10.290729 4.034235 2.260872
H 11.342515 4.264562 2.399500
C 9.722760 4.104799 0.997253
H 10.339561 4.388476 0.148745
C 8.372494 3.810851 0.793046
C 7.294293 2.968352 4.388360
H 6.544525 2.259127 4.023112
C 8.073387 2.275609 5.510411
H 8.727947 2.973158 6.044708
H 8.691674 1.456372 5.131227
H 7.375381 1.861561 6.244477
C 6.538144 4.184046 4.944624
H 7.240089 4.960511 5.268541
H 5.929358 3.893138 5.807160
H 5.869944 4.618792 4.197762
C 7.789203 3.881508 -0.610103
H 6.712356 3.707647 -0.537078
C 8.000433 5.264721 -1.240357
H 7.627307 6.061659 -0.589849
H 7.477453 5.330176 -2.199839
H 9.061028 5.461499 -1.429865
C 8.357371 2.773709 -1.506816
H 9.449282 2.839089 -1.575317
H 7.949714 2.853286 -2.520150
H 8.089779 1.790312 -1.109831
C 3.532352 -0.313760 1.691464
C 3.468046 -0.899574 2.974237
C 2.997162 -2.207843 3.079600
H 2.934310 -2.677860 4.055836
C 2.616768 -2.928171 1.953024
H 2.259284 -3.948174 2.055120
C 2.699736 -2.341625 0.698420
H 2.409658 -2.913146 -0.178938
C 3.152783 -1.029887 0.542689
C 3.885245 -0.109316 4.207487
H 4.674009 0.581261 3.893076
C 2.725234 0.739321 4.748884
H 1.870988 0.105201 5.011166
H 2.390272 1.478536 4.017643
H 3.036623 1.280526 5.648585
C 4.463728 -0.983350 5.324662
H 3.692696 -1.592094 5.809835
H 4.907291 -0.348596 6.098048
H 5.241668 -1.655900 4.951804
C 3.254136 -0.419570 -0.846926
H 3.462280 0.647466 -0.730762
C 1.945286 -0.556439 -1.634800
H 1.727631 -1.601513 -1.879227
H 2.011547 -0.006716 -2.579060
H 1.095011 -0.164688 -1.067796
C 4.434304 -1.018930 -1.622480
H 5.374212 -0.803947 -1.106432
H 4.494964 -0.588733 -2.627831
H 4.331222 -2.105277 -1.722494
H 6.911483 0.401041 0.194152
C 7.471041 -1.801975 0.631116
C 7.708743 -0.515751 1.397587
C 9.091655 -0.021736 0.994650
C 9.418082 -0.701372 -0.357385
C 8.215860 -1.618786 -0.694845
H 7.944710 -2.593041 1.230014
H 6.412910 -2.056189 0.531567
H 9.156428 1.068147 0.968852
H 9.778680 -0.360137 1.780185
H 9.589018 0.032664 -1.148366
H 10.336820 -1.288324 -0.267865
H 7.562342 -1.130838 -1.423377
H 8.529207 -2.573482 -1.125331
C 7.179597 -0.283092 2.696687
H 7.803397 0.256895 3.402737
H 6.519728 -1.041443 3.110334

ts5d.log

SCF (wB97x) = -1740.67450594
E(SCF)+ZPE(0 K)= -1739.845875
H(298 K)= -1739.802166
G(298 K)= -1739.920637
Lowest Frequency = -857.4571cm-1

Zn 5.800544 0.958259 1.159863
N 6.052824 2.871790 1.545427
C 5.454925 4.997999 2.600578
H 5.950145 4.960087 3.577204
H 4.551710 5.601520 2.701451
H 6.140847 5.490308 1.908539
N 3.852740 0.917358 1.467137
C 5.123963 3.589538 2.154238
C 3.829201 3.120867 2.448226
H 3.188945 3.818920 2.974291
C 3.224758 1.899834 2.087952
C 1.772197 1.724899 2.478034
H 1.258725 2.687026 2.516674
H 1.719183 1.273389 3.474876
H 1.243905 1.062460 1.790021

C	7.300595	3.410636	1.129069
C	8.390528	3.447238	2.015648
C	9.629657	3.866896	1.526305
H	10.485011	3.894052	2.195914
C	9.787788	4.244855	0.200944
H	10.758395	4.569421	-0.161246
C	8.699969	4.203672	-0.664319
H	8.834992	4.496024	-1.700746
C	7.445785	3.781836	-0.225768
C	8.252444	3.023711	3.469732
H	7.190915	2.854664	3.670084
C	8.972101	1.694054	3.729812
H	10.038428	1.767421	3.488226
H	8.533180	0.897163	3.123632
H	8.881819	1.408025	4.783066
C	8.742977	4.114992	4.430257
H	9.824518	4.266817	4.348286
H	8.528653	3.834307	5.466414
H	8.257844	5.074580	4.226294
C	6.252791	3.733536	-1.170842
H	5.613085	2.912432	-0.833313
C	5.420108	5.021472	-1.091061
H	5.012246	5.177083	-0.089727
H	4.577187	4.973592	-1.788657
H	6.029805	5.893675	-1.352050
C	6.635065	3.443643	-2.625342
H	7.143061	4.294167	-3.093365
H	5.732247	3.244936	-3.211344
H	7.289606	2.571056	-2.707280
C	3.204330	-0.262439	1.011675
C	3.024476	-1.353552	1.879445
C	2.512086	-2.541695	1.353410
H	2.373964	-3.397588	2.008572
C	2.182898	-2.649608	0.010268
H	1.787033	-3.581724	-0.380925
C	2.362505	-1.560130	-0.835266
H	2.104161	-1.655662	-1.884926
C	2.877873	-0.355577	-0.359255
C	3.387608	-1.273367	3.354057
H	3.675570	-0.241588	3.572903
C	2.193441	-1.628796	4.249961
H	1.913801	-2.682639	4.146144
H	1.313857	-1.027294	4.000839
H	2.441756	-1.456635	5.302164
C	4.601933	-2.152790	3.678412
H	4.412160	-3.201363	3.423101
H	4.837612	-2.101832	4.746664
H	5.478663	-1.812727	3.120987
C	3.070051	0.842279	-1.279299
H	3.943047	1.389954	-0.911028
C	1.870257	1.798460	-1.209688
H	0.949111	1.285562	-1.507918
H	2.020971	2.648538	-1.883519
H	1.728638	2.195357	-0.201946
C	3.352588	0.459767	-2.735102
H	4.154820	-0.279893	-2.813779
H	3.655842	1.348099	-3.297857
H	2.465056	0.050919	-3.230705
H	6.740487	-0.184541	1.959072
C	6.718176	-2.071685	0.522071
C	7.179800	-0.630792	0.391305
C	8.670485	-0.462055	0.625113
C	9.415235	-1.292309	-0.434040
C	8.976772	-2.760862	-0.390764
C	7.457585	-2.905599	-0.537860
H	8.950922	0.594920	0.569124
H	6.962647	-2.454020	1.519036
H	5.632783	-2.142334	0.395886
H	9.212226	-0.876023	-1.427711
H	10.494110	-1.211260	-0.265145
H	9.488058	-3.330578	-1.174072
H	9.285174	-3.196929	0.569494
H	7.150722	-2.574692	-1.537014
H	7.163367	-3.955964	-0.442103
H	8.936110	-0.824816	1.624253
C	6.571849	0.168742	-0.620417
H	7.188588	0.912172	-1.118580
H	5.772894	-0.276777	-1.206463

ts6a.log

SCF (wB97x) = -1396.47990468
E(SCF)+ZPE(0 K)= -1395.737553
H(298 K)= -1395.696860
G(298 K)= -1395.808279
Lowest Frequency = -444.8702cm-1

N	-1.775215	-1.181987	-0.857856
N	1.179893	-1.342169	-0.788721
C	0.954225	-2.053841	-1.886096
C	2.446281	-1.403972	-0.136838
C	-3.056138	-1.105550	-0.241424
C	3.504575	-0.556477	-0.514626
C	-1.582470	-1.918613	-1.940228
C	-3.492663	-2.154033	0.592715
C	-0.035416	2.011903	0.279698
C	-3.822919	0.068736	-0.387955
C	2.585550	-2.285509	0.959765
C	-3.352030	1.198889	-1.290500
H	-2.261123	1.137581	-1.353889
C	3.359462	0.480064	-1.617613
H	2.350848	0.386923	-2.032731
C	-0.394446	1.440304	2.788716
H	0.137844	1.615897	3.722519
H	-1.348638	1.967671	2.770832
C	-0.313693	-2.224227	-2.472973
H	-0.328002	-2.802270	-3.389733
C	-2.755724	-2.563178	-2.650230
H	-2.814477	-3.620085	-2.371176
H	-2.610017	-2.518350	-3.732105
H	-3.706432	-2.094062	-2.394986
C	3.817947	-2.350152	1.608771
H	3.950184	-3.030142	2.443586
C	-0.458915	-0.016682	2.312434
H	0.096769	-0.726032	2.923234
H	-1.502482	-0.314741	2.139386
C	-2.659345	-3.408071	0.816808
H	-1.765120	-3.342478	0.190072
C	4.717424	-0.652912	0.171873
H	5.544127	-0.009357	-0.116851
C	-5.043884	0.154651	0.281572
H	-5.653159	1.046105	0.170004
C	1.416236	-3.146084	1.423765
H	0.501501	-2.567762	1.251526
C	2.107137	-2.749728	-2.579780
H	2.658052	-2.025303	-3.187124

H	1.750522	-3.543544	-3.237032
H	2.811027	-3.171672	-1.859258
C	4.884019	-1.550176	1.214625
H	5.835931	-1.617370	1.732097
C	-4.718718	-2.019379	1.246051
H	-5.068306	-2.820301	1.892059
C	-5.495252	-0.880010	1.091154
H	-6.447434	-0.794105	1.605274
C	4.369383	0.267618	-2.754535
H	5.394088	0.437552	-2.406887
H	4.177613	0.969089	-3.572734
H	4.325523	-0.747872	-3.156602
C	-2.187812	-3.504426	2.273575
H	-1.588841	-2.634424	2.558107
H	-1.577177	-4.402543	2.416200
H	-3.037467	-3.565805	2.962003
C	3.507359	1.899933	-1.050371
H	2.833845	2.067825	-0.206172
H	3.286772	2.648597	-1.818261
H	4.529097	2.075484	-0.696540
C	-3.693348	2.588344	-0.743094
H	-4.766417	2.800964	-0.799577
H	-3.179798	3.356423	-1.329585
H	-3.383124	2.692642	0.301110
C	1.285638	-4.438527	0.604904
H	1.093147	-4.232392	-0.450546
H	0.452215	-5.042289	0.981173
H	2.200080	-5.037434	0.678976
C	-3.419869	-4.679407	0.415506
H	-4.273896	-4.858268	1.077630
H	-2.762968	-5.552958	0.479372
H	-3.803772	-4.612475	-0.606264
C	1.472110	-3.471504	2.919578
H	2.269206	-4.185918	3.152292
H	0.530271	-3.925621	3.238371
H	1.636860	-2.572238	3.520914
C	-3.899066	1.027404	-2.714827
H	-3.563550	0.086758	-3.159470
H	-3.558958	1.844869	-3.359020
H	-4.994781	1.029168	-2.710612
H	0.744904	2.318610	-0.411110
H	-0.946723	2.606116	0.246038
Mg	-0.257521	-0.184072	0.064711
C	0.392264	1.550988	1.556754
H	1.447409	1.299924	1.679813

ts6b.log

SCF (wB97x) = -1435.77842766
E(SCF)+ZPE(0 K)= -1435.006071
H(298 K)= -1434.964557
G(298 K)= -1435.077367
Lowest Frequency = -422.7056cm-1

N	-1.708743	-1.086297	-0.793867
N	1.240079	-1.338782	-0.742330
C	0.983470	-2.037806	-1.844415
C	2.512005	-1.470401	-0.110624
C	-2.971149	-0.984006	-0.140990
C	3.586112	-0.631214	-0.460354
C	-1.548925	-1.844443	-1.865433
C	-3.373546	-1.996648	0.753092
C	0.056213	2.121107	0.014370
C	-3.752560	0.175922	-0.317259
C	2.649814	-2.426334	0.920850
C	-3.342365	1.254061	-1.307861
H	-2.264759	1.159034	-1.470873
C	3.438116	0.491589	-1.475131
H	2.415685	0.456393	-1.865155
C	-0.292887	-2.173463	-2.418136
H	-0.334219	-2.750060	-3.335036
C	-2.739492	-2.470561	-2.563884
H	-2.694241	-3.559243	-2.467312
H	-2.698975	-2.238671	-3.632044
H	-3.691694	-2.125446	-2.160989
C	3.898913	-2.578799	1.522162
H	4.031054	-3.317741	2.305406
C	-0.236891	-0.000085	2.277143
H	0.463765	-0.814647	2.497094
H	-1.224903	-0.427763	2.023003
C	-2.550626	-3.258992	0.968785
H	-1.636476	-3.176482	0.373363
C	4.814829	-0.815867	0.177549
H	5.654370	-0.180185	-0.090865
C	-4.944986	0.291629	0.397769
H	-5.564037	1.173477	0.264267
C	1.461719	-3.265870	1.377701
H	0.561475	-2.662405	1.216478
C	2.115485	-2.756341	-2.549457
H	2.768187	-2.026203	-3.036326
H	1.738136	-3.442488	-3.308102
H	2.733260	-3.313636	-1.841447
C	4.981269	-1.792027	1.147237
H	5.945863	-1.928794	1.626073
C	-4.574157	-1.834773	1.446344
H	-4.900086	-2.610686	2.133826
C	-5.358314	-0.703179	1.274305
H	-6.289397	-0.595402	1.821807
C	4.405510	0.349482	-2.658399
H	5.446258	0.445508	-2.330893
H	4.218608	1.132492	-3.400126
H	4.306722	-0.619542	-3.154754
C	-2.128689	-3.423981	2.433895
H	-1.517460	-2.582162	2.772627
H	-1.544331	-4.342347	2.553593
H	-2.997699	-3.498489	3.095993
C	3.639019	1.855660	-0.797825
H	2.992267	1.966483	0.076568
H	3.417261	2.671121	-1.494098
H	4.673997	1.975987	-0.459911
C	-3.605902	2.673507	-0.794100
H	-4.676065	2.903466	-0.756619
H	-3.140756	3.405736	-1.461552
H	-3.195272	2.819108	0.209544
C	1.286858	-4.544168	0.545246
H	1.071601	-4.319924	-0.502177
H	0.450197	-5.135504	0.934080
H	2.189768	-5.163271	0.588696
C	-3.310318	-4.504378	0.489378
H	-4.197038	-4.685931	1.106586
H	-2.671320	-5.391237	0.553276
H	-3.645118	-4.396321	-0.545622
C	1.526701	-3.610494	2.869575
H	2.314977	-4.339615	3.085258
H	0.582026	-4.053859	3.193590
H	1.710976	-2.721048	3.479742

C	-4.028683	1.035592	-2.664167
H	-3.774236	0.060139	-3.086782
H	-3.721976	1.805486	-3.379760
H	-5.118148	1.083288	-2.557567
H	0.918321	2.394235	-0.589868
H	-0.873506	2.624197	-0.242599
C	0.270460	1.824870	1.389269
H	1.311467	1.746399	1.711364
Mg	-0.140167	-0.051852	0.006670
C	-0.653462	2.234725	2.518485
C	-0.415345	1.016874	3.403854
H	0.521289	1.134910	3.959959
H	-1.205844	0.791107	4.125937
H	-0.425429	3.227522	2.925549
H	-1.688592	2.245114	2.155375

ts6c.log

SCF (wB97x) = -1475.09603530
E(SCF)+ZPE(0 K)= -1474.293875
H(298 K)= -1474.251613
G(298 K)= -1474.365288
Lowest Frequency = -389.3269cm-1

N	-1.660528	-1.003478	-0.752093
N	1.275929	-1.344778	-0.772864
C	0.975976	-2.054271	-1.852513
C	2.546422	-1.472109	-0.139158
C	-2.894238	-0.805948	-0.068637
C	3.578117	-0.555133	-0.415681
C	-1.551426	-1.796215	-1.805790
C	-3.342396	-1.764042	0.863616
C	0.184718	2.114660	0.099743
C	-3.587806	0.412348	-0.240098
C	2.720683	-2.479450	0.834430
C	-3.170557	1.401835	-1.318519
H	-2.097319	1.272763	-1.486322
C	3.398189	0.568819	-1.422791
H	2.335610	0.612217	-1.682150
C	-0.322603	-2.174498	-2.382205
H	-0.401289	-2.773652	-3.283315
C	-2.779446	-2.404636	-2.453649
H	-3.708267	-2.031478	-2.021189
H	-2.754533	-3.492340	-2.340840
H	-2.774471	-2.190399	-3.526251
C	3.960578	-2.590777	1.463283
H	4.117433	-3.373103	2.199751
C	0.008508	-0.201491	2.303599
H	0.993132	-0.208417	2.782714
H	-0.116750	-1.223216	1.893481
C	-2.654237	-3.110734	1.039931
H	-1.757486	-3.118837	0.413011
C	4.800191	-0.703704	0.243041
H	5.610405	-0.013996	0.023364
C	-4.709084	0.659604	0.551901
H	-5.251433	1.592291	0.433841
C	1.598566	-3.447327	1.183852
H	0.663660	-3.008978	0.818902
C	2.055587	-2.801784	-2.612749
H	2.275588	-2.258909	-3.538053
H	1.709884	-3.798672	-2.895569
H	2.980131	-2.893750	-2.042560
C	4.999972	-1.718430	1.167552
H	5.959283	-1.824018	1.664567
C	-4.475672	-1.473441	1.625850
H	-4.832696	-2.203851	2.346747
C	-5.150572	-0.269912	1.485202
H	-6.023990	-0.058746	2.094131
C	4.184094	0.292619	-2.712088
H	5.259890	0.250363	-2.508560
H	4.010731	1.084213	-3.448419
H	3.893278	-0.661078	-3.159595
C	-2.205992	-3.360111	2.485260
H	-1.491319	-2.606369	2.823384
H	-1.727665	-4.342006	2.563542
H	-3.055370	-3.352580	3.176329
C	3.792819	1.930604	-0.835851
H	3.300679	2.108750	0.124858
H	3.511299	2.737152	-1.520653
H	4.873558	2.001029	-0.673147
C	-3.411817	2.867017	-0.940828
H	-4.478994	3.113254	-0.926205
H	-2.940561	3.522894	-1.679445
H	-2.996360	3.110923	0.040892
C	1.780088	-4.792075	0.466206
H	1.819812	-4.664687	-0.618536
H	0.949404	-5.466877	0.698831
H	2.710766	-5.276729	0.781778
C	-3.570175	-4.252056	0.570916
H	-4.446746	-4.340087	1.222138
H	-3.037146	-5.208081	0.597218
H	-3.931344	-4.087289	-0.447280
C	1.455381	-3.657739	2.695416
H	2.326774	-4.165246	3.121942
H	0.582696	-4.282468	2.906153
H	1.325056	-2.704767	3.216265
C	-3.877688	1.081316	-2.644477
H	-3.637969	0.074843	-2.993878
H	-3.574914	1.790156	-3.422206
H	-4.964810	1.148228	-2.525643
H	1.135274	2.385827	-0.351726
H	-0.690985	2.591465	-0.335161
C	0.160382	1.876664	1.500215
H	1.115852	1.913255	2.020437
Mg	-0.049997	-0.046225	0.064738
C	-1.015063	2.318678	2.335416
H	-0.902526	3.397615	2.501466
H	-1.939010	2.174036	1.760631
C	-1.102638	1.555189	3.647747
C	-1.113763	0.070240	3.291405
H	-1.989248	1.847062	4.219635
H	-0.224554	1.786579	4.265801
H	-2.079332	-0.172387	2.827209
H	-1.041168	-0.544155	4.199600

ts6d.log

SCF (wB97x) = -1514.38973039
E(SCF)+ZPE(0 K)= -1513.558337
H(298 K)= -1513.514856
G(298 K)= -1513.630996
Lowest Frequency = -397.4794cm-1

N	-1.708982	-0.985750	-0.809480
N	1.227587	-1.316804	-0.708946
C	0.972767	-2.028866	-1.797863

C	2.476824	-1.431705	-0.031205
C	-2.967218	-0.799326	-0.168957
C	3.505815	-0.501594	-0.274334
C	-1.554353	-1.775644	-1.859885
C	-3.431273	-1.757100	0.756775
C	0.091733	2.125672	0.141252
C	-3.671739	0.407287	-0.372155
C	2.630713	-2.435327	0.949100
C	-3.227597	1.398533	-1.437949
H	-2.149066	1.272810	-1.572356
C	3.346531	0.614605	-1.293880
H	2.286051	0.669256	-1.559223
C	-0.302051	-2.149551	-2.383823
H	-0.340272	-2.750434	-3.286584
C	-2.753007	-2.384202	-2.560471
H	-3.699666	-2.025795	-2.154937
H	-2.722360	-3.473370	-2.465628
H	-2.712526	-2.151181	-3.628486
C	3.849459	-2.528761	1.621093
H	3.990501	-3.308078	2.364065
C	-0.123978	-0.221446	2.309441
H	0.927521	-0.244770	2.627703
H	-0.297935	-1.217384	1.846973
C	-2.750637	-3.107610	0.935581
H	-1.832764	-3.106284	0.340004
C	4.706110	-0.633139	0.426407
H	5.514625	0.066255	0.233428
C	-4.819774	0.646155	0.383719
H	-5.370926	1.570155	0.240620
C	1.513075	-3.420148	1.263611
H	0.587918	-3.011855	0.843776
C	2.076916	-2.792299	-2.505983
H	2.276491	-2.314148	-3.470511
H	1.760754	-3.817403	-2.713973
H	3.004145	-2.816437	-1.933607
C	4.886700	-1.643300	1.360102
H	5.829333	-1.735016	1.890624
C	-4.584906	-1.471079	1.489209
H	-4.953594	-2.199935	2.205811
C	-5.271468	-0.277768	1.317461
H	-6.163108	-0.071687	1.901240
C	4.133319	0.312094	-2.576857
H	5.206381	0.245725	-2.365341
H	3.983005	1.103309	-3.318687
H	3.822425	-0.636523	-3.021248
C	-2.352949	-3.390535	2.388588
H	-1.647075	-2.648271	2.765687
H	-1.880872	-4.376013	2.460733
H	-3.224161	-3.394462	3.051972
C	3.757071	1.979002	-0.724424
H	3.276158	2.171322	0.239262
H	3.474788	2.779829	-1.415489
H	4.839820	2.044508	-0.573503
C	-3.486534	2.861533	-1.064175
H	-4.554309	3.104458	-1.089771
H	-2.989331	3.522420	-1.781044
H	-3.110561	3.097435	-0.065640
C	1.762440	-4.775782	0.588178
H	1.864942	-4.669968	-0.494766
H	0.932946	-5.462830	0.786574
H	2.682010	-5.234573	0.968350
C	-3.653936	-4.235751	0.412054
H	-4.552090	-4.332729	1.031885
H	-3.125248	-5.194242	0.436843
H	-3.980351	-4.049248	-0.613974
C	1.299525	-3.599946	2.771139
H	2.160918	-4.075480	3.251512
H	0.432215	-4.240977	2.953817
H	1.122118	-2.638359	3.261249
C	-3.892010	1.077534	-2.785511
H	-3.638051	0.072778	-3.129375
H	-3.568565	1.789410	-3.552067
H	-4.982479	1.140240	-2.699758
H	1.097654	2.374170	-0.185459
H	-0.715032	2.575243	-0.434038
C	-0.129150	1.921300	1.524593
H	0.729478	2.012961	2.184845
Mg	-0.142047	-0.035718	0.092676
C	-1.462089	2.310043	2.129802
H	-1.706802	3.309250	1.747486
H	-2.244563	1.644778	1.747380
C	-1.045512	-0.158984	3.523325
H	-2.094821	-0.242871	3.203893
H	-0.868087	-1.019965	4.185523
C	-1.494910	2.348024	3.660732
C	-0.856695	1.124517	4.331803
H	0.220130	1.289317	4.469446
H	-1.274577	1.009389	5.337953
H	-0.996786	3.259251	4.013518
H	-2.543327	2.431455	3.969612

ts7a.log

SCF (wB97x) =	-1622.73806595
E(SCF)+ZPE(0 K)=	-1621.995251
H(298 K)=	-1621.954535
G(298 K)=	-1622.065250

Lowest Frequency = -430.6110cm-1

N	-1.768392	-1.140548	-0.865714
N	1.185566	-1.352473	-0.754583
C	0.966087	-2.053287	-1.852440
C	2.431484	-1.384844	-0.069490
C	-3.041715	-1.076468	-0.236698
C	3.462499	-0.494038	-0.420881
C	-1.564108	-1.868099	-1.952590
C	-3.456770	-2.132405	0.598504
C	0.044150	1.861678	0.110035
C	-3.826046	0.085357	-0.378592
C	2.575184	-2.263394	1.026936
C	-3.378587	1.223300	-1.281755
H	-2.300411	1.118901	-1.429305
C	3.300645	0.521832	-1.540484
H	2.258152	0.485740	-1.871209
C	-0.383317	1.354624	2.637922
H	0.143142	1.529116	3.575075
H	-1.311785	1.923431	2.585862
C	-0.294954	-2.181533	-2.470347
H	-0.300208	-2.754718	-3.391284
C	-2.738029	-2.474832	-2.694176
H	-2.775745	-3.552778	-2.510195
H	-2.605574	-2.332263	-3.769753
H	-3.692080	-2.043111	-2.391074
C	3.794773	-2.284154	1.702755
H	3.932982	-2.960717	2.539441
C	-0.511191	-0.104348	2.193270

H	0.062170	-0.817236	2.780327
H	-1.556070	-0.387482	2.036958
C	-2.610130	-3.380715	0.802414
H	-1.698278	-3.275014	0.207489
C	4.665289	-0.552851	0.287013
H	5.476857	0.115767	0.013169
C	-5.044555	0.152486	0.297098
H	-5.668465	1.034313	0.186053
C	1.425258	-3.163802	1.459829
H	0.498613	-2.616335	1.256642
C	2.095474	-2.801196	-2.534610
H	2.316678	-2.317527	-3.491371
H	1.792316	-3.828118	-2.752693
H	3.006485	-2.820761	-1.936772
C	4.840489	-1.447100	1.331498
H	5.783747	-1.483781	1.867621
C	-4.681661	-2.017018	1.258235
H	-5.017241	-2.825515	1.902323
C	-5.475887	-0.889074	1.108819
H	-6.427169	-0.818674	1.627065
C	4.196063	0.187666	-2.741993
H	5.254631	0.240764	-2.464218
H	4.027551	0.898742	-3.557278
H	4.003596	-0.818705	-3.120936
C	-2.182649	-3.536811	2.267099
H	-1.605941	-2.672925	2.608846
H	-1.561951	-4.431535	2.385317
H	-3.050181	-3.645102	2.926829
C	3.588976	1.948328	-1.050935
H	3.013984	2.189940	-0.152767
H	3.331983	2.676884	-1.826693
H	4.649432	2.082330	-0.811958
C	-3.622970	2.602988	-0.661159
H	-4.689306	2.848403	-0.609370
H	-3.140299	3.377023	-1.266209
H	-3.215817	2.659549	0.353093
C	1.369507	-4.462913	0.643836
H	1.223270	-4.264831	-0.420100
H	0.536044	-5.088151	0.983090
H	2.295902	-5.035708	0.762238
C	-3.341539	-4.642548	0.323566
H	-4.219055	-4.850838	0.945414
H	-2.679803	-5.513055	0.379667
H	-3.686580	-4.539408	-0.708713
C	1.451300	-3.483309	2.957903
H	2.270049	-4.163573	3.216451
H	0.520324	-3.975151	3.251550
H	1.561938	-2.577260	3.561802
C	-4.045250	1.120493	-2.660839
H	-3.799091	0.174558	-3.151306
H	-3.711564	1.935107	-3.312028
H	-5.135783	1.180587	-2.571135
H	0.861652	2.151203	-0.541388
H	-0.832139	2.504391	0.064454
Zn	-0.257803	-0.202275	-0.002514
C	0.425026	1.386360	1.415266
H	1.467058	1.096982	1.558417

ts7b.log

SCF (wB97x) = -1662.03516928
E(SCF)+ZPE(0 K)= -1661.262455
H(298 K)= -1661.220827
G(298 K)= -1661.333636
Lowest Frequency = -412.5868cm-1

N	-1.667907	-1.023006	-0.767034
N	1.270667	-1.347740	-0.730660
C	0.991234	-2.042012	-1.828040
C	2.541763	-1.491817	-0.102480
C	-2.907784	-0.881533	-0.080121
C	3.614298	-0.653530	-0.455207
C	-1.533683	-1.783818	-1.837136
C	-3.265759	-1.843051	0.889178
C	0.041693	1.992811	-0.094903
C	-3.710276	0.256038	-0.298181
C	2.684762	-2.461175	0.914410
C	-3.368870	1.275218	-1.373707
H	-2.319579	1.127580	-1.643865
C	3.449920	0.489551	-1.443733
H	2.425089	0.456547	-1.825602
C	-0.289391	-2.147190	-2.395990
H	-0.349390	-2.720691	-3.313550
C	-2.747537	-2.338369	-2.555186
H	-2.644627	-3.416287	-2.701937
H	-2.808641	-1.881215	-3.548146
H	-3.677107	-2.137817	-2.022250
C	3.941194	-2.630264	1.495814
H	4.077805	-3.379908	2.268234
C	-0.126453	-0.197249	2.200528
H	0.797746	-0.706446	2.482284
H	-0.883572	-0.940749	1.925398
C	-2.437669	-3.101757	1.112455
H	-1.468973	-2.953559	0.625638
C	4.850593	-0.854777	0.162305
H	5.690034	-0.219670	-0.107853
C	-4.871870	0.407622	0.460450
H	-5.505381	1.274181	0.296034
C	1.493957	-3.289780	1.382112
H	0.603528	-2.673221	1.230671
C	2.108563	-2.782521	-2.532712
H	2.794493	-2.064294	-2.991132
H	1.718162	-3.436866	-3.312518
H	2.696492	-3.378177	-1.830645
C	5.023745	-1.846336	1.115469
H	5.994134	-1.996406	1.578462
C	-4.438447	-1.647474	1.619736
H	-4.733500	-2.384238	2.361088
C	-5.237639	-0.531635	1.414658
H	-6.145655	-0.396359	1.993957
C	4.407853	0.383656	-2.637658
H	5.452025	0.469767	-2.318268
H	4.213728	1.187998	-3.354335
H	4.302476	-0.570117	-3.162044
C	-2.172171	-3.394456	2.593826
H	-1.662516	-2.562669	3.088467
H	-1.539862	-4.283476	2.684953
H	-3.097763	-3.600306	3.141137
C	3.641420	1.835782	-0.729483
H	2.996700	1.910426	0.150632
H	3.406772	2.668565	-1.400765
H	4.676681	1.957466	-0.392495
C	-3.535626	2.723664	-0.896514
H	-4.590255	2.986726	-0.761838
H	-3.122065	3.413753	-1.638820
H	-3.024100	2.900217	0.053673

C	1.295666	-4.568214	0.555752
H	1.064445	-4.344886	-0.488411
H	0.460109	-5.151007	0.959767
H	2.193114	-5.196147	0.585790
C	-3.108129	-4.316841	0.453889
H	-4.071705	-4.530241	0.929981
H	-2.476002	-5.205425	0.553568
H	-3.293358	-4.148134	-0.609026
C	1.566923	-3.630414	2.874504
H	2.347986	-4.367967	3.088399
H	0.618643	-4.061097	3.206094
H	1.763710	-2.740607	3.479783
C	-4.215260	1.039385	-2.633769
H	-4.075931	0.030560	-3.028822
H	-3.944561	1.753252	-3.418673
H	-5.280625	1.166931	-2.411446
H	0.935314	2.383547	-0.571588
H	-0.893706	2.417963	-0.445990
C	0.115975	1.682035	1.308962
H	1.105004	1.760012	1.762460
Zn	-0.059367	-0.053019	-0.060788
C	-1.008099	1.930278	2.294795
C	-0.689670	0.776925	3.241069
H	0.085262	1.071196	3.956920
H	-1.543065	0.396180	3.809830
H	-1.010092	2.950862	2.698462
H	-1.970719	1.755626	1.799751

ts7c.log

SCF (wB97x) = -1701.35299207
E(SCF)+ZPE(0 K)= -1700.550339
H(298 K)= -1700.508099
G(298 K)= -1700.621334
Lowest Frequency = -410.0806cm-1

N	-1.676662	-0.974554	-0.728569
N	1.268255	-1.321395	-0.740878
C	0.968116	-2.020244	-1.823964
C	2.529411	-1.449892	-0.091057
C	-2.908359	-0.785685	-0.040433
C	3.565115	-0.536311	-0.362133
C	-1.557854	-1.748416	-1.793152
C	-3.329869	-1.740957	0.907165
C	0.171416	1.983765	-0.063914
C	-3.634713	0.408376	-0.234492
C	2.696125	-2.460802	0.879208
C	-3.247412	1.394710	-1.326283
H	-2.191559	1.230473	-1.556906
C	3.404891	0.570358	-1.390925
H	2.352009	0.596381	-1.685683
C	-0.326615	-2.120662	-2.367501
H	-0.401952	-2.707034	-3.277062
C	-2.784642	-2.321670	-2.473901
H	-3.712384	-2.021661	-1.986200
H	-2.729596	-3.413401	-2.488279
H	-2.808433	-1.985198	-3.514907
C	3.926319	-2.564311	1.528538
H	4.076685	-3.347928	2.264998
C	0.002092	-0.305936	2.205749
H	1.012010	-0.327240	2.624993
H	-0.190945	-1.317685	1.821764
C	-2.606455	-3.067408	1.096593
H	-1.668140	-3.022197	0.535332
C	4.776693	-0.677225	0.316957
H	5.588949	0.012283	0.104032
C	-4.766396	0.635075	0.548904
H	-5.336456	1.548672	0.410625
C	1.583847	-3.450056	1.197212
H	0.650733	-3.027615	0.811813
C	2.048606	-2.771855	-2.577330
H	2.273821	-2.228331	-3.501049
H	1.700452	-3.767415	-2.861701
H	2.970480	-2.866190	-2.003346
C	4.964748	-1.685228	1.251160
H	5.915822	-1.784125	1.765141
C	-4.475420	-1.472212	1.658770
H	-4.816797	-2.201755	2.387687
C	-5.185036	-0.291445	1.495087
H	-6.068478	-0.097425	2.095307
C	4.238858	0.284022	-2.647629
H	5.308055	0.261392	-2.409003
H	4.078374	1.060166	-3.403113
H	3.976923	-0.681456	-3.088147
C	-2.255584	-3.356538	2.560940
H	-1.601300	-2.589781	2.980910
H	-1.741516	-4.320161	2.635706
H	-3.151556	-3.417081	3.187324
C	3.765554	1.944352	-0.810662
H	3.234594	2.133102	0.127070
H	3.505098	2.738170	-1.518426
H	4.838537	2.025964	-0.606448
C	-3.417258	2.861781	-0.915392
H	-4.472674	3.140051	-0.824151
H	-2.976711	3.514549	-1.675650
H	-2.929918	3.078414	0.039030
C	1.810640	-4.787349	0.478130
H	1.877994	-4.653738	-0.604395
H	0.987018	-5.479091	0.684574
H	2.741459	-5.255862	0.816855
C	-3.444491	-4.222629	0.526377
H	-4.369038	-4.348362	1.100809
H	-2.886993	-5.163702	0.576067
H	-3.724271	-4.044214	-0.514302
C	1.408800	-3.674630	2.703318
H	2.279691	-4.168875	3.146159
H	0.544117	-4.318202	2.888648
H	1.248034	-2.729179	3.228744
C	-4.050109	1.118024	-2.607029
H	-3.889784	0.100433	-2.969754
H	-3.756786	1.811618	-3.401979
H	-5.123003	1.244023	-2.423300
H	1.106291	2.271682	-0.533160
H	-0.706571	2.501399	-0.440159
C	0.201472	1.733698	1.355149
H	1.185350	1.744858	1.821315
Zn	-0.069502	-0.041311	-0.002288
C	-0.904544	2.259760	2.233603
H	-0.723824	3.335219	2.357128
H	-1.861369	2.147938	1.708851
C	-0.960965	1.534622	3.567239
C	-1.055973	0.048482	3.235442
H	-1.805439	1.876090	4.173734
H	-0.044045	1.737113	4.136843
H	-2.051793	-0.157994	2.822310
H	-0.961826	-0.558419	4.146582

ts7d.log

SCF (wB97x) = -1740.64545249
E(SCF)+ZPE(0 K)= -1739.813507
H(298 K)= -1739.770003
G(298 K)= -1739.886216
Lowest Frequency = -430.6401cm-1

N	-1.730242	-0.958277	-0.777971
N	1.215806	-1.301455	-0.663591
C	0.960853	-1.992939	-1.762666
C	2.456169	-1.425886	0.026977
C	-2.988841	-0.779034	-0.137470
C	3.491654	-0.501033	-0.205194
C	-1.564035	-1.716009	-1.847996
C	-3.414856	-1.724397	0.819723
C	0.061100	1.992136	-0.033965
C	-3.738912	0.390107	-0.381608
C	2.602017	-2.441919	0.994592
C	-3.340188	1.371096	-1.474141
H	-2.283665	1.198847	-1.698252
C	3.354459	0.609427	-1.233370
H	2.307717	0.639261	-1.548393
C	-0.308275	-2.085845	-2.366041
H	-0.340999	-2.663513	-3.283775
C	-2.759124	-2.262385	-2.603123
H	-3.705510	-2.015016	-2.121551
H	-2.681858	-3.348330	-2.698931
H	-2.761883	-1.847527	-3.615952
C	3.811717	-2.538261	1.682657
H	3.945225	-3.325838	2.418332
C	-0.100667	-0.314928	2.226054
H	0.959779	-0.336203	2.502431
H	-0.322287	-1.310315	1.807904
C	-2.678089	-3.040800	1.028282
H	-1.701465	-2.956839	0.541830
C	4.682207	-0.635439	0.511093
H	5.494405	0.062661	0.328429
C	-4.899667	0.604867	0.362793
H	-5.488945	1.499323	0.184457
C	1.491819	-3.444944	1.272783
H	0.572145	-3.045572	0.835074
C	2.066749	-2.752868	-2.470081
H	2.293049	-2.246269	-3.414122
H	1.739172	-3.765806	-2.715842
H	2.982176	-2.807694	-1.881216
C	4.849874	-1.647726	1.444761
H	5.784860	-1.740954	1.988439
C	-4.583490	-1.464295	1.536892
H	-4.928213	-2.184466	2.273203
C	-5.318166	-0.306150	1.323227
H	-6.221892	-0.119829	1.895058
C	4.210574	0.323145	-2.475125
H	5.274529	0.289730	-2.215202
H	4.071574	1.105034	-3.228966
H	3.948709	-0.637189	-2.926756
C	-2.433495	-3.378711	2.502386
H	-1.846925	-2.606950	3.003171
H	-1.887792	-4.324499	2.578699
H	-3.371734	-3.502837	3.053024
C	3.707636	1.981475	-0.643996
H	3.166303	2.166368	0.288665
H	3.454468	2.777288	-1.352156
H	4.778144	2.064075	-0.427600
C	-3.503616	2.838632	-1.061175
H	-4.558622	3.122822	-0.984078
H	-3.047908	3.491620	-1.812318
H	-3.030670	3.043613	-0.098453
C	1.778545	-4.789216	0.589256
H	1.905861	-4.669864	-0.489664
H	0.955075	-5.490858	0.759605
H	2.695479	-5.238425	0.986866
C	-3.443727	-4.191735	0.356115
H	-4.412409	-4.344216	0.845116
H	-2.876963	-5.125700	0.429945
H	-3.634806	-3.990269	-0.699877
C	1.245331	-3.645790	2.772472
H	2.102003	-4.115188	3.267145
H	0.383622	-4.301494	2.927264
H	1.043009	-2.693382	3.270218
C	-4.141552	1.105068	-2.757958
H	-3.999731	0.084628	-3.119300
H	-3.834001	1.793141	-3.552341
H	-5.212629	1.250658	-2.578203
H	1.047719	2.273832	-0.385944
H	-0.766711	2.466504	-0.553716
C	-0.100146	1.790145	1.379576
H	0.796024	1.884254	1.988106
Zn	-0.159771	-0.033633	0.034205
C	-1.383534	2.255935	2.035195
H	-1.600311	3.249795	1.623796
H	-2.211182	1.613809	1.714745
C	-0.979410	-0.160691	3.463113
H	-2.041452	-0.211384	3.183133
H	-0.809693	-1.014009	4.136963
C	-1.334756	2.355390	3.562101
C	-0.710035	1.128563	4.237316
H	0.376789	1.257281	4.325520
H	-1.088422	1.047755	5.262012
H	-0.783202	3.257452	3.853064
H	-2.361082	2.492671	3.921356

5. References

1 S. P. Green, C. Jones and A. Stasch, *Angew. Chem., Int. Ed.*, 2008, **47**, 9079–9083.

2 J. Spielmann, D. Piesik, B. Wittkamp, G. Jansen and S. Harder, *Chem. Commun.*, 2009, **23**, 3455–3456.

3 R. Y. Kong and M. R. Crimmin, *J. Am. Chem. Soc.*, 2020, **142**, 11967–11971.

4 L. Garcia, C. Dinoi, M. F. Mahon, L. Maron and M. S. Hill, *Chem. Sci.*, 2019, **10**, 8108–8118.

5 R. D. Holmes-Smith, S. R. Stobart, J. L. Atwood and W. E. Hunter, *J. Chem. Soc., Dalt. Trans.*, 1982, 2461–2468.

6 B. A. Shainyan and E. Kleinpeter, *Tetrahedron*, 2012, **68**, 114–125.

7 O. V. Dolomanov, L. J. Bourhis, R. J. Gildea, J. A. K. Howard and H. Puschmann, *J. Appl. Cryst.*, 2009, **42**, 339–341.

8 G. M. Sheldrick, SHELXTL v5.1 Bruker AXS: Madison WI., 1998.

9 G. M. Sheldrick, *Acta Cryst., C*. 2015, **71**, 3-8.

10 M. J. Frisch, G. W. Trucks, H. B. Schlegel, G. E. Scuseria, M. A. Robb, J. R. Cheeseman, G. Scalmani, V. Barone, B. Mennucci and G. A. Peterson, Gaussian 09; Revision D.01, Gaussian Inc. 2009.

11 J. Da Chai and M. Head-Gordon, *Phys. Chem. Chem. Phys.*, 2008, **10**, 6615–6620.

12 W. J. Hehre, R. Ditchfield and J. A. Pople, *J. Chem. Phys.*, 1972, **56**, 2257–2261.

13 P. C. Hariharan and J. A. Pople, *Theor. Chim. Acta*, 1973, **28**, 213–222.

14 T. Clark, J. Chandrasekar, W. Spitznagel, Gunther and P. von R. Schleyer, *J. Comput. Chem.*, 1983, **4**, 294–301.

15 M. Garçon, C. Bakewell, G. A. Sackman, A. J. P. White, R. I. Cooper, A. J. Edwards and M. R. Crimmin, *Nature*, 2019, **574**, 390–393.

16 M. Garçon, N. W. Mun, A. J. P. White and M. R. Crimmin, *Angew. Chem., Int. Ed.*, 2021, **60**, 6145–6153.

17 M. J. Butler, A. J. P. White and M. R. Crimmin, *Angew. Chem., Int. Ed.*, 2016, **55**, 6951–6953.

18 A. Hicken, A. J. P. White and M. R. Crimmin, *Angew. Chem., Int. Ed.*, 2017, **56**, 15127–15130.

19 J. Tomasi, B. Mennucci and R. Cammi, *Chem. Rev.*, 2005, **105**, 2999–3093.

20 K. Fukui, *Acc. Chem. Res.*, 1981, **14**, 363–368.

21 H. P. Hratchian and H. B. Schlegel, *Chapter 10 - Finding minima, transition states, and following reaction pathways on ab initio potential energy surfaces. Theory and Applications of Computational Chemistry – The First Forty Years*, 2005, **Ch. 10** 195-249.

22 R. Dennington, T. Keith and J. Milliam, GaussView 5.0, Semichem Inc., Shawnee Mission, KS., 2009.

23 M. Ernzerhof and G. E. Scuseria, *J. Chem. Phys.*, 1999, **110**, 5029–5036.

24 C. Adamo and V. Barone, *J. Chem. Phys.*, 1999, **110**, 6158–6170.

25 J. P. Perdew, K. Burke and M. Ernzerhof, *Phys. Rev. Lett.*, 1996, **77**, 3865–3868.

26 S. Grimme, S. Ehrlich and L. Goerigk, *J. Comput. Chem.*, 2011, **32**, 1456–1465.

27 E. D. Glendening, J. K. Badenhoop, A. E. Reed, J. E. Carpenter, J. A. Bohmann, C. M. Morales, C. R. Landis and F. Weinhold, NBO 6.0., 2013.

28 F. Weinhold and C. R. Landis, *Chem. Educ. Res. Pr.*, 2001, **2**, 91–104.

29 T. A. Keith, AIMALL (Version 19.10.12). TK Gristmill Software, Overland PArk KS, USA., 2019.

30 F. Cortés-Guzmán and R. F. W. Bader, *Coord. Chem. Rev.*, 2005, **249**, 633–662.

31 D. Svatunek and K. N. Houk, *J. Comput. Chem.*, 2019, **40**, 2509–2515.

32 E. R. Johnson, S. Keinan, P. Mori-Sánchez, J. Contreras-García, A. J. Cohen and W. Yang, *J. Am. Chem. Soc.*, 2010, **132**, 6498–6506.
